# Cytochrome P450 Monooxygenase CYP53 Family in Fungi: Comparative Structural and Evolutionary Analysis and Its Role as a Common Alternative Anti-Fungal Drug Target

**DOI:** 10.1371/journal.pone.0107209

**Published:** 2014-09-15

**Authors:** Poojah Jawallapersand, Samson Sitheni Mashele, Lidija Kovačič, Jure Stojan, Radovan Komel, Suresh Babu Pakala, Nada Kraševec, Khajamohiddin Syed

**Affiliations:** 1 Department of Health Sciences, Faculty of Health and Environmental Sciences, Central University of Technology, Bloemfontein, Free State, South Africa; 2 Conway Institute, School of Medicine and Medical Sciences, University College Dublin, Belfield, Dublin 4, Ireland; 3 Department of Molecular Biomedical Sciences, Jožef Stefan Institute, Jamova 39, 1000 Ljubljana, Slovenia; 4 Faculty of Medicine, Institute of Biochemistry, University of Ljubljana, Vrazov trg 2, SI-1000 Ljubljana, Slovenia; 5 National Institute of Chemistry, Hajdrihova 19, SI-1000 Ljubljana, Slovenia; 6 Department of Biochemistry, Sri Krishnadevaraya University, Anantapur, Andhra Pradesh, India; Woosuk University, Korea, Republic of

## Abstract

Cytochrome P450 monooxygenases (CYPs/P450s) are heme-thiolate proteins whose role as a drug target against pathogenic microbes has been explored because of their stereo- and regio-specific oxidation activity. We aimed to assess the CYP53 family's role as a common alternative drug target against animal (including human) and plant pathogenic fungi and its role in fungal-mediated wood degradation. Genome-wide analysis of fungal species revealed the presence of CYP53 members in ascomycetes and basidiomycetes. Basidiomycetes had a higher number of CYP53 members in their genomes than ascomycetes. Only two CYP53 subfamilies were found in ascomycetes and six subfamilies in basidiomycetes, suggesting that during the divergence of phyla ascomycetes lost CYP53 P450s. According to phylogenetic and gene-structure analysis, enrichment of CYP53 P450s in basidiomycetes occurred due to the extensive duplication of CYP53 P450s in their genomes. Numerous amino acids (103) were found to be conserved in the ascomycetes CYP53 P450s, against only seven in basidiomycetes CYP53 P450s. 3D-modelling and active-site cavity mapping data revealed that the ascomycetes CYP53 P450s have a highly conserved protein structure whereby 78% amino acids in the active-site cavity were found to be conserved. Because of this rigid nature of ascomycetes CYP53 P450s' active site cavity, any inhibitor directed against this P450 family can serve as a common anti-fungal drug target, particularly toward pathogenic ascomycetes. The dynamic nature of basidiomycetes CYP53 P450s at a gene and protein level indicates that these P450s are destined to acquire novel functions. Functional analysis of CYP53 P450s strongly supported our hypothesis that the ascomycetes CYP53 P450s ability is limited for detoxification of toxic molecules, whereas basidiomycetes CYP53 P450s play an additional role, i.e. involvement in degradation of wood and its derived components. This study is the first report on genome-wide comparative structural (gene and protein structure-level) and evolutionary analysis of a fungal P450 family.

## Introduction

Among microorganisms, fungi, the largest biological kingdom comprising diverse lower eukaryotic microorganisms, have acquired a special place owing to their ability to be pathogens for not only humans but also other animals and plants (Table 1). These lower eukaryotes develop or are constantly developing new strategies to adapt to diverse ecological niches. In order to develop novel drugs by identifying potential novel drug targets and harnessing their potentials for the production of human valuables, a large number of fungal genomes have been sequenced and many fungal genome sequencing projects are currently in progress. Efforts of the Broad Institute of MIT and Harvard (http://www.broadinstitute.org/), Wellcome Trust Sanger Institute (https://www.sanger.ac.uk/), and Joint Genome Institute (JGI) United States Department of Energy (US-DOE) (http://genome.jgi.doe.gov/programs/fungi/index.jsf) resulted in genome sequencing of a large number of fungal species.

**Table 1:** Genome-wide comparative analysis of CYP53 family in fungi.

Species	Lifestyle	CYP53 subfamily						Total count
		A	B	C	D	H	NS	
**Ascomycota**								
*Magnaporthe grisea*	Plant pathogen	1						1
*Neurospora crassa*	Model organism	1						1
*Neurospora discreta*	Distantly related to *Neurospora crassa*	1						1
*Fusarium graminearum*	Plant pathogen	3						3
*Fusarium solani f. batatas (Nectria haematococca)*	Plant pathogen and animal pathogen (opportunistic human pathogen)	2						2
*Fusarium verticillioides*	Plant pathogen and animal pathogen (opportunistic human pathogen)	2						2
*Fusarium oxysporum*	Plant pathogen and animal pathogen (opportunistic human pathogen)	2			1			3
*Neosartorya fischeri*	Animal pathogen (including human)	1						1
*Aspergillus nidulans*	Model organism for study of eukaryotic cell biology	1						1
*Aspergillus fumigatus*	Animal pathogen (opportunistic human pathogen)	1						1
*Aspergillus terreus*	Human, animal and plant pathogen	1						1
*Aspergillus oryzae*	Economically important, used for fermentation	2						2
*Aspergillus flavus*	Plant and animal pathogen (human pathogen)	1						1
*Aspergillus niger*	Plant and animal pathogen (human pathogen)	1						1
*Aspergillus clavatus*	Animal pathogen (human pathogen)	1						1
*Coccidioides immitis*	Animal pathogen (human pathogen)	1						1
*Histoplasma capsulatum*	Animal pathogen (human pathogen)	0						0
*Uncinocarpus reesii*	Non-pathogen	1						1
*Mycosphaerella fijiensis*	Plant pathogen	1						1
*Zymoseptoria tritici* (formerly named as *Mycosphaerella graminicola*)	Plant pathogen	1						1
*Thielavia terrestris*	Non-pathogen	1						1
*Myceliophthora thermophila*	Non-pathogen	1						1
*Cochliobolus lunatus*	Plant and animal pathogen (human pathogen)	1						1
**Total count**		**28**			**1**			**29**
**Basidiomycota**								
*Phanerochaete chrysosporium*	Model white rot fungus – study of wood degradation			1				1
*Postia placenta*	Model brown rot fungus – study of wood degradation			1	7			8
*Ustilago maydis*	Plant pathogen			1				1
*Cryptococcus neoformans*	Animal pathogen (human)							0
*Cryptococcus gattii*	Animal pathogen (human)							0
*Laccaria bicolor*	Symbiotic fungus (ectomycorrhizas)							0
*Malassezia globosa*	Animal pathogen (human)							0
*Puccinia graminis*	Plant pathogen		1					1
*Sporobolomyces roseus*	Non-pathogen		1					1
*Phanerochaete carnosa*	Model white rot fungus - study of soft wood degradation			6			1	7
*Bjerkandera adusta*	Wood-degrading white rot fungus			1		7		8
*Ceriporiopsis subvermispora*	Wood-degrading white rot fungus			4				4
*Ganoderma sp.*	Wood-degrading white rot fungus			1				1
*Ganoderma lucidum*	Medicinal mushroom (wood-degrading white rot fungus)			1				1
*Phlebia brevispora*	Wood-degrading white rot fungus			1				1
*Agaricus bisporus*	Litter-degrading fungus			2				2
*Serpula lacrymans*	Model fungus known as dry rot fungus – study of dry wood degradation			1				1
*Stereum hirsutum*	Wood-degrading white rot fungus			1				1
*Trametes versicolor*	Wood-degrading white rot fungus			2				2
*Wolfiporia cocos*	Wood-degrading brown-rot fungus			9				9
*Auricularia delicata*	Wood-degrading white rot fungus	1		1				2
*Coniophora puteana*	Wood-degrading brown rot fungus	1		2				3
*Dacryopinax sp.*	Wood-degrading brown rot fungus			1				1
*Dichomitus squalens*	Wood-degrading white rot fungus			1				1
*Fomitiporia mediterranea*	Wood-degrading white rot fungus			9			1	10
*Fomitopsis pinicola*	Wood-degrading brown rot fungus			4				4
*Gloeophyllum trabeum*	Wood-degrading brown rot fungus			1				1
*Punctularia strigosozonata*	Wood-degrading white rot fungus	1		1				2
**Total count**		**3**	**2**	**52**	**7**	**7**	**2**	**73**
**Total CYP53 members in fungi**		**31**	**2**	**52**	**8**	**7**	**2**	**102**

Twenty-three species from ascomycota and 28 species from basidiomycota were used in this study. Identification of CYP53 members in fungal species was carried out as described in the “Materials and methods” section. If no CYP53 member was found in the species, the space was left blank. The abbreviation NS indicates a new subfamily. Fungal species capable of causing diseases in humans were indicated with the word “human” in the table.

Genome sequencing analysis of fungal species revealed the presence of a large number of cytochrome P450 monooxygenases (P450s) in their genomes, with some exceptions. P450s are heme-thiolate proteins ubiquitously present across the biological kingdoms [1]. In fungi P450s are known to be involved in both primary and secondary metabolic processes [2], [3] and in the degradation of xenobiotic compounds [4]. P450s have been explored as anti-fungal drug targets owing to their key role in fungal physiology through involvement in stereo- and regio-specific oxidation of substrates [5]. Among fungal P450s CYP51, also known as sterol 14α-demethylase, the highly conserved P450 across the biological kingdoms [6], is the primary target of conventional antifungal azole drugs [7]. CYP51 performs demethylation of lanosterol, a key step in biosynthesis of cell membrane ergosterol [6]. Studies have indicated that fungal organisms are developing resistance to azole drugs [8], [9]. Furthermore, the currently available anti-fungal drugs have limitations because of similar metabolic pathways between fungi and other organisms (mainly mammals) and hence researchers are in search of alternative novel fungal drug targets [10].

Research on fungal P450s revealed that the P450 family CYP53 can serve as a novel alternative anti-fungal drug target [11]. CYP53 family members are well known as benzoate para-hydroxylases that are involved in the detoxification of a benzoate molecule [12]. Benzoate is a naturally occurring anti-fungal plant material [13] and also a naturally occurring intermediate in the degradation of aromatic compounds in fungi [14]–[16]. Benzoate exhibits its toxicity by disruption of the membrane, inhibiting essential cellular processes, changing pH balance and inducing stress response in fungi [13], [17]. CYP53 P450-mediated para-hydroxylation of benzoate is the only known pathway in fungi that ultimately channels this toxic compound into the β-ketoadipate pathway [18]. Furthermore, the CYP53 gene was found to be essential for fungal species' survival [19]. The CYP53 gene-knock out fungal strain growth was found to be inhibited by the accumulation of toxic intermediate benzoate [19]. This clearly suggests that this P450 is critical in the survival of fungal species, by playing a key role in the detoxification of benzoate.

Considering the fungal resistance to the currently available drugs, especially CYP51 enzyme-based azoles [8], and a preliminary study suggesting that CYP53 P450 family members can serve as novel alternative fungal drug targets [11], in the present study we aimed to understand the role of CYP53 members in fungal physiology per se, performing comparative evolutionary and structural analysis of CYP53 members to check their distribution and structural conservation in fungi. In this way we can determine whether this P450 family can serve as a common drug target against a broad range of fungal pathogens. Furthermore, we also explored its role in adaptation of basidiomycetes to diverse ecological niches such as colonization on wood.

## Materials and Methods

### Genome data mining and annotation of CYP53 members

Fifty-one fungal species were selected for the analysis of CYP53 member P450s. As shown in Table 1, 23 species from ascomycota and 28 species from basidiomycota were included in this analysis. CYP53 members of the basidiomycete species, such as *Phanerochaete chrysosporium*, *Phanerochaete carnosa*, *Bjerkandera adusta*, *Ganoderma* sp., *Phlebia brevispora*, and *Ceriporiopsis subvermispora*, and ascomycete species, such as *Thielavia terrestris* and *Myceliophthora thermophila,* were retrieved from an author's contributed and original work that has been published and is publicly available [4], [20]-[25]. CYP53 members in the remaining 20 ascomycetes were obtained from the Cytochrome P450 Webpage [26]. Two basidiomycete species, namely *Agaricus bisporus* and *Serpula lacrymans* CYP53 members, were obtained from the Fungal Cytochrome P450 Database (FCPD) [27]. CYP53 members belonging to *Postia placenta* were taken from published literature [28].

To identify CYP53 members in the basidiomycete species, such as Wolfiporia cocos, Auricularia delicata, Coniophora puteana, Dacryopinax sp., Dichomitus squalens, Fomitiporia mediterranea, Fomitopsis pinicola, Gloeophyllum trabeum, Punctularia strigosozonata, Stereum hirsutum, and Trametes versicolor, genome data mining was performed as described by one of the authors in his recent publications [24], [29], with slight modifications. Blast analysis was performed at the respective species' genome data base that is publicly available [30], using P. chrysosporium CYP53C2 (protein ID: 130996). Considering the presence of CYP53 members in low copies (one or two numbers) in ascomycetes and basidiomycetes, the top 20 hits' proteins were selected for further analysis. The hit proteins were subjected to the NCBI Batch Web CD-Search Tool [31] to separate proteins belonging to the P450 superfamily. This software groups the proteins into different superfamilies based on the conserved domain characteristics of the protein family. The proteins that are grouped under the P450 superfamily were selected for further assignment to the P450 family and subfamily. Assigning the family and subfamily names to the P450 proteins was performed in the same way as described by one of the authors in his recent study [24], [29]. Briefly, individual proteins were blasted against all named fungal P450s at the Cytochrome P450 Webpage [26]. A family and subfamily were assigned to the P450 proteins based on standard International P450 Nomenclature criteria, i.e. >40% homology for a family and >55% homology for a subfamily. Among the selected proteins those grouped under the CYP53 family were used in the analysis. The Cytochrome P450 Webpage [26] was visited to check for the presence of CYP53 members, if any, in the basidiomycetes Ustilago maydis, Cryptococcus neoformans, Cryptococcus gattii, Laccaria bicolor, Malassezia globosa, Puccinia graminis and Sporobolomyces roseus. A CYP53 member for Cochliobolus lunatus was obtained from one of an author's contributed work, which is publicly available [11].

### Phylogenetic analysis

Phylogenetic analysis of CYP53 members was carried out using the Molecular Evolutionary Genetics Analysis (MEGA) software [32] in the same way as described in one of the author's recent publications [24], [25]. Phylogenetic analysis was inferred using the minimum evolution method [33]. The minimum evolution method is widely used in P450 research, based on pairwise distance algorithms for the reconstruction of phylogenies [24], [25], [37]. In this study we used the minimum evolution method for phylogenetic analysis of CYP53 member P450s. The evolutionary distances were computed using the Poisson correction method [34] and are in the units of the amino acid substitution per site. The minimum evolution tree was searched using the close-neighbor-interchange algorithm [35]. The neighbor-joining algorithm [36] was used to generate the initial tree.

### Intron-exon analysis

Gene structure organization of CYP53 family members was carried out as described by an author in his recent publication [25]. Briefly, each CYP53 member gene was accessed at its genome data base at the JGI, US-DOE (http://genome.jgi.doe.gov/programs/fungi/index.jsf; accessed on 5 Feb, 2014) or Broad Institute of MIT and Harvard (http://www.broadinstitute.org/; accessed on 5 Feb, 2014). For each P450 the size of the exons and the location of introns were recorded. A schematic diagram showing horizontal lanes representing the exons and vertical lanes representing the introns' location were drawn. The length of the horizontal lane corresponds to the gene length. CYP53 members that showed high conservation in terms of the size of exons and the location of introns were shown in the figure.

### Analysis of homology

To identify the percentage homology between CYP53 members, we performed ClustalW2 multiple sequence analysis [38]. CYP53 members in FASTA format were included in the analysis and the result summary showing the percentage identity matrix was downloaded. After the file had been downloaded, the results were converted into table format and checked for the percentage homology between CYP53 members.

### Analysis of amino acid conservation

The number of amino acids conserved in CYP53 members across the fungi and between ascomycota and basidiomycota was determined using PROfile Multiple Alignment with predicted Local Structures and 3D constraints (PROMALS3D) [39]. PROMALS3D aligns multiple protein sequences and/or structures, with enhanced information from database searches, secondary structure prediction, 3D structures or user-defined constraints and it will also give a conservation index [40]. The conservation index follows numbers above 4, where 9 is the invariantly conserved amino acid across the input sequences.

### Homology modeling

3D-modelling of CYP53 member P450s namely; CYP53A (protein ID: 2107910) from *T. terrestris* (Tter) and CYP53C2 (protein ID: 130996) from *P. chrysosporium* (Pchr) was carried out as described by one of the authors in his publication [41], with slight modifications. The Basic Local Alignment Search Tool (BLAST) was used for selecting the closest homologues (template) available in the Protein Data Bank (www.pdb.org; accessed on 5 Feb, 2014). Among the hits at the PDB databank, recently crystallized full-length P450 protein CYP51 from *Saccharomyces cerevisiae* [42] was superior. Hence this P450 was used as a template. The coordinates of the crystal structures of CYP51 (PDB ID: 4LXJ) [42] were used as templates to build the models of CYP53 P450s. 3D-models of the Pchr and Tter were generated using the homology modeling program Modeller 9v11 [43]. The modelling was performed with default parameters using the “allHmodel” protocol to include hydrogen atoms and the “HETATM” protocol to include prosthetic group HEM (heme). The 3D-model's accuracy was validated using DFire [44], QMEAN [45], and Verify3D [46]. Heme-binding residues were identified using 3DLigandSite [47]. Structure alignment between the template and CYP53A of *T. terrestris* and the CYP53C2 of *P. chrysosporium* was performed using PROMALS3D [39]. P450 characteristic secondary structure annotations and substrate recognition sites (SRS) in modelled P450s were identified according to their alignment with the template P450s and standard SRS localization regions, as described in the literature [48], [49]. Protein models were visualized using PyMOL Molecular Graphics System, Version 1.7.1.1. Schrödinger, LLC (http://www.pymol.org/; accessed on 5 Feb, 2014).

### Active site cavity residues mapping

To identify the amino acid residues lining the active site cavity CASTp was used [50]. The CYP53A of *T. terrestris* (abbreviated as Tter) 3D-model generated in this study was used to predict the protein active site cavity using CASTp. The cavity showing the higher volume and covering the SRS regions was selected and the program automatically listed the residues lining the active site. The active site cavity structure and the residues lining the cavity were presented as a figure using PyMOL Molecular Graphics System, Version 1.7.1.1. Schrödinger, LLC.

## Results and Discussion

### CYP53 distribution in fungi

The CYP53 family is one of the P450 families apart from CYP51 and CYP61 that are conserved between the phyla ascomycota and basidiomycota [2], [27]. In this study we screened 51 fungal species belonging to ascomycota (23 species) and basidiomycota (28 species) for analysis of CYP53 family members in their genomes. Genome data mining of ascomycetes (23 species) and basidiomycetes (28 species) revealed the presence of one to nine copies of CYP53 members in their genomes (Table 1). The CYP53 family member count ranged from one to three in ascomycetes and one to ten in basidiomycetes. The basidiomycete species *F. mediterranea* showed the maximum number of CYP53 members (10 CYP53 P450s) in its genome. No CYP53 member was identified in the ascomycete *Histoplasma capsulatum* and in the basidiomycetes *C. neoformans*, *C. gattii* and *M. globosa* or the symbiotic *L. bicolor* (Table 1). Overall, ascomycete species showed a lower number of CYP53 member P450s in their genomes compared to basidiomycete species (Table 1), suggesting a possible duplication of CYP53 members after the phylum divergence. Moreover, our analysis revealed the complete absence of CYP53 member P450s in phyla zygomycota and chytridiomycota. Furthermore, in ascomycota only species belonging to subphyla pezizomycotina showed CYP53 members in their genomes and CYP53 member P450s were not found in species of the subphyla saccharomycotina and taphinomycotina, which is in accordance with the smaller size of the P450ome in relation to the growth form of the fungus [51]. Overall, contrary to the established assumption that this family is conserved in fungi, our study showed that CYP53 is not conserved across the fungal species. In future, further genome sequencing analysis of species belonging to chytridiomycota and zygomycota and the subphylum taphrinomycotina could be performed that may provide more information on the presence of this protein family in their genome. However, considering the life style and small size genomes of saccharomycotina species, the absence of CYP53 family members is expected.

Analysis of the CYP53 family suggested the dominance of specific CYP53 subfamilies in ascomycota and basidiomycota (Table 1). Ascomycete species showed only the CYP53A subfamily in their genomes, with the exception of *Fusarium oxysporum,* which showed a single copy CYP53 member belonging to the CYP53D subfamily (Table 1). In contrast to ascomycete species, basidiomycete species showed divergence in CYP53 subfamilies. Five subfamilies were observed in basidiomycetes, i.e. CYP53A, CYP53B, CYP53C, CYP53D, and CYP53H (Table 1). Our analysis of CYP53 members in basidiomycetes revealed the presence of two new CYP53 subfamilies in *P. carnosa* and *F. mediterranea*. Among the CYP53 subfamilies observed for basidiomycota, the CYP53C subfamily was dominant, with 52 members, followed by CYP53D (eight members) and CYP53H (seven members). A single copy of CYP53A members was found in *A. delicata*, *P. strigosozonata*, and *C. puteana* (Table 1). Considering the presence of CYP53A and CYP53D subfamilies in both phyla, one can assume that after the divergence of phyla, ascomycete species might have lost CYP53 subfamilies such as CYP53B, C and H. On the other hand, basidiomycete species enhanced CYP53 numbers in their genome, possibly by genome duplication of CYP53 members in view of the possible requirement of these P450 family members to adapt to diverse ecological niches.

### Phylogenetic analysis of CYP53 P450 family

**Figure 1:**
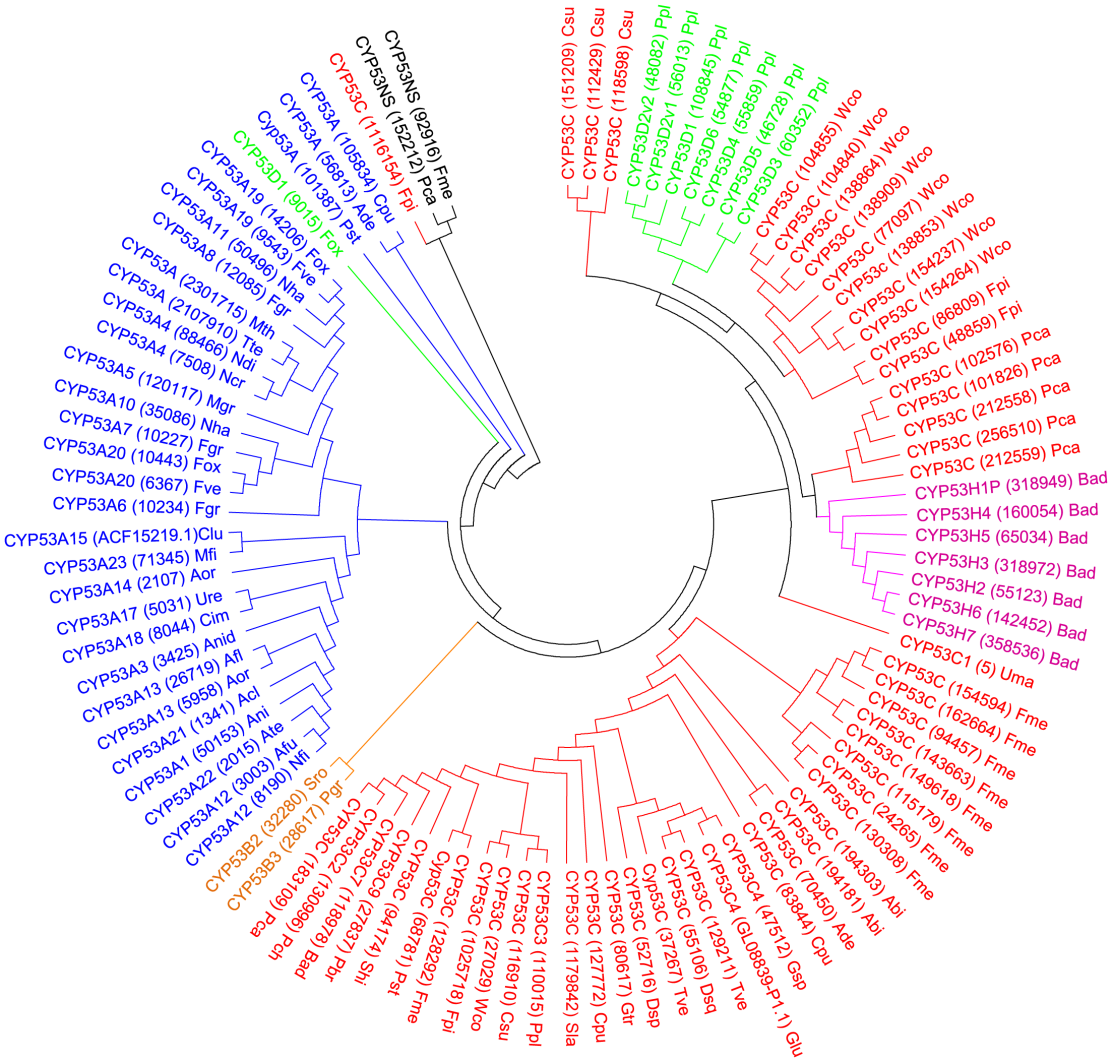
Phylogenetic analysis of CYP53 family in fungi. The tree was constructed with 101 CYP53 P450s belonging to six different CYP53 subfamilies. Phylogeny was inferred using the minimum evolution method [33] and the tree was constructed with MEGA (5.05) software [32]. For details on construction of the tree and the parameter employed for tree construction see the section “phylogenetic analysis” under “Materials and methods”. For ease of visual identity, the tree branch color, protein name, protein ID (parenthesis) and species name were presented in unique color as per sub-family. Fungal species' names were indicated with three letters, where the first letter is taken from the genus name and the other two letters from the species name. Abbreviations: Abi, *Agaricus bisporus*; Acl, *Aspergillus clavatus*; Ade, *Auricularia delicata*; Afl, Aspergillus flavus; Afu, *Aspergillus fumigatus*; Ani, *Aspergillus nidulans*; Aor, *Aspergillus oryzae*; Ate, *Aspergillus terreus*; Bad, *Bjerkandera adusta*; Cim, *Coccidioides immitis*; Clu, *Cochliobolus lunatus*; Cpu, *Coniophora puteana*; Csu, *Ceriporiopsis subvermispora*; Dsq, *Dichomitus squalens*; Fgr, *Fusarium graminearum*; Fme, *Fomitiporia mediterranea*; Fox, *Fusarium oxysporum*; Fpi, *Fomitopsis pinicola*; Fve, *Fusarium verticillioides*; Glu, *Ganoderma lucidum*; Gsp, Ganoderma sp.; Gtr, *Gloeophyllum trabeum*; Mfi, *Mycosphaerella fijiensis*; Mgr, *Magnaporthe grisea*; Mth, *Myceliophthora thermophila*; Ncr, *Neurospora crassa*; Ndi, *Neurospora discreta*; Nfi, *Neosartorya fischeri*; Nha, *Nectria haematococca*; Pbr, *Phlebia brevispora*; Pca, *Phanerochaete carnosa*; Pch, *Phanerochaete chrysosporium*; Pgr, *Puccinia graminis*; Ppl, *Postia placenta*; Pst, *Punctularia strigosozonata*; Shi, *Stereum hirsutum*; Sla, *Serpula lacrymans*; Sro, *Sporobolomyces roseus*; Tte, *Thielavia terrestris*; Tve, *Trametes versicolor*; Uma, *Ustilago maydis*; Ure, *Uncinocarpus reesii*; Wco, *Wolfiporia cocos*.

In order to understand the evolution of the CYP53 family and its distribution in fungi, we performed evolutionary analysis of the CYP53 family using the minimum evolution method [33]. Minimum evolution analysis of CYP53 members showed subfamily-specific and species-specific alignment/grouping of CYP53 members (Fig. 1), suggesting that after divergence of phyla (ascomycota and basidiomycota) CYP53 members have been subjected to phylum-specific amino acid changes in their structure. The most striking feature was that CYP53 members belonging to a particular basidiomycete species were grouped together (Fig. 1). This clearly indicates that paralogous evolution of CYP53 members, possibly *via* genome duplication, occurred in basidiomycete species. In a recently published study [25], we observed the same phenomenon of genome duplication of member P450s in basidiomycete species. Furthermore, we also showed that these P450 duplications were necessitated by the fungal species to adapt to diverse ecological niches [25]. Interestingly, CYP53D1 of *F. oxysporum* (ascomycete) did not align with its counterpart present in *P. placenta* (basidiomycete) (Fig. 1), suggesting that extensive changes specific to phyla might have occurred in their primary structure.

### High conservation of primary structure of CYP53 members in ascomycota

From the above study it is highly positive that after divergence of ascomycota and basidiomycota, CYP53 members have been subjected to phyla-specific changes or conservation in their primary structure. In order to understand these phyla-specific changes or conservations in CYP53 members, we followed two methods. Firstly we analyzed the percentage homology and secondly we deduced amino acids conserved in CYP53 members in both ascomycetes and basidiomycetes.

ClustalW2 analysis of CYP53 members revealed a high percentage homology among CYP53 members (Table 2) in ascomycota; some of the members showed >90% homology compared to CYP53 members in basidiomycota. The observed high percentage homology in CYP53 members of ascomycota (Table 2) might be due to the dominance of a single CYP53A subfamily. It is noteworthy that although the CYP53C subfamily is dominant in basidiomycota (Table 1), most of its members seem to be subjected to major amino acid changes, as the percentage homology between CYP53C members is not high with exception of a few P450s, as observed for CYP53A members for ascomycota (Table 2).

**Table 2:** Analysis of homology between CYP53 members in fungi.

CYP name	Species name	Homology (%)	CYP name	Species name
**Ascomycota**				
CYP53A4 (7508)	*Neurospora crassa*	98	CYP53A4 (88466)	*Neurospora discreta*
CYP53A (2107910)	*Thielavia terrestris*	91	CYP53A (2301715)	*Myceliophthora thermophila*
CYP53A19 (9543)	*Fusarium verticillioides*	98	CYP53A19 (14206)	*Fusarium oxysporum*
CYP53A8 (12085)	*Fusarium graminearum*	95	CYP53A19 (9543)	*Fusarium verticillioides*
CYP53A8 (12085)	*Fusarium graminearum*	95	CYP53A19 (14206)	*Fusarium oxysporum*
CYP53A20 (6367)	*Fusarium verticillioides*	99	CYP53A20 (10443)	*Fusarium oxysporum*
CYP53A7 (10227)	*Fusarium graminearum*	97	CYP53A20 (6367)	*Fusarium verticillioides*
CYP53A7 (10227)	*Fusarium graminearum*	97	CYP53A20 (10443)	*Fusarium oxysporum*
CYP53A12 (8190)	*Neosartorya fischeri*	98	CYP53A12 (3003)	*Aspergillus fumigatus*
CYP53A12 (3003)	*Aspergillus fumigatus*	94	CYP53A21 (1341)	*Aspergillus clavatus*
CYP53A13 (5958)	*Aspergillus oryzae*	99	CYP53A13 (26719)	*Aspergillus flavus*
**Basidiomycota**				
CYP53C4 (47512)	*Ganoderma* sp.	95	CYP53C4 (GL08839-P1.1)	*Ganoderma lucidum*
CYP53D3 (60352)	*Postia placenta*	95	CYP53D5 (46728)	*Postia placenta*
CYP53C (104855)	*Wolfiporia cocos*	67	CYP53C (154237)	*Wolfiporia cocos*

The percentage (%) homology between CYP53 members was obtained from the Cytochrome P450 Webpage [26] based on their highest hit to reference proteins and also estimated using ClustalW2 [38]. P450s showing more than 90% homology were selected and presented in the table. A detailed report on the percentage homology between identified proteins and hit proteins at Cytochrome P450 Webpage [26] was presented in Table S1. As shown in the table, a higher number of CYP53 members from ascomycota showed more than 90% homology, suggesting high conservation of the primary structure in ascomycete species CYP53 members compared to basidiomycete species CYP53 members. For each P450 protein IDs were shown in parenthesis.

**Figure 2:**
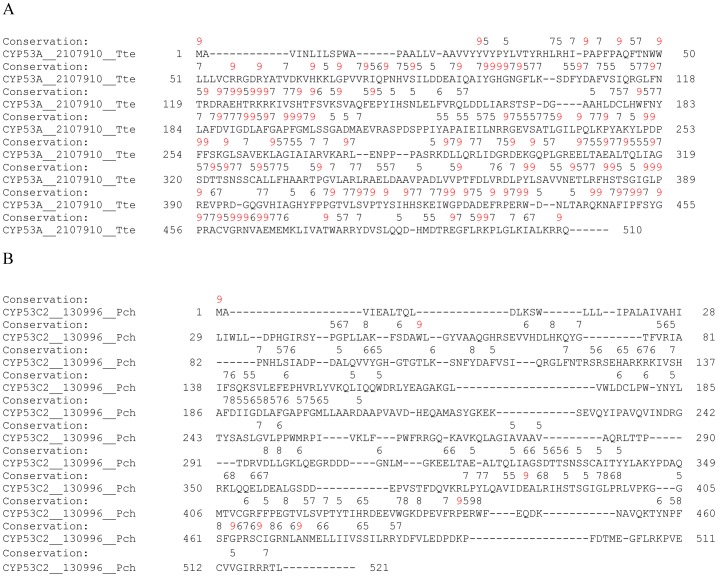
Analysis of amino acid conservations in CYP53 family members of ascomycota (A) and basidiomycota (B). Analysis of amino acid conservations was carried out using PROMALS3D [39]. CYP53A from *Thielavia terrestris* and CYP53C2 from *Phanerochaete chrysosporium* were presented as a representative of ascomycota (A) and basidiomycota (B) CYP53 members. The residues conserved in CYP53 members of ascomycota (A) and basidiomycota (B) are shown with the conservation index [40] on top of the amino acid residue. Complete alignment of CYP53 members of ascomycota and basidiomycota and a conservation index for the amino acids was presented in Fig. S1.

**Figure 3:**
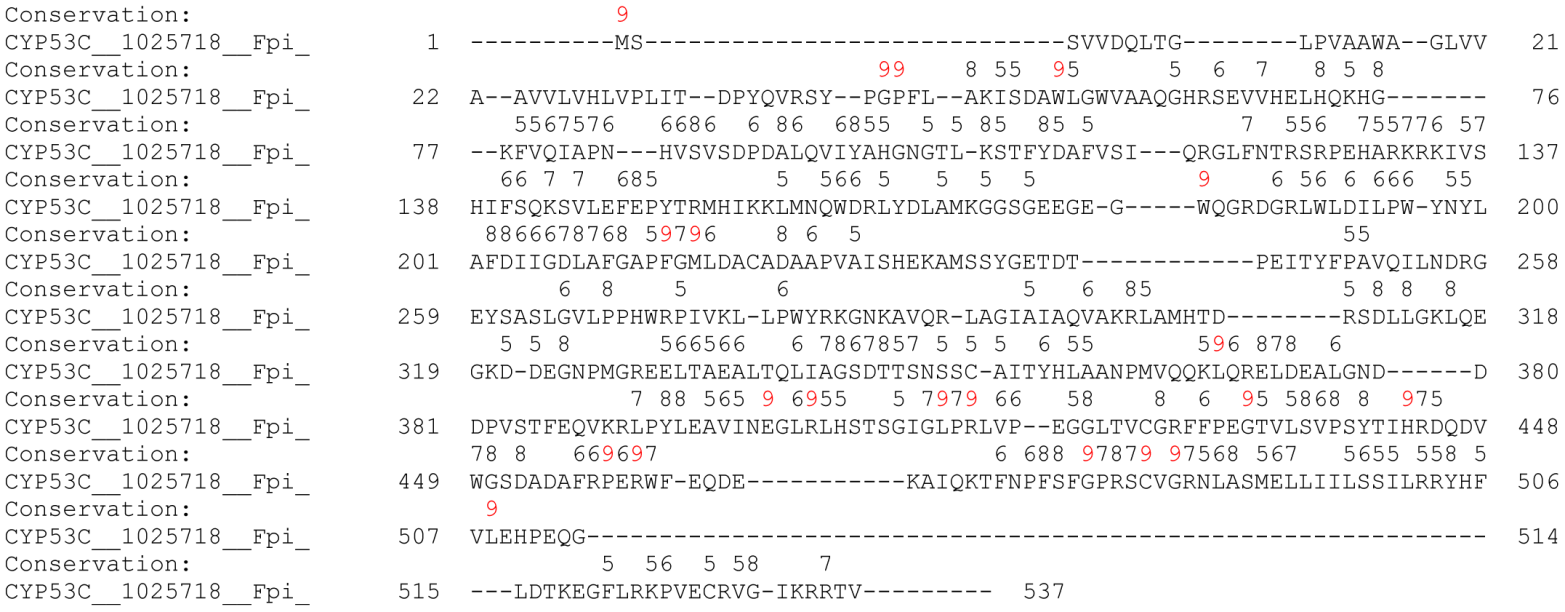
Analysis of amino acid conservations in CYP53C subfamily of basidiomycota. Analysis of amino acid conservations was carried out using PROMALS3D [39]. CYP53C from *Fomitopsis pinicola* is presented as a representative of CYP53C members. The residues conserved in CYP53C members are shown with the conservation index [40] on top of the amino acid residue. Complete alignment of CYP53C members of basidiomycota and a conservation index for the amino acids was presented in Fig. S1.

To link the high percentage homology observed for CYP53 members of ascomycetes towards conservation of amino acid in their primary structure, we performed amino acid conservation studies using PROMALS3D (Figures 2 and 3; Fig. S1). PROMALS3D analysis of CYP53 members across fungi suggested conservation of eight amino acids (Fig. S1A). Conservation of only eight amino acids in CYP53 members across fungi is understandable, considering the high diversity of CYP53 members across fungal species (five subfamilies and two new subfamilies). The most striking difference was observed in the number of amino acids conserved in the CYP53 members of ascomycota and basidiomycota (Figures 2 and 3 and Fig. S1). A hundred and three amino acids were found conserved in CYP53 members of ascomycota compared to CYP53 members of basidiomycota, which showed only seven amino acids conserved in their primary structure (Fig. 2). This strongly suggests that the observed high percentage homology between CYP53 members of ascomycota is due to the high conservation of amino acids in their primary structure.

One can argue that the high conservation of amino acids (103 amino acids) in CYP53 members of ascomycota (Fig. 2A) is due to the presence of a single CYP53A subfamily whereas five subfamilies and two new subfamilies exist in basidiomycota. To rule out this argument, we present two types of evidence. Firstly, we collected CYP53A members from ascomycete species belonging to 11 different genera (Table 1), suggesting the high diversity of host species, which should thus reflect in CYP53A primary structure as well. However, this was not true, as ascomycete CYP53 members showed high conservation in the primary structure (Fig. 2). Secondly, we estimated the number of amino acids conserved in the CYP53C subfamily alone (Fig. 3), the subfamily that is dominant in basidiomycota. Interestingly, our analysis revealed conservation of only 20 amino acids in CYP53C subfamily members in basidiomycota (Fig. 3), further strengthening our hypothesis that basidiomycota CYP53 members have been subjected to extensive primary structure changes. Further studies were carried out to map the location of conserved amino acids to extrapolate the effect of the conservation in CYP53 substrate specificity or catalytic activity, if any.

### Gene conservation and genome duplications of CYP53 members

**Figure 4:**
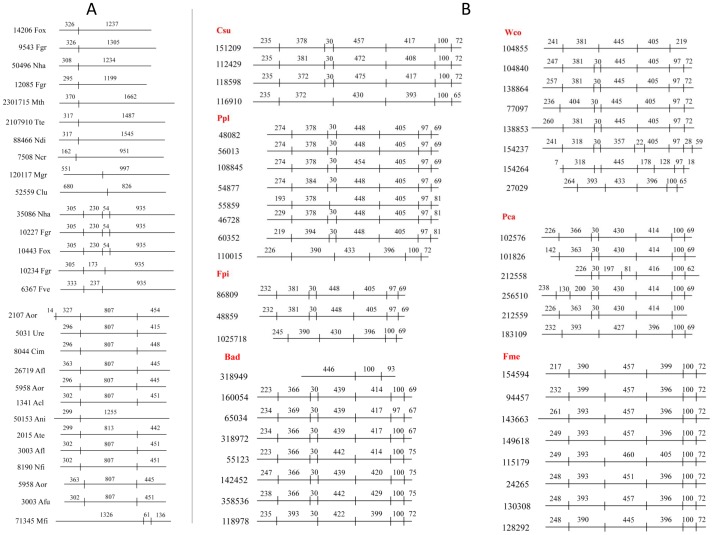
Gene-structure analysis of CYP53 family in (A) ascomycete species and (B) basidiomycete species. Intron-exon analysis was carried out as described in the “Material and methods” section. Horizontal lines indicate gene size and vertical lines indicate introns. For each CYP53 gene the size of the exons (base pairs) and protein ID from the JGI US-DOE [30] is shown in the figure. Abbreviations: Acl, *Aspergillus clavatus*; Afl, Aspergillus flavus; Afu, *Aspergillus fumigatus*; Ani, *Aspergillus nidulans*; Aor, *Aspergillus oryzae*; Ate, *Aspergillus terreus*; Bad, *Bjerkandera adusta*; Cim, *Coccidioides immitis*; Clu, *Cochliobolus lunatus*; Csu, *Ceriporiopsis subvermispora*; Fgr, *Fusarium graminearum*; Fme, *Fomitiporia mediterranea*; Fox, *Fusarium oxysporum*; Fpi, *Fomitopsis pinicola*; Fve, *Fusarium verticillioides*; Mfi, *Mycosphaerella fijiensis*; Mgr, *Magnaporthe grisea*; Mth, *Myceliophthora thermophila*; Ncr, *Neurospora crassa*; Ndi, *Neurospora discreta*; Nfi, *Neosartorya fischeri*; Nha, *Nectria haematococca*; Pca, *Phanerochaete carnosa*; Ppl, *Postia placenta*; Tte, *Thielavia terrestris*; Ure, *Uncinocarpus reesii*; Wco, *Wolfiporia cocos*.

The above study indicated high conservation of CYP53 members' primary structure (at a protein level) in ascomycetes compared to basidiomycetes. To gain insight into this aspect, we further analyzed the gene structure of CYP53 members (Fig. 4). Analysis of the size of exons and the location of introns indicated high conservation of the gene-structure in CYP53 members belonging to both fungal phyla ascomycota (Fig. 4A) and basidiomycota (Fig. 4B). Gene structure analysis suggested that some ascomycete species, such as *F. oxysporum*, *Fusarium solani f. batatas (Nectria haematococca*), *Fusarium verticillioides*, and *Fusarium graminearum,* contain two types of ortholog P450s in their genome. The first type contains a single intron and the second one contains three introns (Fig. 4A). Paralog P450s were found in *F. graminearum* (protein IDs: 10234 and 10227) and *Aspergillus oryzae* (protein IDs: 2107 and 5958), suggesting the genome duplication of these P450s. Overall, ascomycete species CYP53 members showed simple gene structure with single and triple introns (Fig. 4A).

It is evident from Fig. 4B, especially considering the exon sizes and location of introns, that basidiomycete species enriched CYP53 members in their genome by genome duplications (paralogous evolution). The high conservation in the size of exons and location of introns of CYP53 members of basidiomycetes strongly suggests that CYP53 members are genome-duplicated. In comparison to ascomycete species, CYP53 members of basidiomycete species showed more introns in their structure (Fig. 4B). An interesting discovery we made was that basidiomycete species selectively enriched a single type of CYP53 member in their genome (Fig. 4B). In support of this argument, we present a few examples: (i) in *P. placenta* we observed two orthologs, of which one duplicated seven times while no duplication was observed for the second ortholog (protein ID: 110015); (ii) in *W. cocos* three orthologs were found: one ortholog duplicated seven times whereas no duplications were observed for the remaining two orthologs (protein IDs: 138909 and 27029); (iii) in *F. pinicola*, *P. carnosa* and *F. mediterranea* two orthologs were found in their genomes; in these species one ortholog was duplicated whereas no duplication was observed for the second ortholog (protein ID: 1025718 (*F. pinicola*); protein ID: 183190 (*P. carnosa*); protein ID: 162664 (*F. mediterranea*); (iv) *B. adusta* showed three ortholgos; two orthologs (protein IDs: 318949 and 118978) have remained the same since the divergence of this species.

From the above results it is clear that the higher number of CYP53 members in basidiomycetes is due to the genome duplication of selective CYP53 members. Despite the conservation of gene structure and the paralogous evolution of CYP53 members in basidiomycete species, the low percentage of homology among them suggests that during the genome duplication events, extensive changes in the primary structure occurred. Most of the changes might be destined to acquire novel functions to serve fungal species (basidiomycete) to adapt to diverse ecological niches. In this direction, we further investigated whether amino acid changes play any role in CYP53 substrate specificity and/or catalytic activity.

### Structure and amino acid conservation analysis of CYP53 members

Primary structure analysis and gene-structure organization studies suggested that ascomycete CYP53 members are highly conserved and basidiomycete CYP53 members are subjected to evolutionary pressure to change their primary structure composition. To identify the role of amino acids conserved as observed for ascomycete CYP53 members (Fig. 2) and variants as observed for basidiomycete CYP53 members (Figures 2 and 3) in CYP53 substrate specificity and/or catalytic activity, we performed comparative CYP53 homology modeling studies to map these conserved/variant residue locations in the protein structure. In the present study we selected two CYP53 members, CYP53A from *T. terrestris* that was recently identified and characterized in an author's laboratory [24] as representative of ascomycete CYP53 P450s and CYP53C2 from the model white rot *P. chrysosporium* (abbreviated as Pchr) [4] as representative of basidiomycete CYP53 P450s. The 3D-models of CYP53 P450s were constructed and validated as described in materials and methods. As shown in Table 3 all the parameters employed in assessing the quality of the models were favorable suggesting that models of CYP53 P450s were of good quality.

**Table 3:** CYP53A Tter and CYP53C2 Pchr 3D-models validation.

CYP name^a^	Sequence Identity (%)^b^	Length^c^	Coverage^c^ (%)	dDFIRE^d^	DFIRE2d^d^	QMEAN6 score^e^	Average Verify3D score ± SD^f^
CYP53A Tter	16	510	100%	−1101.04	−849.785	0.57	0.23±0.22
CYP53C2 Pchr	18	519	99.6%	−1028.1	−789.42	0.57	0.32±0.24

Both CYP53 P450 models were based on the template CYP51 (PDB ID: 4LXJ) from *Saccharomyces cerevisiae* [42] and were generated using Modeller [43]. Abbreviations: Tter *Thielavia terrestris*; Pchr, *Phanerochaete chrysosporium*.

a Models were based on the template CYP51 (lanosterol 14α-demethylase) from *Saccharomyces cerevisiae* (PDB ID: 4LXJ) [42].

b Sequence identity between CYP53 P450s (CYP53A Tter and CYP53C2 Pchr) and the template CYP51 (PDB ID:4LXJ).

c Number of P450s amino acids modeled and their percentage compared to the full-length P450s.

d dDFire and DFIRE2 pseudo-energy (lower values signify a better model) [44].

e QMEAN6 composite score ranging from 0 to 1 (higher values signify a better model) [45].

f verify3D scores ranges from -1 (bad score) to +1 (good score). This program analyzes the compatibility of an atomic model (3D) with its own amino acid sequence (1D) [46].

**Figure 5:**
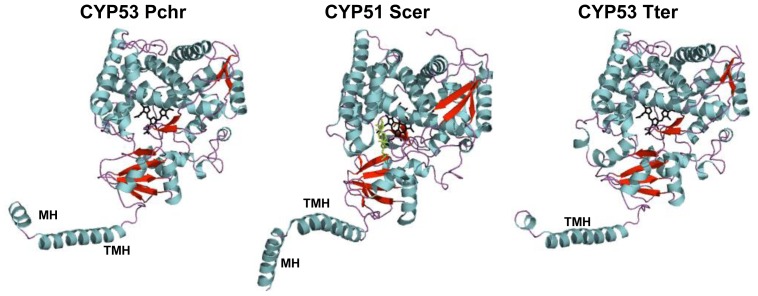
Structural analysis of CYP53 P450s. 3D-models for CYP53C2 from *Phanerochaete chrysosporium* (CYP53 Pchr) [4] and CYP53A from *Thielavia terrestris* (CYP53 Tter) [24] were constructed based on the template CYP51 from *Saccharomyces cerevisiae* (CYP51 Scer) (PDB ID: 4LXJ) [42]. The CYP51 Scer crystal structure was downloaded from the Protein Data Bank (www.pdb.org) with ID: 4LXJ. The overall structures of P450s were presented using PyMOL Molecular Graphics System, Version 1.7.1.1. Schrödinger, LLC (http://www.pymol.org/). The parameters used for validation of 3D-models of CYP53 Tte and CYP53 Pchr were presented in Table 3. The heme prosthetic group is shown in black color and the bound substrate for CYP53 Scer in green color. Alpha-helices and beta-strands are shown with blue and red. The membrane helix (MH) and the trans-membrane helix (TMH) are indicated in the models. P450 characteristics secondary structure notations are presented in Fig. 6.

**Figure 6:**
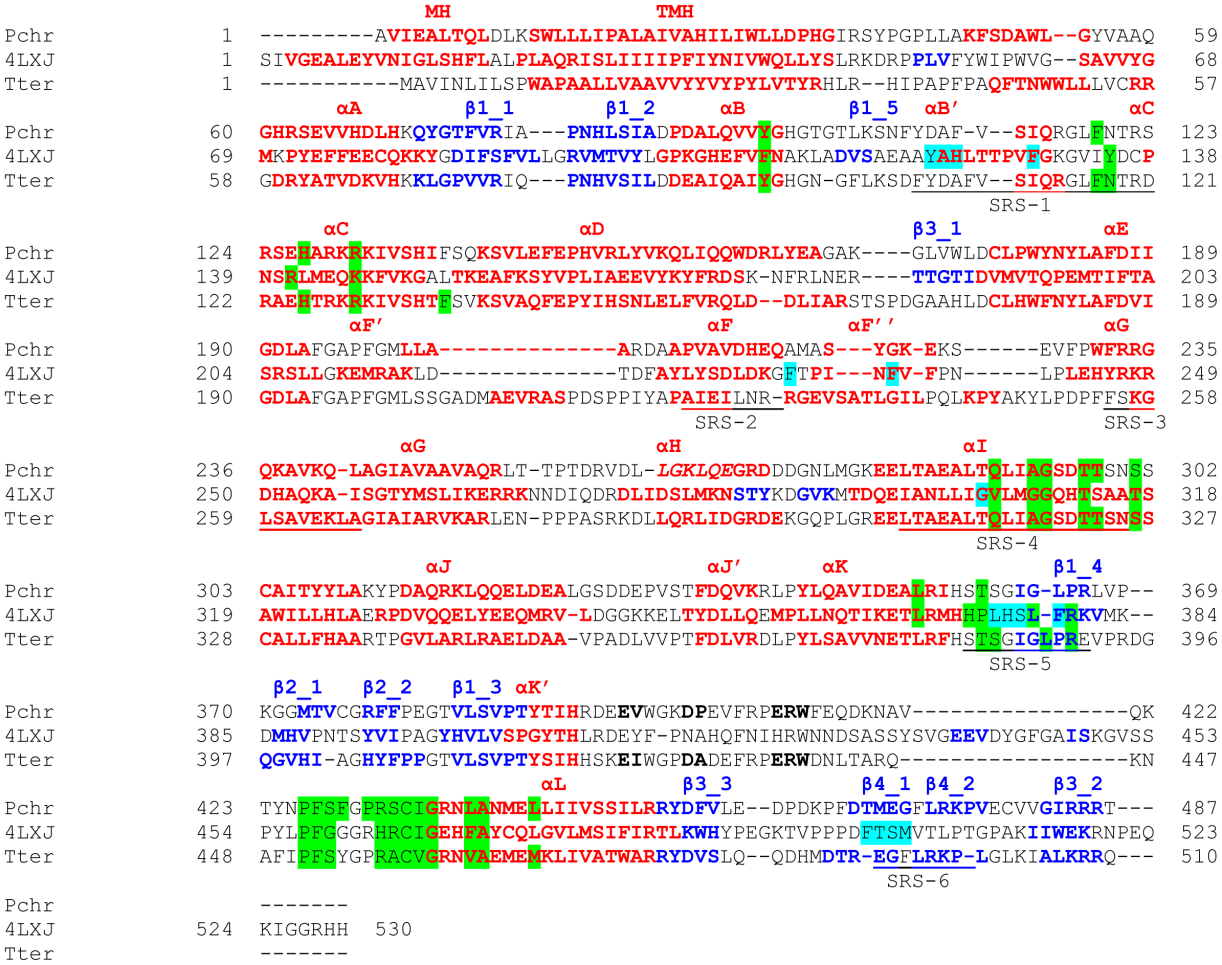
Structural alignment of CYP53C2 (Pchr) and CYP53A (Tter) models with CYP51 (4LXJ) using PROMALS3D [39]. 3D-models for CYP53C2 from *Phanerochaete chrysosporium* (Pchr) [4] and CYP53A from *Thielavia terrestris* (Tter) [24] were constructed using the template CYP51 (4LXJ) from *Saccharomyces cerevisiae* [42]. P450 characteristic notations for *α*-helices (shown in red font) and *β*-strands (shown in blue font) and SRS were mapped as per the template (4LXJ) [42] and published literature [48], [49]. Residues highlighted in green and turquoise appear in contact with the heme and substrate.

As shown in Fig. 5, CYP53A Tter and CYP53C2 Pchr showed all P450 motifs in the same way as CYP51 of *S. cerevisiae* [42]. Interestingly the membrane helix (MH) and transmembrane helix (TMH) found in CYP51 were also observed in CYP53C2 Pchr (Fig. 5). However, CYP53A Tter showed only the membrane helix (Fig. 5). This suggests that CYP53A Tter and CYP53C2 Pchr are biotopic membrane proteins with one transmembrane helix. A detailed secondary structure analysis including heme-binding residues, substrate binding residues and substrate recognition sites (SRS1-SRS6) is shown in Fig.6.

**Figure 7:**
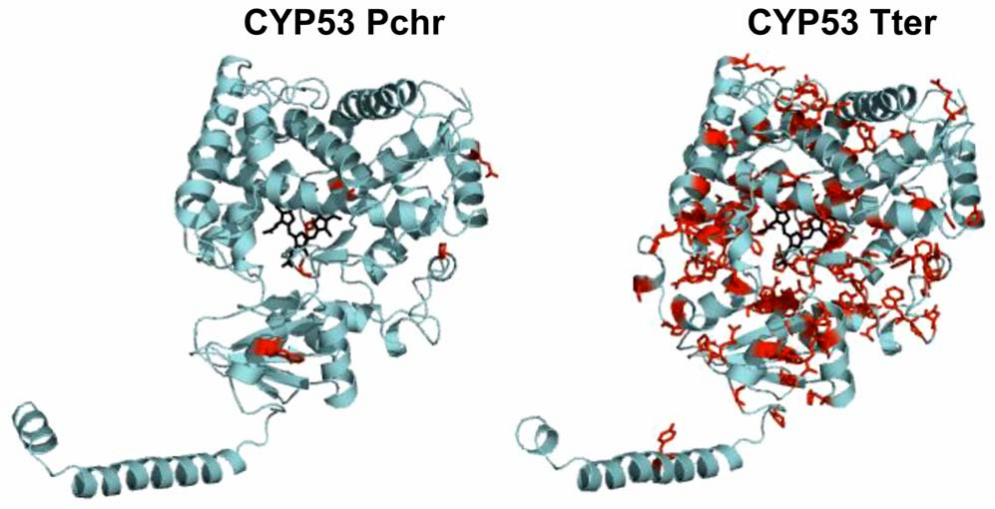
Analysis of amino acid conservations in CYP53 member P450s. 3D-models of CYP53C2 from *Phanerochaete chrysosporium* (Pchr) and CYP53A from *Thielavia terrestris* (Tter) as a representative of basidiomycota and ascomycota CYP53 member P450s are shown in the figure. The conserved residues among CYP53 members of two different phyla identified in this study (Fig. 2) are highlighted with red and shown in stick form. The heme-prosthetic group is presented in black. The models are presented using PyMOL Molecular Graphics System, Version 1.7.1.1. Schrödinger, LLC (http://www.pymol.org/).

**Figure 8:**
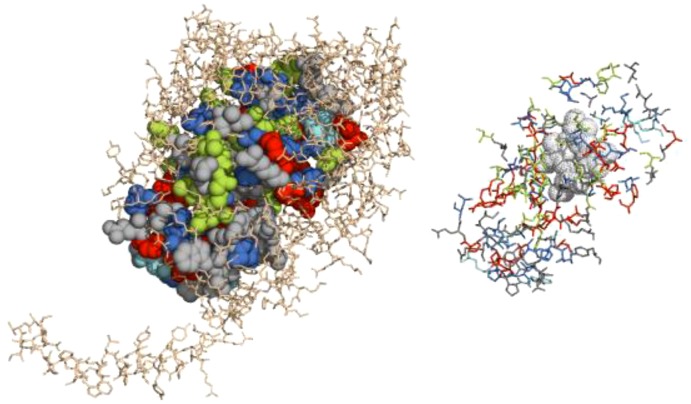
CYP53A Tte active site cavity mapping and analysis of the nature of the active site amino acids. The active site cavity and amino acid residues surrounding the cavity were identified using CASTp [50]. Conservation of amino acid residues in ascomycete species CYP53 P450s were identified using PROMALS3D [39] (Fig. 2). The CYP53A Tte model was used as a representative of ascomycete CYP53 members. The figure on the left shows the mapped active site cavity (space filled) and the protein backbone is presented in lines style, in the right-hand figure the conserved residues lining the cavity mapping. Different colors correspond their conservation index [40], 9 (conserved residues) – red, conservation index 7 – blue, conservation index 6 – cyan, conservation index 5 – green and residues with no conservation index – grey. The heme group is shown in grey dots. For details on the conserved nature of amino acid residues lining the active site cavity, see Table 4. The activity center is represented using PyMOL Molecular Graphics System, Version 1.7.1.1. Schrödinger, LLC (http://www.pymol.org/).

After successful construction and analysis of 3D-models of CYP53C2 Pchr and CYP53A Tter we proceeded to map the conserved amino acids observed for CYP53 members of ascomycota and basidiomycota (Fig. 2) to investigate the role of these residues in substrate specificity and/or catalytic activity. As shown in Fig. 7, CYP53 members of ascomycetes possess conserved amino acid residues throughout the protein structure, whereas CYP53 members of basidiomycetes show conservation at P450 signature motifs such as EXXR and CXG. In order to understand how many of these conserved amino acids are actually part of the active site cavity, we identified the active site cavity of CYP53 Tter using CASTp (Fig. 8 and Table 4) [50]. As shown in Fig. 8 and Table 4, among 125 amino acids lining the active site cavity, 35 (28%) are conserved (conservation index 9) and 62 amino acid residues (50%) are moderately conserved (conservation index 5–7) across the CYP53 members of ascomycetes. Overall, the high conservation of amino acids (78%) in the active site cavity and in the rest of the protein structure (Fig. 2) strongly suggests that the active site cavity and overall structure of CYP53 members of ascomycete species are highly conserved. Considering the structural conservation, any inhibitor developed against one of the CYP53 members could possibly act as common inhibitor against CYP53 members of ascomycete species and hence could act as a common anti-fungal (towards pathogenic ascomycetes) agent. On the other hand, basidiomycete CYP53 members showed much less conserved residue in their structure (Figures 2 and 7), suggesting basidiomycete members have been subjected to evolutionary pressure to acquire novel functions to help the organism adapt to diverse ecological niches.

**Table 4:** Analysis of amino acid conservation in CYP53A Tter active site cavity.

Conservation index	Amino acids	Number of Amino acids	Percent (%) contribution in active site cavity
9	R76, P79, G99, L101, K102, Y106, F117, R120, R122, H125, R129, F136, L192, P241, E311, T314, A318, T322, L370, S382, G387, L388, P389, R390,G409, S413, F449, P451, F452, C459, G461, R462, A465, E466, K498	35	28
7	L51, H81, N98, L116, N118, V132,V188, I189, L227, A235, T236, L243, L288, L313, G319, D321, N325, S326, V364, L378, P393, G398, V399, P408, V411, L412, S453, R457, A458, M467, E468, G494	32	26
6	Q78, R365, V392, F406, V464, M469	6	5
5	F100, D104, F105, S111, T119, F144, I148, A193, A221, A223, I224, I226, N228, E232, A310, Q315, I317, S320, T323, V374, T383, G385, V460, L496	24	19
None	N48, W49, L52, R57, Y61, V74, S103, I112, L152, A185, I219, E225, I239, L240, Q242, S371, N375, S384, I386, E391, R394, Q397, P407, T410,K470, L471, R492, F495	28	22
**Total**		**125**	**100**

Active site cavity residues were identified using CASTp [50] (Fig. 8). Conservation of amino acid residues in ascomycete species CYP53 P450s was identified using PROMALS3D [39] (Fig. 2). Conservation index 9 means amino acid is conserved. CYP53A Tter amino acid residues were used as a representative of ascomycete CYP53 members. CYP53A Tter P450 amino acids and their numbering are presented as representative of ascomycetes CYP53 P450s. Abbreviation Tter indicates *Thielavia terrestris*.

### Functional significance of CYP53 family and its potential role as a common anti-fungal drug target

CYP53 family members play a key role in fungal primary metabolism, i.e. the β-ketoadipate pathway [12], [19], and secondary metabolism, i.e. detoxification of phenolic compounds [28], [52]. The β-ketoadipate pathway is a convergent pathway for aromatic compound degradation [18] that is widely distributed in soil bacteria and fungi. Fungal-mediated degradation of aromatic compounds such as phenylalanine, toluene, and cinnamic acid leads to the formation of benzoate [14]–[16]. As part of the β-ketoadipate pathway CYP53 is involved in detoxification of this toxic compound and key intermediate molecule. CYP53 hydroxylates benzoate to 4-hydroxybenozate [12], the prime reaction in the benzoate metabolism that subsequently leads to protocatechuate as the ring fission substrate [53]. This reaction is critical for fungal organisms in order to detoxify the benzoate; to date this hydroxylation reaction carried out by CYP53 is the only way to detoxify this compound. Further support of CYP53's critical role in fungal primary metabolism can be obtained from a study where CYP53 deletion proved to be lethal for fungal organisms' survival (19). This suggests that the CYP53 family can serve as a novel alternative drug target against fungal pathogens, especially ascomycete pathogens. Results from this work showing high conservation of the primary and tertiary structure of CYP53 members (Figures 2 and 7) across the ascomycetes (consisting of animal and plant pathogen fungal species) indicate that any inhibitor developed against a CYP53 member could serve as a novel common drug against a large number of pathogenic ascomycete fungi. Results from authors laboratory showed inhibitors directed at this P450 effectively inhibited CYP53 activity [11] and also growth inhibition of different fungal species such as *C. lunatus*, *Aspergillus niger* and *Pleurotus ostreatus* [54]. Furthermore, this P450 family offers an advantage over the CYP51 family, the currently exploited target against fungal infections, as CYP53 does not have a homolog in higher eukaryotes. This will offer researchers the opportunity to design selective and potent inhibitors of pathogenic fungi.

Overall, the facts discussed above, such as (i) the critical role of CYP53 in fungal primary metabolism, (ii) high conservation of the primary and secondary structure of CYP53 members in ascomycetes and (iii) CYP53 not having any homolog in higher eukaryotes (advantage over CYP51 family), strongly support our hypothesis that the CYP53 family can be a potential novel alternative anti-fungal drug target and an inhibitor designed against this P450 family can serve as a common drug against pathogenic ascomycetes.

The most interesting aspect of the CYP53 family's role in basidiomycete fungi extends beyond detoxification of benzoate. Our study showed that most of the ascomycetes contain a single CYP53 member in their genomes, whereas basidiomycetes showed multiple CYP53 members (Table 1). Results from this study (Fig. 4B) revealed that the number of CYP53 members increase in basidiomycete species genomes by duplication of CYP53 members after speciation (paralogous evolution). Here we propose the critical role of these CYP53 members in basidiomycetes that forced basidiomycetes to enhance this P450 family member in their genomes.

First, basidiomycetes are well known for their role as bio-degraders of wood [55]. Wood is composed of many aromatic compounds, including benzoic acid derivatives and other phenolic compounds, among others eugenol, isoeugenol and guaiacol [56]. Most of these compounds are anti-fungal and toxic to fungi [13]. The multi-factorial phenomenon of toxicity of these compounds, including membrane disruption, inhibition of essential metabolic reactions, changes in pH homeostasis, and accumulation of toxic anions, has been proposed toward fungi [17]. If basidiomycete species want to colonize on wood they need an enzyme that can detoxify the benzoate molecule, as this molecule is an intermediate in detoxification of wood components comprising many aromatic compounds. Since there is an enormous need for successful wood colonization, wood-degrading basidiomycetes amplified their CYP53 members in their genomes.

Secondly, synthesis of aryl-metabolites, including veratryl alcohol by basidiomycete fungi, involves the formation of benzoate and para-hydroxybenzoic acid as intermediate molecules [14]. Veratryl alcohol is a secondary metabolite and plays a key role in lignin-peroxidase-mediated oxidation of wood components [57]. In a recent study, veratryl alcohol was shown to be the dominant extracellular ligninolytic oxidant in decaying wood [58]. The presence of a high number of CYP53 members and the generation of benzoate and para-hydroxybenzoate as an intermediate in the biosynthesis of veratryl alcohol suggest that in basidiomycete species CYP53 members also play a role in the generation of veratryl alcohol and help basidiomycete species directly in the degradation and subsequent colonization of wood.

Thirdly, demethylation of stilbene, a class of molecule found in plants, by CYP53D subfamily members from *P. placenta* (basidiomycete) [28] indicates that CYP53 family members play a critical role in the detoxification or degradation of plant compounds and help fungi in the colonization of wood. It is noteworthy that CYP53D members are present in the highest numbers (seven P450s) in *P. placenta* and are all evolved *via* paralogous evolution (Fig. 4B). This strongly indicates that *P. placenta* duplicated CYP53D members in its genome in order to colonize successfully on wood.

The above-mentioned role of CYP53 in wood-degrading basidiomycete species physiology (primary or secondary metabolism) is based on the available data and further experimentation would provide more insight into this aspect.

Collectively, the above results indicate that in ascomycetes the CYP53 role is limited to the detoxification of toxic molecules, whereas in basidiomycetes CYP53 plays an additional role, i.e. involvement in the generation of veratryl alcohol and degradation of wood-derived compounds.

## Conclusion

In this advanced scientific era, understanding of animal (including human) and plant pathogenic fungal organisms in terms of controlling their causative diseases and developing effective drugs is still poorly understood. Currently available drugs and drug targets are becoming ineffective because fungal species develop resistance. Genome sequencing analysis of the fungal species gives researchers the opportunity to look for novel drug targets against these pathogens and to search for novel enzymes for the generation of human valuables. The present study is such an example; we explored fungal genome sequencing results to understand the role of a P450 family (CYP53) in serving as a common drug target against pathogenic ascomycetes and in basidiomycetes, particularly in terms of the wood-degradation process. The CYP53 family plays a key role in the detoxification of the toxic molecule benzoate and this family has proven to be essential for the organism's survival. Our findings suggest that this P450 family can serve as a common anti-fungal (toward pathogenic ascomycetes) drug target in view of its highly conserved protein structure in ascomycetes. The most striking features of ascomycete CYP53 P450s were a large number of amino acids conserved in their active site cavity (78%), strongly indicating that any inhibitor developed for this family can act against a wide range of animal and plant pathogenic ascomycetes. We also identified that CYP53 P450s can play an additional role in basidiomycetes, i.e. in the generation of the wood-degrading oxidant veratryl alcohol and degradation of wood-derived compounds. This additional role of basidiomycetes seems to have enriched this P450 family by extensive duplication of CYP53 members in their genomes (paralogous evolution). During the duplication process extensive changes in the protein primary structure occurred to enhance/acquire novel functions, such as involvement in wood degradation.

## Supporting Information

Figure S1 **Comparative-structural analysis and subsequent identification and estimation of conserved amino acids of CYP53 family members in fungi.** Amino acid conservation was observed at three levels, i.e. (i) kingdom level (Fig. S1A), (ii) phylum level: ascomycota (Fig. S1B) and Basidiomycota (Fig. S1C) and (iii) family level (Fig. S1D). The first line in each block shows conservation indices for positions with a conservation index above 5. Each representative sequence has a magenta name and is colored according to PSIPRED [1] secondary structure predictions (red: alpha-helix, blue: beta-strand). A representative sequence and the immediate sequences below it with black names, if there are any, form a closely related group (determined by the option “Identity threshold”). Sequences within each group are aligned in a fast way. The groups are aligned using profile consistency with predicted secondary structures. The last two lines show a consensus amino acid sequence (Consensus_aa) and consensus-predicted secondary structures (Consensus_ss). Representative sequences have magenta names and they are colored according to predicted secondary structures (red: alpha-helix, blue: beta-strand). If the sequences are in aligned order, the sequences with black names directly below a representative sequence are in the same pre-aligned group and are aligned in a fast way. The first and last residue numbers of each sequence in each alignment block are shown before and after the sequences respectively. Consensus-predicted secondary structure symbols: alpha-helix: h; beta-strand: e. Consensus amino acid symbols are: conserved amino acids in bold and uppercase letters; aliphatic (I, V, L): *l*; aromatic (Y, H, W, F): *@*; hydrophobic (W, F, Y, M, L, I, V, A, C, T, H): *h*; alcohol (S, T): o; polar residues (D, E, H, K, N, Q, R, S, T): p; tiny (A, G, C, S): t; small (A, G, C, S, V, N, D, T, P): s; bulky residues (E, F, I, K, L, M, Q, R, W, Y): b; positively charged (K, R, H): **+**; negatively charged (D, E): **−**; charged (D, E, K, R, H): c.
(PDF)

Table S1 **Analysis of homology between CYP53 members in fungi.** Annotation of CYP53 P450 family members in fungi. Hit proteins were blasted at the Cytochrome P450 Webpage [26]. CYP53 members were assigned to subfamilies based on their percentage homology to reference proteins. The reference proteins were also included in the table.
(XLSX)
