# Supplementary material for: Cytochrome P450 Monooxygenase CYP53 Family in Fungi: Comparative Structural and Evolutionary Analysis and Its Role as a Common Alternative Anti-Fungal Drug Target
Source: PLoS One. 2014 Sep 15;9(9):e107209. doi: 10.1371/journal.pone.0107209 (PMC4164535; doi:10.1371/journal.pone.0107209)
Supplement: Figure S1 — Comparative-structural analysis and subsequent identification and estimation of conserved amino acids of CYP53 family members in fungi. Amino acid conservation was observed at three levels, i.e. (i) kingdom level (Fig. S1A), (ii) phylum level: ascomycota (Fig. S1B) and Basidiomycota (Fig. S1C) and (iii) family level (Fig. S1D). The first line in each block shows conservation indices for positions with a conservation index above 5. Each representative sequence has a magenta name and is colored according to PSIPRED [1] secondary structure predictions (red: alpha-helix, blue: beta-strand). A representative sequence and the immediate sequences below it with black names, if there are any, form a closely related group (determined by the option “Identity threshold”). Sequences within each group are aligned in a fast way. The groups are aligned using profile consistency with predicted secondary structures. The last two lines show a consensus amino acid sequence (Consensus_aa) and consensus-predicted secondary structures (Consensus_ss). Representative sequences have magenta names and they are colored according to predicted secondary structures (red: alpha-helix, blue: beta-strand). If the sequences are in aligned order, the sequences with black names directly below a representative sequence are in the same pre-aligned group and are aligned in a fast way. The first and last residue numbers of each sequence in each alignment block are shown before and after the sequences respectively. Consensus-predicted secondary structure symbols: alpha-helix: h; beta-strand: e. Consensus amino acid symbols are: conserved amino acids in bold and uppercase letters; aliphatic (I, V, L): l; aromatic (Y, H, W, F): @; hydrophobic (W, F, Y, M, L, I, V, A, C, T, H): h; alcohol (S, T): o; polar residues (D, E, H, K, N, Q, R, S, T): p; tiny (A, G, C, S): t; small (A, G, C, S, V, N, D, T, P): s; bulky residues (E, F, I, K, L, M, Q, R, W, Y): b; positively charged (K, R, H): +; negatively charged (D, E [file pone.0107209.s001.pdf]

<u>Fig. S1A</u>

Colored PROMALS3D alignment (sequences in aligned order)

```
Conservation:                   9
CYP53A__2107910__Tte         1  MAVI---------------------------NLI----------LSPWA------PAA--LLVAAVVYYV   25
CYP53A15__ACF15219.1_Clu     1  MFLT---------------------------SLL----------LTPYT-----------ILLLPVLFYL   22
CYP53A23__71345__Mfi         1  MFLS--------------------------------------FLFTPWA-----------LLASPFLFYL   21
CYP53A17__5031__Ure          1  MLLA--------------------------------------FLFNP--------YII--AGFTICYFYI   22
CYP53A18__8044__Cim          1  MLLS--------------------------------------FLFNP--------YVL--AGIFIFVFYI   22
CYP53A21__1341__Acl          1  MITE---------------------------------------ILTPQN-----TGYV--LLGLLTAYYI   24
CYP53A12__8190__Nfi          1  MITD---------------------------------------LLTPQN-----TGFI--LLGLIAAYYI   24
CYP53A12__3003__Afu          1  MITD---------------------------------------LLSLQN-----AGLI--LLGLIAVYYV   24
CYP53A22__2015__Ate          1  MIAD----------------------------------------LAAIN-----PAYL--LLAAVAAYYI   23
CYP53A13__5958__Aor          1  MIAE---------------------------------------LLTPTG-----AAYV--LTAAVIVYYI   24
CYP53A13__26719__Afl         1  MIAE---------------------------------------LLTPTG-----AAYV--LTAAVFVYYI   24
CYP53A1__50153__Anig         1  MLAL---------------------------------------LLSPYG------AYL--GLALLVLYYL   23
CYP53A3__3425__Anid          1  MITD---------------------------------------FLTPENITPERIALA--LLGLLAAYYV   29
CYP53A__2301715__Mth         1  MAIV---------------------------NFI----------LSPWA------PVA--LLAAAVVYYV   25
CYP53A4__88466__Ndi          1  MAII---------------------------SLL----------MSSWA------PVV--LLAGVAFYYL   25
CYP53A4__7508__Ncr           1  MAII---------------------------SLL----------MSPWA------PVV--LLAGVAFYYL   25
CYP53A19__9543__Fve          1  MAVV---------------------------DIL----------FTWWS------IPI--ATGVLIATYL   25
CYP53A19__14206__Fox         1  MAVV---------------------------DIL----------FTWWS------IPI--AACVLIATYL   25
CYP53A8__12085__Fgr          1  MAIV---------------------------DLL----------FTWWS------LPI--AAGLVAASYL   25
CYP53A11__50496__Nhe         1  MAIV---------------------------DLL----------LSWWT------LPI--GVAVLVGTYL   25
CYP53A20__6367__Fve          1  MALT---------------------------ELL----------ISPWA------PLA--LAVALVAWYI   25
CYP53A20__10443__Fox         1  MAIT---------------------------ELL----------ISPWA------PLA--LAVALVAWYI   25
CYP53A7__10227__Fgr          1  MAIT---------------------------ELL----------VSPWA------PVA--LVVAFVAWYI   25
CYP53A10__35086__Nhe         1  MALI---------------------------NLL----------ISPWA------PVA--LVVLFVGWYL   25
CYP53A5__120117__Mgrs        1  MAIV---------------------------NLV----------FTPLG-----LASL--GAFMLVAYYV   26
CYP53A14__2107__Aor          1  MDGDS--------------CPSCCSTCTSLNVF-----------LCPHI------PIL--TLGLLLVYYV   37
CYP53C__1025718__Fpi_        1  MS-------------------SVVDQLTGL--------------PVAAW------AGL--VVAAVVLVHL   29
Cyp53C__68781__Pst           1  MVLS----------------------------------------DPLTL------AGL--GLAAVVVAHI   22
CYP53C__80617__Gtr           1  ----------------------MLSGILNA--------------DVSNI------LIL--LVAVVAVAHI   26
CYP53C__127772__Cpu          1  MDSVIATLKDLVPINLNFDFDALLDRLRSI--------------SPSQL------AAG--VPVCLLLYFL   48
CYP53C__94174__Shi           1  MLA------------------SLVNAVTNV--------------DGKTC------LVA--VPVLLVLAHV   30
CYP53c__27029__Wco           1  MS--------------------GLLAPLNL--------------HPAAL------LLL--IPAAVLAVHF   28
CYP53C3__110015__Ppl         1  MS--------------------APAALFTS--------------NLVYG------LAV--IPVAVLLVHF   28
CYP53C__116910__Csu          1  ME----------------------FLHLSF--------------DWTTA------LLV--LAVGVALVHV   26
CYP53C__1179842__Sla         1  MNTI---------------LQLNPFQDFHF--------------SFTTA------LAG--VPIIFILVHV   33
CYP53C__55106__Dsq           1  MS--------------------LVDRLLNS--------------EPATW------AVV--GFTAVLLIHF   28
CYP53C4__GL08839-P1.1__Gl    1  MS--------------------LLDPLLAF--------------DFATW------AVI--GLSAIVLFHL   28
CYP53C4__47512__Gsp          1  MS--------------------FLNPLLAL--------------DFATW------AAL--GLAAIVLFHL   28
CYP53C__129211__Tve          1  MA--------------------LLGILSSL--------------DGPSW------AAL--VFAAVVLVHV   28
CYP53C9__27837__Pbr          1  MS-------------------SLLTSFSLD--------------NVTNL------LLV--IPGLLVLGHV   29
```

```
CYP53C7__118978__Bad        1  MAVL----------------DYVLSPLESL--------------GLASS------LLI--IPCLVLAGHF   32
CYP53C__183109__Pca         1  MA--------------------ILEVLAQL--------------NLTSW------LVL--IPALAIAAHV   28
CYP53C2__130996__Pch        1  MA--------------------VIEALTQL--------------DLKSW------LLL--IPALAIVAHI   28
CYP53C__128292__Fme         1  MGFTG--------------VSTLTNALARV--------------DLISL------AVA--VPLVLILGLI   34
Cyp53C__37267__Tve          1  MA--------------------IFELLASL--------------DLPSW------AAI--VLAAVVLVHL   28
CYP53C__70450__Ade          1  ME-----------------------DLLRL---------------AVYL------ALA--GSIGTTLFVL   24
CYP53C__52716__Dsp          1  MSSL---------------LDYFPTPFSSN--------------ALLLY------GGL--AVLLLVASHL   33
CYP53C__83844__Cpu          1  MDVQ----------------QLIQHLPQNV--------------DLTSA------AAA--SFAAFLAVHL   32
CYP53C__194303__Abi         1  MII-----------------ETFKNFVAQA--------------DTTTL------ACA--IPAAVVLFHV   31
CYP53C__194181__Abi         1  MI-LDR------------LSDTFLELYDKL--------------DLQIV------AFA--VPGTFLAFHL   35
CYP53C__143663__Fme         1  MI-------------------LIILRVFVDIG------------YRIWL------FLP--WIFLVVHLIP   31
CYP53C__130308__Fme         1  MIA------------------SIFYSTFFTKY------------LWICA------GLT--AGFLVVHVVP   32
CYP53C__24265__Fme          1  MIL------------------KILYSVFFTNY------------LWLCT------GLT--AAFFLVHVVP   32
CYP53C__149618__Fme         1  MSMS-----------------SGIVQELLNVT------------FWLWF------FLL--AIIFAVHLDA   33
CYP53C__94457__Fme          1  MV-------------------PNIAHNFLLGY------------SWILL------LLP--AIILVVHLVS   31
CYP53C__154594__Fme         1  -----------------------MQMISNIS-------------PWYFC------LLP--AAVLVLLVVP   26
CYP53C__115179__Fme         1  MLP------------------NILDALAQL--------------NTSQL------CAS--ALAVAAVVYL   30
CYP53C1__5__Uma_            1  MVETD-------------LVPRIGAAIQWSV-------------ESPAH------VVI--TLLGAVVLFH   36
CYP53B2__32280__Sro         1  MTQS----------------HVFTDFFEPTT-------------LAVYF------LAA--PLGAVFLYLF   33
CYP53B3__28617__Pgr         1  ML--------------------VIGLIA---------------AFLEYS------ILL--GLVGISCYYL   27
CYP53C__48859__Fpi          1  MS--------------------IVQQLTNFTRG-----------NPLLV------LAA--LPVVLVVAKV   31
CYP53C__86809__Fpi          1  MS--------------------IVQQLIDFTRG-----------NPLLV------LAA--LPVVLVVAKV   31
CYP53C__138909__Wco         1  MS--------------------LLRDITVQALPF--------VDKLIFW------LAL--IPIISIAVYT   34
CYP53C__104840__Wco         1  MP--------------------LIGDITVQTLPF--------VGISTIW------LALISITGIVTVICV   36
CYP53C__77097__Wco          1  MS--------------------LVDDTTAHVPS-----------YPAFW------IAL--IAITVFAVQF   31
CYP53C__138864__Wco         1  MS--------------------LLRDITVQALSF--------VDKLIFW------LAL--IPIIGIAMYI   34
CYP53C__104855__Wco         1  MS--------------------LLGDIAPQTLDY--------VGKLTFW------LAS--IPIVIIAAHI   34
CYP53C__154264__Wco         1  MP--------------------LLASITAYALDY--------MCMPTFW------LAL--IPAVLTTVYI   34
CYP53c__138853__Wco         1  MP--------------------LLDRITTHALDY--------VCMPTFW------LAL--IPAVLTTVYI   34
CYP53C__154237__Wco         1  M---------------------------------------------------------------------    1
CYP53D1__108845__Ppl        1  MDSSTPP---------LPPLGSIILAREGLTGLL--------LPGLSVL------ACL--IATVIASSIL   45
CYP53D6__54877__Ppl         1  MDSSTPP---------LPLLGGIILTREGLTGLL--------LPGLSVL------ACL--IATVIASLIL   45
CYP53D4__55859__Ppl         1  M--------------------------------------------LSVL------VCL--IPTVIASTFL   18
CYP53D2v1__56013__Ppl       1  MDSSTSL---------LPPLGSIILACEGLTSLV--------PLILSVM------VCL--IATVTISPTL   45
CYP53D2v2__48082__Ppl       1  MDSSTSL---------LPPLGSIILACEGLTSLV--------PLILSVL------VCL--VATVTISPTL   45
CYP53D5__46728__Ppl         1  MLT------------------FDEALNFST--------------ILSAL------ACL--SAGVITLLLF   30
CYP53D3__60352__Ppl         1  MLT------------------YDEALNFST--------------VLSAL------TCL--SAGVITLSLL   30
CYP53C__112429__Csu         1  M---------------------ILDVVHSVLERP----------L--LL------VVL--LPSVLLAVAL   29
CYP53C__151209__Csu         1  MLP-----------------AELVDCLPGS---------------LPLL------FGL--FLTALLAVFF   30
CYP53C__118598__Csu         1  MDW-----------------RMMIDGFTTLTSRETYTFSGFVSILLPLL------FLC--LSITLLLFPC   45
CYP53H3__318972__Bad        1  MDE-----------------NFAIPLS-----------------SGLKA-----------LVFIVLLTHV   25
CYP53H7__358536__Bad        1  MT-------------------LNVPLP-----------------TAVWV-----MFLLSSLPVLAILFHV   29
CYP53H6__142452__Bad        1  MAL-----------------SFGAPDP-----------------TTLAV------ISL--PLVFAILVHV   28
CYP53H4__160054__Bad        1  MDP-----------------AVVLILS-----------------TGLKA------AVA--LLAFAFLAHL   28
CYP53H2__55123__Bad         1  MDA-----------------AWGQIVT-----------------SGVKA------VLL--LISFVLLVHL   28
CYP53H5__65034__Bad         1  MD--------------------AIRDV------------------LLPF------GTV--VAISTLLAYL   24
CYP53C__101826__Pca         1  ---------------------------------------------------------------------M    1
CYP53C__256510__Pca         1  MEQV----------------RSLLPQL-----------------DLKLA------SVL--LVAAPLFAWA   29
CYP53C__102576__Pca         1  MEQS----------------HFILPQL-----------------DLKLI------TAI--LAASLLAAWI   29
CYP53C__212559__Pca         1  MEQT----------------HFLLPQL-----------------DLRLT------AGL--LAAALLAAWV   29
CYP53D1__9015__Fox          1  MS--------------------LDSLL-----------------LSPWA------PLA--VLLCVLLFYI   25
```

```
CYP53A6__10234__Fgr              1  MA--------------------MFTVL-----------------PLIWL------APL--GLISLFFYYI   25
Cyp53A__101387__Pst              1  MQN-----------------STLFSSATHNLP------------EAMQD------YTW--LVFLPAVVVG   33
CYP53A__105834__Cpu                 ----------------------------------------------------------------------
CYP53A__56813__Ade               1  MS-------------------------------------------SFWL-------CL--AAAALLV---   15
CYP53__1116154__Fpi              1  MP-------------------SLPLGL-----------------KIPQL-----SALE--ICGIVTALFL   27
CYP53NS__152212__Pca             1  MSG------------------TIVNLGLDLREW-----------LSPAR------VVL--CASALLIARV   33
CYP53NS__92916__Fme              1  MS-------------------DIIAPFLPVKRF-----------VASLP------FAA--CILCLALLKI   32
Consensus_aa:                       M......................................................hhh..h..hhhhhhh
Consensus_ss:                                             hhhhh                 hhhhh      hhh  hhhhhhhhhh


Conservation:                                        567  8    6  9               6   8   7           565
CYP53A__2107910__Tte            26  YPYLV--TYRHLRHI--PAPFPAQ--FTNWWL--LLVCRRGDRYATVDKVHKKLG---------PVVRIQ   78
CYP53A15__ACF15219.1_Clu        23  LPYLR---NWRIRDI--PAPFPAA--WTNLWL--LYQCRRGRRFLAVHEAHQKLG---------KLVRIQ   74
CYP53A23__71345__Mfi            22  LPFLR---NWSIRDV--PGPFLAK--FTTLWY--MYECRRCRRYYTVYKLHEKYG---------KFVRVQ   73
CYP53A17__5031__Ure             23  VPYLQ---RWDLRDI--PAPFPAS--LSHLWL--LYQSRKGKRYQAVHNAHGKYG---------KLVRIQ   74
CYP53A18__8044__Cim             23  VPYLR---LSYLRDI--PSPFAAG--FSNLWL--LYQCRRGKRYQAVHDAHKKYG---------KLVRIQ   74
CYP53A21__1341__Acl             25  IPYLQ---TWHLHDI--PSPGFAA--FSNLWL--LLQARQGHRFLKVDEAHKKHG---------KLVRIA   76
CYP53A12__8190__Nfi             25  VPYLQ---KWHLHDI--PSPSFAA--FSNLWL--LLQARRGRRFLKVDEAHKKYG---------KLVRIA   76
CYP53A12__3003__Afu             25  IPYLQ---KWHLHDI--PSPRFAA--FSNLWL--LLQARRGRRFLKVDEAHKKYG---------KLVRIA   76
CYP53A22__2015__Ate             24  VPYLK---RWHLRSI--PTPSVAG--FTNLWL--LIQARRGNRFEVVDNLHKKHG---------KLVRLA   75
CYP53A13__5958__Aor             25  LPYLQ---LWRLRDI--PSPGFAA--FSNLWL--MLQYRKGNRFVTVDNAHKKYG---------KLVRIA   76
CYP53A13__26719__Afl            25  LPYLQ---LWRLRDI--PSPGFAA--FSNLWL--MLQYRKGNRFVTVDNAHKKYG---------KLVRIA   76
CYP53A1__50153__Anig            24  LPYLK---RAHLRDI--PAPGLAA--FTNFWL--LLQTRRGHRFVVVDNAHKKYG---------KLVRIA   75
CYP53A3__3425__Anid             30  VPYLQ---TWRLSDI--PAPGLAA--WTNFWL--LLQTRLGHRFISVDNAHKKYG---------KLVRIA   81
CYP53A__2301715__Mth            26  YPYLV--TYRHLRHI--PAPFPAQ--FTNWWL--LLVCRRGNRYETVDKLHKKLG---------PVVRIQ   78
CYP53A4__88466__Ndi             26  VPYFV--TYSALRKI--PSPFPAQ--FTDLWL--LSVCRRGNRYQRVDELHKKLG---------PVVRIQ   78
CYP53A4__7508__Ncr              26  VPYFV--TYSALRKI--PSPFPAQ--FTDLWL--LSVCRRGNRYQRVDELHKKLG---------PVVRIQ   78
CYP53A19__9543__Fve             26  YSYFV--TYGHLRDI--PAPFPAQ--FTNLWL--LYVCRRGERYRVVDGIHKRLG---------PVVRIQ   78
CYP53A19__14206__Fox            26  YSYFV--TYGHLRDI--PAPFPAQ--FTNLWL--LYVCRRGGRYRVVDEIHKRLG---------PVVRIQ   78
CYP53A8__12085__Fgr             26  YSYFI--TYGHLRDI--PAPFPAQ--FSNLWL--LYVCRRGERYRVVDQIHKELG---------PVVRIQ   78
CYP53A11__50496__Nhe            26  YAYFV--TYGYLRGI--PAPFPAQ--FSNLWL--LYVCRRGERYDVMDKIHKKMG---------PVVRIQ   78
CYP53A20__6367__Fve             26  LPWI---SNSNLRGI--PAPFLAQ--FSNLWL--LSTCRRGKRYEIVDQVHKKLG---------VLVRIA   77
CYP53A20__10443__Fox            26  LPWI---SNSNLRGI--PAPFPAQ--FTNLWL--LSTCRRGKRYEIVDQVHKKLG---------VLVRIA   77
CYP53A7__10227__Fgr             26  LPWV---SNKDLRGI--PAPFPAQ--FSNLWL--LSTCRRGKRYEIVDQVHKKLG---------PLVRIA   77
CYP53A10__35086__Nhe            26  VPYFG--ANRGLRGI--PAPFPAQ--FSNLWL--MSTCRRGKRFEVVDQVHKRLG---------TVVRIA   78
CYP53A5__120117__Mgrs           27  VPYFT--TFGHLRSI-QPASPLAG--FSNLWL--LYTSRVGKRSLLVDEAHARLG---------PVLRVQ   80
CYP53A14__2107__Aor             38  TGYL---KRWHLRDI--PGPFIAG--FSRIWL--IVQVRQGYRSLVVHDLHRRYG---------KIVRLA   89
CYP53C__1025718__Fpi_           30  VPLIT--DPYQVRSY--PGPFLAK--ISDAWL--GWVAAQGHRSEVVHELHQKHG---------KFVQIA   82
Cyp53C__68781__Pst              23  GAYLL--DPHNIRDI--PGPTLAK--FSDAWL--GWVAAKGHRSEVVHEMHAQYG---------PVVRIA   75
CYP53C__80617__Gtr              27  VPYFT--DPHAIKSY--PGPWLAK--FSDAWL--GKVSAQGHRSEVVHDLHKKYG---------TFVRLA   79
CYP53C__127772__Cpu             49  VPWLW--DPYHQRSI--PGPFLAK--FSNAWL--GWVSAHGHRSEIVHELHKKYG---------PVVRIA  101
CYP53C__94174__Shi              31  IPWLV--DVHGIRSY--PGPFWAR--FTDLWL--GWVAAQGHRSEVVHEMHNKYG---------PIVRIA   83
CYP53c__27029__Wco              29  VPYLL--DPHGIRSY--PGPFLAK--LSDAWL--GWVAAKGHRSEVVHQLHQRYG---------TFVRIA   81
CYP53C3__110015__Ppl            29  VPYLL--DPHGIRAY--PGPFLAR--LSDIWL--GWIAAQGHRSETVHELHKQYG---------TFVRIA   81
CYP53C__116910__Csu             27  VPWLL--DPHGIRSY--PGPLLAK--FSDAWL--GWVAAQGHRSEVVHELHKKYG---------TFVRLA   79
CYP53C__1179842__Sla            34  FPYLA--DPFKQRAI--PGPLLAK--FSDAWL--GWVSSQGHRSEVVHKMHLKYG---------TFVRIA   86
CYP53C__55106__Dsq              29  VPYLA--DPHHIREY--PGPLLAK--LSDIWL--GYVAAQGHRSERVHELHKQYG---------TFVRIA   81
CYP53C4__GL08839-P1.1__Gl       29  VPYLV--DSHHIRGY--PGPLLAK--FSDVWL--GYVAAQGHRSEQVHELHKQYG---------TFVRIA   81
CYP53C4__47512__Gsp             29  VPYLV--DSHHIRGY--PGPLLAK--FSDVWL--GYVAAQGHRSEKVHELHEQYG---------TFVRIA   81
CYP53C__129211__Tve             29  VPYLL--DPHGFRSY--PGPFLAK--LSDFWL--GKVAADGHRSERVHELHEIYGN-------WTFVRIA   83
```

```
CYP53C9__27837__Pbr         30  VFYLV--DPYKIRSY--PGPLLAR--FSDLWL--GRVAAEGHRSEIVHKLHQKYG---------TFVRLA   82
CYP53C7__118978__Bad        33  IYFVI--DPHRIRSY--PGPLLAK--LTDAWL--GYVAAHGHRSEVVHGLHQKYG---------KFVRIA   85
CYP53C__183109__Pca         29  VHWLL--DPHGIRSY--PGPLLAR--FSDAWL--GYVAAQGHRSEVVHDLHKKHG---------TFVRLA   81
CYP53C2__130996__Pch        29  LIWLL--DPHGIRSY--PGPLLAK--FSDAWL--GYVAAQGHRSEVVHDLHKQYG---------TFVRIA   81
CYP53C__128292__Fme         35  VPYFV--DPHCIRNNGITGPLSAR--FSDAWL--GWVAAQGHRSEVVHEMHKKYG---------TFVRLA   89
Cyp53C__37267__Tve          29  VPYVL--DPHGIRAY--PGPFWAK--LTDLWL--GKIAADGHRSERVHDLHKKYG---------PFVRIA   81
CYP53C__70450__Ade          25  VPYFK--DEHGVRDI--PGPLAAH--LSNLWL--AYWSSQGKRSEMVHEQHLKCG---------KLVRIA   77
CYP53C__52716__Dsp          34  VPWVA--DPFGYRKKHIPGPFLAQ--LSDVWL--ARVAAQGHRSEIIHGLHQKYG---------KVVRIA   88
CYP53C__83844__Cpu          33  GPYAW--DRYHLKSI--PGPFWAK--FSDAWL--AWVAANGHRSEEVHKLHEKLG---------PVVRIA   85
CYP53C__194303__Abi         32  IPWLT--DSHSLRKY--PGPFFAK--FSDFWL--AFTSRGGRRSEIIHDYHKKFG---------PVVRIA   84
CYP53C__194181__Abi         36  LPWLW--DPHGLRAY--PGPFIAK--FSDIWL--TCVSKGAHRSELVHEAHLKYG---------PVVRIA   88
CYP53C__143663__Fme         32  YLVDR--YHIRRNGI--TGPSLAR--FSDAWL--GWVVANGRQSEVVHEMHKKFG---------PVVRLA   84
CYP53C__130308__Fme         33  YLLDK--HHIRRNGI--TGPFLAM--FSDAWL--GWVAAQGRRAEVVHEKHKKYG---------KFVRLA   85
CYP53C__24265__Fme          33  YLFDK--HHIRRNDI--SGPLLAR--FSDAWL--GWVAAQGPRSEVVHQIHKKYG---------KFVRLA   85
CYP53C__149618__Fme         34  YFIDS--HRIRRNGI--SGPFLAR--FSGAWL--GWVVFQGRQSEVVHSLHKKFG---------TFVRLS   86
CYP53C__94457__Fme          32  YFVDS--KHIRRNDI--PGPTLAK--VSGSWL--GRVALEGRQSEVVHELHKKFG---------TFVRLS   84
CYP53C__154594__Fme         27  YFIDP--YWIRRNEV--RGPFLAS--LTSLWF--GWNATRGHLSQVVHDLHKKFG---------TFVRLS   79
CYP53C__115179__Fme         31  QPYLVDSHFIRRNGI--TGPFFAR--FSDAWL--GWVAAHGNRSVVVHKLHKKYG---------LFVRLA   85
CYP53C1__5__Uma             37  VVPYI--TNTACIKY--PGPFFAK--FTDFWL--LRTALIGHRFEEVHKQHQKYG---------KFVRIA   89
CYP53B2__32280__Sro         34  VPHFT--SLAPLRRF--PGPFWAG--YTRLWL--ARTARVGKRSELVHREHLKHG---------KFVRIG   86
CYP53B3__28617__Pgr         28  TGYLR--NKHQLNRY--PGPFLAK--FSRLWL--GYATRFGNRYQIIHQLHQKHG---------RFVRIA   80
CYP53C__48859__Fpi          32  VHYLV--DSSDLRSY--PGPFLAK--FTDAWI--FWTVSRNRWSRSVEDAHIKYG---------PIVRIA   84
CYP53C__86809__Fpi          32  VHYLA--DSSDLRSY--PGPFLAK--FTDAWI--FWTVSSNRWSHSVEDAHIKYG---------PIVRIA   84
CYP53C__138909__Wco         35  VPYLL--DPLGLRAF--PGPILAK--FTIGWL--PWIVSQNRWSLTVNRLHQKYG---------IFVRLS   87
CYP53C__104840__Wco         37  VPYLL--DSLGLRAF--PGPTLAK--FASGWL--PWVISQNRWSVTVGRLHEKYG---------TFVRLA   89
CYP53C__77097__Wco          32  VPYLL--DPLGLSSF--PGPVLAK--FSNVWL--PWIVSQNRWSVTVDQLHRKYGKTLIYCGAGTFVRLA   93
CYP53C__138864__Wco         35  VPYFL--DPLGLRAF--PGPILAK--FTIGWL--PWVVSQNRWSLTVNRLHQKYG---------IFVRLS   87
CYP53C__104855__Wco         35  VSYLL--DPLGLRAF--PGPIFAR--FTSGWL--PWIISQNRWSVTVDRLHQKYG---------TFVRLS   87
CYP53C__154264__Wco         35  VPYLL--DPLGLRTF--PGPIFAK--FTSGWL--PWVISQNRWSAIVDSLHRKDG---------IFVRLS   87
CYP53c__138853__Wco         35  VPYLL--DPLGLRTF--PGPIFAK--FTSGWL--PWVVSQNRWSVAVDSLHRKYG---------IFVRLS   87
CYP53C__154237__Wco          2  -----------------------------------------------------YG---------IFVCLS    9
CYP53D1__108845__Ppl        46  LPYFN--DPYKLRAY--PGPFFAK--FTSAWL--SWIIGHNRWSETVYHLHRQHG---------PIVRLG   98
CYP53D6__54877__Ppl         46  IPYFN--DPYKLRAY--PGPFLAK--FTSAWI--SWTISHNPISEIVDHLHRQYG---------PIVRLG   98
CYP53D4__55859__Ppl         19  IPYFS--DPYKLRAY--PGPFLAK--FTSVWA--SWVINHNRWSETVDLLHRKYG---------PIVRLG   71
CYP53D2v1__56013__Ppl       46  LAYFN--DPFELRAY--PGPFLAR--FTSAWI--SWIISQNRWSETVDLMHRQHG---------PIVRLS   98
CYP53D2v2__48082__Ppl       46  LAYFN--DPFELRAY--PGPFLAK--FTSAWI--SWIISQNRWSETVDLMHRQHG---------PIVRLS   98
CYP53D5__46728__Ppl         31  LPYFI--DQLQLREY--PGALLAK--FTSGWI--SWIISRNQWSETVDRLHVQHG---------SFVRLA   83
CYP53D3__60352__Ppl         31  LPYFI--DQLQLREY--PGALLAK--FTSGWI--SWIISRNQWSETVDRLHSAHSM-------GSFVRLA   85
CYP53C__112429__Csu         30  VAHLR--DPHHLRSY--PGPFLAS--LTDLWL--AYKVWVGDRSPGVHELHKKHG---------TFLRIG   82
CYP53C__151209__Csu         31  APYIL--DRHRLRSY--PGPFLAR--FSDLWL--ASQVWKSHRSEEVHRLHKKYGK-------CRFLRIG   85
CYP53C__118598__Csu         46  VTYLL--DFYGLRSY--PGPFLAK--FTDLWL--AYKVWEGNRSPDIHLLHKKHG---------PFMRIG   98
CYP53H3__318972__Bad        26  VPFLR--DEHDVRSH--PGPALAK--LSDAWL--AWCAACGKINRSIYEAHKVYG---------PIVRIA   78
CYP53H7__358536__Bad        30  VSYLK--DTLHLRRF--PGPPLAR--LSDVWL--AWHCGRGTINRAVLAAHRTYG---------PVVRIA   82
CYP53H6__142452__Bad        29  LPYLA--DKLHLRRY--PGPPLAR--FSDVWL--AWHCARGSINRAVLEAHRTYG---------PVVRIS   81
CYP53H4__160054__Bad        29  VPFAL--DKFGVRQY--PGPLLAK--LSDIWL--GWHAAHGKINQAVWNAHRAYG---------PIVRIA   81
CYP53H2__55123__Bad         29  VPYAL--DRLSLSSH--PGPFLAS--LSDLWL--GWYAARGKMVRAVWDAHRVYG---------PVVRIA   81
CYP53H5__65034__Bad         25  LFSGF--HRSYLSSF--PGPLLAR--LSDAWI--GWHTARGTVNRAVYEAHKAYG---------PFVRIA   77
CYP53C__101826__Pca          2  IPFLV--DKYGLRGY--PGPLVAK--FSSLWL--ASKAHKGKTTSAVHALHQKYG---------PFVRIS   54
CYP53C__256510__Pca         30  VPFLV--DKHGLMAF--PGPLLAK--FSSLWF--ALKAYKGTTSLTVHALHERYG---------PFVRLS   82
CYP53C__102576__Pca         30  IPFLV--DKYRLRGY--PGPLLAK--FSGFWL--ASKAYKGTTTSAVYALHQKYE--------GPFVRIS   83
CYP53C__212559__Pca         30  VPFLI--DRYRLKGI--PGPLLAK--FSCVWL--ASKAYKGTMSSAVHVLHEKYG---------PFVRIS   82
```

```
CYP53D1__9015__Fox          26  LPYFY--TYRHLRGI--PGPLLAR--FSDLWL--LYICRQSKRSYTVYDLHERLG---------PVVRIQ   78
CYP53A6__10234__Fgr         26  IPYFW--NYRHLRSI--PGPLFAR--LSNWWL--VYACREKSRWKYVNDAHTRYG---------PVVRIQ   78
Cyp53A__101387__Pst         34  YIVAV--SRDPLQKV--PGPLLAR--WSNLWQ--AFYTRFGIRYKAIHAVHKTYG---------PVVRIS   86
CYP53A__105834__Cpu          1  ------MKPDPLADV--PGPLLAR--WTPLWL--GYYARIGQRFTAVHKLHMEYG---------PIVRIA   49
CYP53A__56813__Ade          16  -LWRW--RADPLRNV--PGPWLAR--WTPFWL--MYHARRGERYLAVHDAHKKYG---------PAVRIA   67
CYP53__1116154__Fpi         28  VSYLR-RRSDPIHAIPTVGPSWPLLSYLGAWR--YFRDAK----GMILEGCSKY----------EVFKIP   80
CYP53NS__152212__Pca        34  VLFVY-AYIKARRQF--PGPPV-----TNIWKGNLDETMTEDVHDKWRRWHRQYG---------PIYQTV   86
CYP53NS__92916__Fme         33  VLFFI-AYFKARGQF--PGPPV-----SSLWSGNLSESMADDVHDKWRTWHRKYG---------PVFQTW   85
Consensus_aa:                   h.@h...p...lp.h..PGP.hAp..@oshWh.....h.psp....l.phHpp@G.........hVRls
Consensus_ss:                   hhhh                      hh  hh  hhhh    hhhhhhhhhhhh           eeee

Conservation:                         7  576      5   665       6     8  5          7   56  65 676  7
CYP53A__2107910__Tte        79  ------PNHVSILDD--EAIQAIYGH-GNGFLK--SDFYDAFVSI---QRGLFNTRDRAEHTRKRKIVSH  134
CYP53A15__ACF15219.1_Clu    75  ------PNHVSIADA--DAITQVYGH-GNGFLK--SEYYDAFVSI---RRGLFNTRDRAEHTRKRKTVAH  130
CYP53A23__71345__Mfi        74  ------PNHVSIAEP--EAIPIIYGH-GTGFLK--SEYYDAFVSI---QRGLFNTRDRAEHTRKRKTVSH  129
CYP53A17__5031__Ure         75  ------PNHVSVADA--DAIQTIYGH-GNGFLK--SEYYDAFVSI---RRGLFNTRNRAEHTRKRKTVSH  130
CYP53A18__8044__Cim         75  ------PDHVSVADA--DAIQTIYGH-GNGFLK--SEYYDAFVSI---RRGLFNTRSRAEHTRKRKTVSH  130
CYP53A21__1341__Acl         77  ------PGHISIADD--GAIQAVYGH-GNGFLK--ADFYDAFVSI---RRGLFNTRDRAEHTRKRKTVSH  132
CYP53A12__8190__Nfi         77  ------PRQVSIADD--AAIQAIYGH-GNGFLK--SDFYDAFVSI---RRGLFNTRDREEHTRKRKTVSH  132
CYP53A12__3003__Afu         77  ------PKHVSIADD--AAIQAIYGH-GNGFLK--ADFYDAFVSI---RRGLFNTRDRAEHTRKRKTVSH  132
CYP53A22__2015__Ate         76  ------PRHVSIADD--AAINAIYGH-GNGFLKALSDFYDAFVSI---RRGLFNTRDRAEHTRKRKTVSH  133
CYP53A13__5958__Aor         77  ------PRHVSIADD--EAIQAIYGH-GNGFLK--ADFYDAFVSI---RRGLFNTRDRAEHTRKRKTVSH  132
CYP53A13__26719__Afl        77  ------PRHVSIADD--EAIQAIYGH-GNGFLK--ADFYDAFVSI---RRGLFNTRDRAEHTRKRKTVSH  132
CYP53A1__50153__Anig        76  ------PRHTSIADD--GAIQAVYGH-GNGFLK--SDFYDAFVSI---HRGLFNTRDRAEHTRKRKTVSH  131
CYP53A3__3425__Anid         82  ------PRHISIADD--AAIQAVYGH-GNGFLK--SDFYDAFVSI---RRGLFNTRDRAEHTRKRKTVSH  137
CYP53A__2301715__Mth        79  ------PNHVSLCDD--AAIQVVYGH-GNGFLK--SDFYDAFVSI---QRGLFNTRDRAEHTRKRKIVSH  134
CYP53A4__88466__Ndi         79  ------PNHVSICDD--AAIPTIYGH-GNGFLK--SDFYDAFVSI---RRGLFNTRDRAEHTRKRKIVSH  134
CYP53A4__7508__Ncr          79  ------PNHVSICDD--AAIPTIYGH-GNGFL---NDFYDAFVSI---RRGLFNTRDRAEHTRKRKIVSH  133
CYP53A19__9543__Fve         79  ------PNHTSIADP--DAIATIYGH-GNGFLK--SDFYDAFVSI---RRGLFNTRDRAEHTRKRKLISH  134
CYP53A19__14206__Fox        79  ------PNHTSIADP--DAIATIYGH-GNGFLK--SDFYDAFVSI---RRGLFNTRDRAEHTRKRKLISH  134
CYP53A8__12085__Fgr         79  ------PNHTSIADA--EAIATIYGH-GNGFLK--SEFYDAFVSI---RRGLFNTRDRAEHTRKRKLISH  134
CYP53A11__50496__Nhe        79  ------PNHVSIADD--EAIPIIYGH-GNGFLK--SEFYDAFVSI---RRGLFNTRDRAEHTRKRKLISH  134
CYP53A20__6367__Fve         78  ------PNHVSVADA--DAINTIYGH-GNGFLK--ADFYDTFVSI---RRGLFNTRDRAEHSRKRKIVSH  133
CYP53A20__10443__Fox        78  ------PNHVSVADA--DAINTIYGH-GNGFLK--ADFYDTFVSI---RRGLFNTRNRAEHSRKRKLVSH  133
CYP53A7__10227__Fgr         78  ------PNHVSVADA--DAINTIYGH-GNGFLK--ADFYDTFVSI---RRGLFNTRDRAEHSRKRKIVSH  133
CYP53A10__35086__Nhe        79  ------PNHVSIADA--DAINVIYGH-GNGFLK--SDFYDPFVSI---RRGLFNTRSRAEHSRKRKIVSH  134
CYP53A5__120117__Mgrs       81  ------PNHVSIADD--EAINIIYGH-GNGFLK--SSFYDAFVSI---RRGLFNTRDRAEHTRKRKLISH  136
CYP53A14__2107__Aor         90  ------PNHISIADE--SAIQAIYGH-GNGFLE--TDFYNAFLNV---DWSIFTTRSRAEHTRKRKIVSH  145
CYP53C__1025718__Fpi_       83  ------PNHVSVSDP--DALQVIYAH-GNGTLK--STFYDAFVSI---QRGLFNTRSRPEHARKRKIVSH  138
Cyp53C__68781__Pst          76  ------PNHVSIAEP--QALQIVYAH-GNGSLK--SNFYDAFVSI---QRGLFNTRNRADHARKRKIVSA  131
CYP53C__80617__Gtr          80  ------PNHLSIADP--EALQTVYAH-GNGSLK--SDFYDAFVSI---RRGLFNTRDRAEHARKRKIVSH  135
CYP53C__127772__Cpu        102  ------PNHVSVADP--EALQVVYAH-GNGSLK--SDFYDAFVSI---HRGLFNTRDRQQHARKRKIVSG  157
CYP53C__94174__Shi          84  ------PNHISISDP--EALQIVYAH-GNGSLK--SNFYDAFVSI---HRGLFNTRDRAAHARKRKIVSH  139
CYP53c__27029__Wco          82  ------PNHVSISDP--DALSEVYAH-GNGTMK--SNFYDAFVSI---QRGLFNTRSRPEHARKRKIVSH  137
CYP53C3__110015__Ppl        82  ------PNHVSISDP--EAIQYVYAH-GNGTTK--SNFYDAFVSI---RRGLFNTRSRPEHARKRKIVSH  137
CYP53C__116910__Csu         80  ------PNHVSISDP--DAIQIVYAH-GNGSLK--SNFYDAFVSI---QRGLFNTRSRPEHARKRKIVSH  135
CYP53C__1179842__Sla        87  ------PNHVSVADP--DALQVVYAH-GNGSLK--ANFYDAFVSI---QRGLFNTRNRNEHARKRKIVSH  142
CYP53C__55106__Dsq          82  ------PNHLSISDP--EALQVVYGH-GTGTLK--SDFYDAFVSI---QRGLFNTRSRVQHARKRKIVSN  137
CYP53C4__GL08839-P1.1__Gl   82  ------PNHLSISDP--DALQVVYGH-GTGTLK--STFYDAFVSI---QRGLFNTRSRVQHARKRKIVSN  137
CYP53C4__47512__Gsp         82  ------PNHLSISDP--DALQIVYGH-GTGTLK--STFYDAFVSI---QRGLFNTRSRVQHARKRKIVSN  137
```

CYP53C__129211__Tve	84	------PNHLSIADP--DALQIVYGH-GTGTLK--SDFYDAFVSI---QRGLFNTRSRTDHARKRKIVSH	139
CYP53C9__27837__Pbr	83	------PNHVSVSDP--DALQVVYAH-GNGTLK--ANFYDAFVSI---QRGLFNTRSRPEHARKRKIVSH	138
CYP53C7__118978__Bad	86	------PNHVSIADP--DALPIVYGH-GNGTLK--SNFYDAFVSI---ERGLFNTRSRHEHARKRKIVSH	141
CYP53C__183109__Pca	82	------PNHVSISDP--DALQVVYGH-GTGTLK--SDFYDAFVSI---QRGLFNTRSRPEHARKRKIVSH	137
CYP53C2__130996__Pch	82	------PNHLSIADP--DALQVVYGH-GTGTLK--SNFYDAFVSI---QRGLFNTRSRSEHARKRKIVSH	137
CYP53C__128292__Fme	90	------PNHVSISDP--AALQIVYAH-GNGTLK--SSFYDAFVSI---RRGLFNTRSRPEHTRKRKIVSH	145
Cyp53C__37267__Tve	82	------PNHLSISDP--DALPVVYGH-GTGTLK--SDFYDAFVSV---QRGLFSTRSRPEHTRKRKIVAH	137
CYP53C__70450__Ade	78	------PNHISVNDP--DALPIVYGH-GTGTLK--SEFYDAFVSI---QRGLFNTRSRTQHTRKRKIVSH	133
CYP53C__52716__Dsp	89	------PNHISLSEP--GALQIVYAH-GNGALK--SDFYEAFVSI---RKNIFSTRDRAEHTRKRKIVSH	144
CYP53C__83844__Cpu	86	------PNHISIADP--DALQIIYAH-GSNTLK-KSNFYDAFVSI---RRAIFNTREKADHARKRKIVAN	142
CYP53C__194303__Abi	85	------PNHVSISDP--DALNAVYGH-GTGTLK--SEFYDAFVAM---DRGLFNVRDRHDHTRKRKVISH	140
CYP53C__194181__Abi	89	------PNHLSIANP--EALQIVYAH-GNGALK--SIFYDAFVSI---RRGLFNVRDRNEHTRKRKIVSH	144
CYP53C__143663__Fme	85	------PNHVSISDP--GALHVIYGH-GSGLLK--SGYYEPFTAV---RPSIFSTRSREVHSKKRKIISH	140
CYP53C__130308__Fme	86	------PNHVSIADP--EAIGDIYGH-GNGTLK--TDFYDAFISI---GVTVFTTRSREEHTRKRKVISH	141
CYP53C__24265__Fme	86	------PNHVSIAYP--EAIGEIYGH-GNGTLK--TDFYDAFLSI---DRTIFTTRSREEHTRKRKVIAH	141
CYP53C__149618__Fme	87	------PNHVSISDP--DALRLVYGR-GNGALK--SDYYDAFLAV---RPSIFTTRSKEEHARKRTAIAH	142
CYP53C__94457__Fme	85	------PNHVSISDP--EALQVVYGH-GNGMLK--SEYYDAFAAP-NLRRSVFDTRSREEHARKRKAISH	142
CYP53C__154594__Fme	80	------PNHVSISDP--DALQTIYGH-GKGLMK--SDYYDAFKGL---RPSIFSTRDRAFHAWKRKAISH	135
CYP53C__115179__Fme	86	------PNHVSISDP--EALHIVYGH-GSGTLK--SDYYDAFLAI---RHTVLTTRDREDHSMKRKLVAP	141
CYP53C1__5__Uma_	90	------PNHVSIADH--EALQPIYGH-GTGTLK--PAYYDAFVPPRPFPRGLFNTRDRAEHTRKRKIVSH	148
CYP53B2__32280__Sro	87	------PNEVSIADP--AALPIVYAH-GSGSIK--ADFYDAFVAS--PVRGLFNTRNRAEHTRKRKIVSH	143
CYP53B3__28617__Pgr	81	------PNELSIADP--DAVHIVLGH-GTGTTK--SKFYDAFVAI---HRGLFNTRDRADHTRKRKIISS	136
CYP53C__48859__Fpi	85	------PNHISIDDP--KALAMVYGH-STGFMK--SNWYDIFAAF--SVSNIFDTRSRSEHARKRRMEAH	141
CYP53C__86809__Fpi	85	------PNHISIADP--KALATVYGH-STGFTK--ANWYNAFSEF--AAKNIFNTRSRSEHARKRRMEAH	141
CYP53C__138909__Wco	88	------PNYVSIAHP--AALSAVYGH-SSGATK--APYYEVFGDF--RARNLFNILSRPEHARRRRLEAH	144
CYP53C__104840__Wco	90	------PNHVSIAHP--AALSAVYGH-SSGALK--APFYDASGNF--KARNMFNTRSRSEHARKRRSESH	146
CYP53C__77097__Wco	94	------PNHVSIAHP--AALPAVYGH-SSGTLK--APLYDVFGPF--RARSIFSTRSRTEHARKRRIESP	150
CYP53C__138864__Wco	88	------PNHVSIAHP--AALSAVYGY-SSGVTK--APYYDVFGDF--RAKNLFNIISRTEHTRKRRIVSP	144
CYP53C__104855__Wco	88	------PNHVSIAHP--AALPAVYGH-GSGAPK--APYYDGFVNF--KSRNMFNTLSRSEHARKRRIESH	144
CYP53C__154264__Wco	88	------PNHISIAHP--VALPAVYGH-SSGALK--APFYDAFASF--KTRNMFNTLSRTEHTRKRRIESQ	144
CYP53c__138853__Wco	88	------PNYVSIAHP--VALPAVYGH-SSGALK--APFYDALSGF--KTRNMFNTLSRTEHARKRRIESH	144
CYP53C__154237__Wco	10	------PNHISIVHP--VALPAVYGH-SSGALK--APFYDAFASF--KTRNMFNTISRTGHTRKRRIESQ	66
CYP53D1__108845__Ppl	99	------PDNVSISDP--SALAAIYGH-SSGALK--STFYDAISSI--RIRNLFNTRDRAEHSRKRRIEAH	155
CYP53D6__54877__Ppl	99	------PNNVSIADPSGSAFSVIYSH-SSGVTK--SAFYDTFANF--RIRNIFNTRDRAEHSRKRRVEAH	157
CYP53D4__55859__Ppl	72	------PDSVSIADP--SAFAVIYGH-SSGALK--APFYDAFANH--RIRDLFNTRDRAEHSRKRRVEAH	128
CYP53D2v1__56013__Ppl	99	------PDHVSVASP--AAFAAVYGH-SSGALK--APFYNAFANF--KIRSIFNTRDRAEHSRKRRVEAH	155
CYP53D2v2__48082__Ppl	99	------PDHVSVASP--AAFAAVYGH-SSGALK--APFYNAFANF--KTRSIFNTRDRAEHSRKRRVEAH	155
CYP53D5__46728__Ppl	84	------PNHVSVSGP--SAFEAIYGH-PSSAAK--APFYDIFSAG--GAANIFTTRDRAEHARKRRVEAH	140
CYP53D3__60352__Ppl	86	------PNHVSVSGP--SAFEAIYSH-PSSALK--APFYDIFSAG--GAANIFTTRDRAEHARKRRVEAH	142
CYP53C__112429__Csu	83	------PNHISIASP--AALGVIYSH-SHPLLK--SDFYDGLATF--SAPGTFTVRDRVAHARKRRVVAH	139
CYP53C__151209__Csu	86	------PNHVSVADP--AAIPILYSH-SNPLMK--SDFYDGFTTF--RTPGIFVERDRVAHARKRRVVSH	142
CYP53C__118598__Csu	99	------PNHISVVSP--AAISTIYSH-IDPLPK--SAFYDGLATF--SVPDIFTTRDRVTHGRKQRMVSH	155
CYP53H3__318972__Bad	79	------PNHISIADV--SALQPIYGH-GSGILK--AESYDTFVAF--DTPSLFTTRSRDEHARKRKVVSN	135
CYP53H7__358536__Bad	83	------PNHVSIADV--SALHLVYGH-GSGALK--ADFYDAFVGR--ATSSVFTTRSRHDHTRKRKALAH	139
CYP53H6__142452__Bad	82	------PTQISVADV--SALQPIYGH-GSGAPK--AESYDAFSGL--GRPSIFTTRSREEHTRKRKSLAH	138
CYP53H4__160054__Bad	82	------PNHVSVADA--SALHQIYGH-STGILK--ADLYDAFVSF--NRASIFSTRSREEHARKRKILAH	138
CYP53H2__55123__Bad	82	------PNHVSIADV--AALRTVYGH-SAGILK--ADLYDVFNPF--GRTTLFTTRSREEHARKRKIVAH	138
CYP53H5__65034__Bad	78	------PNHISISHS--SALQPIYGH-SVGILK--SEFYDIFTSF-NGTKSVFTTRSREEHARKRKILSH	135
CYP53C__101826__Pca	55	------SKHVSIADP--EALQAIYGH-NSGALK--TDFYDAFVAF---RHNIFTSRSRLEHSRKRKYTAY	110
CYP53C__256510__Pca	83	------PQHVSIADP--EALRAIYGH-SSGTLK--TELYDAFVTF--------------FLARKRKYTAH	127
CYP53C__102576__Pca	84	------PKYVSIADP--EALQAIYGH-TSGTLK--TDFYDAFVTF---RRNIFTSRSRLEHSRKRKYTSH	139

```
CYP53C__212559__Pca          83   ------PKYVSIADP--EALQAIYGY-SSGALK--TELYDAFVFF---RPTMFSTRSRLEHSRKRKYTAH  138
CYP53D1__9015__Fox           79   ------PNHVSIVDE--RAINLVYGH-GNGLEK--SSWYDSSISL---TRSIFTARKRAEHARKRRYIAH  134
CYP53A6__10234__Fgr          79   ------PNHVSIANE--EVINAIYGH-GNGMLK--SSFYDASVIT---TYSIFTSRDRAEHSRKRKVVSH  134
Cyp53A__101387__Pst          87   ------PNHVSIADM--SLLPSIYGQGMAAFNK--SPFYDAFLSE---KPSIFSTRDKQEHAQKRRNYSG  143
CYP53A__105834__Cpu          50   ------PNHISVADK--DALDLVYAQGSNAFDK--STFYHAFVSD---KASVFSTTDRHDHAQKRRLVSN  106
CYP53A__56813__Ade           68   ------PWHVSFASP--DAPARVYAQGSAALDK--SPFYRAFYVQ--GAESLFSTQNRALHAAKRRLLSQ  125
CYP53__1116154__Fpi          81   L---SDQWLVVVSGR--DMNDELRKY-PDDTMS--ALEAQKWVV-----QTEYTLGNNNPDATAKKCISG  137
CYP53NS__152212__Pca         87   RWNGLFSRVIYVGDP--RLIRKIANE--NWPKF--PAQYAGFRPL--SGSALFAQMDQARWKTQRRGLAP  148
CYP53NS__92916__Fme          86   NG--LFSRVVYVGDP--RIISKIGNS--NWPKF--HAQYSGFKPL--SGSALFAQMDQERWKQQRKGLAP  145
Consensus_aa:                     ......PphlSltcs...Al..lYtc.tsthhK..t.hYcsFhsh.....slFsh.sR.pHspKR+hht.
Consensus_ss:                             eeee  h  hhhhhhhh              hhhh        eee    hhhhhhhhhhhh


Conservation:                      76 55    6         5   5                                   6   6   5
CYP53A__2107910__Tte        135   TFSVKSVAQFEPYIHSNLELFVRQLDDLIARSTSP-----------------DG--AAHLDCLHW-FNYL  184
CYP53A15__ACF15219.1_Clu    131   TFSAKSVLQFEQYIHHNLQELQNQWDRRAESV--------------------KGG-WYEMDALNW-FNYL  178
CYP53A23__71345__Mfi        130   TFSAKSVGQFEQYIHHNLELLAKRWDEIAKNT--------------------GAGKYTRFDALHW-FNYV  178
CYP53A17__5031__Ure         131   TFSAKSIGQFEQYIHANLQLFLQQWTQICDLQRNP-----------------RSG-YASIDALNW-FNYL  181
CYP53A18__8044__Cim         131   TFSAKSVGQFEQYIHANLQLFFQQWTNISEVQRNP-----------------KSG-YASIDALNW-FNYL  181
CYP53A21__1341__Acl         133   TFSTKSIGQFEQYIHHNIELFVKQWTKLSKLNGNP-----------------RSG-YATIDALNW-FNFL  183
CYP53A12__8190__Nfi         133   TFSMKSIGQFEQYIHQNVELFVQQWTKLAKLNGNP-----------------RSG-YATIDALNW-FNYL  183
CYP53A12__3003__Afu         133   TFSMKSIGQFEQYIHQNVELFVQQWTKLAKLNGNP-----------------RSG-YATIDALNW-FNYL  183
CYP53A22__2015__Ate         134   TFSMKSIGQFEQYIHHNIELFVKQWTRLSETQGNP-----------------RSG-YATIDALNW-FNFL  184
CYP53A13__5958__Aor         133   TFSMKSIGQFEQYIHGNAELFVKQWNRIADTQSNP-----------------KTG-YATIDALNW-FNYL  183
CYP53A13__26719__Afl        133   TFSMKSIGQFEQYIHGNAELFVKQWNRIADTQSNP-----------------KTG-YATIDALNW-FNYL  183
CYP53A1__50153__Anig        132   TFSMKSIGQFEQYIHGNIELFVKQWNRMADTQRNP-----------------KTG-FASLDALNW-FNYL  182
CYP53A3__3425__Anid         138   TFSAKSIGQFEQYIHHNIENLVKQLTRISNLQRNP-----------------KNG-YATVDALNW-FNFV  188
CYP53A__2301715__Mth        135   TFSVKSVAQFEPYIHSNLELFVRQLDNLIARSTNPDG-------------------AAHLDCLHW-FNYL  184
CYP53A4__88466__Ndi         135   TFSAKSVQQFEPYMHSNLELFVKQWDSMIKNTKNPDK-------------------AAHLDCLEW-FNYL  184
CYP53A4__7508__Ncr          134   TFSAKSVQQFEPYMHSNLELFVKQWDSMIKNSKNPDK-------------------AAHLDCLEW-FNYL  183
CYP53A19__9543__Fve         135   VFSAKSISQFEPYIHANLELFVKQLDKLVASGQTT--------------DKNGKR-QALIDCLPW-FNYL  188
CYP53A19__14206__Fox        135   VFSAKSISQFEPYIHANLELFVKQLDKLVASGQTT--------------DKNGKR-QALIDCLPW-FNYL  188
CYP53A8__12085__Fgr         135   VFSAKSISQFEPYIHANLELFVKQLDKLVASGQMA---------------KNGKR-EALMDCLPW-FNYL  187
CYP53A11__50496__Nhe        135   TFSTKSISQFEPYIHSNLELFVKQLDKLITSGTTK--------------DNQGHQ-QALIDCLPW-FNYL  188
CYP53A20__6367__Fve         134   TFAPKSVLEFEPYIRQNLDIFINQWDRIAS-NKEADG-------------------YGSVDCLNW-FNFL  182
CYP53A20__10443__Fox        134   TFAPKSVLEFEPYIRQNLDIFINQWDRIAS-NKDADG-------------------YGSVDCLNW-FNFL  182
CYP53A7__10227__Fgr         134   TFAPKSVLEFEPYIRQNLEIFVKQWDRISS-NKERDG-------------------YGRVDCLNW-FNFL  182
CYP53A10__35086__Nhe        135   TFAPKSVLEFEPYIHQNLDLFVKQWDRASS-NPEADG-------------------AGRLDCLSW-FNYL  183
CYP53A5__120117__Mgrs       137   TFAPKSVGQFEPYIHGNLELFAKKWDELIERTKKSDG-------------------WAPVECLQW-----  182
CYP53A14__2107__Aor         146   AFSARSLAQVEQHAHNNMEHLVRQWRKMIDSEEGL-----------------DDP-YAVIDARVW-CNYL  196
CYP53C__1025718__Fpi_       139   IFSQKSVLEFEPYTRMHIKKLMNQWDRLYDLAMKGGSGEEGE-G-----WQGRDG-RLWLDILPW-YNYL  200
Cyp53C__68781__Pst          132   IFSMKNVLEFEPHVREYVGLLIKQWDRLCAEAVKGGSGDEGEGG-----WRGESG-RLWLDCLPW-YNYL  194
CYP53C__80617__Gtr          136   IFSQKSVLEFEPHVRLYVRQFIEQWDRLCGLAAKGERGEEGNG------WEGREG-RLWLDCLPW-YNYL  197
CYP53C__127772__Cpu         158   IFSQKNVLEFEPHVRLYVGQLMEQWDRLCARAEKGESGDEGEGG-----WQGRGG-KLWLDCLPW-YNYL  220
CYP53C__94174__Shi          140   IFSQKNVLEFEPHVREYVKSLIAQWDRLYDLAVNGESGTEGEGG-----WVGREG-RLWLDCLPW-YNYL  202
CYP53c__27029__Wco          138   IFSQKSVLEFEPYTRQHVGALFKQWDRMCELGTKGLFGEEGEGG-----WHGRDG-RVWFDCLPW-YNYL  200
CYP53C3__110015__Ppl        138   IFSMKSVMEFEPYTRMHVAQLLKQWDRLYELGIKGASGEEGEG------WKGRDG-RVWLDCLPW-YNYL  199
CYP53C__116910__Csu         136   IFSQKNVLEFEPHVREHIRTLISQWDRLYELGKKGLSGTEGEGG-----WQGKNG-RVWLDCLPW-WNYL  198
CYP53C__1179842__Sla        143   IFSQKNVLEFEPNVRLYVGQLISQWDRLYDSAAKGASGTEGEGG-----WFGKDG-RLWLDSLPW-YNYL  205
CYP53C__55106__Dsq          138   IFAQKNVLDFEPHVRQHLANLFRQWDKLCEGGKNGLSGDEGEGG-----WQGRDG-RVWYDCLPW-YNYL  200
CYP53C4__GL08839-P1.1__Gl   138   IFAQKNVLEFEPHVREHLGTLFQQWDKLCDGGKKGLSGTEGEGG-----WHGGEG-RVWYDCLPW-YNYL  200
```

```
CYP53C4__47512__Gsp        138  IFAQKNVLEFEPHVRQHLGTLFQQWDKLCDGGKKGLSGTEGEGG-----WHGSDG-RVWYDCLPW-YNYL  200
CYP53C__129211__Tve        140  IFSQKNVLEFEPHVRVHLIQLFKQWDRLCAGGARGEAGDEGEGG-----WRGRDG-RVWYDCLPW-YNYL  202
CYP53C9__27837__Pbr        139  IFSQKSVLEFEPNTRLYVRQLIAQWDRLCELGAKGLSGDEGEGG-----WKGRNG-RVWLDCLPW-YNYL  201
CYP53C7__118978__Bad       142  IFSQKSVLEFEPNVRTYVGQLIGKWDRLYENGAKGLSGDEGEGG-----WTGRNG-RVWLDCLPW-YNYL  204
CYP53C__183109__Pca        138  IFSQKSVLEFEPHVRLYVNQLIRQWDRLYEAGAKGLSGDDGESG-----WTGRNG-RVWLDCLPW-YNYL  200
CYP53C2__130996__Pch       138  IFSQKSVLEFEPHVRLYVKQLIQQWDRLYEAGAKGL---------------------VWLDCLPW-YNYL  185
CYP53C__128292__Fme        146  IFSQKSVLEFEPHIRLHVGELFTQWDKLCDGGKRGLKGTEGDG------WEGHDG-WVWFDCLPW-FNYL  207
Cyp53C__37267__Tve         138  SFSQKSVHEFEPHVRENLSKLFKQWDTLCEGGAKGLSGNEGEGG-----WQGREG-RVWYDCLPW-YNYL  200
CYP53C__70450__Ade         134  VFSQKNVLGFEPNLHSALSRFVSQWDRMCAAGVKGGRGNE-QDG-----WHGDGQ-RVWMDCLPCAYNYL  196
CYP53C__52716__Dsp         145  IFSQKSVLEFEPYIRQALGKLVKQWDSLLSDDRKLASHRLR---------PNENG-TAWFDCLNW-YNYL  203
CYP53C__83844__Cpu         143  TFSQKNVIEFEPRVRIYVGQIIDQWDRLSKLAAADGSGDEGESG-----WYGKDE-RLWLDVLPW-MNYL  205
CYP53C__194303__Abi        141  IFAQKSVVAFEPKIAIYVTQLLNQWDRLYDMAVKGGSGNEGEGG-----WKGKDG-KLYLDILPW-MNYL  203
CYP53C__194181__Abi        145  IFSQKNVLEFEPHIRMYVAQLQNQWDRLYDMAVKGMSGNDGEGG-----WEGRDG-RLWLDCLPW-ANYL  207
CYP53C__143663__Fme        141  VFSQKSVLEFEPFVHLHLAELFEHWDKMCDGGKEGLSGTESEGG-----WKRRGG-QAWFDIMPW-FNYL  203
CYP53C__130308__Fme        142  GFSQKSVSEFEPYIRLHVSELFEQWDELYDGGRKGLTGVEGEGG-----WKGHDG-RVWFNAMPW-CNYL  204
CYP53C__24265__Fme         142  GFSQKSISQLEPYIRLHVAELFEKWDKLYDGGRKGLTGVEGHNS-----WEGHDG-RVWFNAMPW-LNYL  204
CYP53C__149618__Fme        143  AFSQKSVLEFEPYIRLHVAELFNQWDRMCNRGKNGLSGTEGEGG-----WIGQGG-RVWFDIMPW-FHYL  205
CYP53C__94457__Fme         143  IFSQKSVLEFEPYIHTHLTDFFKQWDKLCDGGRKGFSGIEGEGG-----WKGHDG-RVWFNAMPW-YNYL  205
CYP53C__154594__Fme        136  IFSPKSVIDFEPYIHLHLTELFEQWDKLYDGGKRGLSGVEGEG------WNGRQG-RVWFNIMPW-FNYL  197
CYP53C__115179__Fme        142  IFSQKSVLGFEPCVHSHVTELFEQWDKLCDGGKQGLTGNAGKGG-----WKGRDG-RVWFDALPW-LYYM  204
CYP53C1__5__Uma_           149  TFAPKTIVAFEPFIRREVQLLLERWDEFCDKATKDNTEGP----------RGIKG-RAWLDSLMW-LNYF  206
CYP53B2__32280__Sro        144  TFAPKSVREFEPYIASTVNLLLKKWDQLAAKAQKSPPSGTG--------GERMKG-YAVIDSLDW-FNAL  203
CYP53B3__28617__Pgr        137  TFSQKSILEFEPYIADTLACFLRKIDQVASEPNLVQLPSHSWDS-----KHLNER-WRIIDILPW-FNYL  199
CYP53C__48859__Fpi         142  MFAPQSIRAVEPISHSHVNELLRQWDGLISRVAKAQQGGPNGGHIGATTWNVQDG-RVWIDCMPW-LNFW  209
CYP53C__86809__Fpi         142  MFAPQSIRAVEPISHAHVNELLRQWDGLISRVAKAQQGGPNGGHIGATTWNVQDG-RVWIDCMPW-LNFW  209
CYP53C__138909__Wco        145  MFSLQSVRALEINARVHHNNLVNQWERLYSYVKQAEKGGIREDKLGASAWRVEDG-RVWFDCMPW-FSYW  212
CYP53C__104840__Wco        147  MFSPQSVRALEGTARVHHGNFVNQWGKLYSYVAQAKSGEAKEGKLGACAWKVEDG-RVWFNCMPW-FNYW  214
CYP53C__77097__Wco         151  MFSPQSVRALEGTTRVHHSDLASQWDNLYSYVKQAENGGSREGMLGESAWKVEDG-RVWFDCMPW-FNYW  218
CYP53C__138864__Wco        145  MFSQQSVRALEINARVHHNNLVSQWERLYSYVKQAEKGGIREDKLGASAWRVEDG-RVWFDCMPW-FSYW  212
CYP53C__104855__Wco        145  MFSPQSVRALEGTASVHHGNLVSQWDKLYSYVKQAEGGGAKEGQLGASVWKVEGG-RVWFDCMPC-----  208
CYP53C__154264__Wco        145  IFSQQSVRELEGTARVHHIDLVSQWDKLYSYVKRAESGAAREG-----------------------FNYW  191
CYP53c__138853__Wco        145  MFSPQSVRALEGIARMHHNDLVSQWDKLYSYVKRAESGAAREGSLGECAWKVKDG-RVWLDCMPW-FNYW  212
CYP53C__154237__Wco         67  IFSQQSVRELEGTTRVHHIDLVSQWDKLYSYVKRAESDGVREG-----------------------FNYW  113
CYP53D1__108845__Ppl       156  MFSPRGIRALEDTARVHFQVLVRQWDTLCAHTDKAIRGSAEGTIGT-VHWKVHSG-RVWFDCMPW-FTFW  222
CYP53D6__54877__Ppl        158  MFAPQSIRALEETARVHFQVLLRQWDAMCAHAEKAGRGRADGAIGA-VPWKVHGG-RVWFNCMIW-FSYW  224
CYP53D4__55859__Ppl        129  IFAPQSIRALEDTARVHFEILVRQWDAMCAHAEKAGRGSAEGAIGT-VPWKVHDG-RVFFNCMLW-FSFW  195
CYP53D2v1__56013__Ppl      156  MFSPRSIRALEDTARVHFQVLVRQWDALCAPTGKTVRGSAEGTLGT-ISWKVHGD-RVWFDCMPW-FNFW  222
CYP53D2v2__48082__Ppl      156  MFSPRSIRALEDTARVHFQVLVRQWDALCAPTGKTGRGSAEGTLGT-ISWKVHGD-RVWFDCMPW-FNFW  222
CYP53D5__46728__Ppl        141  MFSPQSIRTLESTVSVHFHALVDQWDALCAHIQKAGSGGAEGIIGS-VSWKVHES-RVWFDCMPW-FMFW  207
CYP53D3__60352__Ppl        143  MFSPQSIRTLESTVSVHFHALEDQWDALCAHIQKAGSGGAEGIIGS-VSWKVHDS-RVWFD---W-FMFW  206
CYP53C__112429__Csu        140  LFAPKTVRMFEGALHKYIGQLVQQWDGMYKNVETALPGTATAGKAGDMSWIVRDG-RVWFDCMPW-LNFL  207
CYP53C__151209__Csu        143  LFAPKTVKAFEPAVQNYVGQLVRQWDRLCKN-ADAQSVSVMSGVLGSMTWRAYDG-CVWFDCMPW-LNFL  209
CYP53C__118598__Csu        156  LFAPKTVRLFEEDVQKYVGQLVAQWDDMC---ARAKDGVVLTGHNGAMEWSTREG-KVWFDCMPW-FNLL  220
CYP53H3__318972__Bad       136  AFAQKSVLEFEPVVKQYVGAVLKHWSHMCNAASSGDGGIIGDMK-----WTAQGG-RATFNVIKW-YNFM  198
CYP53H7__358536__Bad       140  TFSPKSVLEFEPIIWEYLGAVIRRWDQMCTAASQGRGGVVGEMT-----WSSEGG-CAVFDAVKW-YNFM  202
CYP53H6__142452__Bad       139  TFALKTVLEFEPVVKEYVGSIIKKWDRMCAAATKGEGGTIGEMT-----WISQGG-RAVFDTLKW-YNFM  201
CYP53H4__160054__Bad       139  TFSQKTTLEFEPVVRQYIGDMFKQWDRMCTAAVMGKGGVIGEMP-----WKDQDG-RAEFDTLKW-YNFM  201
CYP53H2__55123__Bad        139  TFARKTVLEFEPIVQRYIHAVVRQWEHMCAVAADGKGGVIGNAS-----WTSQDR-RAVFNTLKW-YNFM  201
CYP53H5__65034__Bad        136  TFSQKSTLEFEPMMGQHIGDLIRQWDYMCAMAAEGKGGVVGETA-----WTSHEG-RAVVDTLDW-YSFV  198
CYP53C__101826__Pca        111  AMSMKGIMEFEPNVREYQHMLVRQLDTLCAVGAQGIDGVLGSCP-----WTARDG-WVLFDCMPW-LNFA  173
CYP53C__256510__Pca        128  AMSVKGIMQFEPNVREHQQMLVKRLDTLCTVGAQGVDGVLGSCP-----WAARDG-WVWFDCMPW-FNFE  190
```

```
CYP53C__102576__Pca        140  AMSMKGITEFEPNVREYQHMLLKQLDTLCAVGAQGIDGVLGSCP-----WTTRDG-WVLFDCMPW-LNFD  202
CYP53C__212559__Pca        139  AMSMKYIMEFEPNVREHHHMLVKQLDTLCAAGAQGKDGILGTRP-----WTARDG-WAWFDCMPW-FNYE  201
CYP53D1__9015__Fox         135  SFAPKSSRAAEGPIADKVELLVRKWDEIIDKGPQ------------------FDG-FTQLECRRW-FTYL  184
CYP53A6__10234__Fgr        135  SFAPQSMRNFEPFIQQHLNVFLQKWDAMAANEAK------------------FDG-YADVESRVW-LNYL  184
Cyp53A__101387__Pst        144  AFAPKTIRSYTTTVHRFLEELLVKLDKRAALPGEPD--------------------KAPIDMLIW-SNYL  192
CYP53A__105834__Cpu        107  IFAAKSLQDCTPFIRDIVDSFVVQLDRLAAKN-------------------------EELNLLYW-FHFL  150
CYP53A__56813__Ade         126  PFSYQSIRGFEGFMRESLGRFVRRLDAVCAGECFGDA---------------VRP-GGAIDALLW-FNYL  178
CYP53__1116154__Fpi        138  PLTHK----LGHVLPDVVDEMIHSFNDIMPDAEHD---------------------WQTVPALET-MIKI  181
CYP53NS__152212__Pca       149  AFQPRTVHAQYPALHKYLLQFADTIDRSAAAR------------------------GRAVDLAQL-HVLL  193
CYP53NS__92916__Fme        146  AFQPITVNDQYPMLQRYLTEFIEVID-AAARS------------------------GSVIDLSTL-HVLL  189
Consensus_aa:                   hFt.pol..hEshhp..h..hhpphDphhs.s.ps..................pt...hh-hh.W.hs@h
Consensus_ss:                   hh hhhhhhhhhhhhhhhhhhhhhhhhhhhh                           eeehhhh hhhh


Conservation:                    7855658576 57565    5
CYP53A__2107910__Tte       185  AFDVIGDLAFGAPFGMLSSGADMAEVRAS-PDSP----------------------PIYAPAIEILNRRG  231
CYP53A15__ACF15219.1_Clu   179  AFDVIGDLAFGEPFGMLKKGRDEAEVARG-------------------------GKITYAPAIEVLNRRG  223
CYP53A23__71345__Mfi       179  AFDIIGDLAFGAPFGMLEKGADIAEVQLN-PDGP----------------------VTYAPAIEVLNRRG  225
CYP53A17__5031__Ure        182  AFDIIGDLAFGAPFGMLSKGRDVAEMKKS-PNSP----------------------ASYVPAIQVLNRRG  228
CYP53A18__8044__Cim        182  AFDIIGDLAFGAPFGMLSKGRDVAEMRKS-PDSP----------------------ASYVPAIQVLNRRG  228
CYP53A21__1341__Acl        184  AFDIIGDLAFGAPFGMLEKGKDFAEMRKT-PDSP----------------------PTYVEAIEVLNRRG  230
CYP53A12__8190__Nfi        184  AFDIIGDLAFGAPFGMLEKSKDIAEMRKA-PDSD----------------------PTYVQAVEVLNRRG  230
CYP53A12__3003__Afu        184  AFDIIGDLAFGAPFGMLEKGKDIAEMRKT-PDSE----------------------PTYVQAVEVLNRRG  230
CYP53A22__2015__Ate        185  AFDIIGDLAFGAPFGMLEKGQDIAEMRKS-ADAA----------------------PTYVQAVEVLNRRG  231
CYP53A13__5958__Aor        184  AFDIIGDLAFGAPFGMLEKGQDIAEMRKS-PNDK----------------------PSYVQAVEVLNRRG  230
CYP53A13__26719__Afl       184  AFDIIGDLAFGAPFGMLEKGQDIAEMRKS-PNDK----------------------PSYVQAVEVLNRRG  230
CYP53A1__50153__Anig       183  AFDIIGDLAFGAPFGMLDKGKDFAEMRKT-PDSP----------------------PSYVQAVEVLNRRG  229
CYP53A3__3425__Anid        189  AFDIIGDLAFGAPFGMLDKGQDIAEMRKS-PDSP----------------------PQYVQAVEVLNRRG  235
CYP53A__2301715__Mth       185  AFDVIGDLAFGAPFGMLSSGADMAEVRAS-PESP----------------------PIYAPAIEILNRRG  231
CYP53A4__88466__Ndi        185  AFDVIGDLSFGQPFGMLSSGADMAEIRSS-PDAA----------------------PIYAPAIEILNRRG  231
CYP53A4__7508__Ncr         184  AFDVIGDLSFGQPFGMLSSGADMAEIRSS-PDAA----------------------PIYAPAIEILNRRG  230
CYP53A19__9543__Fve        189  AFDVIGDLAFGVPFGMLANGADVAEVRAT-PDSA----------------------PIYASAIEILNRRG  235
CYP53A19__14206__Fox       189  AFDVIGDLAFGVPFGMLANGADVAEVRET-PDSA----------------------PIYASAIEILNRRG  235
CYP53A8__12085__Fgr        188  AFDVIGDLAFGVPFGMLASGADVAEVRDT-PDSP----------------------PIYASAIEILNRRG  234
CYP53A11__50496__Nhe       189  AFDVIGDLAFGAPFGMLANGADVAEVRAT-PESP----------------------PIYASAIEILNRRG  235
CYP53A20__6367__Fve        183  AFDIIADLAFGKPFGMLSTGADIAEVKVS-PTSP----------------------TIYAPAVEIMNRRG  229
CYP53A20__10443__Fox       183  AFDIIADLAFGKPFGMLSTGADIAEVKAS-PTSP----------------------TIYAPAVEIMNRRG  229
CYP53A7__10227__Fgr        183  AFDIIADLAFGKPFGMLASGADIAEVKAS-PTSP----------------------TIYAPAVEIMNRRG  229
CYP53A10__35086__Nhe       184  AFDVIADLAFGKPFGMLATGADIAEVKAS-PTSP----------------------AIYAPAVEIMNRRG  230
CYP53A5__120117__Mgrs      183  --------FFGAPFGMLNAGADIAEVRMS-VDSE----------------------PIYAPAVEILNRRG  221
CYP53A14__2107__Aor        197  TFDIIGDLAFGAPFGMLERENATVSMRKA-PENP----------------------EVTLDAVEVLNHRG  243
CYP53C__1025718__Fpi_      201  AFDIIGDLAFGAPFGMLDACADAAPVAIS-HEKAMSSYGETD-----------TPEITYFPAVQILNDRG  258
Cyp53C__68781__Pst         195  AFDIIGDLAFGQSFGMLHACKDSAPVALS-QDEAMKAYGSAS-----------GYKVVSIPAVQILNDRG  252
CYP53C__80617__Gtr         198  AFDIIGDLAFGSPFGMLKACKDSAPVAVS-HADAMAAYGKDD----------SAVQVRSLPAVQILNDRG  256
CYP53C__127772__Cpu        221  AFDIIGDLAFGSPFGMIHSAKDSAPVAVS-HADAMSAYGSSA----------SNIKVVHIPAVQILNDRG  279
CYP53C__94174__Shi         203  AFDIIGDLAFGSPFGMLQAAKDSAPVAKS-AKDAIAAYGQDE----------AKVEVVHIPAVQILNDRG  261
CYP53c__27029__Wco         201  AFDIIGDLAFGAPFGMLQACADAAPVVVS-HADAMASYGKGD-----------APEVAYFPAVQVLNDRG  258
CYP53C3__110015__Ppl       200  AFDIIGDLAFGAPFGMLHACADAAPVATE-HKDAMASYGADN-----------APKVTYFPAVQVLNDRG  257
CYP53C__116910__Csu        199  AFDIIGDLAFGAPFGMLHACADSAPVAIS-HEAAMKNYGDDA-----------APEVEHFPAVQVLNSRG  256
CYP53C__1179842__Sla       206  AFDIIGDLAFGSPFGMILNAKDSAPVAVS-QKDAMKSYGSES-----------TYEVIEIPAVQILNDRG  263
CYP53C__55106__Dsq         201  AFDIIGDLAFGAPFGMLDACKDSAPVAVS-HKAAMAAYGSSD-SSKE-------IQIEHFPAVQVLNDRG  261
```

```
CYP53C4__GL08839-P1.1__Gl  201  AFDIIGDLAFGAPFGMLLACKDSAPVAVS-CEAAMASYGSAS-SSKE-------IQIEHFPAVQVLNDRG  261
CYP53C4__47512__Gsp        201  AFDIIGDLAFGAPFGMLIACKDSAPVAVS-CEAAMASYGSAA-SSKE-------IQIEHFPAVQVLNDRG  261
CYP53C__129211__Tve        203  AFDIIGDLAFGAPFGMLTSCKDSAPVAVS-QDDAMATYGKDA-----------AYKVEHFPAVQVLNDRG  260
CYP53C9__27837__Pbr        202  AFDIIGDLAFGHPFGMLQACQDAAPVAVS-QEAAMAAYGEGK-----------QFEVTNIPAVRILNDRG  259
CYP53C7__118978__Bad       205  AFDIIGDLAFGSPFGMLQACRDAAPVAVS-QEDAMAGYGGKQ------------CDVVYIPAVQILNDRG  261
CYP53C__183109__Pca        201  AFDIIGDLAFGAPFGMLLAARDAAPVAVN-HEQAMASYGKEK------------SEVQYIPAVQVINDRG  257
CYP53C2__130996__Pch       186  AFDIIGDLAFGAPFGMLLAARDAAPVAVD-HEQAMASYGKEK------------SEVQYIPAVQVINDRG  242
CYP53C__128292__Fme        208  AFDIIGDLAFGSPFGMILKGKDAAPVAKD-QKAAIAGYGRESASEKSA------CDVTELPAVQVLNDRG  270
Cyp53C__37267__Tve         201  AFDIIGDLAFGAPFGMLTSGKDSAPIAVS-QVDAMAAYGQGG-----------TLKVKHVPAIQVINDRG  258
CYP53C__70450__Ade         197  AFDIIGDLAFGSPFGMLDACADSANAAVG-GVNAL---KDGK-----------PMQTVSVPAIRILNERG  251
CYP53C__52716__Dsp         204  AFDIIGDLAFGEPFGMINSGADSASVAIH-GDDPTHLASGEK-----------KLEIVRVPAVKILNDRG  261
CYP53C__83844__Cpu         206  AFDIIGDLAFGQPFGMILKAKDSAPVAVS-QDAAMDSYGKE-------------CKVIEVPAVKILNDRG  261
CYP53C__194303__Abi        204  AFDTIGDLAFGEPFGMLAAAKDMAVVPKD-QQSAMNSYGKET----------KEEDILTVPVIEAFNNRG  262
CYP53C__194181__Abi        208  AFDIIGDLAFGEPFGMLQAAKDSAVVPKD-QKSMMKSYGKED----------ASIEVMEIPAVQILNGRG  266
CYP53C__143663__Fme        204  AFDIIGDLAFGSPFGMVRNAKDAAPIAVD-RKSAMAQYGPVITDNRGLEK--PVIDVREVHAISVLENRM  270
CYP53C__130308__Fme        205  AFDIISDLAFALPFGMLRNAKDAALTAVD-QKAAMSENGQVNTDMQDIEK--PVVAVREVPAVKVLNGRS  271
CYP53C__24265__Fme         205  AFDIIGDLAFGAPFGMLRNAKDAAPTAVD-QKAAMSENGQVN--IQDLEK--PVVAVREVPAVKVLNGRS  269
CYP53C__149618__Fme        206  AFDVMSDLAFGASFGMVRNAKDAAPIAVD-QRAAMAQYQGTRVDSLDLEK--PSIDVKEVPAVTMLNAHI  272
CYP53C__94457__Fme         206  SFDIISDLAFGTPFGMIRKARDAVPVAID-HKAAMAQYGQIDTEYRDVKK--LVIDTREVPAIQVVNEQG  272
CYP53C__154594__Fme        198  TFDIIGDLAFGAPFGMIRKGKDAAPVAVD-LKAAIAQYGQAGIDGQDLEK--PAIQVKEVPAVQILNDRT  264
CYP53C__115179__Fme        205  CFDIIGDLVLGAPFNMVHKGTDTVPVALE-PSAVIAQYGQSS-ITGSHDTEKPICAVKEAPAMELMNGRS  272
CYP53C1__5__Uma_           207  AFDTIGALAFGKTFGMLENGVDQAKVEYE-DANGNK-------------------QVDYCSAVQIINERG  256
CYP53B2__32280__Sro        204  AFDVIGELAFGTPFGMVERDAADIVTITK-EDGT----------------------VIHAGGVQILNMRG  250
CYP53B3__28617__Pgr        200  AFDIIGDLAFGERFGMIERGADIAAVEKE-------------------------GKVIYLPAIQILNERG  244
CYP53C__48859__Fpi         210  SFDTIGDLAFGLPFGMLKSGRDTAKVAKS-AEDALKAIDTVS-KGGDVLA----IEEEEIPYIEYQSARA  273
CYP53C__86809__Fpi         210  SFDTIGDLAFGLPFGMLKSGRDTAKVAKS-AEEGFKAIDAMS-KGGDALV----VEEEEIPYIEYLSARA  273
CYP53C__138909__Wco        213  SFDTIGDLAFGSPFGMLSAASDTVRVAKS-VKASLATFGTSS-NAEEFGF-----ETEEMAIEKVLNERG  275
CYP53C__104840__Wco        215  SFDTIGDLAFGAPFGMLLSAKDTVRVATS-VKAGMAAFGTSS-TTGKFTL-----ETEEIPATKLLNKRA  277
CYP53C__77097__Wco         219  SFDTIGDLAFGAPFGMLLAAKDTARVAKS-VKAGLATFGTVS-RTGEFAF-----ETEEIPVTKLLNKRA  281
CYP53C__138864__Wco        213  SFDTIGDLAFGSPFGMLSAANDTVRVAKS-VKASLATFGTSS-NAGEFGF-----ETEEMAIEKMLSERG  275
CYP53C__104855__Wco        209  ------DLAFGAPFGMILAAKDTARFAKS-VMAGMAAFGTSS-KTSEYAF-----ETDETPVTKLMAERA  265
CYP53C__154264__Wco        192  SFDTIGDLAFGDPFGMILAAKDTARSAKS-VKASLETFSTSS-NTEKLAF-----ETEELPVTKIMGQRG  254
CYP53c__138853__Wco        213  SFDTIGDLAFGAPFGMILAAKDTARVAKS-VKASLATFGTPS-QTGKFAF-----ETEELPVIKIVGQRA  275
CYP53C__154237__Wco        114  SFDIIGDLAFGDPFGMILAAKDTARSAKS-VNASLATFSTSS-NTKKFAF-----EMEELPVTEIIGQRG  176
CYP53D1__108845__Ppl       223  SFDTISDLAFGHPFGMLEAAKDTAKISKS-NIKGMQAISQGN-SHSDEAE----LELEEIPAIEMLAEHL  286
CYP53D6__54877__Ppl        225  SFDTIGDLAFGHPFGMLETGKDVAQIAKS-NARGMQAIAQGT-SDSEKAT----LELVDIPAIEVLTARA  288
CYP53D4__55859__Ppl        196  SFDTIGDLAFGHPFGMLETGKDTAQTVKS-DVRGMEAIAQAT-SNSEKTK----LELVDIPAIEALTARA  259
CYP53D2v1__56013__Ppl      223  SFDTISDLAFGRPFGMLEAAKGSAHVSKS-NTKSVQAVSQDT-SHSNEAQ----SELLEIPAMEVLSELL  286
CYP53D2v2__48082__Ppl      223  SFDTISDLAFGRPFGMLEAAKGSAHVSKS-NTKSVQAVSQDT-SHSDEAQ----SELLEIPAMEVLSELL  286
CYP53D5__46728__Ppl        208  SFDSIADLSFGRPFGMLVSAKDVVRIPKS-NASGIQAIAEAA-SHSEKTE----LEMADVPLIEVLEIRG  271
CYP53D3__60352__Ppl        207  SFDTIADLSFGRPFGMLVSAKDVVRIPKS-NASGIQAIAEAA-SHSKKTE----LEMVEVPLIEVLVKRG  270
CYP53C__112429__Csu        208  AFDTIGDLAFGSPFGMLVSGKDTARIAKS-LKAAMQTLGSTP-SATEKPS---TIEEEDIPAISSINRRS  272
CYP53C__151209__Csu        210  SFDTIGDLAFGKAFGMVESGKDIARVAKD-YTDAMRTYNAKQ-ELPEWTP---AYEEEEIPAISSLKERS  274
CYP53C__118598__Csu        221  AFDTISDLAFGSPFGMLIAGRDTARVARS-VDIAMKNLGVAQ-TAQESDR---IYEEEDIPAISANNTRS  285
CYP53H3__318972__Bad       199  VFDIIGDLVFRAPFGMTEHGTDIARIAKN-RDHAMASYDSGE-VKL---------EYDTVNAVQALLSQN  257
CYP53H7__358536__Bad       203  VFDIIGHLVFRHPFGMTERATEMTLIVKQGRDDAMELQDKPG-REL---------EYTSIPAVQSINTRS  262
CYP53H6__142452__Bad       202  VFDIIGDLVFRAPFGMTERATEIAVIAKR-RDKAIESYETSE-QKL---------EYTTLPAVQAINKRG  260
CYP53H4__160054__Bad       202  VFDIIGDLVFRAPFSMTERGTDMARIVKR-PEKAMSSYESVD-TKL---------EYDTINAIEAVNARS  260
CYP53H2__55123__Bad        202  VFDIVGDLVFRNPFGMTERGSDMALIAKH-PDQVMASYNSIT-EEKI--------QYDAVNAVDAVTALN  261
CYP53H5__65034__Bad        199  IFDVIGELVFRIPFGMTNRGSDETLIVKH-PNQTMALDESSS-TKT---------EHDSVRAVQMMNERS  257
CYP53C__101826__Pca        174  TFDVIGDLAVGAPFGMLEAGKDTALVPVS-EEQAMKSFGQQD-TDL---------EWATIPTIKLLNETV  232
```

```
CYP53C__256510__Pca          191  TFDIIGDLAFGASFGMLEAGKDTAPVPVY-TDQAMKSYGQKD-TDL---------EWSTAPAVQILNEAI  249
CYP53C__102576__Pca          203  TFDVIGDLAFGKPFGMLEAGKDAALVPVS-EEQAMKSFGRQD-TDL---------KWATIPAIKLLNETV  261
CYP53C__212559__Pca          202  TFDIIGDLAFGAPFGMVEAGKDTASVPVS-EKQAMKFYGQKG-AEI---------EWSTAPAIKIVNEAV  260
CYP53D1__9015__Fox           185  SFDITGDLLFSEPFGMLENGSDLVKIDNK--------------------------PRSYVSMMNSLAQRS  228
CYP53A6__10234__Fgr          185  VLDIIGDLAFGAPFGVLAKGSEVVDFETE-------------------------KGPSSLPVITSLSTRS  229
Cyp53A__101387__Pst          193  VFDIMSTLAFGTPLGMLEKESDVLQAGSP------------------------------KGAIENTDVRE  232
CYP53A__105834__Cpu          151  AFDVLSDLAFGQRIGMVEKGSDAVTVQKR-DGS-----------------------VSTENAIALVDERE  196
CYP53A__56813__Ade           179  AFDIISDLAFGEPLGMVNKGSDLLPAERK-DGT-----------------------IFEEHAAALIDQRG  224
CYP53__1116154__Fpi          182  IARVTNRVFVGMPFCR---NEMLL-----------------------------------ETAVEFAKDVM  213
CYP53NS__152212__Pca         194  TLDFVGEVAFGAELRAVRDG-AACRILQI------------------------------------FHAVL  226
CYP53NS__92916__Fme          190  TLDFVGEVAFGAELNALRDG-ASCRILQI------------------------------------FHDIL  222
Consensus aa:                     sFDhlt-LtFG.PFGMl..tp-hh.h..p..p.s.........................hstlphhspp.
Consensus ss:                     hhhhhhhhh             hhhhhhh hhhhhh                   hhhhhhhhhhhhhhh

Conservation:                           7  6                                  5   5
CYP53A__2107910__Tte         232  EVSATLGILPQ-LKPY----AKYLP-DPFFSKGL-SAVEKLAGIAIARV---------KARLENPPP---  282
CYP53A15__ACF15219.1_Clu     224  EVSGTVGIFPA-IKPY----AKYFP-DPFFSQGM-KAVENLAGIAIARV---------NARLEKP-----  272
CYP53A23__71345__Mfi         226  EVSNAVGCWPA-IKPY----AKYLP-DPFFSKGM-EAIANLAGIATARV---------NQRLAAAERG--  277
CYP53A17__5031__Ure          229  EVSATLGCFPA-LKPF----AKYLP-DRFFRDGL-EAVENLAGIAVARV---------AERLRPEVMA--  280
CYP53A18__8044__Cim          229  EVSATLGCFPA-LKPF----AKYLP-DKFFRDGL-EAVEHLAGIAVARV---------SERLRPEVMA--  280
CYP53A21__1341__Acl          231  EVSAALGCFPR-LIPY----AKWIP-DRFFKDGL-QAVENLAGIAVARV---------NERLKPEVMA--  282
CYP53A12__8190__Nfi          231  EVSATLGCLPR-LIPY----AKYLP-DRFFKDGI-QAVENLAGIAVARV---------NERLKPEVME--  282
CYP53A12__3003__Afu          231  EVSATLGCLPR-LIPY----AKYLP-DRFFKDGV-QAVENLAGIAVARV---------NERLKPEVME--  282
CYP53A22__2015__Ate          232  EVSATLGTLPA-LIPY----AKYIP-DRFFKDGI-QAVENLAGIAIARV---------NERLRPEVMA--  283
CYP53A13__5958__Aor          231  EVSATLGACPS-LIPW----AKYIP-DRFFRDGL-EAVENLAGIAVARV---------NERLRPEVMA--  282
CYP53A13__26719__Afl         231  EVSATLGACPS-LIPW----AKYIP-DRFFRDGL-EAVENLAGIAVARV---------NERLRPEVMA--  282
CYP53A1__50153__Anig         230  EVSATLGCYPA-LKPF----AKYLP-DSFFRDGI-QAVEDLAGIAVARV---------NERLRPEVMA--  281
CYP53A3__3425__Anid          236  EVSATLGCYPA-LKPF----AKYLP-DRFFRDGL-EAVENLAGIAIACV---------NERLKPEVMA--  287
CYP53A__2301715__Mth         232  EVSATLGILPA-LKPY----AKYFP-DPFFSRGL-QAVENLAGIAIARV---------KARLENPPP---  282
CYP53A4__88466__Ndi          232  EVSATLGIHPA-LKPF----AKYLP-DPFFTKGL-AAVENLAGIAIACV---------KSRLENPPP---  282
CYP53A4__7508__Ncr           231  EVSATLGIHPA-LKPF----AKYLP-DPFFTKGL-AAVENLAGIAIACV---------KSRLDNPPP---  281
CYP53A19__9543__Fve          236  EVSATLGCWPQ-LKPY----AQWLP-DPFFSNGL-NAVKNLAGIAIARV---------KARLDNPPS---  286
CYP53A19__14206__Fox         236  EVSATLGCWPQ-LKPY----AQWLP-DPFFSNGL-NAVKNLAGIAIARV---------KARLDNPPS---  286
CYP53A8__12085__Fgr          235  EVSATLGCFPQ-LKPY----AQYLP-DPFFSNGL-NAVKNLAGIAIARV---------KNRLDNPPS---  285
CYP53A11__50496__Nhe         236  EVSATLGCFPQ-LKPY----AKWLP-DPFFSNGL-NAVQNLAGIAIARV---------KARLDNPPP---  286
CYP53A20__6367__Fve          230  EVSATLGCLPQ-LKPY----AKYLP-DPFFSQGL-QAVENLAGIAIARV---------SERLERGGD---  280
CYP53A20__10443__Fox         230  EVSATLGCLPQ-LKPY----AKYFP-DPFFSQGL-QAVENLAGIAIARV---------SERLERGGD---  280
CYP53A7__10227__Fgr          230  EVSATLGCLPQ-LKPY----AKYLP-DPFFSQGL-QAVENLAGIAIARV---------SERLERGGD---  280
CYP53A10__35086__Nhe         231  EVSATLGCMPW-LKPY----AKWLP-DPFFSQGL-QAVENLAGIAIARV---------SERLERGAD---  281
CYP53A5__120117__Mgrs        222  EVSATLGTLPE-LKPY----AGYLP-DSFFSKGL-AAVQNLAGIAIARV---------KSRLENPPD---  272
CYP53A14__2107__Aor          244  DVSAAFGICPD-LIPY----AKWLP-DPFFRQGA-EAIANLAGVAGAAV---------DRRLKMDTSM--  295
CYP53C__1025718__Fpi_        259  EYSASLGVLPPHWRPI----VKLL---PWYRKGN-KAVQRLAGIAIAQV---------AKRLAMH-----  306
Cyp53C__68781__Pst           253  EFSASIGVLPPAWRPF----VKNLI--PWYRNGS-KAVKNLAGLAVAAV---------AKRLDRDTLN--  304
CYP53C__80617__Gtr           257  EYSASMGVLPVWFRPV----VQRLH--PWYRNGN-KAVKDLAGLAVAAV---------AKRLRNP-----  305
CYP53C__127772__Cpu          280  EYSAAMGVLPPAIRPF----MQRFV--PWYRKGG-KAVRNLAGIAVAAV---------AKRLNEP-----  328
CYP53C__94174__Shi           262  EFSASMGVLPPWLRPY----VKRYI--PWFSKGD-QAVKNLAGLAIAAV---------SKRLNQP-----  310
CYP53c__27029__Wco           259  EFSASLGVLPPHWRPL----VVRFI--PWYRNGN-KAVKRLAGIAIAAV---------AKRLTAP-----  307
CYP53C3__110015__Ppl         258  EYSASMGVLPPHWRPL----VVRFI--PWYRNGG-KAVKRLAGIAIAAV---------SKRLTAP-----  306
CYP53C__116910__Csu          257  EYSASMGVLPPHWRPL----AKRI---PWFRRGN-QAVQRLAGIAVAAV---------AKRLSAP-----  304
CYP53C__1179842__Sla         264  EFSASMGVLPPHWRPL----VRLL---PWYRKGG-KAVKNLAGLAVAAV---------AKRLTTP-----  311
```

```
CYP53C__55106__Dsq         262  EYSAAMGVLPPHWRPL----AKKI---PWYSKGN-QAVQKLAGIAVAAV---------AQRFANP-----  309
CYP53C4__GL08839-P1.1__Gl  262  EYSASMGVLPPHWRPL----AKRI---PWFAKGN-QAVQRLAGIAVAAV---------AQRLGSP-----  309
CYP53C4__47512__Gsp        262  EYSASMGVLPPHWRPL----AKRI---PWYARGN-QAVQRLAGIAVAAV---------AQRMASP-----  309
CYP53C__129211__Tve        261  EYSASMGVVPPRWRPL----VKRL---PWYNKGN-QAVQRLAGIAIAAV---------ARRLSVPE----  309
CYP53C9__27837__Pbr        260  MFSASLGVLPPWMRPL----AKQL---PWFKKGN-AAVKTLAGIAVAAV---------ARRLATP-----  307
CYP53C7__118978__Bad       262  NFSASLGVLPPWMRPI----VKQL---PWFKKGQ-KAVKDLAGIAIAAV---------AKRLTTP-----  309
CYP53C__183109__Pca        258  MYSASLGVLAPWMRPI----VKLF---PWFRQGQ-QAVKLLAGIAVAAV---------SQRLTTP-----  305
CYP53C2__130996__Pch       243  TYSASLGVLPPWMRPI----VKLF---PWFRRGQ-KAVKQLAGIAVAAV---------AQRLTTP-----  290
CYP53C__128292__Fme        271  EYSASMGVLPPWWRPF----VRRI---PWYANGN-RAVKNLAGLAVAAV---------AKRLANP-----  318
Cyp53C__37267__Tve         259  EYAASVGVLPPHWRPF----VKRL---PWYNTGD-KAVQNLTGMAIAAV---------ARRMEESD----  307
CYP53C__70450__Ade         252  EFSATMGVLAPWMRPL----VLKL---PWFARGL-SAVRALAGLAIAAV---------GRRLAEP-----  299
CYP53C__52716__Dsp         262  EYSASMGCLPIWIRPY----AKKI---PWYAKGN-QAVKNLAGIAIAAV---------DKRLATP-----  309
CYP53C__83844__Cpu         262  DYNATLGTMPPWVRPY----VRKL---PWFSQGS-EAAASVAGMAVAAV---------SRRLTTP-----  309
CYP53C__194303__Abi        263  EFNLVMGSLPLHWRPL----ARRL---PGLAQGS-RDFKTVAGIAVAAA---------SKRLSSS-----  310
CYP53C__194181__Abi        267  EFSLTMGTLPPYWRPI----ARRL---PGFRQGA-QDVKNLAGIAIAAV---------AKRLATP-----  314
CYP53C__143663__Fme        271  RLSAQMGVLPAWWRPI----VRQL---PRFAQGV-QNSKDLVDLAVAAV---------AKRMAYP-----  318
CYP53C__130308__Fme        272  EYSASMGVLPPWWRPI----VRLL---PWYADGS-QDVEDLAGLAVAAV---------AKRLAIP-----  319
CYP53C__24265__Fme         270  EYSASMGVLPPRWRPI----ARLL---PWYAEGS-KDVEDLAGLAVAAV---------AKRLAIP-----  317
CYP53C__149618__Fme        273  KVSARMAAVPPRWRPI----LQRL---PCFARDM-RASEDLVALAVAAV---------ARRLVFP-----  320
CYP53C__94457__Fme         273  EGVAQIAAFPPLWVPF----LRCL---PRFAKGM-RRVEDFIGLVVLAV---------ANRLAFP-----  320
CYP53C__154594__Fme        265  FLASHQAAFPKPLRPL----LALL---PQYAEMA-KHSDEFIGFAVAAV---------AKRLVFP-----  312
CYP53C__115179__Fme        273  AVIASLGVLPPWWRPI----ASLF---PWYARGN-RDVGDLAGFATLAI---------SKRLARP-----  320
CYP53C1__5__Uma_           257  EFSGTMGLAPVWMRPY----LIKL---PWFSSRL-KSVKKLTGIALARV---------NDRLQNG-----  304
CYP53B2__32280__Sro        251  EYSATLGCLPPWSRKY-----MKYI-DPWFARGL-ESVKNLTGIARTRV---------NDRLEKGA----  300
CYP53B3__28617__Pgr        245  EFSATQGQFDHIKFSKKKKEKKKYI-DPWFSRGA-ASVQNLTGIATNQV---------NLRISQTG----  299
CYP53C__48859__Fpi         274  ETDACLAWLPPIWVRI----LGKL---PMFSVHA-LTGQKLAALSIMAV---------ARRIADP-----  321
CYP53C__86809__Fpi         274  ERNACLAWLPPVWARV----VLTL---PAFSGYA-LTGRKLAALSIMAV---------ARRLANP-----  321
CYP53C__138909__Wco        276  HLITILGWLPEYWRRI----VQML---PAYRFGM-EAAPKMAGLAVAAV---------GKRLNNP-----  323
CYP53C__104840__Wco        278  KLVTTLGWFPKYWQPI----IELL---PPFRAGR-EATPKLAGLAVAAV---------AKRLSNP-----  325
CYP53C__77097__Wco         282  ELVAILGWLPKYWQSI----VGTL---AVFSGGS-NASPKLAGLAVASV---------AKRLSNP-----  329
CYP53C__138864__Wco        276  HLISILGWLPEYWRRI----VQML---PAYRFGT-EAAPKMAGLAVAAV---------GKRLNNP-----  323
CYP53C__104855__Wco        266  DLVATIGWLPEYWQPI----VRML---PAFRGGR-KSTPQLAGLAVAAV---------AKRLSKP-----  313
CYP53C__154264__Wco        255  ELVAMLGWLPEYVRPI----ILMM---PGFRSNL-QAIPKVAGLAVAAV---------AKRMNNP-----  302
CYP53c__138853__Wco        276  ELVAMFGWLPEYVWPI----ILMM---PGFRGSR-RAIPQVSGLAVAAV---------AKRMNNP-----  323
CYP53C__154237__Wco        177  ELITMLGWLPKYVRPI----ILMM---PGFRSNL-QAIPKVAGSAVTAV---------AKRMNDL-----  224
CYP53D1__108845__Ppl       287  DIIVSLAFLPAWLQPI----VGRL---PSVRYGY-DAAPKLAGLAVAAV---------ANRFASKTK---  336
CYP53D6__54877__Ppl        289  DALFVVAYLPPWAQKI----VGRL---PSFRSGY-AAAPKLAGMAVAAV---------ANRLASQY----  337
CYP53D4__55859__Ppl        260  DTLFVVAYLPAWLQPI----VGHL---PSLQSGY-NAAPKLAGLAVAAV---------ANKFASKT----  308
CYP53D2v1__56013__Ppl      287  DFTVALAYLPAWVQPV----FGRL---PMFRDGY-DAAPKLANLSLTAV---------ANRVASQT----  335
CYP53D2v2__48082__Ppl      287  DFTVALAYLPAWVQPV----FGRL---PMFRDGY-DAAPKLANLSLTAV---------ANRVASQT----  335
CYP53D5__46728__Ppl        272  KTVAALAYLPAWAQPI----IGRL---PGFREGY-GAIPKLNGIAIAAV---------ANRLRSPN----  320
CYP53D3__60352__Ppl        271  KTIAALAYLPAWAQPI----IGRL---PGFREGY-GAIPKLNGIAIAAV---------ADRLRSPN----  319
CYP53C__112429__Csu        273  EFLIAFASLPAWIRPI----VKRL---PMASDGM-AATREIMSMAVTTV---------SRRVRTLYDGD-  324
CYP53C__151209__Csu        275  KYMIFVGTLPKSLRGI----MRFL---RSRDHQV-MALRRIMSMATSSV---------ARRIRAGRE---  324
CYP53C__118598__Csu        286  DFLMFLAYLPEWLRPI----VVRL---PLFDDGV-AAAGKIMSMAVTTV---------LRRLHALSSDSG  338
CYP53H3__318972__Bad       258  KFLTVVAQLPAYLRPL----LQSVP-LPWVRSGA-EAFQKFSTLSVTAV---------ANSQQYP-----  307
CYP53H7__358536__Bad       263  TFLATMGMLPKSWRPL----LRKLP-LSWFNGGH-LASEFMLSLAMTAV---------SHRLQHQ-----  312
CYP53H6__142452__Bad       261  TFLATMGMLPKYWWPL----LRKLP-LSWFSAGH-AAAEMMLTLAITAV---------AHRSQHN-----  310
CYP53H4__160054__Bad       261  AFLITLGVLPALWRPL----IKKLP-LPWVRAGT-EAFQKITKLTVTVV---------ADRSHHS-----  310
CYP53H2__55123__Bad        262  VVLTAVGMLRPFWRPF----ILKLP-MPWIRAGN-EGVAKIAMMAVAAV---------AQCSQHP-----  311
CYP53H5__65034__Bad        258  ALMVSLGMLPKYWRPL----IERLP-MEWIRRGN-AGVEKMSSLVMSAI---------ADGSKNS-----  307
```

```
CYP53C__101826__Pca        233  PWIFFLGCLP-----------------PQARFLM-SKLQSFNAGASRKLFQKLGVAAVSKRLSSE-----  279
CYP53C__256510__Pca        250  PWFFFLGCLP-----------------PQARRLV-STLQSFNAGGSRNLIGKIAVAAVSKRLTSE-----  296
CYP53C__102576__Pca        262  PWTFFLGCLP-----------------PQARFLM-SKLPSFNAGTSRKLFVKLAVATVSKRLASE-----  308
CYP53C__212559__Pca        261  PWVFFLGCLP-----------------PHVRPLL-SKLRSFTTKASARALVKIAVAAVSKRLATG-----  307
CYP53D1__9015__Fox         229  AAVATLGVLPWL-KPH----AHHLP-DPFFHRGM-DGLQNLLGVTSAHV---------KERMAAGQD---  279
CYP53A6__10234__Fgr        230  EIAATVGALPEL-KPY----LKWSP-DPFFRTGF-NGMINLRTLGTSRI---------TDRLNNPPG---  280
Cyp53A__101387__Pst        233  HYLTVIGWAPAL-AYI----ARLIP-DPFFQKGS-KSSDELRDIARMCI---------KQRLASGSD---  283
CYP53A__105834__Cpu        197  HLGAVLGVHPSF-KFW----SKFLP-DPFFIQGR-KSSDGLVDFARRQV---------SRRIDNR-----  245
CYP53A__56813__Ade         225  RTAAVVGLMPSI-EDI----TKKLP-IPFITAGY-KSTESLSRIAVRCV---------KHRIQSG-----  273
CYP53__1116154__Fpi        214  KTKFIVNLFPDVLKPI----VGHR--LPWTTARRRKMAEILGDTVRERR---------RQMLEYGTDY--  266
CYP53NS__152212__Pca       227  PELMKCGLFPL---------RAKVPVFESTRAMH-RAIAELRGMARAAV---------EDARRA------  271
CYP53NS__92916__Fme        223  PELMKCGLFPL---------RSQIPIMESTRRMH-RSIKELRGMAHVAV---------KNAR--------  265
Consensus aa:                   .h.h.hthhP..hpPh....h..h...s.hp....psh.pl.sht...V..........pRh........
Consensus ss:                   hhhhhhhhhhhhhhhh    hhh    hhhhhhh hhhhhhhhhhhhhh         hhhhh


Conservation:                          8 8  6            6         66  5      5 66 5656 5  5  5
CYP53A__2107910__Tte       283  ---ASRKDLLQRLIDGRDEK----GQPL---GREELTAE-ALTQLIAGSDTTSNSSCALLFHAARTPGVL  341
CYP53A15__ACF15219.1_Clu   273  ---SDRVDLLARLMEGRDEN----GNKL---GREELTAE-ALTQLIAGSDTTSNTSCALLYHCLQHPEVV  331
CYP53A23__71345__Mfi       278  --EIDRVDLLARLMEGKDEN----GNKL---AKAELTAE-ALTQLIAGSDTTSNTSCALLFHCLKNPHVV  337
CYP53A17__5031__Ure        281  --KNTRVDLLSRLMEGRDET----GAKL---GREELTAE-ALTQLIAGSDTTSNTSCAMLYWVLRTPGVI  340
CYP53A18__8044__Cim        281  --KNTRVDLLSRLMEGRDET----GAKL---GREELTAE-ALTQLIAGSDTTSNTSCAMLYWVLRTPGVI  340
CYP53A21__1341__Acl        283  --NNTRVDLLSRLMEGKDSN----GNKL---GREELTAE-ALTQLIAGSDTTSNTACAILYWCMQTPGVI  342
CYP53A12__8190__Nfi        283  --KNTRVDLLSRLMEGKDSN----GNKL---GREELTAE-ALTQLIAGSDTTSNTTCAILYWCMSTPGVI  342
CYP53A12__3003__Afu        283  --KNTRVDLLSRLMEGKDSN----GNKL---GREELTAE-ALTQLIAGSDTTSNTTCAILYWCMSTPGVI  342
CYP53A22__2015__Ate        284  --NNTRVDLLARLMEGKDAN----GNKL---GREELTAE-ALTQLIAGSDTTSNTACAILYWCMSTPGVI  343
CYP53A13__5958__Aor        283  --NNTRVDLLARLMEGKDSN----GNKL---GREELTAE-ALTQLIAGSDTTSNTSCAILYWCLRTPGVI  342
CYP53A13__26719__Afl       283  --NNTRVDLLARLMEGKDSN----GNKL---GREELTAE-ALTQLIAGSDTTSNTSCAILYWCLRTPGVI  342
CYP53A1__50153__Anig       282  --NNTRVDLLARLMEGKDSN----GEKL---GRAELTAE-ALTQLIAGSDTTSNTSCAILYWCMRTPGVI  341
CYP53A3__3425__Anid        288  --NNTRVDLLARLMEGKDAN----GNKL---GRAELTAE-ALTQLIAGSDTTSNTSCAILYYCLRTPGVI  347
CYP53A__2301715__Mth       283  ---SHRKDLLQRLIEGRDEK----GEPL---GRQELTAE-ALTQLIAGSDTTSNSSCALLYHAVRTPGVM  341
CYP53A4__88466__Ndi        283  ---VTRKDLLQRLMEGRDEK----GEPL---SREELTAE-ALTQLIAGSDTTSNSSCALLFHAVRTPGVM  341
CYP53A4__7508__Ncr         282  ---VTRKDLLQRLMEGRDEK----GEPL---GREELTAE-ALTQLIAGSDTTSNSSCALLFHAVRTPGVM  340
CYP53A19__9543__Fve        287  ---VERKDLLARLMEGRDEK----GEPL---GREELTAE-ALTQLIAGSDTTSNSSCALLYHVTRTPGVL  345
CYP53A19__14206__Fox       287  ---VERKDLLARLMEGRDEK----GEPL---GREELTAE-ALTQLIAGSDTTSNSSCALLYHVTRTPGVL  345
CYP53A8__12085__Fgr        286  ---IERMDLLARLMEGRDEK----GEPL---GREELTAE-ALTQLIAGSDTTSNSSCALLYHVTRTPGVL  344
CYP53A11__50496__Nhe       287  ---EERMDLLARLMEGRDEK----GEPL---GREELTAE-ALTQLIAGSDTTSNSSCALLYHVTRTPGVL  345
CYP53A20__6367__Fve        281  ---STRKDLLARLMQGRDEK----GEPL---GRDELTAE-ALTQLIAGSDTTSNSSCALLYHVVRTPGVM  339
CYP53A20__10443__Fox       281  ---STRKDLLARLMQGRDEK----GEPL---GRDELTAE-ALTQLIAGSDTTSNSSCALLYHVVRTPGVM  339
CYP53A7__10227__Fgr        281  ---STRKDLLARLMQGRDEK----GEPL---GRDELTAE-ALTQLIAGSDTTSNSSCALLYHIVRTPGVM  339
CYP53A10__35086__Nhe       282  ---TTRKDLLARLMQGRDEK----GEPL---GRDELTAE-ALTQLIAGSDTTSNSSCALLYHVVKTPGVL  340
CYP53A5__120117__Mgrs      273  ---VNRKDLLARLQEGRDAK----GEPL---GFEELTAE-ALTQLIAGSDTTSNSSCALLYWTARTPGVL  331
CYP53A14__2107__Aor        296  --TEKRGDLLALLIDAEDQA----GAKL---GHRELTGE-AVTLIAAGSDTSSSTLCALLYWVSSTPRVL  355
CYP53C__1025718__Fpi_      307  ---TDRSDLLGKLQEGKDDE----GNPM---GREELTAE-ALTQLIAGSDTTSNSSCAITYHLAANPMVQ  365
Cyp53C__68781__Pst         305  -GGSDRVDLLAKLQQGKDDE----GKPM---GREELTAE-ALTQLIAGSDTTSNSSCAITYHLAANPNVQ  365
CYP53C__80617__Gtr         306  ---TDRVDLLSKLQEGKDDE----GKPM---GREELTAE-ALTQLIAGSDTTSNSSCAITYYLALHPRVQ  364
CYP53C__127772__Cpu        329  ---SDRVDLLRRLQEAKDDE----GNPM---GREELTAE-ALTQLIAGSDTTSNSSCAITYYLALHPEIQ  387
CYP53C__94174__Shi         311  ---TYRVDLLSKLQEGKDDE----GRPM---GREELTAE-ALTQLIAGSDTTSNTSCAITYYLAANPAVQ  369
CYP53c__27029__Wco         308  ---TDRSDLLAKLQEGKDDD----GNPM---GREELTAE-ALTQLIAGSDTTSNSSCAITYYLAANPLVQ  366
CYP53C3__110015__Ppl       307  ---TDRADLLGKLQEGKDDE----GNPM---GREELTAE-ALTQLIAGSDTTSNSSCALTYHLAANPRVQ  365
CYP53C__116910__Csu        305  ---SDRTDLLSKLQEGKDDE----GKLM---GKPELTAE-ALTQLIAGSDTTSNSSCAITYHLAANPHVQ  363
```

```
CYP53C__1179842__Sla       312  ---TDRVDLLSKLQEGRDDE----GKLM---GREELTAE-ALTQLIAGSDTTSNSSCAITYYLAQNPDAQ  370
CYP53C__55106__Dsq         310  ---SDRADLLSKLQEGRDDN----GDPM---GREELTAE-ALTQLIAGSDTTSNSSCALTYWLAKNQAAQ  368
CYP53C4__GL08839-P1.1__Gl  310  ---SDRVDLLAKLQEGRDDN----GDPM---GREELTAE-ALTQLIAGSDTTSNSSCAITYWLARNQAAQ  368
CYP53C4__47512__Gsp        310  ---SDRVDLLAKLQEGRDDN----GDPM---GREELTAE-ALTQLIAGSDTTSNSSCAITYWLARNPAAQ  368
CYP53C__129211__Tve        310  ---SDRHDLLEKLQEGRDDN----GDPM---GRAELTAE-ALTQLIAGSDTTSNSSCAITYYLAKYQHVQ  368
CYP53C9__27837__Pbr        308  ---VDRVDLLGKLQDGRDDE----GNPM---GREELTAE-ALTQLIAGSDTTSNSSCAITYHLAKNPDVQ  366
CYP53C7__118978__Bad       310  ---SDRTDLLGKLQQGRDDE----GNPM---GRPELTAE-ALTQLIAGSDTTSNSSCAITYHLAKNPEVQ  368
CYP53C__183109__Pca        306  ---TDRVDLLGKLQGGRDDD----GNLM---GKEELTAE-ALTQLIAGSDTTSNSSCAITYYLAKYPDVQ  364
CYP53C2__130996__Pch       291  ---TDRVDLLGKLQEGRDDD----GNLM---GKEELTAE-ALTQLIAGSDTTSNSSCAITYYLAKYPDAQ  349
CYP53C__128292__Fme        319  ---TDRTDLLSKLQEGKDDE----GRPM---GREELTAE-ALTQLIAGSDTTSNSSCAITYHLAHNPHVL  377
Cyp53C__37267__Tve         308  ---SDHRDLLAKLREARDED----GNPM---GREELTAE-ALAQLVAGSDTTSNSSCAITYYLAKHQRVQ  366
CYP53C__70450__Ade         300  ---SDRNDLLAKLQDAKDDD----GMPM---GPEELTAE-ALTQLIAGSDTTSNSSCAIAYYVARYPRVQ  358
CYP53C__52716__Dsp         310  ---TDRVDLLARLQQGKDEQ----GNLM---ARSELTAE-ALAQLIAGSDTTSNSSCAITYWLAKYPDAQ  368
CYP53C__83844__Cpu         310  ---TDRVDILSKLQQGKDEN----GEIM---GPEELTAE-ALTHLVAGSDTTANSSCAIIYYLAAYPHVQ  368
CYP53C__194303__Abi        311  ---TDRIDLMSKLQNSRDSN----GNPM---SREEMTAE-ALTLLVAGSDTSSNACAAFLYHVAANPSVQ  369
CYP53C__194181__Abi        315  ---TDRNDLLSNLQAGRDSE----GKPL---GREELTAE-ALTLLIAGSDTTSNSTCAILYYLARNRGAQ  373
CYP53C__143663__Fme        319  ---TQRDDILSKLQQSRDEY----GRPL---TQEDLTTD-AITQLVAGSDTISISSCGIAYHLAANPDVQ  377
CYP53C__130308__Fme        320  ---TDRTDLLSKLQQGRHED----GRPL---NREELTAD-ALTQLIAGSDTTANSSCAVLYHIISSPRVQ  378
CYP53C__24265__Fme         318  ---TDRADILSKLQQGRHED----GSPM---SREELTAD-ALTVLIAGSDTTSNSTCALMYYITSNPRVQ  376
CYP53C__149618__Fme        321  ---AERIDVLSKLQETEDEH----GRVS---NMEDLTTD-AFTQLVAGSDTVSSTACGIAHCVAANSRVR  379
CYP53C__94457__Fme         321  ---TERVDILSKLQQGKGED----GVPL---TKEELTSE-ALVQLIAGSDTTSNTTCAITYYVAANPHVQ  379
CYP53C__154594__Fme        313  ---TERVDILSKLQQSKDEN----GNPQ---SREDLTTD-GITQLVAGSDTVANTSCGITYHIASNPCVQ  371
CYP53C__115179__Fme        321  ---TEPLGLLSALLELKDDE----GKPL---SKEQLSAD-GLLLLIAGSDMVANPTCAVLYQIIANPPVQ  379
CYP53C1__5__Uma_           305  ---SEREDLLAKLQAAKDDR----GEPM---GKMELTAE-ALTQLIAGSDTTSNTSCAIVYHLATHPDKM  363
CYP53B2__32280__Sro        301  ---LDRKDILSHLQAGRDEN----GQPM---SKDELTME-ALTQLIAGSDTTSNSSCAILFQIVSTPHAH  359
CYP53B3__28617__Pgr        300  ---QSRRDLLARLQTGQDAD----GNPM---GKDELIAE-ALTQLIAGSDTTSNSSCAILWWVVKHPEVH  358
CYP53C__48859__Fpi         322  ---NPREDMLQKLLEARDEE----GKPL---SPQEMSAE-AFVLIIAGSDTIANTTCGTTYYLARDKRVQ  380
CYP53C__86809__Fpi         322  ---NPREDMLQKLLEARDEE----GKPL---SPQEMSSE-AFLLIIAGSDTIANTTCGTTYYLARDKRVQ  380
CYP53C__138909__Wco        324  ---AAREDMLNRLLDARDEN----GKPM---SPEELSAE-AFVLIIAGADTTANTSCATTYYLARDQRVQ  382
CYP53C__104840__Wco        326  ---DAREDMLNRLLDARDEN----GEPL---SPEELSAE-AWLLIIAGADTVANTSCATTYYLARDQRVQ  384
CYP53C__77097__Wco         330  ---QAREDMLNRLLDARDDN----GEPM---SPEELSAE-AMTLIIAGADTVANTSCATTYYLARNQRVQ  388
CYP53C__138864__Wco        324  ---AAREDMLNRLLDARDEN----GKPM---SPEELSAD-AFQLIVAGADTTANTSCATTYYLARDQRVQ  382
CYP53C__104855__Wco        314  ---DAREDMLNRLLNARDED----DEPL---SREELSAE-AAMLIAAGADTVANTSCATTYYLARDQRVQ  372
CYP53C__154264__Wco        303  ---DARADMLNRLLDA-----------------------------PGAITVANTSCATTYYLARDQRVQ  339
CYP53c__138853__Wco        324  ---DARADMLNKLLDARDEN----GEPM---SPEELSSE-AFLLIVAGSDTVSNTSCATTYYLARDQRVQ  382
CYP53C__154237__Wco        225  ---DAHADMLNRLLDARDEN----GEPM---SPEELSSE-ASLIIVAGAITVANTSCAITYYLARDQRVQ  283
CYP53D1__108845__Ppl       337  ---IDRADMLSELLRGRDED----GKPY---GPEELSAE-AELLIIAGGDTTANSSCATTYHLARNPRIR  395
CYP53D6__54877__Ppl        338  ----DRADMLSKLLQGRDED----GKPY---SPEELSAE-AWVLIIAGGDTTANSSCALTYHLARNPRVQ  395
CYP53D4__55859__Ppl        309  ----DRADMLSKLLEGRDKN----GNLY---GPEELSAE-TWLLIIAGGDTTANTSCATTYYLARNPRIQ  366
CYP53D2v1__56013__Ppl      336  ----DRADMLSELLRGRDEE----GKPY---GLEELSTE-AELLIIAGGDTTANTSCATAYYIARDLQIQ  393
CYP53D2v2__48082__Ppl      336  ----DRADMLSELLRGRDEE----GKPY---GLEELSTE-AELLIIAGGDTTANTSCATAYYIARDLQIQ  393
CYP53D5__46728__Ppl        321  ----GRADMLTKLLEGRDGD----GHSY---SPQELSAE-ARTLIAAGGDTTASASCAITYYIARDPRIQ  378
CYP53D3__60352__Ppl        320  ----GRADMLTKLLEGRDGE----GYRY---GPQELSAE-AKTLIAAGGDTTASASCAITYYIARDPRIQ  377
CYP53C__112429__Csu        325  AEERQRPDFLTKLLEGRDEE----GSPL---SPDELSSE-AQTLLIAGSDTISNSTCAIVYWIARNPDVQ  386
CYP53C__151209__Csu        325  ---QDRDDFLARLLQARDDD----GNPL---SPDELSSE-AQTLLTAGADTISNSTCATVFWIARAPPVK  383
CYP53C__118598__Csu        339  DEKKNYEDFLIKLLQGHNDD----GNRM---GPEELTSE-AQVLLIAGSDTISNSTCATVYWVARHLNVQ  400
CYP53H3__318972__Bad       308  ---APRNDILGRYFEATDEK----GQKM---GNHELSSE-AVSLLIAGTDTTSNSAAALTFYLAHNPAAQ  366
CYP53H7__358536__Bad       313  ---TTFDDILGKYLEATDDR----GQKM---NDEELIAE-ALTLLIGGTDTTSSTVAALTFYLAQNPVVQ  371
CYP53H6__142452__Bad       311  ---AIRSDILGKYMEARDDR----GQQL---DNRELSSE-ALTLLIGGTDTTSNSAAALTFYLAQNTIVQ  369
CYP53H4__160054__Bad       311  ---ATRNDILAKYFDATDEN----GQKL---DAQELSSE-AITLLIAGTDTTSNSVAAMTFYIAHNPVVQ  369
CYP53H2__55123__Bad        312  ---ASRNDILAKYFDAIDDR----GEKM---HDLELSAE-AVGLLIAGTDTTSNSLGALTFYLAQHPSAQ  370
```

```
CYP53H5__65034__Bad         308  ---SQRNDILSKYFNATDED----GRKM---GISELYTE-ALVLLAAGADTTAHSLAALTFYLAQCPAAQ  366
CYP53C__101826__Pca         280  ---ATRRDFLSQLVAARDDE----GKPL---SAQELTSE-ALNLIIAGSDTTSSSIGAIIYHIARNRDVQ  338
CYP53C__256510__Pca         297  ---VTRRDFLSHLVAAHDDQ----GRPL---SQQELTSE-AISLIVAGSDTTSTSIAAITYHVARTQDVQ  355
CYP53C__102576__Pca         309  ---ATRRDFLSHLVAARNDE----GKPL---SAQELTAE-ALNLIVGGSDTTSSSIGVVIYHVARNRDVQ  367
CYP53C__212559__Pca         308  ---VTRRDFLSHLIAVRDDQ----GRPL---TEQELTSE-AISLIVAGSDTTSSSIAAIAYHVARNQDVQ  366
CYP53D1__9015__Fox          280  ----NHDDWLSLLLRARDDQ----GELL---KFEEIASE-SLTLFMAAIETVSNTLSAMMYYLATSPSSL  337
CYP53A6__10234__Fgr         281  --DEREKDLLERVREGRDHK----GQPF---GKGELIAE-ALTVLIAGTDTTSSTMAALLYHVVRTPGVL  340
Cyp53A__101387__Pst         284  ---DAKSDILGHLIAAHMEY----KNHL---DVEELTSE-ALTLLIAGTDATSNAITAIIHALSVNPRPL  342
CYP53A__105834__Cpu         246  ---LQRNDILDKLIRARVAD----DQEIVGENFADLVAE-TVTLLIAGSDTTSNSETAIMHLLFTNPRVY  307
CYP53A__56813__Ade          274  ---VTRDDMLERLIDGVREK---QGGEV---SEEEVVTE-AMLLLTAGADTTANSLTAILYFILTRPDVY  333
CYP53__1116154__Fpi         267  --EGKPDDYLTWVVEEDLKN---RGKGE---SIDGVMEV-IAASNFAAIHTSSMAMAHALYYLCAMPQYI  327
CYP53NS__152212__Pca        272  -------------HEDSQEKDCGAEKGK---KIFEILAH-HVPRRRRRSHGTHDDFCRG-HRQLRNPAIH  323
CYP53NS__92916__Fme         266  ---------------SSEEKSAVQPGSK---RIYEILAQLSVNLKLLHVLVT---FLPL-LFQLRNPEIL  313
Consensus_aa:                    ...sp+.DhLs+Lbptcccp....Gp.h...s.pEl.t-.thhblhAGs-Thtso.tthh@@lh.p..hb
Consensus_ss:                            hhhhhhhhh               hhhhhhh hhhhhh   hhhhhhhhhhhhhhhh hhhh


Conservation:                     68   667                             7 77   55 9 68   5  5 7868        5
CYP53A__2107910__Tte        342  ARLRAELDAAVPAD------------LVVPTFDLVRDLPYLSAVVNETLRFHSTSGIGLPREVPRDG-QG  398
CYP53A15__ACF15219.1_Clu    332  QKLQNELDAALPNP------------DAVPSYAQVKDLPYVDAVIKETMRIHSTSSLGLPRVIPPG--PG  387
CYP53A23__71345__Mfi        338  KKLQAELDEALPSD-------------DVPTYEQVKNLQYLDQVISETLRIHSTSSQGLPRVVPPG--DG  392
CYP53A17__5031__Ure         341  EKLQEVLDEAIPAH------------VEVPTFSMVKDIPYLQWVILETMRIHSTSSLGLPREIPQGS-PP  397
CYP53A18__8044__Cim         341  EKLQEALDEAVPAH------------VNVPSFSMVRDIPYLQWVIWETMRIHSTSSLGLPREIPPNS-PP  397
CYP53A21__1341__Acl         343  TKLQKVLDEAIPAD------------VDVPTHSMVKEIPYLQWVIWETMRIHSTSSMGLPREIPPGN-PP  399
CYP53A12__8190__Nfi         343  PKLQKVLDEAIPDD------------VDVPTHAMVKDIPYLQWVIWETMRIHSTSAMGLPREIPPGN-PP  399
CYP53A12__3003__Afu         343  PKLQKVLDEAIPDD------------VDVPTHAMVKDIPYLQWVIWETMRIHSTSAMGLPREIPPGN-PP  399
CYP53A22__2015__Ate         344  DKLHKVLDEAIPAD------------VDVPTHSMVKDIPYLQWVIWETMRIHSTSAMGLPREIPAGS-PP  400
CYP53A13__5958__Aor         343  EKLHKVLDESIPKD------------VDVPVHAMVKDIPYLQWVIWETMRIHSTSAMGLPREIPAGN-PP  399
CYP53A13__26719__Afl        343  EKLHKVLDESIPKD------------VDVPVHAMVKDIPYLQWVIWETMRIHSTSAMGLPREIPAGN-PP  399
CYP53A1__50153__Anig        342  EKLHKALDEAIPQD------------VDVPTHAMVKDIPYLQWVIWETMRIHSTSAMGLPREIPAGN-PP  398
CYP53A3__3425__Anid         348  DKLHKVLDEAIPQD------------VEVPTHAMVKEIPYLQWVIWETMRIHSTSAMGLPREIPEGN-PP  404
CYP53A__2301715__Mth        342  QKLQAELDAAIPAD------------MDVPTFDMVRDLPYLSAVVNETLRFHSTSGIGLPRQVPPDG-QG  398
CYP53A4__88466__Ndi         342  QKLQAELDANIPSE------------VDVPTYDMVKDLPYLEAIINEVLRFHSTSGIGLPRQIPCDAAQG  399
CYP53A4__7508__Ncr          341  QKLQAELDANIPPE------------VDVPTYDMVKELPYLEAVINEVLRFHSTSGIGLPRQIPHDASQG  398
CYP53A19__9543__Fve         346  EKLQAELDEAIPAD------------VSVPTYDMVRDLTYLNNVISETLRYHSTSGIGLPRQIPDNS-PG  402
CYP53A19__14206__Fox        346  EKLQAELDEAIPAD------------VSVPTYDMVRDLTYLNNVISETLRYHSTSGIGLPRQIPDNS-PG  402
CYP53A8__12085__Fgr         345  EKLQSELDNAIPSE------------VSVPTYDMVRDLPYLANVINETLRYHSTSGIGLPRQIPPNS-PG  401
CYP53A11__50496__Nhe        346  EKLQAELDASIPSH------------VSVPTFDMVRDLPYLNCVINETLRYHSTSGIGLPRQVPEGS-PG  402
CYP53A20__6367__Fve         340  QKLYEEISAVVPED------------VAIPDYESVKHLPYLGHCINETLRIHSPSGIGLPREIPPNH-KG  396
CYP53A20__10443__Fox        340  QKLYEEISAVVPED------------VAIPDYESVKHLPYLGYCINETLRIHSPSGIGLPREIPPNH-KG  396
CYP53A7__10227__Fgr         340  KKVYEEISAVMPDG------------VDIPDFESVKHLPYLGYCINETLRIHSPSGIGLPREVPPNH-KG  396
CYP53A10__35086__Nhe        341  QKLQQEIDEATADE------------GVIPSYESVKHLPYLGMCINETLRHHSPSGIGLPREIPAKS-KG  397
CYP53A5__120117__Mgrs       332  AKLQAELDAAIPDG------------VFAPAFDMIRNLPYLEAVINETLRIHSTSGIGLPRQIPADS-PG  388
CYP53A14__2107__Aor         356  WKLQNVLDEVIPVD------------IEVPYLAMVKKITYLQWVIWEALRIHSTFGQGLPREVPPER-GP  412
CYP53C__1025718__Fpi_       366  QKLQRELDEALGNDD-----------DPVSTFEQVKRLPYLEAVINEGLRLHSTSGIGLPRLVPEG---G  421
Cyp53C__68781__Pst          366  AKLHAELDEALGTDD-----------DPVAIFDQVKRLTYLQAVIDETLRIHSTSGIGLPRIVPAGS-GG  423
CYP53C__80617__Gtr          365  EKLQAELDEALGNDD-----------DPVSTFEQVKRLKYLEAVINEALRVHSTSGIGLPRVVPEG---G  420
CYP53C__127772__Cpu         388  TKLQRELDDALGTDD-----------DPVSTFDAVKRLPYLDSVINEALRLHSTSSIGLPRIAPEG---G  443
CYP53C__94174__Shi          370  EKLHVELDAALGNED-----------DPASTFEQTKNLKYLQAVIDESIRLHSTSGIGLPRIAPEG---G  425
CYP53c__27029__Wco          367  QKLQRELDEALGNDD-----------DPVAMYEQVKRLPYLEAVINEGLRLHSTSGIGLPRIVPEG---G  422
CYP53C3__110015__Ppl        366  QKLQRELDEALGSDD-----------DPVATYEQVKRLPYLEAVVNEALRVHSTSGIGLPRVVPEG---G  421
```

```
CYP53C__116910__Csu        364  EKLQAELDAALGDG------------DPVATFDQVKRLPYLEAVINEALRIHSTSGIGLPRIVPQG---G  418
CYP53C__1179842__Sla       371  EKLQKELDEALGDDD-----------HPVSTFEQVKRLPYLEAVINEALRVHSTSSIGLPRIVPEG---G  426
CYP53C__55106__Dsq         369  RKLQQELDAALGSDD-----------DPVASYEQVKRLPYLEAVINEALRIHATSGIGLPRLVPEG---G  424
CYP53C4__GL08839-P1.1__Gl  369  RKLQAELDGALGSASD--------DDS-IASFEDVKRLPYLEAVINEALRIHATSGIGLPRLVPEG---G  426
CYP53C4__47512__Gsp        369  RKLQAELDGALG--SN--------DDP-IASFEDVKRLPYLDAVINEALRIHATSGIGLPRLVPEG---G  424
CYP53C__129211__Tve        369  EKLQKELDDALGGED-----------DSVASYEQVKRLPYLDAVINEALRIHATSGIGLPRLVPAG---G  424
CYP53C9__27837__Pbr        367  KKLQAELDEVLGNDD-----------DPVSTYEEVKKLAYLQAVIDEALRIHSTSGIGLPRVVPEG---G  422
CYP53C7__118978__Bad       369  RRLQKELDDALGAHA----------DEPVVTFEDVKRLPYLQAVIDEALRIHSTSGVGLPRLVPEG---G  425
CYP53C__183109__Pca        365  RKLQQELDEVLGYDD-----------EPVSTYDQVKKLTYLPAVIDEALRVHSTSGVGLPRVVPEG---G  420
CYP53C2__130996__Pch       350  RKLQQELDEALGSDD-----------EPVSTFDQVKRLPYLQAVIDEALRIHSTSGIGLPRLVPKG---G  405
CYP53C__128292__Fme        378  KRLQQELDTALAGED-----------DPVATFQQVKSLPYLDAVINEVLRIHSTSGIGLPRLVPEG---G  433
Cyp53C__37267__Tve         367  EKLQQELDEALASEE-----------DEVALFERVKHLPYLEAVINEALRIHSTAGVGLPRVVPAG---G  422
CYP53C__70450__Ade         359  LKLQQELDAALPN-------------DGVTTYEQVKRLPYLTAVINEGLRLHSTSAMGLPRIVPEG---G  412
CYP53C__52716__Dsp         369  RKLQKELDEALGDD------------EDVPTYEQLKRLRYLDAVVNEGLRIHSTSSLGLPRIVPEG---G  423
CYP53C__83844__Cpu         369  EKLQKELDEALGSED-----------EPVTTYEQVKRLTYLEVVILEVLRLHSTIGLGLPRMAPEG---G  424
CYP53C__194303__Abi        370  DKLHQELDEQLGTED-----------ELVATAEQIKRLTYLEACINEALRIQSVSGIGLPRVVPEG---G  425
CYP53C__194181__Abi        374  EKLQKELDEHLGTEN-----------EFTATEAQVKNLPYLDACINEGLRLHSTSSVGLPREVPEG---G  429
CYP53C__143663__Fme        378  SKLQKEIDDALGGFD-----------DPMVTYAQIKHLQYLEAVINEGLRVHPTPGLGLPRVVPEG---G  433
CYP53C__130308__Fme        379  AKLQKELDEALASLD-----------DPVASYDLVKHLPYLDAVIHEGLRVHSTSGNGLPRLVPEG---G  434
CYP53C__24265__Fme         377  AKLQKELDEALVSFD-----------DPVTSYDLVNHLPYLDAVIHEGLRVHSTLGVGLPRLVPEG---G  432
CYP53C__149618__Fme        380  AKLQQELDVVFGGSY-----------DPVATYAQIKRLPYLEAVIIEGLRVHSTSGLGLPRTVPNG---G  435
CYP53C__94457__Fme         380  TKLQKELDNALGHSE-----------NHVATYSQIKQLSYLDAVVNEGLRVHSTVGIGLPREVPEG---G  435
CYP53C__154594__Fme        372  AKLQAELDDALGKDM----------EDPVVTYAQIKNLPYLEAVLNEGQRVYSTAALGLQRIVPEG---G  428
CYP53C__115179__Fme        380  AKLQKELDDALGAPSPS-------DDS-VSTYSQINHLPYLEAVINEALRVHPMVGLGLPRVVPAS---G  438
CYP53C1__5__Uma_           364  RKLQAELDRELEHA------------EEVPLHADVQELPYLQAVLSESLRYHSTSAIGLPRVIPAG---G  418
CYP53B2__32280__Sro        360  KKLQQELDEAFSGKG----------MSGVLEYEDVKALPYLGACINEALRRHSTSGIGLPRIMMD----D  415
CYP53B3__28617__Pgr        359  KRLMEELDEHLGTE------------EGVISYADCKELKYLNACINETLRIHSTSSIGLPRILPQ----T  412
CYP53C__48859__Fpi         381  AKLQAELDGALASVD-----------SEVAPYDTVKDLPYLDAVIHEGQRLYSTIGAGLPREVPAG---G  436
CYP53C__86809__Fpi         381  AKLQAELDGALASVD-----------SEVVPYDAVKDLPYLDAVIHEGQRLHSTVGAGLPREVPTG---G  436
CYP53C__138909__Wco        383  AKLQAELDEALKSID-----------SAVAPYDAVKNLPYLDAVINEGLRLHATIGAGLPRVVPEG---G  438
CYP53C__104840__Wco        385  TKLQAELDEALKSID-----------SAVAPYDAIKHLPYLDAVVNEGLRLHATVGAGLPRVVPEG---G  440
CYP53C__77097__Wco         389  AKLQAELDEALKAVD-----------SEVALYDAVKYLPYLDAVVNEGLRLHATIGAGLPRVVPEG---G  444
CYP53C__138864__Wco        383  AKLQAELDEALKSID-----------SAVAPYDAIQNLPYLDAVVNEGLRLYTTVGAGLPRVVPEG---G  438
CYP53C__104855__Wco        373  AKLQAELDEALKSVD-----------SVAAPYDAIKHLPYLDAVVNEGLRLHATIGAGLPRVVPEG---G  428
CYP53C__154264__Wco        340  AKLQTELDDALKAVD-----------SVVAPHGAIKHLPYLDAVVNEGLRLHSPVGAGLPRVVPEG---G  395
CYP53c__138853__Wco        383  AKLQAELDDALKAVD-----------SVVAPHDAIKHLPYLDAVVNEGLRLHSAVGAGLPRVVPEG---G  438
CYP53C__154237__Wco        284  AKLQAELDDALKADD-----------SIVAPHDAIKHLPYLDAVVNEGLRLDS----------PAP---G  329
CYP53D1__108845__Ppl       396  AKLQAELDAALEGID-----------SDVAPYDAVKDLPYLDAVINEGLRLHSTIGAGLPRVVPPG---G  451
CYP53D6__54877__Ppl        396  AKLQAELDAALDGID-----------SDVAPYDAVKDLPYLDAVINEGLRLHSTVGGGLPRVVPAG---G  451
CYP53D4__55859__Ppl        367  AKLQAELDTALDGID-----------SDVASYDAVKDLPYLGAVINEGLRLHATVGVGLPCVVPPG---G  422
CYP53D2v1__56013__Ppl      394  AKLQAELDVALDGVE-----------SDVAPYDAVKDLPYLDAVINEGLRLHSTIGAGLPRVVPSG---G  449
CYP53D2v2__48082__Ppl      394  AKLQAELDVALDGVE-----------SDVAPYDAVKDLPYLDAVINEGLRLHSTIGAGLPRVVPSG---G  449
CYP53D5__46728__Ppl        379  AKLQAELDAALDGTG-----------SEIAPYGAVKVLPYLEAVVNEGLRLHSGVGAGLPRVVPAG---G  434
CYP53D3__60352__Ppl        378  AKLQAELDAALDGIG-----------SEIAPYGTVKVLPYLEAVVNEGLRLHSGVGAGLPRVVPAG---G  433
CYP53C__112429__Csu        387  KKLQAELDAALADAG----------EGPIAPVEKTERLLYLNAVIDEGLRVHSTVGANLPRVVGPE---G  443
CYP53C__151209__Csu        384  ARLQAELDATLGISSTD-------LGSPVAPIDKIEHLPYLNAVIDEALRIHSTVAAGLPREVGPG---G  443
CYP53C__118598__Csu        401  RNLQSELDGALADVSSD-------EDSFVAPIDKIDNLPYLNAVVDEGLRVHSAVGANLPRTVGPE---G  460
CYP53H3__318972__Bad       367  AKLQVELDKALGPPCALD----GDNDPAIVSYDQVKNLSYLHDVVNEGLRLFSAVGLGLPRVVPES---G  429
CYP53H7__358536__Bad       372  AKLQKELDEAFGNPSAVAANDDDDGQPNLVKYERIKNLTHLQDVVNEGLRLFSTIGLGLPRVVPDG---G  438
CYP53H6__142452__Bad       370  ARLQKELDEALGEPVYGDE---DAERPVLVAYELIKNLPYLQDAVNEGLRLFSTIGLGLPRVVPES---G  433
CYP53H4__160054__Bad       370  ARLQAELDEALGCPVCES-----REEPVLATYEQIKGLSYLQDVVNEGLRVFSTVGLGLPRIVPDG---G  431
```

```
CYP53H2__55123__Bad        371  SKLQAELDGALGAPHAFG-----NDDVESVSYENIKNLQYLQDVVNEGLRLFSIAGFGLPRIVPEG---G  432
CYP53H5__65034__Bad        367  TKLQVELDEALGWPNAVD----DGNRPTIASYDSVKNLPYLQDVVNEGLRLFSAVGVGLPRVVPEG---G  429
CYP53C__101826__Pca        339  ERLQKALDDVLGVPNSMF-----STDEVVASFDLVKNLTYLQDVINEGLRLHSTVGVGLPREVPEG---G  400
CYP53C__256510__Pca        356  AKLQEELDDALGVPDASS-----NADNVVAPFDLVKNLAYLQDVINEGLRLHSTIGVGLPREVPEE---G  417
CYP53C__102576__Pca        368  ERLQKELDDVLGVPNSTF-----STDEVVAPFELVKNLTYLQDVINEGLRLHSTVGVGLPREVPEG---G  429
CYP53C__212559__Pca        367  AKLQAELDDVLGAPGSDS-----STDDVVAPFDRVKNLTYLQDVINEGLRVHSTLGAGLPREVPEG---G  428
CYP53D1__9015__Fox         338  QKLQAEVDSIDIPG-------------TVPPFTNVRALPYLDAVLNETMCLHSVLGIGLPREVLPGS-KG  393
CYP53A6__10234__Fgr        341  KKLQAELDEAIPAD------------VSIPSFEMVKNLKYLGFVVNEALRHHSTISLGLPRLVPENG-NG  397
Cyp53A__101387__Pst        343  AKLREELDEALSPG------------GLQGPTSDLNDLPYLNACIHEAIRLHSPTGMGLPRIVPEG---G  397
CYP53A__105834__Cpu        308  NKLIGILEEAVDEE--------------LPTADHVRDIPYLDAVINEGLRYHATTAIGLHRAVHEK---G  360
CYP53A__56813__Ade         334  KKLMAELDSINAPTAELDT---GTTIDGLPTHDQVKNLPYLNAVIEEGLRLFATNAFGLPRVSSRE---G  397
CYP53__1116154__Fpi        328  KPLKQEAEEKIKEH--------------GWTKTAMDAMWKTDSFFKESLRLNGVNHLSLFRKSMKD---V  380
CYP53NS__152212__Pca       324  RKLRDELDALLPAD------------CVVPSIEQVSRLPYLRLVIKETLRYNGP-GFGTFRYTPA----D  376
CYP53NS__92916__Fme        314  AKVRTELDEVLPPD------------SEIPTVEQASRLRYLHLVIKETLRYNGP-GFGTFRYTSK----D  366
Consensus_aa:                   .KLp.ELD.hl...............s.lsshp.lcpL.YLpsVlpEsLRl@thsthGLPR.hs.s...s
Consensus_ss:                   hhhhhhhhhh                     hhhhh  hhhhhhhhhhhhh   hh


Conservation:                       6  5  8  57 7 5  65    78 8   7 9598                          6 58
CYP53A__2107910__Tte       399  VHIAGHYFPPGTVLSVPTYSIHHSKEIWGPDADEFRPERWD----NLT------------ARQKNAFIPF  452
CYP53A15__ACF15219.1_Clu   388  VTILGRHFPQGTVLSVPAYTIHHSTEIWGPDADTFRPERWE----KVT------------EQQKAAFIPF  441
CYP53A23__71345__Mfi       393  VEVAGRHFPPGVVLSVPAYVMHHSKEIWGPDADEFRPERWE----KVT------------ERQKLAFIPF  446
CYP53A17__5031__Ure        398  VTIQGHVFHPGTILSVPAYTIHHSSEIWGPDVEEFVPTRWD----PARLT----------AQQKAAFIPF  453
CYP53A18__8044__Cim        398  VTIEGHVFHPGTILSVPAYTIHHSPEIWGPDVEEFVPTRWD----PARLT----------PRQKAAFIPF  453
CYP53A21__1341__Acl        400  VTISGHVFYPGDVVSVPSYTIHRSREIWGPDAEQFVPERWD----PARLT----------PRQKAAFIPF  455
CYP53A12__8190__Nfi        400  VTISGHTFYPGDVVSVPSYTIHRSKEIWGPDAEEFVPERWD----PARLT----------ARQKAAFIPF  455
CYP53A12__3003__Afu        400  VTISGHTFYPGDVVSVPSYTIHRSKEIWGPDAEKFVPERWD----PARLT----------ARQKAAFIPF  455
CYP53A22__2015__Ate        401  INISGHVFYPGDVVSVPSYTIHRSREIWGPDAEKFVPERWD----PARLT----------PRQKAAFIPF  456
CYP53A13__5958__Aor        400  VTISGHTFYPGDVVSVPTYTIHRSKEIWGPDAEQFVPERWD----PKRLT----------ARQKAAFIPF  455
CYP53A13__26719__Afl       400  VTISGHTFYPGDVVSVPTYTIHRSKEIWGPDAEQFVPERWD----PKRLT----------ARQKAAFIPF  455
CYP53A1__50153__Anig       399  VTISGHTFYPGDVVSVPSYTIHRSKEIWGPDAEQFVPERWD----PARLT----------PRQKAAFIPF  454
CYP53A3__3425__Anid        405  VEISGHIFKPGDILSVPTYTIHHSKEIWGADADEFIPERWA----PERLT----------ARQKAAFIPF  460
CYP53A__2301715__Mth       399  VHFGGHYFPPGTVLSVPTYSIHHSKEIWGPDADEFRPERWE----RLT------------PRQKNAFIPF  452
CYP53A4__88466__Ndi        400  VHIQGYYFPPGTVLSVPTYSIHHSKEIWGPDADEFKPERWE----RLT------------PRQKNAFIPF  453
CYP53A4__7508__Ncr         399  VHIQGYYLPPGTVLSVPTYSIHHSKEIWGPDADEFKPERWE----RLT------------ARQKNAFIPF  452
CYP53A19__9543__Fve        403  VTIKGHYFPPGSVLSVPTYTLHHSKEIWGPDADDFKPERWD----SLN------------ELQKTAFNPF  456
CYP53A19__14206__Fox       403  VTIKGHYFPPGSVLSVPTYTLHHSKEIWGSDADDFKPERWD----SVN------------NLQKTAFNPF  456
CYP53A8__12085__Fgr        402  VTIKDHFFPPGSILSVPTYTLHHSKEIWGADADDFRPERWE----NPT------------ELQKTAFNPF  455
CYP53A11__50496__Nhe       403  VTIRGHFFPAGSVLSVPTYSIHHSKEIWGPDADDFKPERWE----DVT------------PRQKNAFIPF  456
CYP53A20__6367__Fve        397  VTLHGRYFGPGTVLSVPTYTIHHSTEIWGPDADEFKPERWE----SLT------------DKQKNAFIPF  450
CYP53A20__10443__Fox       397  VTLHGRYFGPGTILSVPTYTIHHSTEIWGPDADEFKPERWE----NLT------------DKQKTAFIPF  450
CYP53A7__10227__Fgr        397  VTIHGRYFGPGTVLSVPTYTIHHSTEIWGPDADDFKPERWE----TLT------------DKQKSAFIPF  450
CYP53A10__35086__Nhe       398  VTLHGRYFGPGTVLSVPTYTVHHSTEIWGPDAEEFKPERWE----NIT------------DKQKIAFIPF  451
CYP53A5__120117__Mgrs      389  VTIRGQYYPPGTVLSVPTYTIHHSKEIWGPDADEFRPERWI----ENGGLT---------DRQKNAFIPF  445
CYP53A14__2107__Aor        413  VEICGHTFYPGDVLSVPGYTMHHSADIWGIDVEDFVPERWD----PRRLTQ----------RQKDSFIPF  468
CYP53C__1025718__Fpi_      422  LTVCGRFFPEGTVLSVPSYTIHRDQDVWGSDADAFRPERWF----EQDE-----------KAIQKTFNPF  476
Cyp53C__68781__Pst         424  MHVAGHFFPEGTVLSVPTYTIHRDKEVWGEDVEVFRPERFL----EGDQ-----------AVIQKTFNPF  478
CYP53C__80617__Gtr         421  LTVLGRTFPEGTIMSVPTYTIHRYEEVWGPDVDEFRPERWF----EIDQ-----------AQINKAFNPF  475
CYP53C__127772__Cpu        444  LALRGLWFPPGAILSVPSYTIHRDAGVWGADTEAFRPERWA----EEERRD----------AVQRAFNPF  499
CYP53C__94174__Shi         426  LTVCGKYFPEGTILSVPSYTIHRDVDVWGYDVEAFRPERWF----ERDA-----------EMIQKAYNPF  480
CYP53c__27029__Wco         423  LTVRGQFFPEGTVLSVPSYTIHRDREVWGADVDAFRPERWM----ELDK-----------NAVQKTFNPF  477
```

```
CYP53C3__110015__Ppl       422  LSVCGRFFPAGTVLSVPTYTVHRDAETWGADVDAFRPERWE----ERDK-----------NAVQKAFNPF  476
CYP53C__116910__Csu        419  LTAAGQYFPEGTVLSVPTYTVHRDKEAWGEDADLFRPERWF----EHDE-----------KTLQRAFNPF  473
CYP53C__1179842__Sla       427  LIVQGQHFPQGAVLSVPSYTIHRDTTVWGADPDQFRPERWF----ECDH-----------AAIQKTFNPF  481
CYP53C__55106__Dsq         425  LTVCGKFFPEGTVLSVPTYTIHRDKAVWGEDVDDFRPERWF----EQDK-----------NLVQKTFNPF  479
CYP53C4__GL08839-P1.1__Gl  427  LTVCGRFFPEGTVLSVPTYTIHRDREVWGEDVDAFRPERWF----ERDK-----------NLVQQAFNPF  481
CYP53C4__47512__Gsp        425  LTVCGRFFPEGTVLSVPTYTIHRDREVWGEDVDAFRPERWF----ERDK-----------NLIQKTFNPF  479
CYP53C__129211__Tve        425  LEVCGRWFPEGAVLSVPTYTIHRDKAVWGDDVEEFRPERWF----EQDK-----------VAVQKTFNPF  479
CYP53C9__27837__Pbr        423  LTVCGQFFPEGTVLSVPTYTIHRDTHVWGDDVETFRPERWF----EQDD-----------KLIQKTYNPF  477
CYP53C7__118978__Bad       426  LTVCGQYFQEGTVLSVPTYTIHRDKEIWGEDCEAFRPERWF----EQDK-----------NGIQKTFNPF  480
CYP53C__183109__Pca        421  MTVCGRTFPEGTILSVPTYTIHRDEEVWGKDVEVFRPERWF----SQDK-----------NEVQKTFNPF  475
CYP53C2__130996__Pch       406  MTVCGRFFPEGTVLSVPTYTIHRDEEVWGKDPEVFRPERWF----EQDK-----------NAVQKTYNPF  460
CYP53C__128292__Fme        434  LTVCGKTFPEGTVLSVPTYTIHRDKEVWGEDVEAMRPERWL----EGDQ-----------AAIQKTFNPF  488
Cyp53C__37267__Tve         423  LEVCGKFFPEGTVLSVPGYTIHRDKAVWGDDADEFRPERWF----GKDK-----------AALQKAFAPF  477
CYP53C__70450__Ade         413  LTVAGRFFTEGSILSVPSYTIHRDPEVWGEDFDKFRPERWS----EGDQ-----------TLIQKTFNPF  467
CYP53C__52716__Dsp         424  LEVSGIHFPAGSVLSVPSYTIHRDTAIWGPDPDIYRPERWF----EQDA-----------EGIQATFNAF  478
CYP53C__83844__Cpu         425  LTVHGTYFPEGTILSVPTYTLHRDKRVWGDDPEIFRPERWF----EENS-----------AKMHKAFNTF  479
CYP53C__194303__Abi        426  LEVLGNFFPEGTVLSVPSYSVHRDTKSWGDDTETYRPERWF----ERDQ-----------AAMNKAFNPY  480
CYP53C__194181__Abi        430  MMVCGQFFAEGTVLSVPSYTIHRDRGVWGEDFEAYRPERWF----ERDQ-----------TLMQKTFNPF  484
CYP53C__143663__Fme        434  LNVCGKWFPEGTILSVPTYTIHRNTGVWGEDANVFRPERWF----EGDQ-----------AAMQKVFNAF  488
CYP53C__130308__Fme        435  LTVCGKWFPAGTVLSAPTYTIHRDPKVWGEDADVFRPERWL----ERGQ-----------ATLLKAFNTF  489
CYP53C__24265__Fme         433  LTVCGKWFPEGTVLSAPTYTIHRDPKVWGEDADVFRPERWL----ERDH-----------ATLLKVFNTF  487
CYP53C__149618__Fme        436  LIVCGKWFSEGTVLSVPTYTIHRDPIVWGEDADAFRPDRWF----ERDQ-----------TILQKAFSPF  490
CYP53C__94457__Fme         436  ITVLGKSFPEGTVLSVPIYTIHRDPKVWGKDVDSFRPERWI----EGDK-----------AAMQKVFSPF  490
CYP53C__154594__Fme        429  LTISGKWFPEGTIVSVPTYTIHRDPKVWGEDVDVFRPERWL----EGDH-----------SAMSKTLNTF  483
CYP53C__115179__Fme        439  LTVCGKHFPEGTVLSVPTYTIHRDKEVWGEDADTFRPERWF----EGDK-----------STMQKVFNAY  493
CYP53C1__5__Uma_           419  ATVCGQQFPSGTILSVPAYTLHRDKSVFGADAEEYNPDRWL----APNAKR----------DFEKAFIPF  474
CYP53B2__32280__Sro        416  TEVLGEVFPKGTVLSVPSYTIHWSTEFWGPDAAEFKPERWL----ESEEKTR---------QLEKQLNVF  472
CYP53B3__28617__Pgr        413  VSFKGHILPKGLVCSVPTFEIHHDPDVWG-DPFTFRPERWL----EPNA-----------KDREKAFMPF  466
CYP53C__48859__Fpi         437  ATILGHHFKEGTTISVPIYRLHRDESTWGPDAAEFRPERWI----EASPERKK--------LMMDAFAPF  494
CYP53C__86809__Fpi         437  ATILGHHFREGITLSVPIYRLHRDESIWGPDAAEFRPERWI----EASPERKK--------LMMDAFAPF  494
CYP53C__138909__Wco        439  LTVLGHTFKEGTWVSVPVYHLHRDESIWGENASEFYPERWI----EASGDRKK--------AMLDAFAPF  496
CYP53C__104840__Wco        441  LTVLGHTFKEGTWVSVPVYHLHRDESIWGENANEFYPERWI----EASGDRKK--------AMLDAFAPF  498
CYP53C__77097__Wco         445  ITVLGHTFKEGTCVSVPIYYLHRDESIWGANATEFYPERWL----DATGERKK--------AMLDAFTPF  502
CYP53C__138864__Wco        439  LTVLGHTFKEGTWVSVPIYRLHQDESIWGENVNEFYPERWI----EASGDRKK--------AMLDAFAPF  496
CYP53C__104855__Wco        429  LTMLGHTFKEGTWVSVPVYHLHRDESIWGENASEFYPERWI----EASGERKK--------AMLDAFAPF  486
CYP53C__154264__Wco        396  MTVLGHTFKEGTWVSVPTYHLHRDENIWGENASEFYPERWI----EASGDQKK--------AMLDAFVPF  453
CYP53c__138853__Wco        439  MTVLGHTFKEGTWVSVPIYHLHRDENIWGENASVFYPERWI----EASGDQKK--------AMLDAFVPF  496
CYP53C__154237__Wco        330  SAYLR----------------------GENASEFYPERWV----EASGDQKK--------AMLDAFVPF  364
CYP53D1__108845__Ppl       452  LTVLGQHLKEGTVVSSPLYCLHRNEVVWGENAHEFYPERWL----EASADAKK--------EMMRSFAPF  509
CYP53D6__54877__Ppl        452  LTVLGQHLKEGTVVSSPIYTLHTNEVVWGENAHEFYPERWL----EASADAKK--------EMMRSFVPF  509
CYP53D4__55859__Ppl        423  LTVLGHHLKEGSVVSSPIYSLQRSEAVWGENAREFYPERWL----EASADAKK--------EMMRSFAPF  480
CYP53D2v1__56013__Ppl      450  MTVLGQHLKEGTVVSSPIYTLHRNEAVWGKNAYEFYPERWL----EASADAKK--------EMMQSFAPF  507
CYP53D2v2__48082__Ppl      450  MTVLGQHLKEGTVVSSPIYTLHRNEAVWGKNACEFYPERWL----EASADAKK--------EMMQSFAPF  507
CYP53D5__46728__Ppl        435  MTILGHHLMEGTVVSSPIYTLHRSKAVWGANADEFYPERWI----DASADTKK--------EMMSSFAPF  492
CYP53D3__60352__Ppl        434  MTILGHHLMEGTVVSSPIYTLHRSKAVWGANADEFYPERWI----DASADTKK--------EMMSSFAPF  491
CYP53C__112429__Csu        444  VTILGHTFTEGTVVSVPAYSTHRDENIWGHDAEFFRPERWL----EADKEKRD--------AMNKAFVPF  501
CYP53C__151209__Csu        444  LNVLGHFFPEGAVLSVPTYSAHRDESIWAPDPDAYRPERWI----EADKEKRE--------AMNKAFIPF  501
CYP53C__118598__Csu        461  ATVLGHSFQEGTILSVPAYSAHRDEQVWGLDCEEFRPERWL----EADREQQE--------LMKKAFIPF  518
CYP53H3__318972__Bad       430  LTVLGRTFAPGTVVSVPTHVLHHDKTIWGDDAESFNPDRWT----EGDK-----------TAMMEAFAPF  484
CYP53H7__358536__Bad       439  LTVLGNTLAPGTVVSVPTYIIHHDEEIWGNDAWSFNPNRWQ----TRDK-----------DTMSKAFAPF  493
CYP53H6__142452__Bad       434  LVVLGEAFTPGTVVSVPTYVTHHDEAIWGEDSWAFNPERWQ----TGDK-----------AVMAKAFAPF  488
```

```
CYP53H4__160054__Bad        432  LTVLGRTLSPGTVVSVPTYVLNRDKSIWGDDAEYFNPDRWA----NGNK-----------AAMSKAFAPF  486
CYP53H2__55123__Bad         433  LTILGHHFAPGAVISVPLYVVHRDKSVWGDDAEVFNPDRWA----AGDR-----------VAMTKAFAPF  487
CYP53H5__65034__Bad         430  LTILGRTLPPGTVVSVPAYVVHRDQAAWGDDVESFNPDRWA----KGDK-----------TGMMRAFAPF  484
CYP53C__101826__Pca         401  MTVAGKTLLAGTHVSCPTYTLHRLKSIWGDDADEFNPDRWT----RGDR-----------NMMLKYFAPF  455
CYP53C__256510__Pca         418  LTVAGKALLPGTHVSCPLYTLHRLKSIWGDDADEFNPDRWA----RGDR-----------KAMLKYFAPF  472
CYP53C__102576__Pca         430  MTVAGKALLPGTHVSCPTYTLHRLKSIWGDDADEFNPDRWT----RGDR-----------NMMLKYFAPF  484
CYP53C__212559__Pca         429  LTVAGKTLLAGTHVSCPSYTLHRLKSIWGDDADQFNPDRWT----LGDR-----------NAMLKYFSPF  483
CYP53D1__9015__Fox          394  VHLDSFYFPPGTVLSVPIYAIHRSRDIWGQDANNFRPERWE----KLS------------DRQKTSFIPF  447
CYP53A6__10234__Fgr         398  VTIAGYHFAPGTVLSIPIYTVHHLKEVWGPDADEFKPERWE----DVT------------QRQKQAFIPF  451
Cyp53A__101387__Pst         398  LTYRDYYFPPGTDVSVPTWTMSRDRAAWGEDADVFRPERWI----EDP-------------SLTKYFMAF  450
CYP53A__105834__Cpu         361  AMFGGKYFPPGTEMSVPAWTIQHDPEIWG-DPEVFRPERWI----ENP-------------DLKKYLMTF  412
CYP53A__56813__Ade          398  FELDGWTIPAGVEVSAPAYTIQRDPRIWGPDADDYRPERWI----DETD-----------SGLKKHMLTF  452
CYP53__1116154__Fpi         381  VLSNGTVIPAGTIVVATSTGTHLQEALY-KDAAEFRPFRFS----DVREKGGADAQKQQFHIPTAEYIAF  445
CYP53NS__152212__Pca        377  VEIEGVVLPANTTLALWNPQVHRCPNVWGADADTFRPERWM----STQGEGNE------KAALPGSYFPF  436
CYP53NS__92916__Fme         367  VEINGVTLPANTTLALWNPQVHRDPKLWGPDSDEFRPERWLVSTGSTSSEFSR------FIPPPGSYFPF  430
Consensus_aa:                    hpl.Gphh..GohlShPs@slH+s..lWG.Dhp.F.P-RW.....p.s..............b..s@.sF
Consensus_ss:                    eee  eee    eeeeehhhh   hhh    hh


Conservation:                    8 96769 86 69  66    65   57
CYP53A__2107910__Tte        453  SYGPRACVGRNVAEMEMKLIVATWARRYDVSLQQDH--------------------MDTRE-GFLRKPLG  501
CYP53A15__ACF15219.1_Clu    442  SYGPRACVGRNVAEMELALIVATVFRRYEFELRQGE--------------------METRE-GFLRKPLA  490
CYP53A23__71345__Mfi        447  SYGPRACVGRNVAEMELALIVATVFRRYEFELYQDE--------------------LETRE-GFLRKPLG  495
CYP53A17__5031__Ure         454  SHGPRACVGRNVAEMELHCIAATVFKNFEFQLEQNGP-------------------METSE-GFLRKPLG  503
CYP53A18__8044__Cim         454  SYGPRACVGRNVAEMELHCIAATVFKNFEFRLEQDGP-------------------METSE-GFLRKPLG  503
CYP53A21__1341__Acl         456  STGPRACVGRNVAEMELLVMTATVFRLFEFEMQQDGP-------------------METRE-GFLRKPLG  505
CYP53A12__8190__Nfi         456  STGPRACVGRNVAEMELLVMTGTIFRLFEFEMQQDGP-------------------METRE-GFLRKPLG  505
CYP53A12__3003__Afu         456  STGPRACVGRNVAEMELLVMTGTIFRLFEFEMQQDGP-------------------METRE-GFLRKPLG  505
CYP53A22__2015__Ate         457  STGPRACVGRNVAEMELLVMAGTVFRLFDFEMQQKGP-------------------METRE-GFLRKPLG  506
CYP53A13__5958__Aor         456  STGPRACVGRNVAEMELLVIVGTVFRLFDFEIQQDGP-------------------METRE-GFLRKPLG  505
CYP53A13__26719__Afl        456  STGPRACVGRNVAEMELLVIVGTVFRLFDFEIQQDGP-------------------METRE-GFLRKPLG  505
CYP53A1__50153__Anig        455  STGPRACVGRNVAEMELLVICGTVFRLFEFEMQQEGP-------------------METRE-GFLRKPLG  504
CYP53A3__3425__Anid         461  STGPRACVGRNVAEMELLVICSTVFRMFDWELQQKGP-------------------METRE-GFLRKPLG  510
CYP53A__2301715__Mth        453  SHGPRACVGRNVAEMEMKLIVATWARRYDVSLRQAH--------------------MDTRE-GFLRKPLG  501
CYP53A4__88466__Ndi         454  SHGPRSCVGRNVAEMEMKLNVATWARRYEVKLLQDY--------------------MDTSE-GFLRKPLG  502
CYP53A4__7508__Ncr          453  SHGPRSCVGRNVAEMEMKLIVATWARRYEVKLLQDY--------------------MDTRE-GFLRKPLG  501
CYP53A19__9543__Fve         457  SHGPRACVGRNVAEMEMKLIAATWARRYTPELKQEV--------------------METRE-GFLRKPLG  505
CYP53A19__14206__Fox        457  SHGPRACVGRNVAEMEMKLIAATWARRYTPELKQEV--------------------METRE-GFLRKPLG  505
CYP53A8__12085__Fgr         456  SHGPRACVGRNVAEMEMKLIAATWARRYVPELRQGV--------------------METRE-GFLRKPLG  504
CYP53A11__50496__Nhe        457  SHGPRACVGRNVAEMEMKLIAATWARRYNVELKQEI--------------------METRE-GFLRKPLG  505
CYP53A20__6367__Fve         451  SYGPRSCVGRNLAEMQMRLIAATWIKRYDVRLRQDI--------------------METRE-GFLRKPMG  499
CYP53A20__10443__Fox        451  SYGPRSCVGRNLAEMQMRLIATTWIKRYDVRLRQDI--------------------METRE-GFLRKPMG  499
CYP53A7__10227__Fgr         451  SYGPRSCVGRNLAEMQMRMIAATWIKRYDVVLRQDV--------------------METRE-GFLRKPMG  499
CYP53A10__35086__Nhe        452  SHGPRSCVGRNLAEMQMRLIAATWIKRYNIFLRQEK--------------------METRE-GFLRKPLG  500
CYP53A5__120117__Mgrs       446  SYGPRACVGRNVAEMEMKMIAATWARRYDVEVRQDV--------------------MEVRE-GFLRKPLA  494
CYP53A14__2107__Aor         469  SEGPRACIGRNLAEMELFVGCATLFRLFEFRVEGQGP-------------------LKVRE-RWLRKPVS  518
CYP53C__1025718__Fpi_       477  SFGPRSCVGRNLASMELLIILSSILRRYHFVLEHPEQG------------------LDTKE-GFLRKPVE  527
Cyp53C__68781__Pst          479  SFGPRACVGRNLANMELLIIIASILRRYHFVLEHPEKP------------------FDTRE-GFLRKPVE  529
CYP53C__80617__Gtr          476  SYGPRACVGRNLASMELMIIVSSIFRRYHFVLEEPEKK------------------FETRE-GFLRKPVE  526
CYP53C__127772__Cpu         500  SFGPRACVGRNLASMELLVIVSSILRRYTFVLEDAAKP------------------FDTRE-GFLRKPVE  550
CYP53C__94174__Shi          481  SFGPRACVGRNLASMELLIIISSILRRYDFVLEDPTKP------------------FATKE-GFLRKPVD  531
```

```
CYP53c__27029__Wco         478  SFGPRSCVGRNLASMELLIIIGSILRRYHFVLEDADKK------------------FDTRE-GFLRKPVE  528
CYP53C3__110015__Ppl       477  SFGPRSCVGRNLASMELLIIIASILRRYHFVLEEPKHK------------------LETKE-GFLRKPVA  527
CYP53C__116910__Csu        474  SFGPRSCVGRNLANLELLIIIASILHRYHFVLEDPEKH------------------FDTRE-GFLRKPVE  524
CYP53C__1179842__Sla       482  SFGPRACVGRNLASMELLIIISSILRRYHFVLADPEKP------------------FDTRE-GFLRKPQE  532
CYP53C__55106__Dsq         480  SFGPRSCVGRNLANLELLVIVASIFRRYHFVLENPNAQ------------------LETRE-GFLRKPVE  530
CYP53C4__GL08839-P1.1__Gl  482  SFGPRSCVGRNLANLELLVIVASIFRRYEFVLEDPTAE------------------LDTRE-GFLRKPVE  532
CYP53C4__47512__Gsp        480  SFGPRSCVGRNLANLELLVIVASIFRRYEFVLEDPAAE------------------LDTRE-GFLRKPVE  530
CYP53C__129211__Tve        480  SFGPRSCVGRNLANLELLVIVASIFRRYHFVLEDPSAP------------------LATNE-GFLRKPLK  530
CYP53C9__27837__Pbr        478  SYGPRSCVGRNLASMELLIIISSILRRYEFVLENPSKP------------------LETLE-GFLRKPVD  528
CYP53C7__118978__Bad       481  SFGPRSCVGRNLANMELLIIVASILHRYDFVLADPEKP------------------FDTAE-GFLRKPVD  531
CYP53C__183109__Pca        476  SFGPRSCVGRNLASMELLIIISSILRRYDFVLEEPDKP------------------FDTME-GFLRKPVE  526
CYP53C2__130996__Pch       461  SFGPRSCIGRNLANMELLIIVSSILRRYDFVLEDPDKP------------------FDTME-GFLRKPVE  511
CYP53C__128292__Fme        489  SFGPRACVGRNLASMELLIIIASIFRRYEFVLEKPDEQ------------------FDTRE-GFLRKPLR  539
Cyp53C__37267__Tve         478  SVGPRSCVGRNLAHLELLTFVASIFRRYSFVLENPDEP------------------LPTNE-GFLRKPLK  528
CYP53C__70450__Ade         468  SWGPRACVGRNLAMMELLLIVSTTFRRYHLVLESDDEA------------------SQTRE-GFLRKPVL  518
CYP53C__52716__Dsp         479  SFGPRACVGKNLASMELLLIVATIFHRYEFALLSQDQP------------------LETRE-GFLRKPVS  529
CYP53C__83844__Cpu         480  SFGPRACVGRNLANLELLIIVSSLLRRYDFVLKNPGDA------------------LGTCE-GFLRKPTD  530
CYP53C__194303__Abi        481  SVGPRSCVGRNLAAMELSIILASIMRRYEFVLKDEDKP------------------LVISE-GFLRKPLS  531
CYP53C__194181__Abi        485  SYGPRACVGRNLASMELLIILASIMRRYDIVLEDPDLI------------------LDTRE-GFLRKPLA  535
CYP53C__143663__Fme        489  SFGPRACIGRNLAMMELYIIIASIFHRYELILEEPNKP------------------LEIHE-AFMRKPVA  539
CYP53C__130308__Fme        490  SYGPRACIGRNVATMELFIIVSSIFRRYEFVLEEPHKP------------------LEVHE-GFIRKPMA  540
CYP53C__24265__Fme         488  SYGPRACIGRNVATMELFIFISSIFRRYDLVLEEQNKP------------------LEVHE-GFIRKPMA  538
CYP53C__149618__Fme        491  SFGPRACIGRELAIMELCIFVSSIFHRYDLELEAPDKP------------------LTIRE-DFIRKPVA  541
CYP53C__94457__Fme         491  SVGPRACTGRNLALMSLHIFIASIFRRYDIVLEQPDKP------------------LEVHD-AFARKPNS  541
CYP53C__154594__Fme        484  SIGPRACVGRNLALLELHIFIASIFYRYELVLEEPDKP------------------LETHE-GFIRKPLT  534
CYP53C__115179__Fme        494  SYGLRACAGRVLANVELQIFISSIFRRYEFVLEEPENP------------------VEVFE-GFIMRPKS  544
CYP53C1__5__Uma            475  SVGPRACVGRNVAMMELSILIAAIFRRYDIVLAEPDKP------------------LDTFE-GFLRKPVK  525
CYP53B2__32280__Sro        473  SFGPRSCVGRNVAMIELFCFMATLVYRYDFKLVDPNQK-----------------ELEVVE-GFLRKPTG  524
CYP53B3__28617__Pgr        467  SCGPRSCIGRNLAMMELYMITSTIFKRYEFALVDPDLA-----------------ELETRE-GFLRKV--  516
CYP53C__48859__Fpi         495  SVGPRACIGRSLAIMQLHIIVATLFHRYDFTLQSDEP-------------------LRVRD-SLARRPLE  544
CYP53C__86809__Fpi         495  SVGPRACIGRSLAIMQLHIIVATLLRRYDFALQSDEP-------------------LRVRD-SFARRPQE  544
CYP53C__138909__Wco        497  SMGPRSCIGRNLALMQLHIVLSTLFHRFDFVLESDDP-------------------LPVQD-SFVRKPKR  546
CYP53C__104840__Wco        499  SVGPRACIGRNLALMQLHIVLSTLFHRFDFVLESHDP-------------------LPVQD-SFVRKPKR  548
CYP53C__77097__Wco         503  SVGPRACIGRNLALMQLHIVIATLFRRFNVVLESDYS-------------------LPVQD-SFVRKPRW  552
CYP53C__138864__Wco        497  SMGPRSCIGRNLALMQLHIVLSTLFHRFDFVLESDDP-------------------LHVQD-SLIRKPKW  546
CYP53C__104855__Wco        487  SVGPRACIGRNLALMQLHKTLATLFYRFSFVLESSDPVRLRSCIRFCSTADSVRNQLPVQD-GFARKPRR  555
CYP53C__154264__Wco        454  SIGPRACIGRNLALMQLHIVLATLFHRFNVVLESDDP-------------------VRSQA-YALYVAGH  503
CYP53c__138853__Wco        497  SIGPRACIGRNLALMQLHIVLATLFRRFNVVLESDDP-------------------LPVQD-SFGRRPKQ  546
CYP53C__154237__Wco        365  SIGPRACIGCNLASMQLHTVLATLFHRFNVVLESDDP-------------------VRSQV---------  406
CYP53D1__108845__Ppl       510  SVGPRACLGRNLALQQMHIVLATIFHRYNLVLESDAP-------------------LPLRD-GFVRKPKN  559
CYP53D6__54877__Ppl        510  SAGPRACLGRNLALQQLHVMFATIFRRYSFALENDAQ-------------------LTIQE-SFVRKPKN  559
CYP53D4__55859__Ppl        481  SVGPRACLGRNLALQQLYIMFATLFRRYDFVLENDAP-------------------LAVQD-RLVQKSKE  530
CYP53D2v1__56013__Ppl      508  SVGPRACLGRSLALQQLHILLATIFHRYSLVLENNAPA-----------------QLPLRD-GFARKPMK  559
CYP53D2v2__48082__Ppl      508  SMGPRACLGRSLALQQLHILLATIFHRYSLVLENNAPA-----------------QLPLRD-GFARKPMK  559
CYP53D5__46728__Ppl        493  SIGLRACIGRNLAMQQLQIVTATIFRRYSIVLQDGAP-------------------LEIQD-SFGRKPKK  542
CYP53D3__60352__Ppl        492  SIGLRACIGRNLAMQQLQIVTATIFRRYSIVLQDDAP-------------------LEIQD-SFGRKPKK  541
CYP53C__112429__Csu        502  SVGPRACVGRNLAKMQLLVNIATVFRLYDIVLENPDLP------------------LPVHD-NFIRKPLN  552
CYP53C__151209__Csu        502  SVGPRACVGRNLAKIQLLINVATIFRCYDVVLEKPHDP------------------MPVHD-DFTRKVIE  552
CYP53C__118598__Csu        519  SVGPRACVGRNLAKMQLLINIATIFRLYKVVLADQDKE------------------LEVFD-NFVRKPLN  569
CYP53H3__318972__Bad       485  SVGPRACIGRNLALMELLIVVANVFYYFDAAPTSSDQP------------------MEIWD-GFLRQAFK  535
CYP53H7__358536__Bad       494  SLGPRACIGRNLAFMELLLVVANIFHRFEASSPYPGRE------------------AHIHD-GLPSKPLS  544
```

```
CYP53H6__142452__Bad       489  SVGPRACLGRNLALMELLLVVANVFHRFEVRLADPAQT------------------MEITD-GFLRKPVS  539
CYP53H4__160054__Bad       487  STGPRACIGRNFAFMELLLVIANVFHRYEVVPGQSEPT------------------MEVRE-GFLRKPLA  537
CYP53H2__55123__Bad        488  SIGPRACIGRNLAFMELLLVVANIFHRFEAKIVSAEQV------------------MEVRD-GFLRQPLS  538
CYP53H5__65034__Bad        485  SIGPRACIARNLASMEMLLFVANVFHRYEAKLASQLQ-------------------MDIHD-SFTRRPLA  534
CYP53C__101826__Pca        456  SIGPRACIGRNLAMMEMTICIATMFHRYRLVLASPDQQ------------------LECSE-GFVSKPKD  506
CYP53C__256510__Pca        473  STGPRACVGRNLATMEMTICIATIFHRYRVVLASPDQQ------------------LECHE-GLVRKPNS  523
CYP53C__102576__Pca        485  SIGPRACIGRNLAMMEMTICIATIVHRYRLVLANPDQQ------------------LECSE-GFVRKPKN  535
CYP53C__212559__Pca        484  SVGPRACIGRNLAMMELTICIATIFHRYRVVLASPDQR--------------------VSQ-LFNVLDKD  532
CYP53D1__9015__Fox         448  GHGPAACVGRNLAEVEMKIIAATLIKRYNFCMMDSE--------------------IESTE-GTTRKLIK  496
CYP53A6__10234__Fgr        452  SHGPRACLGRNLAEMELKVITATWARRYDLIMRDDT--------------------MEILE-GLARKPEA  500
Cyp53A__101387__Pst        451  SSGTRGCLGKSLAILELKMIVATLLQRYDITPQSLV--------------------LQTTE-KLMHKPTH  499
CYP53A__105834__Cpu        413  GKGPRACLGRHLAYMEMRLVLSTVLLRYDLQLKSQV--------------------METTE-GFMHKPNE  461
CYP53A__56813__Ade         453  GMGPRACIGKNLAYVQMQLALATSLLRYEFLLSPKAQ-------------------LRSIE-GFMHKPVE  502
CYP53__1116154__Fpi        446  GHGKHACSGRWFAAAEVKAILAYILLNYDFKLEKPGGRP---------------ENMNLGP-SILPHP-R  498
CYP53NS__152212__Pca       437  SYGPRKCMGEGLAMLEMSLTLATLFKRYDLELEPGFE-------------------MDFQPSFTLCSRNG  487
CYP53NS__92916__Fme        431  SYGPRKCLGEGLAILEMSLTLATLFKRYDLKLQEGFV-------------------MEFLPSFTLCSKNG  481
Consensus_aa:                   ShGPRtClGRslA.hph.lhhtolh+R@ch.L.ps.....................hph.p.shhp+P..
Consensus_ss:                            hhhhhhhhhhhhhhhhhheeeee                              eeee


Conservation:                     5    7
CYP53A__2107910__Tte       502  LKIALKRRQ------------  510
CYP53A15__ACF15219.1_Clu   491  LQVGMRKRSFA----------  501
CYP53A23__71345__Mfi       496  LQVGMRKRS------------  504
CYP53A17__5031__Ure        504  LLVGIKRRQLDPVVN------  518
CYP53A18__8044__Cim        504  LMVGIRRRQPNLN--------  516
CYP53A21__1341__Acl        506  LIVGMKRRAAHASV-------  519
CYP53A12__8190__Nfi        506  LIVGMKRRAVHASV-------  519
CYP53A12__3003__Afu        506  LIVGMKRRAVHASV-------  519
CYP53A22__2015__Ate        507  LIVGMKRRTPA----------  517
CYP53A13__5958__Aor        506  LMVGMKRRSVAV---------  517
CYP53A13__26719__Afl       506  LMVGMKRRSVAV---------  517
CYP53A1__50153__Anig       505  LQVGMKRRQPGSA--------  517
CYP53A3__3425__Anid        511  LTVGVKRRTIV----------  521
CYP53A__2301715__Mth       502  LEIALKRRKRD----------  512
CYP53A4__88466__Ndi        503  LKVGLKLRK------------  511
CYP53A4__7508__Ncr         502  LKVGLKLRK------------  510
CYP53A19__9543__Fve        506  LDIALKMR-------------  513
CYP53A19__14206__Fox       506  LDIALKMR-------------  513
CYP53A8__12085__Fgr        505  LDIALMMRE------------  513
CYP53A11__50496__Nhe       506  LDIALKIR-------------  513
CYP53A20__6367__Fve        500  LDVGLARR-------------  507
CYP53A20__10443__Fox       500  LDVGLARR-------------  507
CYP53A7__10227__Fgr        500  LDVGLARR-------------  507
CYP53A10__35086__Nhe       501  VEIGVSRR-------------  508
CYP53A5__120117__Mgrs      495  LEIGLKRRS------------  503
CYP53A14__2107__Aor        519  LQVGIRRRYLDARSS------  533
CYP53C__1025718__Fpi_      528  CRVGIKRRTV-----------  537
Cyp53C__68781__Pst         530  CKVGIKRRSA-----------  539
CYP53C__80617__Gtr         527  CRVGIKRRN------------  535
CYP53C__127772__Cpu        551  CRVGIRRRV------------  559
```

CYP53C__94174__Shi	532	CKIGIKRRNL-----------	541
CYP53c__27029__Wco	529	CKVGMKRRSL-----------	538
CYP53C3__110015__Ppl	528	CKVGLRRRSA-----------	537
CYP53C__116910__Csu	525	CRVGIKRRHD-----------	534
CYP53C__1179842__Sla	533	CRVGIKRRQT-----------	542
CYP53C__55106__Dsq	531	CKVGMKQRHT-----------	540
CYP53C4__GL08839-P1.1__Gl	533	CKVGMKRRNV-----------	542
CYP53C4__47512__Gsp	531	CKVGMKRRNA-----------	540
CYP53C__129211__Tve	531	CIVGMKRRNV-----------	540
CYP53C9__27837__Pbr	529	CVVGIRRRSL-----------	538
CYP53C7__118978__Bad	532	CQVGIKKRAN-----------	541
CYP53C__183109__Pca	527	CLVGIKRRSL-----------	536
CYP53C2__130996__Pch	512	CVVGIRRRTL-----------	521
CYP53C__128292__Fme	540	CRVGMRRREL-----------	549
Cyp53C__37267__Tve	529	CNVGMQRRNV-----------	538
CYP53C__70450__Ade	519	CRIGLKRRSPV----------	529
CYP53C__52716__Dsp	530	CYVGMKRRST-----------	539
CYP53C__83844__Cpu	531	CWVGLRRRAL-----------	540
CYP53C__194303__Abi	532	VDLGIKRRDV-----------	541
CYP53C__194181__Abi	536	CRVGIKRRDI-----------	545
CYP53C__143663__Fme	540	CHVGLKRRGA-----------	549
CYP53C__130308__Fme	541	CRVGMKRRNV-----------	550
CYP53C__24265__Fme	539	CRVGMKRRNV-----------	548
CYP53C__149618__Fme	542	CRVGMKRRNI-----------	551
CYP53C__94457__Fme	542	CRIGLKRRDV-----------	551
CYP53C__154594__Fme	535	CRVGMKRRDV-----------	544
CYP53C__115179__Fme	545	CRVGMKRRAI-----------	554
CYP53C1__5__Uma_	526	LEVGLKRRN------------	534
CYP53B2__32280__Sro	525	CQIGFKLRDATQ---------	536
CYP53B3__28617__Pgr	517	-GFSLQQKT------------	524
CYP53C__48859__Fpi	545	CMVGITRRKGELGYGSFEL--	563
CYP53C__86809__Fpi	545	CMVGITRRK------------	553
CYP53C__138909__Wco	547	CMIGIKSRKL-----------	556
CYP53C__104840__Wco	549	CVIGIKSRKL-----------	558
CYP53C__77097__Wco	553	CAIGIKLRKL-----------	562
CYP53C__138864__Wco	547	CMIGIKSRKL-----------	556
CYP53C__104855__Wco	556	CMIGIRSRKL-----------	565
CYP53C__154264__Wco	504	AAMGIEAVMLRQKEF------	518
CYP53c__138853__Wco	547	CMVGIKPRNL-----------	556
CYP53C__154237__Wco		---------------------	
CYP53D1__108845__Ppl	560	CVVGVQRRK------------	568
CYP53D6__54877__Ppl	560	CFVGVQRRK------------	568
CYP53D4__55859__Ppl	531	CIIGVQRRKGLVSRQ------	545
CYP53D2v1__56013__Ppl	560	CIVGVQRRK------------	568
CYP53D2v2__48082__Ppl	560	CIVGVQRRK------------	568
CYP53D5__46728__Ppl	543	CIIGVKRRDLKVL--------	555
CYP53D3__60352__Ppl	542	CIIGVKRRDLKVL--------	554
CYP53C__112429__Csu	553	CHVGVKRRS------------	561
CYP53C__151209__Csu	553	CFIGLKRREI-----------	562
CYP53C__118598__Csu	570	CRVGMQRRDLSSKREWSI---	587
CYP53H3__318972__Bad	536	CDVSLKSRGVA----------	546

```
CYP53H7__358536__Bad       545   SVIALRRRSGV----------  555
CYP53H6__142452__Bad       540   SVVALKRRVSA----------  550
CYP53H4__160054__Bad       538   STVAFKRRGAAL---------  549
CYP53H2__55123__Bad        539   SVVSVKRRGVF----------  549
CYP53H5__65034__Bad        535   SIVALKRRGL-----------  544
CYP53C__101826__Pca        507   VYVGMQRRV------------  515
CYP53C__256510__Pca        524   VPVGMRRRV------------  532
CYP53C__102576__Pca        536   VHVGMQRRL------------  544
CYP53C__212559__Pca        533   VG-------------------  534
CYP53D1__9015__Fox         497   VNIGIKRRQ------------  505
CYP53A6__10234__Fgr        501   VNVGIRRRM------------  509
Cyp53A__101387__Pst        500   DWVRLRRRNVD----------  510
CYP53A__105834__Cpu        462   MFVRLSRRERKGAAVQAQAQA  482
CYP53A__56813__Ade         503   LWVGIRRRHQPTA--------  515
CYP53__1116154__Fpi        499   AKVMFRKRKASRA--------  511
CYP53NS__152212__Pca       488   LPVYARLRK------------  496
CYP53NS__92916__Fme        482   LPVTARVRAQV----------  492
Consensus aa:                    h.lth+pR.............
Consensus_ss:                    eeeeeee
```

Fig. S1B

Colored PROMALS3D alignment (sequences in aligned order)

```
Conservation:                9                                         95  5      75 7 9 7  9 57  9
CYP53A__2107910__Tte      1  MA------------VINLILSPWA------PAALLV-AAVVYYVYPYLVTYRHLRHI-PAPFPAQFTNWW   50
23CYP53A__2301715__Mth    1  MA------------IVNFILSPWA------PVALLA-AAVVYYVYPYLVTYRHLRHI-PAPFPAQFTNWW   50
3CYP53A4__88466__Ndi      1  MA------------IISLLMSSWA------PVVLLA-GVAFYYLVPYFVTYSALRKI-PSPFPAQFTDLW   50
2CYP53A4__7508__Ncr       1  MA------------IISLLMSPWA------PVVLLA-GVAFYYLVPYFVTYSALRKI-PSPFPAQFTDLW   50
9CYP53A19__9543__Fve      1  MA------------VVDILFTWWS------IPIATG-VLIATYLYSYFVTYGHLRDI-PAPFPAQFTNLW   50
10CYP53A19__14206__Fox    1  MA------------VVDILFTWWS------IPIAAC-VLIATYLYSYFVTYGHLRDI-PAPFPAQFTNLW   50
6CYP53A8__12085__Fgr      1  MA------------IVDLLFTWWS------LPIAAG-LVAASYLYSYFITYGHLRDI-PAPFPAQFSNLW   50
8CYP53A11__50496__Nhe     1  MA------------IVDLLLSWWT------LPIGVA-VLVGTYLYAYFVTYGYLRGI-PAPFPAQFSNLW   50
CYP53A20__6367__Fve       1  MA------------LTELLISPWA------PLALAV-ALVAWYILPWI-SNSNLRGI-PAPFLAQFSNLW   49
11CYP53A20__10443__Fox    1  MA------------ITELLISPWA------PLALAV-ALVAWYILPWI-SNSNLRGI-PAPFPAQFTNLW   49
5CYP53A7__10227__Fgr      1  MA------------ITELLVSPWA------PVALVV-AFVAWYILPWV-SNKDLRGI-PAPFPAQFSNLW   49
7CYP53A10__35086__Nhe     1  MA------------LINLLISPWA------PVALVV-LFVGWYLVPYFGANRGLRGI-PAPFPAQFSNLW   50
1CYP53A5__120117__Mgrs    1  MA------------IVNLVFTPLG------LASLGAFMLVAYYVVPYFTTFGHLRSIQPASPLAGFSNLW   52
21CYP53A17__5031__Ure     1  M-------------LLAFLFNP---------YIIAGFTICYFYIVPYLQRW-DLRDI-PAPFPASLSHLW   46
20CYP53A18__8044__Cim     1  M-------------LLSFLFNP---------YVLAGIFIFVFYIVPYLRLS-YLRDI-PSPFAAGFSNLW   46
19CYP53A21__1341__Acl     1  M--------------ITEILTPQN-----TGYVLLG-LLTAYYIIPYLQTW-HLHDI-PSPGFAAFSNLW   48
12CYP53A12__8190__Nfi     1  M--------------ITDLLTPQN-----TGFILLG-LIAAYYIVPYLQKW-HLHDI-PSPSFAAFSNLW   48
13CYP53A12__3003__Afu     1  M--------------ITDLLSLQN-----AGLILLG-LIAVYYVIPYLQKW-HLHDI-PSPRFAAFSNLW   48
```

```
14CYP53A22__2015__Ate      1  M--------------IAD-LAAIN-----PAYLLLA-AVAAYYIVPYLKRW-HLRSI-PTPSVAGFTNLW   47
15CYP53A13__5958__Aor      1  M--------------IAELLTPTG-----AAYVLTA-AVIVYYILPYLQLW-RLRDI-PSPGFAAFSNLW   48
17CYP53A13__26719__Afl     1  M--------------IAELLTPTG-----AAYVLTA-AVFVYYILPYLQLW-RLRDI-PSPGFAAFSNLW   48
18CYP53A1__50153__Anig     1  M--------------LALLLSPYG------AYLGLA-LLVLYYLLPYLKRA-HLRDI-PAPGLAAFTNFW   47
CYP53A3__3425__Anid        1  M--------------ITDFLTPENITPERIALALLG-LLAAYYVVPYLQTW-RLSDI-PAPGLAAWTNFW   53
22CYP53A23__71345__Mfi     1  M-------------FLSFLFTPWA---------LLA-SPFLFYLLPFLRNW-SIRDV-PGPFLAKFTTLW   45
16CYP53A14__2107__Aor      1  MDGDSCPSCCSTCTSLNVFLCPHI------PILTLG-LLLVYYVTGY-LKRWHLRDI-PGPFIAGFSRIW   61
CYP53D1__9015__Fox         1  MS------------LDSLLLSPWA------PLAVLL-CVLLFYILPYFYTYRHLRGI-PGPLLARFSDLW   50
4CYP53A6__10234__Fgr       1  MA------------MFTVLPLIWL------APLGLI-SLFFYYIIPYFWNYRHLRSI-PGPLFARLSNWW   50
Consensus_aa:                 M.............hshlhs.hs.......s.hhh...lhh@YlhPYh.p..pLRsI.PtP..A.FoshW
Consensus_ss:                               hhhhhhhhhh      hhhhhh hhhhhhhhhh     hh          hhh  h


Conservation:                 7    9   9   7   9 5 9  79569 7595 5  79  7999979577   5597 755  57797
CYP53A__2107910__Tte      51  LLLVCRRGDRYATVDKVHKKLGPVVRIQPNHVSILDDEAIQAIYGHGNGFLK--SDFYDAFVSIQRGLFN  118
23CYP53A__2301715__Mth    51  LLLVCRRGNRYETVDKLHKKLGPVVRIQPNHVSLCDDAAIQVVYGHGNGFLK--SDFYDAFVSIQRGLFN  118
3CYP53A4__88466__Ndi      51  LLSVCRRGNRYQRVDELHKKLGPVVRIQPNHVSICDDAAIPTIYGHGNGFLK--SDFYDAFVSIRRGLFN  118
2CYP53A4__7508__Ncr       51  LLSVCRRGNRYQRVDELHKKLGPVVRIQPNHVSICDDAAIPTIYGHGNGFL---NDFYDAFVSIRRGLFN  117
9CYP53A19__9543__Fve      51  LLYVCRRGERYRVVDGIHKRLGPVVRIQPNHTSIADPDAIATIYGHGNGFLK--SDFYDAFVSIRRGLFN  118
10CYP53A19__14206__Fox    51  LLYVCRRGGRYRVVDEIHKRLGPVVRIQPNHTSIADPDAIATIYGHGNGFLK--SDFYDAFVSIRRGLFN  118
6CYP53A8__12085__Fgr      51  LLYVCRRGERYRVVDQIHKELGPVVRIQPNHTSIADAEAIATIYGHGNGFLK--SEFYDAFVSIRRGLFN  118
8CYP53A11__50496__Nhe     51  LLYVCRRGERYDVMDKIHKKMGPVVRIQPNHVSIADDEAIPIIYGHGNGFLK--SEFYDAFVSIRRGLFN  118
CYP53A20__6367__Fve       50  LLSTCRRGKRYEIVDQVHKKLGVLVRIAPNHVSVADADAINTIYGHGNGFLK--ADFYDTFVSIRRGLFN  117
11CYP53A20__10443__Fox    50  LLSTCRRGKRYEIVDQVHKKLGVLVRIAPNHVSVADADAINTIYGHGNGFLK--ADFYDTFVSIRRGLFN  117
5CYP53A7__10227__Fgr      50  LLSTCRRGKRYEIVDQVHKKLGPLVRIAPNHVSVADADAINTIYGHGNGFLK--ADFYDTFVSIRRGLFN  117
7CYP53A10__35086__Nhe     51  LMSTCRRGKRFEVVDQVHKRLGTVVRIAPNHVSIADADAINVIYGHGNGFLK--SDFYDPFVSIRRGLFN  118
1CYP53A5__120117__Mgrs    53  LLYTSRVGKRSLLVDEAHARLGPVLRVQPNHVSIADDEAINIIYGHGNGFLK--SSFYDAFVSIRRGLFN  120
21CYP53A17__5031__Ure     47  LLYQSRKGKRYQAVHNAHGKYGKLVRIQPNHVSVADADAIQTIYGHGNGFLK--SEYYDAFVSIRRGLFN  114
20CYP53A18__8044__Cim     47  LLYQCRRGKRYQAVHDAHKKYGKLVRIQPDHVSVADADAIQTIYGHGNGFLK--SEYYDAFVSIRRGLFN  114
19CYP53A21__1341__Acl     49  LLLQARQGHRFLKVDEAHKKHGKLVRIAPGHISIADDGAIQAVYGHGNGFLK--ADFYDAFVSIRRGLFN  116
12CYP53A12__8190__Nfi     49  LLLQARRGRRFLKVDEAHKKYGKLVRIAPRQVSIADDAAIQAIYGHGNGFLK--SDFYDAFVSIRRGLFN  116
13CYP53A12__3003__Afu     49  LLLQARRGRRFLKVDEAHKKYGKLVRIAPKHVSIADDAAIQAIYGHGNGFLK--ADFYDAFVSIRRGLFN  116
14CYP53A22__2015__Ate     48  LLIQARRGNRFEVVDNLHKKHGKLVRLAPRHVSIADDAAINAIYGHGNGFLKALSDFYDAFVSIRRGLFN  117
15CYP53A13__5958__Aor     49  LMLQYRKGNRFVTVDNAHKKYGKLVRIAPRHVSIADDEAIQAIYGHGNGFLK--ADFYDAFVSIRRGLFN  116
17CYP53A13__26719__Afl    49  LMLQYRKGNRFVTVDNAHKKYGKLVRIAPRHVSIADDEAIQAIYGHGNGFLK--ADFYDAFVSIRRGLFN  116
18CYP53A1__50153__Anig    48  LLLQTRRGHRFVVVDNAHKKYGKLVRIAPRHTSIADDGAIQAVYGHGNGFLK--SDFYDAFVSIHRGLFN  115
CYP53A3__3425__Anid       54  LLLQTRLGHRFISVDNAHKKYGKLVRIAPRHISIADDAAIQAVYGHGNGFLK--SDFYDAFVSIRRGLFN  121
22CYP53A23__71345__Mfi    46  YMYECRRCRRYYTVYKLHEKYGKFVRVQPNHVSIAEPEAIPIIYGHGTGFLK--SEYYDAFVSIQRGLFN  113
16CYP53A14__2107__Aor     62  LIVQVRQGYRSLVVHDLHRRYGKIVRLAPNHISIADESAIQAIYGHGNGFLE--TDFYNAFLNVDWSIFT  129
CYP53D1__9015__Fox        51  LLYICRQSKRSYTVYDLHERLGPVVRIQPNHVSIVDERAINLVYGHGNGLEK--SSWYDSSISLTRSIFT  118
4CYP53A6__10234__Fgr      51  LVYACREKSRWKYVNDAHTRYGPVVRIQPNHVSIANEEVINAIYGHGNGMLK--SSFYDASVITTYSIFT  118
Consensus_aa:                 Llh.tRptpR..hVpphHc+hG.lVRl.PsHlSItD..AI.hlYGHGNGhLK..ts@YDt.lSlpRtlFs
Consensus_ss:                 hhhhh    hhhhhhhhhhhh  eeee   eeee  hhhhhhhhh            hhhhh     eee


Conservation:                 59 979959997 779 96 59   595 5  5   6  57                  5  757 9577
CYP53A__2107910__Tte     119  TRDRAEHTRKRKIVSHTFSVKSVAQFEPYIHSNLELFVRQLDDLIARSTSP-DG----AAHLDCLHWFNY  183
23CYP53A__2301715__Mth   119  TRDRAEHTRKRKIVSHTFSVKSVAQFEPYIHSNLELFVRQLDNLIARSTNP-DG----AAHLDCLHWFNY  183
3CYP53A4__88466__Ndi     119  TRDRAEHTRKRKIVSHTFSAKSVQQFEPYMHSNLELFVKQWDSMIKNTKNP-DK----AAHLDCLEWFNY  183
2CYP53A4__7508__Ncr      118  TRDRAEHTRKRKIVSHTFSAKSVQQFEPYMHSNLELFVKQWDSMIKNSKNP-DK----AAHLDCLEWFNY  182
9CYP53A19__9543__Fve     119  TRDRAEHTRKRKLISHVFSAKSISQFEPYIHANLELFVKQLDKLVASGQTT-DKNGKRQALIDCLPWFNY  187
```

```
10CYP53A19__14206__Fox  119  TRDRAEHTRKRKLISHVFSAKSISQFEPYIHANLELFVKQLDKLVASGQTT-DKNGKRQALIDCLPWFNY  187
6CYP53A8__12085__Fgr    119  TRDRAEHTRKRKLISHVFSAKSISQFEPYIHANLELFVKQLDKLVASGQMAKNGK--REALMDCLPWFNY  186
8CYP53A11__50496__Nhe   119  TRDRAEHTRKRKLISHTFSTKSISQFEPYIHSNLELFVKQLDKLITSGTTK-DNQGHQQALIDCLPWFNY  187
CYP53A20__6367__Fve     118  TRDRAEHSRKRKIVSHTFAPKSVLEFEPYIRQNLDIFINQWDRI-ASNKEA-DG----YGSVDCLNWFNF  181
11CYP53A20__10443__Fox  118  TRNRAEHSRKRKLVSHTFAPKSVLEFEPYIRQNLDIFINQWDRI-ASNKDA-DG----YGSVDCLNWFNF  181
5CYP53A7__10227__Fgr    118  TRDRAEHSRKRKIVSHTFAPKSVLEFEPYIRQNLEIFVKQWDRI-SSNKER-DG----YGRVDCLNWFNF  181
7CYP53A10__35086__Nhe   119  TRSRAEHSRKRKIVSHTFAPKSVLEFEPYIHQNLDLFVKQWDRA-SSNPEA-DG----AGRLDCLSWFNY  182
1CYP53A5__120117__Mgrs  121  TRDRAEHTRKRKLISHTFAPKSVGQFEPYIHGNLELFAKKWDELIERTKKS-DG----WAPVECLQWF--  183
21CYP53A17__5031__Ure   115  TRNRAEHTRKRKTVSHTFSAKSIGQFEQYIHANLQLFLQQWTQICDLQRNPRSG----YASIDALNWFNY  180
20CYP53A18__8044__Cim   115  TRSRAEHTRKRKTVSHTFSAKSVGQFEQYIHANLQLFFQQWTNISEVQRNPKSG----YASIDALNWFNY  180
19CYP53A21__1341__Acl   117  TRDRAEHTRKRKTVSHTFSTKSIGQFEQYIHHNIELFVKQWTKLSKLNGNPRSG----YATIDALNWFNF  182
12CYP53A12__8190__Nfi   117  TRDREEHTRKRKTVSHTFSMKSIGQFEQYIHQNVELFVQQWTKLAKLNGNPRSG----YATIDALNWFNY  182
13CYP53A12__3003__Afu   117  TRDRAEHTRKRKTVSHTFSMKSIGQFEQYIHQNVELFVQQWTKLAKLNGNPRSG----YATIDALNWFNY  182
14CYP53A22__2015__Ate   118  TRDRAEHTRKRKTVSHTFSMKSIGQFEQYIHHNIELFVKQWTRLSETQGNPRSG----YATIDALNWFNF  183
15CYP53A13__5958__Aor   117  TRDRAEHTRKRKTVSHTFSMKSIGQFEQYIHGNAELFVKQWNRIADTQSNPKTG----YATIDALNWFNY  182
17CYP53A13__26719__Afl  117  TRDRAEHTRKRKTVSHTFSMKSIGQFEQYIHGNAELFVKQWNRIADTQSNPKTG----YATIDALNWFNY  182
18CYP53A1__50153__Anig  116  TRDRAEHTRKRKTVSHTFSMKSIGQFEQYIHGNIELFVKQWNRMADTQRNPKTG----FASLDALNWFNY  181
CYP53A3__3425__Anid     122  TRDRAEHTRKRKTVSHTFSAKSIGQFEQYIHHNIENLVKQLTRISNLQRNPKNG----YATVDALNWFNF  187
22CYP53A23__71345__Mfi  114  TRDRAEHTRKRKTVSHTFSAKSVGQFEQYIHHNLELLAKRWDEIAKNTGA--GK----YTRFDALHWFNY  177
16CYP53A14__2107__Aor   130  TRSRAEHTRKRKIVSHAFSARSLAQVEQHAHNNMEHLVRQWRKMIDSEEGLDDP----YAVIDARVWCNY  195
CYP53D1__9015__Fox      119  ARKRAEHARKRRYIAHSFAPKSSRAAEGPIADKVELLVRKWDEIIDKGPQF-DG----FTQLECRRWFTY  183
4CYP53A6__10234__Fgr    119  SRDRAEHSRKRKVVSHSFAPQSMRNFEPFIQQHLNVFLQKWDAMAANEAKF-DG----YADVESRVWLNY  183
Consensus_aa:                oRsRAEHoRKRKhlSHoFtsKSh.phE.@Ih.pl-lhlppWsphh.p..p..ss....ht.l-t..WFNY
Consensus_ss:                    hhhhhhhhhhhhhh hhhhhhhhhhhhhhhhhhhhhhhhhhh              eeehhhhhhh


Conservation:                7 7977799597 99979  5 5 7           55 55 575 975557759  9 779 7 5 99
CYP53A__2107910__Tte    184  LAFDVIGDLAFGAPFGMLSSGADMAEVRASPDSPPIYAPAIEILNRRGEVSATLGILPQLKPYAKYLPDP  253
23CYP53A__2301715__Mth  184  LAFDVIGDLAFGAPFGMLSSGADMAEVRASPESPPIYAPAIEILNRRGEVSATLGILPALKPYAKYFPDP  253
3CYP53A4__88466__Ndi    184  LAFDVIGDLSFGQPFGMLSSGADMAEIRSSPDAAPIYAPAIEILNRRGEVSATLGIHPALKPFAKYLPDP  253
2CYP53A4__7508__Ncr     183  LAFDVIGDLSFGQPFGMLSSGADMAEIRSSPDAAPIYAPAIEILNRRGEVSATLGIHPALKPFAKYLPDP  252
9CYP53A19__9543__Fve    188  LAFDVIGDLAFGVPFGMLANGADVAEVRATPDSAPIYASAIEILNRRGEVSATLGCWPQLKPYAQWLPDP  257
10CYP53A19__14206__Fox  188  LAFDVIGDLAFGVPFGMLANGADVAEVRETPDSAPIYASAIEILNRRGEVSATLGCWPQLKPYAQWLPDP  257
6CYP53A8__12085__Fgr    187  LAFDVIGDLAFGVPFGMLASGADVAEVRDTPDSPPIYASAIEILNRRGEVSATLGCFPQLKPYAQYLPDP  256
8CYP53A11__50496__Nhe   188  LAFDVIGDLAFGAPFGMLANGADVAEVRATPESPPIYASAIEILNRRGEVSATLGCFPQLKPYAKWLPDP  257
CYP53A20__6367__Fve     182  LAFDIIADLAFGKPFGMLSTGADIAEVKVSPTSPTIYAPAVEIMNRRGEVSATLGCLPQLKPYAKYLPDP  251
11CYP53A20__10443__Fox  182  LAFDIIADLAFGKPFGMLSTGADIAEVKASPTSPTIYAPAVEIMNRRGEVSATLGCLPQLKPYAKYFPDP  251
5CYP53A7__10227__Fgr    182  LAFDIIADLAFGKPFGMLASGADIAEVKASPTSPTIYAPAVEIMNRRGEVSATLGCLPQLKPYAKYLPDP  251
7CYP53A10__35086__Nhe   183  LAFDVIADLAFGKPFGMLATGADIAEVKASPTSPAIYAPAVEIMNRRGEVSATLGCMPWLKPYAKWLPDP  252
1CYP53A5__120117__Mgrs  184  ----------FGAPFGMLNAGADIAEVRMSVDSEPIYAPAVEILNRRGEVSATLGTLPELKPYAGYLPDS  243
21CYP53A17__5031__Ure   181  LAFDIIGDLAFGAPFGMLSKGRDVAEMKKSPNSPASYVPAIQVLNRRGEVSATLGCFPALKPFAKYLPDR  250
20CYP53A18__8044__Cim   181  LAFDIIGDLAFGAPFGMLSKGRDVAEMRKSPDSPASYVPAIQVLNRRGEVSATLGCFPALKPFAKYLPDK  250
19CYP53A21__1341__Acl   183  LAFDIIGDLAFGAPFGMLEKGKDFAEMRKTPDSPPTYVEAIEVLNRRGEVSAALGCFPRLIPYAKWIPDR  252
12CYP53A12__8190__Nfi   183  LAFDIIGDLAFGAPFGMLEKSKDIAEMRKAPDSDPTYVQAVEVLNRRGEVSATLGCLPRLIPYAKYLPDR  252
13CYP53A12__3003__Afu   183  LAFDIIGDLAFGAPFGMLEKGKDIAEMRKTPDSEPTYVQAVEVLNRRGEVSATLGCLPRLIPYAKYLPDR  252
14CYP53A22__2015__Ate   184  LAFDIIGDLAFGAPFGMLEKGQDIAEMRKSADAAPTYVQAVEVLNRRGEVSATLGTLPALIPYAKYIPDR  253
15CYP53A13__5958__Aor   183  LAFDIIGDLAFGAPFGMLEKGQDIAEMRKSPNDKPSYVQAVEVLNRRGEVSATLGACPSLIPWAKYIPDR  252
17CYP53A13__26719__Afl  183  LAFDIIGDLAFGAPFGMLEKGQDIAEMRKSPNDKPSYVQAVEVLNRRGEVSATLGACPSLIPWAKYIPDR  252
18CYP53A1__50153__Anig  182  LAFDIIGDLAFGAPFGMLDKGKDFAEMRKTPDSPPSYVQAVEVLNRRGEVSATLGCYPALKPFAKYLPDS  251
CYP53A3__3425__Anid     188  VAFDIIGDLAFGAPFGMLDKGQDIAEMRKSPDSPPQYVQAVEVLNRRGEVSATLGCYPALKPFAKYLPDR  257
22CYP53A23__71345__Mfi  178  VAFDIIGDLAFGAPFGMLEKGADIAEVQLNPDGPVTYAPAIEVLNRRGEVSNAVGCWPAIKPYAKYLPDP  247
```

```
16CYP53A14__2107__Aor   196  LTFDIIGDLAFGAPFGMLERENATVSMRKAPENPEVTLDAVEVLNHRGDVSAAFGICPDLIPYAKWLPDP  265
CYP53D1__9015__Fox      184  LSFDITGDLLFSEPFGMLENGSDLVKID---NKPRSYVSMMNSLAQRSAAVATLGVLPWLKPHAHHLPDP  250
4CYP53A6__10234__Fgr    184  LVLDIIGDLAFGAPFGVLAKGSEVVDFE--TEKGPSSLPVITSLSTRSEIAATVGALPELKPYLKWSPDP  251
Consensus_aa:                LtFDlIGDLAFG.PFGML.pG.Dhhchc.sspps..Yhshlp.LspRt-ltATlGhhP.LbP@A+@lPD.
Consensus_ss:                hhhhhhhhhh             hhhhhhhhhhhhhhhhhhhhhhhhhhhhhhhhhhhhhhhhhhh   h


Conservation:                99  9  7   95755 7 7  97           5 979 77  759  9 57   9755977955597
CYP53A__2107910__Tte    254  FFSKGLSAVEKLAGIAIARVKARL--ENPP--PASRKDLLQRLIDGRDEKGQPLGREELTAEALTQLIAG  319
23CYP53A__2301715__Mth  254  FFSRGLQAVENLAGIAIARVKARL--ENPP--PSHRKDLLQRLIEGRDEKGEPLGRQELTAEALTQLIAG  319
3CYP53A4__88466__Ndi    254  FFTKGLAAVENLAGIAIACVKSRL--ENPP--PVTRKDLLQRLMEGRDEKGEPLSREELTAEALTQLIAG  319
2CYP53A4__7508__Ncr     253  FFTKGLAAVENLAGIAIACVKSRL--DNPP--PVTRKDLLQRLMEGRDEKGEPLGREELTAEALTQLIAG  318
9CYP53A19__9543__Fve    258  FFSNGLNAVKNLAGIAIARVKARL--DNPP--SVERKDLLARLMEGRDEKGEPLGREELTAEALTQLIAG  323
10CYP53A19__14206__Fox  258  FFSNGLNAVKNLAGIAIARVKARL--DNPP--SVERKDLLARLMEGRDEKGEPLGREELTAEALTQLIAG  323
6CYP53A8__12085__Fgr    257  FFSNGLNAVKNLAGIAIARVKNRL--DNPP--SIERMDLLARLMEGRDEKGEPLGREELTAEALTQLIAG  322
8CYP53A11__50496__Nhe   258  FFSNGLNAVQNLAGIAIARVKARL--DNPP--PEERMDLLARLMEGRDEKGEPLGREELTAEALTQLIAG  323
CYP53A20__6367__Fve     252  FFSQGLQAVENLAGIAIARVSERL--ERGG--DSTRKDLLARLMQGRDEKGEPLGRDELTAEALTQLIAG  317
11CYP53A20__10443__Fox  252  FFSQGLQAVENLAGIAIARVSERL--ERGG--DSTRKDLLARLMQGRDEKGEPLGRDELTAEALTQLIAG  317
5CYP53A7__10227__Fgr    252  FFSQGLQAVENLAGIAIARVSERL--ERGG--DSTRKDLLARLMQGRDEKGEPLGRDELTAEALTQLIAG  317
7CYP53A10__35086__Nhe   253  FFSQGLQAVENLAGIAIARVSERL--ERGA--DTTRKDLLARLMQGRDEKGEPLGRDELTAEALTQLIAG  318
1CYP53A5__120117__Mgrs  244  FFSKGLAAVQNLAGIAIARVKSRL--ENPP--DVNRKDLLARLQEGRDAKGEPLGFEELTAEALTQLIAG  309
21CYP53A17__5031__Ure   251  FFRDGLEAVENLAGIAVARVAERLRPEVMA--KNTRVDLLSRLMEGRDETGAKLGREELTAEALTQLIAG  318
20CYP53A18__8044__Cim   251  FFRDGLEAVEHLAGIAVARVSERLRPEVMA--KNTRVDLLSRLMEGRDETGAKLGREELTAEALTQLIAG  318
19CYP53A21__1341__Acl   253  FFKDGLQAVENLAGIAVARVNERLKPEVMA--NNTRVDLLSRLMEGKDSNGNKLGREELTAEALTQLIAG  320
12CYP53A12__8190__Nfi   253  FFKDGIQAVENLAGIAVARVNERLKPEVME--KNTRVDLLSRLMEGKDSNGNKLGREELTAEALTQLIAG  320
13CYP53A12__3003__Afu   253  FFKDGVQAVENLAGIAVARVNERLKPEVME--KNTRVDLLSRLMEGKDSNGNKLGREELTAEALTQLIAG  320
14CYP53A22__2015__Ate   254  FFKDGIQAVENLAGIAIARVNERLRPEVMA--NNTRVDLLARLMEGKDANGNKLGREELTAEALTQLIAG  321
15CYP53A13__5958__Aor   253  FFRDGLEAVENLAGIAVARVNERLRPEVMA--NNTRVDLLARLMEGKDSNGNKLGREELTAEALTQLIAG  320
17CYP53A13__26719__Afl  253  FFRDGLEAVENLAGIAVARVNERLRPEVMA--NNTRVDLLARLMEGKDSNGNKLGREELTAEALTQLIAG  320
18CYP53A1__50153__Anig  252  FFRDGIQAVEDLAGIAVARVNERLRPEVMA--NNTRVDLLARLMEGKDSNGEKLGRAELTAEALTQLIAG  319
CYP53A3__3425__Anid     258  FFRDGLEAVENLAGIAIACVNERLKPEVMA--NNTRVDLLARLMEGKDANGNKLGRAELTAEALTQLIAG  325
22CYP53A23__71345__Mfi  248  FFSKGMEAIANLAGIATARVNQRLAAAERG--EIDRVDLLARLMEGKDENGNKLAKAELTAEALTQLIAG  315
16CYP53A14__2107__Aor   266  FFRQGAEAIANLAGVAGAAVDRRL--KMDTSMTEKRGDLLALLIDAEDQAGAKLGHRELTGEAVTLIAAG  333
CYP53D1__9015__Fox      251  FFHRGMDGLQNLLGVTSAHVKERM--AAGQ---DNHDDWLSLLLRARDDQGELLKFEEIASESLTLFMAA  315
4CYP53A6__10234__Fgr    252  FFRTGFNGMINLRTLGTSRITDRL--NNPP-GDEREKDLLERVREGRDHKGQPFGKGELIAEALTVLIAG  318
Consensus_aa:                FFppGhptlbNLhGlt.A+VppRL..p..s..s.p+.DLL..Lhct+DppGp.Lt+.ELhtEALTblhAG
Consensus_ss:                hhhhhhhhhhhhhhhhhhhhhhhh  hh          hhhhhhhhh        hhhhhhhhhhhhh


Conservation:                5795977 595775   59 7  77  557   5    59    76 7 99  55 9577 995 5 999
CYP53A__2107910__Tte    320  SDTTSNSSCALLFHAARTPGVLARLRAELDAAVPADLVVPTFDLVRDLPYLSAVVNETLRFHSTSGIGLP  389
23CYP53A__2301715__Mth  320  SDTTSNSSCALLYHAVRTPGVMQKLQAELDAAIPADMDVPTFDMVRDLPYLSAVVNETLRFHSTSGIGLP  389
3CYP53A4__88466__Ndi    320  SDTTSNSSCALLFHAVRTPGVMQKLQAELDANIPSEVDVPTYDMVKDLPYLEAIINEVLRFHSTSGIGLP  389
2CYP53A4__7508__Ncr     319  SDTTSNSSCALLFHAVRTPGVMQKLQAELDANIPPEVDVPTYDMVKELPYLEAVINEVLRFHSTSGIGLP  388
9CYP53A19__9543__Fve    324  SDTTSNSSCALLYHVTRTPGVLEKLQAELDEAIPADVSVPTYDMVRDLTYLNNVISETLRYHSTSGIGLP  393
10CYP53A19__14206__Fox  324  SDTTSNSSCALLYHVTRTPGVLEKLQAELDEAIPADVSVPTYDMVRDLTYLNNVISETLRYHSTSGIGLP  393
6CYP53A8__12085__Fgr    323  SDTTSNSSCALLYHVTRTPGVLEKLQSELDNAIPSEVSVPTYDMVRDLPYLANVINETLRYHSTSGIGLP  392
8CYP53A11__50496__Nhe   324  SDTTSNSSCALLYHVTRTPGVLEKLQAELDASIPSHVSVPTFDMVRDLPYLNCVINETLRYHSTSGIGLP  393
CYP53A20__6367__Fve     318  SDTTSNSSCALLYHVVRTPGVMQKLYEEISAVVPEDVAIPDYESVKHLPYLGHCINETLRIHSPSGIGLP  387
11CYP53A20__10443__Fox  318  SDTTSNSSCALLYHVVRTPGVMQKLYEEISAVVPEDVAIPDYESVKHLPYLGYCINETLRIHSPSGIGLP  387
5CYP53A7__10227__Fgr    318  SDTTSNSSCALLYHIVRTPGVMKKVYEEISAVMPDGVDIPDFESVKHLPYLGYCINETLRIHSPSGIGLP  387
```

```
7CYP53A10__35086__Nhe    319  SDTTSNSSCALLYHVVKTPGVLQKLQQEIDEATADEGVIPSYESVKHLPYLGMCINETLRHHSPSGIGLP  388
1CYP53A5__120117__Mgrs   310  SDTTSNSSCALLYWTARTPGVLAKLQAELDAAIPDGVFAPAFDMIRNLPYLEAVINETLRIHSTSGIGLP  379
21CYP53A17__5031__Ure    319  SDTTSNTSCAMLYWVLRTPGVIEKLQEVLDEAIPAHVEVPTFSMVKDIPYLQWVILETMRIHSTSSLGLP  388
20CYP53A18__8044__Cim    319  SDTTSNTSCAMLYWVLRTPGVIEKLQEALDEAVPAHVNVPSFSMVRDIPYLQWVIWETMRIHSTSSLGLP  388
19CYP53A21__1341__Acl    321  SDTTSNTACAILYWCMQTPGVITKLQKVLDEAIPADVDVPTHSMVKEIPYLQWVIWETMRIHSTSSMGLP  390
12CYP53A12__8190__Nfi    321  SDTTSNTTCAILYWCMSTPGVIPKLQKVLDEAIPDDVDVPTHAMVKDIPYLQWVIWETMRIHSTSAMGLP  390
13CYP53A12__3003__Afu    321  SDTTSNTTCAILYWCMSTPGVIPKLQKVLDEAIPDDVDVPTHAMVKDIPYLQWVIWETMRIHSTSAMGLP  390
14CYP53A22__2015__Ate    322  SDTTSNTACAILYWCMSTPGVIDKLHKVLDEAIPADVDVPTHSMVKDIPYLQWVIWETMRIHSTSAMGLP  391
15CYP53A13__5958__Aor    321  SDTTSNTSCAILYWCLRTPGVIEKLHKVLDESIPKDVDVPVHAMVKDIPYLQWVIWETMRIHSTSAMGLP  390
17CYP53A13__26719__Afl   321  SDTTSNTSCAILYWCLRTPGVIEKLHKVLDESIPKDVDVPVHAMVKDIPYLQWVIWETMRIHSTSAMGLP  390
18CYP53A1__50153__Anig   320  SDTTSNTSCAILYWCMRTPGVIEKLHKALDEAIPQDVDVPTHAMVKDIPYLQWVIWETMRIHSTSAMGLP  389
CYP53A3__3425__Anid      326  SDTTSNTSCAILYYCLRTPGVIDKLHKVLDEAIPQDVEVPTHAMVKEIPYLQWVIWETMRIHSTSAMGLP  395
22CYP53A23__71345__Mfi   316  SDTTSNTSCALLFHCLKNPHVVKKLQAELDEALPSD-DVPTYEQVKNLQYLDQVISETLRIHSTSSQGLP  384
16CYP53A14__2107__Aor    334  SDTSSSTLCALLYWVSSTPRVLWKLQNVLDEVIPVDIEVPYLAMVKKITYLQWVIWEALRIHSTFGQGLP  403
CYP53D1__9015__Fox       316  IETVSNTLSAMMYYLATSPSSLQKLQAEVDSIDIPG-TVPPFTNVRALPYLDAVLNETMCLHSVLGIGLP  384
4CYP53A6__10234__Fgr     319  TDTTSSTMAALLYHVVRTPGVLKKLQAELDEAIPADVSIPSFEMVKNLKYLGFVVNEALRHHSTISLGLP  388
Consensus_aa:                 oDToSNo.tAlLY@hhpTPtVl.KLp..lD.hlPsclslPs@..V+plsYL.hVl.EhhRhHSh.thGLP
Consensus_ss:                  hhhhhhhhhhhhhhhh hhhhhhhhhhhhhh        hhhhh  hhhhhhhhhhhhh


Conservation:                 9 67     77   5  6 79 77979 9  977 7799 975 9 9799 5    5  99 797997 9
CYP53A__2107910__Tte     390  REVPRD-GQGVHIAGHYFPPGTVLSVPTYSIHHSKEIWGPDADEFRPERW-D--NLTARQKNAFIPFSYG  455
23CYP53A__2301715__Mth   390  RQVPPD-GQGVHFGGHYFPPGTVLSVPTYSIHHSKEIWGPDADEFRPERW-E--RLTPRQKNAFIPFSHG  455
3CYP53A4__88466__Ndi     390  RQIPCDAAQGVHIQGYYFPPGTVLSVPTYSIHHSKEIWGPDADEFKPERW-E--RLTPRQKNAFIPFSHG  456
2CYP53A4__7508__Ncr      389  RQIPHDASQGVHIQGYYLPPGTVLSVPTYSIHHSKEIWGPDADEFKPERW-E--RLTARQKNAFIPFSHG  455
9CYP53A19__9543__Fve     394  RQIPDN-SPGVTIKGHYFPPGSVLSVPTYTLHHSKEIWGPDADDFKPERW-D--SLNELQKTAFNPFSHG  459
10CYP53A19__14206__Fox   394  RQIPDN-SPGVTIKGHYFPPGSVLSVPTYTLHHSKEIWGSDADDFKPERW-D--SVNNLQKTAFNPFSHG  459
6CYP53A8__12085__Fgr     393  RQIPPN-SPGVTIKDHFFPPGSILSVPTYTLHHSKEIWGADADDFRPERW-E--NPTELQKTAFNPFSHG  458
8CYP53A11__50496__Nhe    394  RQVPEG-SPGVTIRGHFFPAGSVLSVPTYSIHHSKEIWGPDADDFKPERW-E--DVTPRQKNAFIPFSHG  459
CYP53A20__6367__Fve      388  REIPPN-HKGVTLHGRYFGPGTVLSVPTYTIHHSTEIWGPDADEFKPERW-E--SLTDKQKNAFIPFSYG  453
11CYP53A20__10443__Fox   388  REIPPN-HKGVTLHGRYFGPGTILSVPTYTIHHSTEIWGPDADEFKPERW-E--NLTDKQKTAFIPFSYG  453
5CYP53A7__10227__Fgr     388  REVPPN-HKGVTIHGRYFGPGTVLSVPTYTIHHSTEIWGPDADDFKPERW-E--TLTDKQKSAFIPFSYG  453
7CYP53A10__35086__Nhe    389  REIPAK-SKGVTLHGRYFGPGTVLSVPTYTVHHSTEIWGPDAEEFKPERW-E--NITDKQKIAFIPFSHG  454
1CYP53A5__120117__Mgrs   380  RQIPAD-SPGVTIRGQYYPPGTVLSVPTYTIHHSKEIWGPDADEFRPERWIENGGLTDRQKNAFIPFSYG  448
21CYP53A17__5031__Ure    389  REIPQG-SPPVTIQGHVFHPGTILSVPAYTIHHSSEIWGPDVEEFVPTRW-DPARLTAQQKAAFIPFSHG  456
20CYP53A18__8044__Cim    389  REIPPN-SPPVTIEGHVFHPGTILSVPAYTIHHSPEIWGPDVEEFVPTRW-DPARLTPRQKAAFIPFSYG  456
19CYP53A21__1341__Acl    391  REIPPG-NPPVTISGHVFYPGDVVSVPSYTIHRSREIWGPDAEQFVPERW-DPARLTPRQKAAFIPFSTG  458
12CYP53A12__8190__Nfi    391  REIPPG-NPPVTISGHTFYPGDVVSVPSYTIHRSKEIWGPDAEEFVPERW-DPARLTARQKAAFIPFSTG  458
13CYP53A12__3003__Afu    391  REIPPG-NPPVTISGHTFYPGDVVSVPSYTIHRSKEIWGPDAEKFVPERW-DPARLTARQKAAFIPFSTG  458
14CYP53A22__2015__Ate    392  REIPAG-SPPINISGHVFYPGDVVSVPSYTIHRSREIWGPDAEKFVPERW-DPARLTPRQKAAFIPFSTG  459
15CYP53A13__5958__Aor    391  REIPAG-NPPVTISGHTFYPGDVVSVPTYTIHRSKEIWGPDAEQFVPERW-DPKRLTARQKAAFIPFSTG  458
17CYP53A13__26719__Afl   391  REIPAG-NPPVTISGHTFYPGDVVSVPTYTIHRSKEIWGPDAEQFVPERW-DPKRLTARQKAAFIPFSTG  458
18CYP53A1__50153__Anig   390  REIPAG-NPPVTISGHTFYPGDVVSVPSYTIHRSKEIWGPDAEQFVPERW-DPARLTPRQKAAFIPFSTG  457
CYP53A3__3425__Anid      396  REIPEG-NPPVEISGHIFKPGDILSVPTYTIHHSKEIWGADADEFIPERW-APERLTARQKAAFIPFSTG  463
22CYP53A23__71345__Mfi   385  RVVPPG--DGVEVAGRHFPPGVVLSVPAYVMHHSKEIWGPDADEFRPERW-E--KVTERQKLAFIPFSYG  449
16CYP53A14__2107__Aor    404  REVPPE-RGPVEICGHTFYPGDVLSVPGYTMHHSADIWGIDVEDFVPERW-DPRRLTQRQKDSFIPFSEG  471
CYP53D1__9015__Fox       385  REVLPG-SKGVHLDSFYFPPGTVLSVPIYAIHRSRDIWGQDANNFRPERW-E--KLSDRQKTSFIPFGHG  450
4CYP53A6__10234__Fgr     389  RLVPEN-GNGVTIAGYHFAPGTVLSIPIYTVHHLKEVWGPDADEFKPERW-E--DVTQRQKQAFIPFSHG  454
Consensus_aa:                 RplP.s.s.sVpl.G.hF.PGsVLSVP.YolH+Sp-IWG.DA-pF.PERW.-..plT.+QKstFIPFShG
Consensus_ss:                           eee  eee    eeeeehhhh   hhh    hh                    eee
```

```
Conservation:                  97795999699776     9 57 7     5   55  97 5997  7 67   9
CYP53A__2107910__Tte      456  PRACVGRNVAEMEMKLIVATWARRYDVSLQQD-HMDTREGFLRKPLGLKIALKRRQ------  510
23CYP53A__2301715__Mth    456  PRACVGRNVAEMEMKLIVATWARRYDVSLRQA-HMDTREGFLRKPLGLEIALKRRKRD----  512
3CYP53A4__88466__Ndi      457  PRSCVGRNVAEMEMKLNVATWARRYEVKLLQD-YMDTSEGFLRKPLGLKVGLKLRK------  511
2CYP53A4__7508__Ncr       456  PRSCVGRNVAEMEMKLIVATWARRYEVKLLQD-YMDTREGFLRKPLGLKVGLKLRK------  510
9CYP53A19__9543__Fve      460  PRACVGRNVAEMEMKLIAATWARRYTPELKQE-VMETREGFLRKPLGLDIALKMR-------  513
10CYP53A19__14206__Fox    460  PRACVGRNVAEMEMKLIAATWARRYTPELKQE-VMETREGFLRKPLGLDIALKMR-------  513
6CYP53A8__12085__Fgr      459  PRACVGRNVAEMEMKLIAATWARRYVPELRQG-VMETREGFLRKPLGLDIALMMRE------  513
8CYP53A11__50496__Nhe     460  PRACVGRNVAEMEMKLIAATWARRYNVELKQE-IMETREGFLRKPLGLDIALKIR-------  513
CYP53A20__6367__Fve       454  PRSCVGRNLAEMQMRLIAATWIKRYDVRLRQD-IMETREGFLRKPMGLDVGLARR-------  507
11CYP53A20__10443__Fox    454  PRSCVGRNLAEMQMRLIATTWIKRYDVRLRQD-IMETREGFLRKPMGLDVGLARR-------  507
5CYP53A7__10227__Fgr      454  PRSCVGRNLAEMQMRMIAATWIKRYDVVLRQD-VMETREGFLRKPMGLDVGLARR-------  507
7CYP53A10__35086__Nhe     455  PRSCVGRNLAEMQMRLIAATWIKRYNIFLRQE-KMETREGFLRKPLGVEIGVSRR-------  508
1CYP53A5__120117__Mgrs    449  PRACVGRNVAEMEMKMIAATWARRYDVEVRQD-VMEVREGFLRKPLALEIGLKRRS------  503
21CYP53A17__5031__Ure     457  PRACVGRNVAEMELHCIAATVFKNFEFQLEQNGPMETSEGFLRKPLGLLVGIKRRQLDPVVN  518
20CYP53A18__8044__Cim     457  PRACVGRNVAEMELHCIAATVFKNFEFRLEQDGPMETSEGFLRKPLGLMVGIRRRQPNLN--  516
19CYP53A21__1341__Acl     459  PRACVGRNVAEMELLVMTATVFRLFEFEMQQDGPMETREGFLRKPLGLIVGMKRRAAHASV-  519
12CYP53A12__8190__Nfi     459  PRACVGRNVAEMELLVMTGTIFRLFEFEMQQDGPMETREGFLRKPLGLIVGMKRRAVHASV-  519
13CYP53A12__3003__Afu     459  PRACVGRNVAEMELLVMTGTIFRLFEFEMQQDGPMETREGFLRKPLGLIVGMKRRAVHASV-  519
14CYP53A22__2015__Ate     460  PRACVGRNVAEMELLVMAGTVFRLFDFEMQQKGPMETREGFLRKPLGLIVGMKRRTPA----  517
15CYP53A13__5958__Aor     459  PRACVGRNVAEMELLVIVGTVFRLFDFEIQQDGPMETREGFLRKPLGLMVGMKRRSVAV---  517
17CYP53A13__26719__Afl    459  PRACVGRNVAEMELLVIVGTVFRLFDFEIQQDGPMETREGFLRKPLGLMVGMKRRSVAV---  517
18CYP53A1__50153__Anig    458  PRACVGRNVAEMELLVICGTVFRLFEFEMQQEGPMETREGFLRKPLGLQVGMKRRQPGSA--  517
CYP53A3__3425__Anid       464  PRACVGRNVAEMELLVICSTVFRMFDWELQQKGPMETREGFLRKPLGLTVGVKRRTIV----  521
22CYP53A23__71345__Mfi    450  PRACVGRNVAEMELALIVATVFRRYEFELYQD-ELETREGFLRKPLGLQVGMRKRS------  504
16CYP53A14__2107__Aor     472  PRACIGRNLAEMELFVGCATLFRLFEFRVEGQGPLKVRERWLRKPVSLQVGIRRRYLDARSS  533
CYP53D1__9015__Fox        451  PAACVGRNLAEVEMKIIAATLIKRYNFCMMDS-EIESTEGTTRKLIKVNIGIKRRQ------  505
4CYP53A6__10234__Fgr      455  PRACLGRNLAEMELKVITATWARRYDLIMRDD-TMEILEGLARKPEAVNVGIRRRM------  509
Consensus aa:                  PRACVGRNlAEMEh.lhhtThh+.@ph.h.pp..hEhpEGhhRKPltl.lGh++R.......
Consensus ss:                        hhhhhhhhhhhhhhhhhheeeee          eeee    eeeeeee
```

Fig. S1C

Colored PROMALS3D alignment (sequences in aligned order)

```
Conservation:                   9
CYP53A15__ACF15219.1_Clu     1  MF-------------------------------------LTSL-----------------LLTPY--TIL  14
CYP53C__1025718__Fpi_        1  MS-----------------------------SVVDQ---LTGLP--------------VAAWAGL---VV  21
Cyp53C__68781__Pst           1  MV---------------------------------------LSD--------------PLTLAGL---GL  14
CYP53C__80617__Gtr           1  --------------------------------MLSG---ILNAD--------------VSNILIL---LV  18
CYP53C__127772__Cpu          1  MDSVIATLKDLVPINLNFDFDAL----------LDR---LRSIS--------------PSQLAAG---VP  40
CYP53C__94174__Shi           1  ML-------------------ASL---------VNA---VTNVD--------------GKTCLVA---VP  22
```

```
CYP53c__27029__Wco           1  MS------------------------------GLLA---PLNLH--------------PAALLLL---IP   20
CYP53C3__110015__Ppl         1  MS------------------------------APAA---LFTSN--------------LVYGLAV---IP   20
CYP53C__116910__Csu          1  ME--------------------------------FL---HLSFD--------------WTTALLV---LA   18
CYP53C__1179842__Sla         1  MN-------------------TILQ-------LNPF--QDFHFS--------------FTTALAG---VP   25
CYP53C__55106__Dsq           1  MS------------------------------LVDR---LLNSE--------------PATWAVV---GF   20
CYP53C4__GL08839-P1.1__Gl    1  MS------------------------------LLDP---LLAFD--------------FATWAVI---GL   20
CYP53C4__47512__Gsp          1  MS------------------------------FLNP---LLALD--------------FATWAAL---GL   20
CYP53C__129211__Tve          1  MA------------------------------LLGI---LSSLD--------------GPSWAAL---VF   20
CYP53C9__27837__Pbr          1  MS-----------------------------SLLTS---FSLDN--------------VTNLLLV---IP   21
CYP53C7__118978__Bad         1  MA-------------------VLDY-------VLSP---LESLG--------------LASSLLI---IP   24
CYP53C__183109__Pca          1  MA------------------------------ILEV---LAQLN--------------LTSWLVL---IP   20
CYP53C2__130996__Pch         1  MA------------------------------VIEA---LTQLD--------------LKSWLLL---IP   20
CYP53C__128292__Fme          1  MG-------------------FTGV-------STLTN-ALARVD--------------LISLAVA---VP   26
Cyp53C__37267__Tve           1  MA------------------------------IFEL---LASLD--------------LPSWAAI---VL   20
CYP53C__70450__Ade           1  ME---------------------------------D---LLRLA---------------VYLALA---GS   16
CYP53C__52716__Dsp           1  MS-------------------SLLD-------YFPT--PFSSNA--------------LLLYGGL---AV   25
CYP53C__83844__Cpu           1  MD-------------------VQQL-------IQHL---PQNVD--------------LTSAAAA---SF   24
CYP53C__194303__Abi          1  MI-------------------IETF--------KNF---VAQAD--------------TTTLACA---IP   23
CYP53C__194181__Abi          1  MI-------------------LDRL-------SDTFLELYDKLD--------------LQIVAFA---VP   27
CYP53C__143663__Fme          1  MI----------------------------LIILRV---FVDIGY-------------RIWLFLP---WI   23
CYP53C__130308__Fme          1  MI----------------------------ASIFYS--TFFTKYL-------------WICAGLT---AG   24
CYP53C__24265__Fme           1  MI----------------------------LKILYS--VFFTNYL-------------WLCTGLT---AA   24
CYP53C__149618__Fme          1  MSM-----------------SSG---------IVQE---LLNVTF-------------WLWFFLL---AI   25
CYP53C__94457__Fme           1  MV----------------------------PNIAHN---FLLGYS-------------WILLLLP---AI   23
CYP53C__154594__Fme          1  ---------------------------------MQM---ISNISP-------------WYFCLLP---AA   18
CYP53C__115179__Fme          1  ML----------------------------PNILDA---LAQLN--------------TSQLCAS---AL   22
CYP53C1__5__Uma              1  MV-------------------ETDLV----PRIGAA---IQWSVE-------------SPAHVVI---TL   28
CYP53B2__32280__Sro          1  MT-------------------QSHV-------FTDF---FEPTTL-------------AVYFLAA---PL   25
CYP53B3__28617__Pgr          1  ML------------------------------VIGL---IAAFL---------------EYSILL---GL   19
CYP53C__101826__Pca             ----------------------------------------------------------------------
CYP53C__256510__Pca          1  ME------------------------------QVRS---LLPQLD-------------LKLASVL---LV   21
CYP53C__102576__Pca          1  ME------------------------------QSHF---ILPQLD-------------LKLITAI---LA   21
CYP53C__212559__Pca          1  ME------------------------------QTHF---LLPQLD-------------LRLTAGL---LA   21
CYP53C__48859__Fpi           1  MS------------------------------IVQQ---LTNFTR-----------GNPLLVLAA---LP   23
CYP53C__86809__Fpi           1  MS------------------------------IVQQ---LIDFTR-----------GNPLLVLAA---LP   23
CYP53C__138909__Wco          1  MS-------------------LLRD-------ITVQ---ALPFVD------------KLIFWLAL---IP   26
CYP53C__104840__Wco          1  MP-------------------LIGD-------ITVQ---TLPFVG------------ISTIWLALI-SIT   28
CYP53C__77097__Wco           1  MS-------------------LVDD-------TTAH---VPSY---------------PAFWIAL---IA   23
CYP53C__138864__Wco          1  MS-------------------LLRD-------ITVQ---ALSFVD------------KLIFWLAL---IP   26
CYP53C__104855__Wco          1  MS-------------------LLGD-------IAPQ---TLDYVG------------KLTFWLAS---IP   26
CYP53C__154264__Wco          1  MP-------------------LLAS-------ITAY---ALDYMC------------MPTFWLAL---IP   26
CYP53c__138853__Wco          1  MP-------------------LLDR-------ITTH---ALDYVC------------MPTFWLAL---IP   26
CYP53C__154237__Wco          1  M---------------------------------------------------------------------    1
CYP53D1__108845__Ppl         1  MD-------------------SSTPPLPPLGSIILAREGLTGLLL-----------PGLSVLACL---IA   37
CYP53D6__54877__Ppl          1  MD-------------------SSTPPLPLLGGIILTREGLTGLLL-----------PGLSVLACL---IA   37
CYP53D4__55859__Ppl          1  M---------------------------------------------------------LSVLVCL---IP   10
CYP53D2v1__56013__Ppl        1  MD-------------------SSTSLLPPLGSIILACEGLTSLVP-----------LILSVMVCL---IA   37
CYP53D2v2__48082__Ppl        1  MD-------------------SSTSLLPPLGSIILACEGLTSLVP-----------LILSVLVCL---VA   37
CYP53D5__46728__Ppl          1  ML-------------------TFDE-------ALN-------FST------------ILSALACL---SA   22
CYP53D3__60352__Ppl          1  ML-------------------TYDE-------ALN-------FST------------VLSALTCL---SA   22
CYP53C__112429__Csu          1  M-------------------------------ILDV---VHSVLER----------PL--LLVVL---LP   21
```

```
CYP53C__151209__Csu         1  ML--------------------PAE-------LVDC---LPGS---------------LPLLFGL---FL   22
CYP53C__118598__Csu         1  MD--------------------WRM-------MIDG---FTTLTSRETYTFSGFVSILLPLLFLC---LS   37
CYP53H3__318972__Bad        1  MD-------------------------------ENF---AIPLSS-------------GLKA------LV   17
CYP53H7__358536__Bad        1  MT------------------------------------LNVPLPT-------------AVWVMFLLSSLP   21
CYP53H6__142452__Bad        1  MA-------------------------------LSF---GAPDPT-------------TLAVISL---PL   20
CYP53H4__160054__Bad        1  MD-------------------------------PAV---VLILST-------------GLKAAVA---LL   20
CYP53H2__55123__Bad         1  MD-------------------------------AAW---GQIVTS-------------GVKAVLL---LI   20
CYP53H5__65034__Bad         1  MD-------------------------------------AIRDV--------------LLPFGTV---VA   16
Cyp53A__101387__Pst         1  MQ-------------------NSTL-------FSSA---THNLPE-------------AMQDYTW---LV   25
CYP53A__105834__Cpu            ----------------------------------------------------------------------
CYP53A__56813__Ade          1  MS--------------------------------------------------------SFWLCL----AA   10
CYP53__1116154__Fpi         1  MP----------------------------SLPLGL---KIPQ-----------------LSALE---IC   19
CYP53NS__152212__Pca        1  MS----------------------------GTIVNLGLDLREWLS-----------PARVVLCAS---AL   28
CYP53NS__92916__Fme         1  MS-----------------------------DIIAPFLPVKRFVA-----------SLPFAACIL---CL   27
Consensus_aa:                  M................................h.....h....................hhhh....h.
Consensus_ss:                                                  hhhh   hhhhh              hhhhhhh   hh

Conservation:                                           588  8    6  9               6   8   7
CYP53A15__ACF15219.1_Clu   15  LLPVLFYLLPYL---RNWRIRDI--PAPFPA--AWTNLWL--LYQCRRGRRFLAVHEAHQKLG-------   68
CYP53C__1025718__Fpi_      22  AAVVLVHLVPLIT--DPYQVRSY--PGPFLA--KISDAWL--GWVAAQGHRSEVVHELHQKHG-------   76
Cyp53C__68781__Pst         15  AAVVVAHIGAYLL--DPHNIRDI--PGPTLA--KFSDAWL--GWVAAKGHRSEVVHEMHAQYG-------   69
CYP53C__80617__Gtr         19  AVVAVAHIVPYFT--DPHAIKSY--PGPWLA--KFSDAWL--GKVSAQGHRSEVVHDLHKKYG-------   73
CYP53C__127772__Cpu        41  VCLLLYFLVPWLW--DPYHQRSI--PGPFLA--KFSNAWL--GWVSAHGHRSEIVHELHKKYG-------   95
CYP53C__94174__Shi         23  VLLVLAHVIPWLV--DVHGIRSY--PGPFWA--RFTDLWL--GWVAAQGHRSEVVHEMHNKYG-------   77
CYP53c__27029__Wco         21  AAVLAVHFVPYLL--DPHGIRSY--PGPFLA--KLSDAWL--GWVAAKGHRSEVVHQLHQRYG-------   75
CYP53C3__110015__Ppl       21  VAVLLVHFVPYLL--DPHGIRAY--PGPFLA--RLSDIWL--GWIAAQGHRSETVHELHKQYG-------   75
CYP53C__116910__Csu        19  VGVALVHVVPWLL--DPHGIRSY--PGPLLA--KFSDAWL--GWVAAQGHRSEVVHELHKKYG-------   73
CYP53C__1179842__Sla       26  IIFILVHVFPYLA--DPFKQRAI--PGPLLA--KFSDAWL--GWVSSQGHRSEVVHKMHLKYG-------   80
CYP53C__55106__Dsq         21  TAVLLIHFVPYLA--DPHHIREY--PGPLLA--KLSDIWL--GYVAAQGHRSERVHELHKQYG-------   75
CYP53C4__GL08839-P1.1__Gl  21  SAIVLFHLVPYLV--DSHHIRGY--PGPLLA--KFSDVWL--GYVAAQGHRSEQVHELHKQYG-------   75
CYP53C4__47512__Gsp        21  AAIVLFHLVPYLV--DSHHIRGY--PGPLLA--KFSDVWL--GYVAAQGHRSEKVHELHEQYG-------   75
CYP53C__129211__Tve        21  AAVVLVHVVPYLL--DPHGFRSY--PGPFLA--KLSDFWL--GKVAADGHRSERVHELHEIYGN------   76
CYP53C9__27837__Pbr        22  GLLVLGHVVFYLV--DPYKIRSY--PGPLLA--RFSDLWL--GRVAAEGHRSEIVHKLHQKYG-------   76
CYP53C7__118978__Bad       25  CLVLAGHFIYFVI--DPHRIRSY--PGPLLA--KLTDAWL--GYVAAHGHRSEVVHGLHQKYG-------   79
CYP53C__183109__Pca        21  ALAIAAHVVHWLL--DPHGIRSY--PGPLLA--RFSDAWL--GYVAAQGHRSEVVHDLHKKHG-------   75
CYP53C2__130996__Pch       21  ALAIVAHILIWLL--DPHGIRSY--PGPLLA--KFSDAWL--GYVAAQGHRSEVVHDLHKQYG-------   75
CYP53C__128292__Fme        27  LVLILGLIVPYFV--DPHCIRNNGITGPLSA--RFSDAWL--GWVAAQGHRSEVVHEMHKKYG-------   83
Cyp53C__37267__Tve         21  AAVVLVHLVPYVL--DPHGIRAY--PGPFWA--KLTDLWL--GKIAADGHRSERVHDLHKKYG-------   75
CYP53C__70450__Ade         17  IGTTLFVLVPYFK--DEHGVRDI--PGPLAA--HLSNLWL--AYWSSQGKRSEMVHEQHLKCG-------   71
CYP53C__52716__Dsp         26  LLLVASHLVPWVA--DPFGYRKKHIPGPFLA--QLSDVWL--ARVAAQGHRSEIIHGLHQKYG-------   82
CYP53C__83844__Cpu         25  AAFLAVHLGPYAW--DRYHLKSI--PGPFWA--KFSDAWL--AWVAANGHRSEEVHKLHEKLG-------   79
CYP53C__194303__Abi        24  AAVVLFHVIPWLT--DSHSLRKY--PGPFFA--KFSDFWL--AFTSRGGRRSEIIHDYHKKFG-------   78
CYP53C__194181__Abi        28  GTFLAFHLLPWLW--DPHGLRAY--PGPFIA--KFSDIWL--TCVSKGAHRSELVHEAHLKYG-------   82
CYP53C__143663__Fme        24  FLVVHLIPYLVDR--YHIRRNGI--TGPSLA--RFSDAWL--GWVVANGRQSEVVHEMHKKFG-------   78
CYP53C__130308__Fme        25  FLVVHVVPYLLDK--HHIRRNGI--TGPFLA--MFSDAWL--GWVAAQGRRAEVVHEKHKKYG-------   79
CYP53C__24265__Fme         25  FFLVHVVPYLFDK--HHIRRNDI--SGPLLA--RFSDAWL--GWVAAQGPRSEVVHQIHKKYG-------   79
CYP53C__149618__Fme        26  IFAVHLDAYFIDS--HRIRRNGI--SGPFLA--RFSGAWL--GWVVFQGRQSEVVHSLHKKFG-------   80
CYP53C__94457__Fme         24  ILVVHLVSYFVDS--KHIRRNDI--PGPTLA--KVSGSWL--GRVALEGRQSEVVHELHKKFG-------   78
CYP53C__154594__Fme        19  VLVLLVVPYFIDP--YWIRRNEV--RGPFLA--SLTSLWF--GWNATRGHLSQVVHDLHKKFG-------   73
CYP53C__115179__Fme        23  AVAAVVYLQPYLVDSHFIRRNGI--TGPFFA--RFSDAWL--GWVAAHGNRSVVVHKLHKKYG-------   79
```

```
CYP53C1__5__Uma_           29  LGAVVLFHVVPYI--TNTACIKY--PGPFFA--KFTDFWL--LRTALIGHRFEEVHKQHQKYG-------   83
CYP53B2__32280__Sro        26  GAVFLYLFVPHFT--SLAPLRRF--PGPFWA--GYTRLWL--ARTARVGKRSELVHREHLKHG-------   80
CYP53B3__28617__Pgr        20  VGISCYYLTGYLR--NKHQLNRY--PGPFLA--KFSRLWL--GYATRFGNRYQIIHQLHQKHG-------   74
CYP53C__101826__Pca         1  -------MIPFLV--DKYGLRGY--PGPLVA--KFSSLWL--ASKAHKGKTTSAVHALHQKYG-------   48
CYP53C__256510__Pca        22  AAPLFAWAVPFLV--DKHGLMAF--PGPLLA--KFSSLWF--ALKAYKGTTSLTVHALHERYG-------   76
CYP53C__102576__Pca        22  ASLLAAWIIPFLV--DKYRLRGY--PGPLLA--KFSGFWL--ASKAYKGTTTSAVYALHQKYE-------   76
CYP53C__212559__Pca        22  AALLAAWVVPFLI--DRYRLKGI--PGPLLA--KFSCVWL--ASKAYKGTMSSAVHVLHEKYG-------   76
CYP53C__48859__Fpi         24  VVLVVAKVVHYLV--DSSDLRSY--PGPFLA--KFTDAWI--FWTVSRNRWSRSVEDAHIKYG-------   78
CYP53C__86809__Fpi         24  VVLVVAKVVHYLA--DSSDLRSY--PGPFLA--KFTDAWI--FWTVSSNRWSHSVEDAHIKYG-------   78
CYP53C__138909__Wco        27  IISIAVYTVPYLL--DPLGLRAF--PGPILA--KFTIGWL--PWIVSQNRWSLTVNRLHQKYG-------   81
CYP53C__104840__Wco        29  GIVTVICVVPYLL--DSLGLRAF--PGPTLA--KFASGWL--PWVISQNRWSVTVGRLHEKYG-------   83
CYP53C__77097__Wco         24  ITVFAVQFVPYLL--DPLGLSSF--PGPVLA--KFSNVWL--PWIVSQNRWSVTVDQLHRKYGKTLIYCG   85
CYP53C__138864__Wco        27  IIGIAMYIVPYFL--DPLGLRAF--PGPILA--KFTIGWL--PWVVSQNRWSLTVNRLHQKYG-------   81
CYP53C__104855__Wco        27  IVIIAAHIVSYLL--DPLGLRAF--PGPIFA--RFTSGWL--PWIISQNRWSVTVDRLHQKYG-------   81
CYP53C__154264__Wco        27  AVLTTVYIVPYLL--DPLGLRTF--PGPIFA--KFTSGWL--PWVISQNRWSAIVDSLHRKDG-------   81
CYP53c__138853__Wco        27  AVLTTVYIVPYLL--DPLGLRTF--PGPIFA--KFTSGWL--PWVVSQNRWSVAVDSLHRKYG-------   81
CYP53C__154237__Wco         2  ------------------------------------------------------------YG-------    3
CYP53D1__108845__Ppl       38  TVIASSILLPYFN--DPYKLRAY--PGPFFA--KFTSAWL--SWIIGHNRWSETVYHLHRQHG-------   92
CYP53D6__54877__Ppl        38  TVIASLILIPYFN--DPYKLRAY--PGPFLA--KFTSAWI--SWTISHNPISEIVDHLHRQYG-------   92
CYP53D4__55859__Ppl        11  TVIASTFLIPYFS--DPYKLRAY--PGPFLA--KFTSVWA--SWVINHNRWSETVDLLHRKYG-------   65
CYP53D2v1__56013__Ppl      38  TVTISPTLLAYFN--DPFELRAY--PGPFLA--RFTSAWI--SWIISQNRWSETVDLMHRQHG-------   92
CYP53D2v2__48082__Ppl      38  TVTISPTLLAYFN--DPFELRAY--PGPFLA--KFTSAWI--SWIISQNRWSETVDLMHRQHG-------   92
CYP53D5__46728__Ppl        23  GVITLLLFLPYFI--DQLQLREY--PGALLA--KFTSGWI--SWIISRNQWSETVDRLHVQHG-------   77
CYP53D3__60352__Ppl        23  GVITLSLLLPYFI--DQLQLREY--PGALLA--KFTSGWI--SWIISRNQWSETVDRLHSAHSM------   78
CYP53C__112429__Csu        22  SVLLAVALVAHLR--DPHHLRSY--PGPFLA--SLTDLWL--AYKVWVGDRSPGVHELHKKHG-------   76
CYP53C__151209__Csu        23  TALLAVFFAPYIL--DRHRLRSY--PGPFLA--RFSDLWL--ASQVWKSHRSEEVHRLHKKYGK------   78
CYP53C__118598__Csu        38  ITLLLFPCVTYLL--DFYGLRSY--PGPFLA--KFTDLWL--AYKVWEGNRSPDIHLLHKKHG-------   92
CYP53H3__318972__Bad       18  FIVLLTHVVPFLR--DEHDVRSH--PGPALA--KLSDAWL--AWCAACGKINRSIYEAHKVYG-------   72
CYP53H7__358536__Bad       22  VLAILFHVVSYLK--DTLHLRRF--PGPPLA--RLSDVWL--AWHCGRGTINRAVLAAHRTYG-------   76
CYP53H6__142452__Bad       21  VFAILVHVLPYLA--DKLHLRRY--PGPPLA--RFSDVWL--AWHCARGSINRAVLEAHRTYG-------   75
CYP53H4__160054__Bad       21  AFAFLAHLVPFAL--DKFGVRQY--PGPLLA--KLSDIWL--GWHAAHGKINQAVWNAHRAYG-------   75
CYP53H2__55123__Bad        21  SFVLLVHLVPYAL--DRLSLSSH--PGPFLA--SLSDLWL--GWYAARGKMVRAVWDAHRVYG-------   75
CYP53H5__65034__Bad        17  ISTLLAYLLFSGF--HRSYLSSF--PGPLLA--RLSDAWI--GWHTARGTVNRAVYEAHKAYG-------   71
Cyp53A__101387__Pst        26  FLPAVVVGYIVAV--SRDPLQKV--PGPLLA--RWSNLWQ--AFYTRFGIRYKAIHAVHKTYG-------   80
CYP53A__105834__Cpu         1  --------------MKPDPLADV--PGPLLA--RWTPLWL--GYYARIGQRFTAVHKLHMEYG-------   43
CYP53A__56813__Ade         11  AALL----VLWRW--RADPLRNV--PGPWLA--RWTPFWL--MYHARRGERYLAVHDAHKKYG-------   61
CYP53__1116154__Fpi        20  GIVTALFLVSYLR-RRSDPIHAIPTVGPSWPLLSYLGAWR--YFRDAK----GMILEGCSKY--------   74
CYP53NS__152212__Pca       29  LIARVVLFVYAY----IKARRQF--PGPP-----VTNIWKGNLDETMTEDVHDKWRRWHRQYG-------   80
CYP53NS__92916__Fme        28  ALLKIVLFFIAY----FKARGQF--PGPP-----VSSLWSGNLSESMADDVHDKWRTWHRKYG-------   79
Consensus aa:                  hhhhhhhhh.hh...c...hp.h..PGP.hA..phoshWb.....h..sp.p..l.phHpp@G.......
Consensus ss:                  hhhhhhhhhhhh                          hh  hhhh    hhhhhhhhhhhh


Conservation:                      566       7  676      6   655       75  8  5         7   56  65
CYP53A15__ACF15219.1_Clu   69  --KLVRIQ------PNHVSIADA--DAITQVYGHG-NGFL-KSEYYDAFVSI---RRGLFNTRDRAEHTR  123
CYP53C__1025718__Fpi_      77  --KFVQIA------PNHVSVSDP--DALQVIYAHG-NGTL-KSTFYDAFVSI---QRGLFNTRSRPEHAR  131
Cyp53C__68781__Pst         70  --PVVRIA------PNHVSIAEP--QALQIVYAHG-NGSL-KSNFYDAFVSI---QRGLFNTRNRADHAR  124
CYP53C__80617__Gtr         74  --TFVRLA------PNHLSIADP--EALQTVYAHG-NGSL-KSDFYDAFVSI---RRGLFNTRDRAEHAR  128
CYP53C__127772__Cpu        96  --PVVRIA------PNHVSVADP--EALQVVYAHG-NGSL-KSDFYDAFVSI---HRGLFNTRDRQQHAR  150
CYP53C__94174__Shi         78  --PIVRIA------PNHISISDP--EALQIVYAHG-NGSL-KSNFYDAFVSI---HRGLFNTRDRAAHAR  132
CYP53c__27029__Wco         76  --TFVRIA------PNHVSISDP--DALSEVYAHG-NGTM-KSNFYDAFVSI---QRGLFNTRSRPEHAR  130
```

```
CYP53C3__110015__Ppl        76  --TFVRIA------PNHVSISDP--EAIQYVYAHG-NGTT-KSNFYDAFVSI---RRGLFNTRSRPEHAR  130
CYP53C__116910__Csu         74  --TFVRLA------PNHVSISDP--DAIQIVYAHG-NGSL-KSNFYDAFVSI---QRGLFNTRSRPEHAR  128
CYP53C__1179842__Sla        81  --TFVRIA------PNHVSVADP--DALQVVYAHG-NGSL-KANFYDAFVSI---QRGLFNTRNRNEHAR  135
CYP53C__55106__Dsq          76  --TFVRIA------PNHLSISDP--EALQVVYGHG-TGTL-KSDFYDAFVSI---QRGLFNTRSRVQHAR  130
CYP53C4__GL08839-P1.1__Gl   76  --TFVRIA------PNHLSISDP--DALQVVYGHG-TGTL-KSTFYDAFVSI---QRGLFNTRSRVQHAR  130
CYP53C4__47512__Gsp         76  --TFVRIA------PNHLSISDP--DALQIVYGHG-TGTL-KSTFYDAFVSI---QRGLFNTRSRVQHAR  130
CYP53C__129211__Tve         77  -WTFVRIA------PNHLSIADP--DALQIVYGHG-TGTL-KSDFYDAFVSI---QRGLFNTRSRTDHAR  132
CYP53C9__27837__Pbr         77  --TFVRLA------PNHVSVSDP--DALQVVYAHG-NGTL-KANFYDAFVSI---QRGLFNTRSRPEHAR  131
CYP53C7__118978__Bad        80  --KFVRIA------PNHVSIADP--DALPIVYGHG-NGTL-KSNFYDAFVSI---ERGLFNTRSRHEHAR  134
CYP53C__183109__Pca         76  --TFVRLA------PNHVSISDP--DALQVVYGHG-TGTL-KSDFYDAFVSI---QRGLFNTRSRPEHAR  130
CYP53C2__130996__Pch        76  --TFVRIA------PNHLSIADP--DALQVVYGHG-TGTL-KSNFYDAFVSI---QRGLFNTRSRSEHAR  130
CYP53C__128292__Fme         84  --TFVRLA------PNHVSISDP--AALQIVYAHG-NGTL-KSSFYDAFVSI---RRGLFNTRSRPEHTR  138
Cyp53C__37267__Tve          76  --PFVRIA------PNHLSISDP--DALPVVYGHG-TGTL-KSDFYDAFVSV---QRGLFSTRSRPEHTR  130
CYP53C__70450__Ade          72  --KLVRIA------PNHISVNDP--DALPIVYGHG-TGTL-KSEFYDAFVSI---QRGLFNTRSRTQHTR  126
CYP53C__52716__Dsp          83  --KVVRIA------PNHISLSEP--GALQIVYAHG-NGAL-KSDFYEAFVSI---RKNIFSTRDRAEHTR  137
CYP53C__83844__Cpu          80  --PVVRIA------PNHISIADP--DALQIIYAHG-SNTLKKSNFYDAFVSI---RRAIFNTREKADHAR  135
CYP53C__194303__Abi         79  --PVVRIA------PNHVSISDP--DALNAVYGHG-TGTL-KSEFYDAFVAM---DRGLFNVRDRHDHTR  133
CYP53C__194181__Abi         83  --PVVRIA------PNHLSIANP--EALQIVYAHG-NGAL-KSIFYDAFVSI---RRGLFNVRDRNEHTR  137
CYP53C__143663__Fme         79  --PVVRLA------PNHVSISDP--GALHVIYGHG-SGLL-KSGYYEPFTAV---RPSIFSTRSREVHSK  133
CYP53C__130308__Fme         80  --KFVRLA------PNHVSIADP--EAIGDIYGHG-NGTL-KTDFYDAFISI---GVTVFTTRSREEHTR  134
CYP53C__24265__Fme          80  --KFVRLA------PNHVSIAYP--EAIGEIYGHG-NGTL-KTDFYDAFLSI---DRTIFTTRSREEHTR  134
CYP53C__149618__Fme         81  --TFVRLS------PNHVSISDP--DALRLVYGRG-NGAL-KSDYYDAFLAV---RPSIFTTRSKEEHAR  135
CYP53C__94457__Fme          79  --TFVRLS------PNHVSISDP--EALQVVYGHG-NGML-KSEYYDAFAAP-NLRRSVFDTRSREEHAR  135
CYP53C__154594__Fme         74  --TFVRLS------PNHVSISDP--DALQTIYGHG-KGLM-KSDYYDAFKGL---RPSIFSTRDRAFHAW  128
CYP53C__115179__Fme         80  --LFVRLA------PNHVSISDP--EALHIVYGHG-SGTL-KSDYYDAFLAI---RHTVLTTRDREDHSM  134
CYP53C1__5__Uma             84  --KFVRIA------PNHVSIADH--EALQPIYGHG-TGTL-KPAYYDAFVPPRPFPRGLFNTRDRAEHTR  141
CYP53B2__32280__Sro         81  --KFVRIG------PNEVSIADP--AALPIVYAHG-SGSI-KADFYDAFVAS--PVRGLFNTRNRAEHTR  136
CYP53B3__28617__Pgr         75  --RFVRIA------PNELSIADP--DAVHIVLGHG-TGTT-KSKFYDAFVAI---HRGLFNTRDRADHTR  129
CYP53C__101826__Pca         49  --PFVRIS------SKHVSIADP--EALQAIYGHN-SGAL-KTDFYDAFVAF---RHNIFTSRSRLEHSR  103
CYP53C__256510__Pca         77  --PFVRLS------PQHVSIADP--EALRAIYGHS-SGTL-KTELYDAFVTF-------------FLAR  120
CYP53C__102576__Pca         77  -GPFVRIS------PKYVSIADP--EALQAIYGHT-SGTL-KTDFYDAFVTF---RRNIFTSRSRLEHSR  132
CYP53C__212559__Pca         77  --PFVRIS------PKYVSIADP--EALQAIYGYS-SGAL-KTELYDAFVFF---RPTMFSTRSRLEHSR  131
CYP53C__48859__Fpi          79  --PIVRIA------PNHISIDDP--KALAMVYGHS-TGFM-KSNWYDIFAAF--SVSNIFDTRSRSEHAR  134
CYP53C__86809__Fpi          79  --PIVRIA------PNHISIADP--KALATVYGHS-TGFT-KANWYNAFSEF--AAKNIFNTRSRSEHAR  134
CYP53C__138909__Wco         82  --IFVRLS------PNYVSIAHP--AALSAVYGHS-SGAT-KAPYYEVFGDF--RARNLFNILSRPEHAR  137
CYP53C__104840__Wco         84  --TFVRLA------PNHVSIAHP--AALSAVYGHS-SGAL-KAPFYDASGNF--KARNMFNTRSRSEHAR  139
CYP53C__77097__Wco          86  AGTFVRLA------PNHVSIAHP--AALPAVYGHS-SGTL-KAPLYDVFGPF--RARSIFSTRSRTEHAR  143
CYP53C__138864__Wco         82  --IFVRLS------PNHVSIAHP--AALSAVYGYS-SGVT-KAPYYDVFGDF--RAKNLFNIISRTEHTR  137
CYP53C__104855__Wco         82  --TFVRLS------PNHVSIAHP--AALPAVYGHG-SGAP-KAPYYDGFVNF--KSRNMFNTLSRSEHAR  137
CYP53C__154264__Wco         82  --IFVRLS------PNHISIAHP--VALPAVYGHS-SGAL-KAPFYDAFASF--KTRNMFNTLSRTEHTR  137
CYP53c__138853__Wco         82  --IFVRLS------PNYVSIAHP--VALPAVYGHS-SGAL-KAPFYDALSGF--KTRNMFNTLSRTEHAR  137
CYP53C__154237__Wco          4  --IFVCLS------PNHISIVHP--VALPAVYGHS-SGAL-KAPFYDAFASF--KTRNMFNTISRTGHTR   59
CYP53D1__108845__Ppl        93  --PIVRLG------PDNVSISDP--SALAAIYGHS-SGAL-KSTFYDAISSI--RIRNLFNTRDRAEHSR  148
CYP53D6__54877__Ppl         93  --PIVRLG------PNNVSIADPSGSAFSVIYSHS-SGVT-KSAFYDTFANF--RIRNIFNTRDRAEHSR  150
CYP53D4__55859__Ppl         66  --PIVRLG------PDSVSIADP--SAFAVIYGHS-SGAL-KAPFYDAFANH--RIRDLFNTRDRAEHSR  121
CYP53D2v1__56013__Ppl       93  --PIVRLS------PDHVSVASP--AAFAAVYGHS-SGAL-KAPFYNAFANF--KIRSIFNTRDRAEHSR  148
CYP53D2v2__48082__Ppl       93  --PIVRLS------PDHVSVASP--AAFAAVYGHS-SGAL-KAPFYNAFANF--KTRSIFNTRDRAEHSR  148
CYP53D5__46728__Ppl         78  --SFVRLA------PNHVSVSGP--SAFEAIYGHP-SSAA-KAPFYDIFSAG--GAANIFTTRDRAEHAR  133
CYP53D3__60352__Ppl         79  -GSFVRLA------PNHVSVSGP--SAFEAIYSHP-SSAL-KAPFYDIFSAG--GAANIFTTRDRAEHAR  135
CYP53C__112429__Csu         77  --TFLRIG------PNHISIASP--AALGVIYSHS-HPLL-KSDFYDGLATF--SAPGTFTVRDRVAHAR  132
CYP53C__151209__Csu         79  -CRFLRIG------PNHVSVADP--AAIPILYSHS-NPLM-KSDFYDGFTTF--RTPGIFVERDRVAHAR  135
```

```
CYP53C__118598__Csu          93  --PFMRIG------PNHISVVSP--AAISTIYSHI-DPLP-KSAFYDGLATF--SVPDIFTTRDRVTHGR  148
CYP53H3__318972__Bad         73  --PIVRIA------PNHISIADV--SALQPIYGHG-SGIL-KAESYDTFVAF--DTPSLFTTRSRDEHAR  128
CYP53H7__358536__Bad         77  --PVVRIA------PNHVSIADV--SALHLVYGHG-SGAL-KADFYDAFVGR--ATSSVFTTRSRHDHTR  132
CYP53H6__142452__Bad         76  --PVVRIS------PTQISVADV--SALQPIYGHG-SGAP-KAESYDAFSGL--GRPSIFTTRSREEHTR  131
CYP53H4__160054__Bad         76  --PIVRIA------PNHVSVADA--SALHQIYGHS-TGIL-KADLYDAFVSF--NRASIFSTRSREEHAR  131
CYP53H2__55123__Bad          76  --PVVRIA------PNHVSIADV--AALRTVYGHS-AGIL-KADLYDVFNPF--GRTTLFTTRSREEHAR  131
CYP53H5__65034__Bad          72  --PFVRIA------PNHISISHS--SALQPIYGHS-VGIL-KSEFYDIFTSF-NGTKSVFTTRSREEHAR  128
Cyp53A__101387__Pst          81  --PVVRIS------PNHVSIADM--SLLPSIYGQGMAAFN-KSPFYDAFLSE---KPSIFSTRDKQEHAQ  136
CYP53A__105834__Cpu          44  --PIVRIA------PNHISVADK--DALDLVYAQGSNAFD-KSTFYHAFVSD---KASVFSTTDRHDHAQ   99
CYP53A__56813__Ade           62  --PAVRIA------PWHVSFASP--DAPARVYAQGSAALD-KSPFYRAFYVQ--GAESLFSTQNRALHAA  118
CYP53__1116154__Fpi          75  --EVFKIPL---SDQWLVVVSGR--DMNDELRKYP-DDTM-SALEAQKWVVQ-----TEYTLGNNNPDAT  130
CYP53NS__152212__Pca         81  --PIYQTVRWNGLFSRVIYVGDP--RLIRKIANEN--WPK-FPAQYAGFRPL--SGSALFAQMDQARWKT  141
CYP53NS__92916__Fme          80  --PVFQTWNG--LFSRVVYVGDP--RIISKIGNSN--WPK-FHAQYSGFKPL--SGSALFAQMDQERWKQ  138
Consensus_aa:                    ...hl+lt......PphlSltcs...Al..lYt@s.ssh..Kt.hYcsF.sb.....slFsh.s+..Hsp
Consensus_ss:                       eeee         eeee  h  hhhhhhhh             hhhh        eee    hhhhh


Conservation:                    676  7  75 5 5   6             55                        7         6
CYP53A15__ACF15219.1_Clu    124  KRKTVAHTFSAKSVLQFEQYIHHNLQELQNQWDRRAESVKG-------------------GWYEMDALN  173
CYP53C__1025718__Fpi_       132  KRKIVSHIFSQKSVLEFEPYTRMHIKKLMNQWDRLYDLAMKGGSGEEGE-G-----WQGRDGRLWLDILP  195
Cyp53C__68781__Pst          125  KRKIVSAIFSMKNVLEFEPHVREYVGLLIKQWDRLCAEAVKGGSGDEGEGG-----WRGESGRLWLDCLP  189
CYP53C__80617__Gtr          129  KRKIVSHIFSQKSVLEFEPHVRLYVRQFIEQWDRLCGLAAKGERGEEGNG------WEGREGRLWLDCLP  192
CYP53C__127772__Cpu         151  KRKIVSGIFSQKNVLEFEPHVRLYVGQLMEQWDRLCARAEKGESGDEGEGG-----WQGRGGKLWLDCLP  215
CYP53C__94174__Shi          133  KRKIVSHIFSQKNVLEFEPHVREYVKSLIAQWDRLYDLAVNGESGTEGEGG-----WVGREGRLWLDCLP  197
CYP53c__27029__Wco          131  KRKIVSHIFSQKSVLEFEPYTRQHVGALFKQWDRMCELGTKGLFGEEGEGG-----WHGRDGRVWFDCLP  195
CYP53C3__110015__Ppl        131  KRKIVSHIFSMKSVMEFEPYTRMHVAQLLKQWDRLYELGIKGASGEEGEG------WKGRDGRVWLDCLP  194
CYP53C__116910__Csu         129  KRKIVSHIFSQKNVLEFEPHVREHIRTLISQWDRLYELGKKGLSGTEGEGG-----WQGKNGRVWLDCLP  193
CYP53C__1179842__Sla        136  KRKIVSHIFSQKNVLEFEPNVRLYVGQLISQWDRLYDSAAKGASGTEGEGG-----WFGKDGRLWLDSLP  200
CYP53C__55106__Dsq          131  KRKIVSNIFAQKNVLDFEPHVRQHLANLFRQWDKLCEGGKNGLSGDEGEGG-----WQGRDGRVWYDCLP  195
CYP53C4__GL08839-P1.1__Gl   131  KRKIVSNIFAQKNVLEFEPHVREHLGTLFQQWDKLCDGGKKGLSGTEGEGG-----WHGGEGRVWYDCLP  195
CYP53C4__47512__Gsp         131  KRKIVSNIFAQKNVLEFEPHVRQHLGTLFQQWDKLCDGGKKGLSGTEGEGG-----WHGSDGRVWYDCLP  195
CYP53C__129211__Tve         133  KRKIVSHIFSQKNVLEFEPHVRVHLIQLFKQWDRLCAGGARGEAGDEGEGG-----WRGRDGRVWYDCLP  197
CYP53C9__27837__Pbr         132  KRKIVSHIFSQKSVLEFEPNTRLYVRQLIAQWDRLCELGAKGLSGDEGEGG-----WKGRNGRVWLDCLP  196
CYP53C7__118978__Bad        135  KRKIVSHIFSQKSVLEFEPNVRTYVGQLIGKWDRLYENGAKGLSGDEGEGG-----WTGRNGRVWLDCLP  199
CYP53C__183109__Pca         131  KRKIVSHIFSQKSVLEFEPHVRLYVNQLIRQWDRLYEAGAKGLSGDDGESG-----WTGRNGRVWLDCLP  195
CYP53C2__130996__Pch        131  KRKIVSHIFSQKSVLEFEPHVRLYVKQLIQQWDRLYEAGAKGL-------------------VWLDCLP  180
CYP53C__128292__Fme         139  KRKIVSHIFSQKSVLEFEPHIRLHVGELFTQWDKLCDGGKRGLKGTEGDG------WEGHDGWVWFDCLP  202
Cyp53C__37267__Tve          131  KRKIVAHSFSQKSVHEFEPHVRENLSKLFKQWDTLCEGGAKGLSGNEGEGG-----WQGREGRVWYDCLP  195
CYP53C__70450__Ade          127  KRKIVSHVFSQKNVLGFEPNLHSALSRFVSQWDRMCAAGVKGGRGNE-QDG-----WHGDGQRVWMDCLP  190
CYP53C__52716__Dsp          138  KRKIVSHIFSQKSVLEFEPYIRQALGKLVKQWDSLLSDDRKLASHRLR---------PNENGTAWFDCLN  198
CYP53C__83844__Cpu          136  KRKIVANTFSQKNVIEFEPRVRIYVGQIIDQWDRLSKLAAADGSGDEGESG-----WYGKDERLWLDVLP  200
CYP53C__194303__Abi         134  KRKVISHIFAQKSVVAFEPKIAIYVTQLLNQWDRLYDMAVKGGSGNEGEGG-----WKGKDGKLYLDILP  198
CYP53C__194181__Abi         138  KRKIVSHIFSQKNVLEFEPHIRMYVAQLQNQWDRLYDMAVKGMSGNDGEGG-----WEGRDGRLWLDCLP  202
CYP53C__143663__Fme         134  KRKIISHVFSQKSVLEFEPFVHLHLAELFEHWDKMCDGGKEGLSGTESEGG-----WKRRGGQAWFDIMP  198
CYP53C__130308__Fme         135  KRKVISHGFSQKSVSEFEPYIRLHVSELFEQWDELYDGGRKGLTGVEGEGG-----WKGHDGRVWFNAMP  199
CYP53C__24265__Fme          135  KRKVIAHGFSQKSISQLEPYIRLHVAELFEKWDKLYDGGRKGLTGVEGHNS-----WEGHDGRVWFNAMP  199
CYP53C__149618__Fme         136  KRTAIAHAFSQKSVLEFEPYIRLHVAELFNQWDRMCNRGKNGLSGTEGEGG-----WIGQGGRVWFDIMP  200
CYP53C__94457__Fme          136  KRKAISHIFSQKSVLEFEPYIHTHLTDFFKQWDKLCDGGRKGFSGIEGEGG-----WKGHDGRVWFNAMP  200
CYP53C__154594__Fme         129  KRKAISHIFSPKSVIDFEPYIHLHLTELFEQWDKLYDGGKRGLSGVEGEG------WNGRQGRVWFNIMP  192
CYP53C__115179__Fme         135  KRKLVAPIFSQKSVLGFEPCVHSHVTELFEQWDKLCDGGKQGLTGNAGKGG-----WKGRDGRVWFDALP  199
CYP53C1__5__Uma_            142  KRKIVSHTFAPKTIVAFEPFIRREVQLLLERWDEFCDKATKDNTEGP----------RGIKGRAWLDSLM  201
```

```
CYP53B2__32280__Sro        137  KRKIVSHTFAPKSVREFEPYIASTVNLLLKKWDQLAAKAQKSPPSGTG--------GERMKGYAVIDSLD  198
CYP53B3__28617__Pgr        130  KRKIISSTFSQKSILEFEPYIADTLACFLRKIDQVASEPNLVQLPSHSWDS-----KHLNERWRIIDILP  194
CYP53C__101826__Pca        104  KRKYTAYAMSMKGIMEFEPNVREYQHMLVRQLDTLCAVGAQGIDGVLGSCP-----WTARDGWVLFDCMP  168
CYP53C__256510__Pca        121  KRKYTAHAMSVKGIMQFEPNVREHQQMLVKRLDTLCTVGAQGVDGVLGSCP-----WAARDGWVWFDCMP  185
CYP53C__102576__Pca        133  KRKYTSHAMSMKGITEFEPNVREYQHMLLKQLDTLCAVGAQGIDGVLGSCP-----WTTRDGWVLFDCMP  197
CYP53C__212559__Pca        132  KRKYTAHAMSMKYIMEFEPNVREHHHMLVKQLDTLCAAGAQGKDGILGTRP-----WTARDGWAWFDCMP  196
CYP53C__48859__Fpi         135  KRRMEAHMFAPQSIRAVEPISHSHVNELLRQWDGLISRVAKAQQGGPNGGHIGATTWNVQDGRVWIDCMP  204
CYP53C__86809__Fpi         135  KRRMEAHMFAPQSIRAVEPISHAHVNELLRQWDGLISRVAKAQQGGPNGGHIGATTWNVQDGRVWIDCMP  204
CYP53C__138909__Wco        138  RRRLEAHMFSLQSVRALEINARVHHNNLVNQWERLYSYVKQAEKGGIREDKLGASAWRVEDGRVWFDCMP  207
CYP53C__104840__Wco        140  KRRSESHMFSPQSVRALEGTARVHHGNFVNQWGKLYSYVAQAKSGEAKEGKLGACAWKVEDGRVWFNCMP  209
CYP53C__77097__Wco         144  KRRIESPMFSPQSVRALEGTTRVHHSDLASQWDNLYSYVKQAENGGSREGMLGESAWKVEDGRVWFDCMP  213
CYP53C__138864__Wco        138  KRRIVSPMFSQQSVRALEINARVHHNNLVSQWERLYSYVKQAEKGGIREDKLGASAWRVEDGRVWFDCMP  207
CYP53C__104855__Wco        138  KRRIESHMFSPQSVRALEGTASVHHGNLVSQWDKLYSYVKQAEGGGAKEGQLGASVWKVEGGRVWFDCMP  207
CYP53C__154264__Wco        138  KRRIESQIFSQQSVRELEGTARVHHIDLVSQWDKLYSYVKRAESGAAREG--------------------  187
CYP53c__138853__Wco        138  KRRIESHMFSPQSVRALEGIARMHHNDLVSQWDKLYSYVKRAESGAAREGSLGECAWKVKDGRVWLDCMP  207
CYP53C__154237__Wco         60  KRRIESQIFSQQSVRELEGTTRVHHIDLVSQWDKLYSYVKRAESDGVREG--------------------  109
CYP53D1__108845__Ppl       149  KRRIEAHMFSPRGIRALEDTARVHFQVLVRQWDTLCAHTDKAIRGSAEGTI-GTVHWKVHSGRVWFDCMP  217
CYP53D6__54877__Ppl        151  KRRVEAHMFAPQSIRALEETARVHFQVLLRQWDAMCAHAEKAGRGRADGAI-GAVPWKVHGGRVWFNCMI  219
CYP53D4__55859__Ppl        122  KRRVEAHIFAPQSIRALEDTARVHFEILVRQWDAMCAHAEKAGRGSAEGAI-GTVPWKVHDGRVFFNCML  190
CYP53D2v1__56013__Ppl      149  KRRVEAHMFSPRSIRALEDTARVHFQVLVRQWDALCAPTGKTVRGSAEGTL-GTISWKVHGDRVWFDCMP  217
CYP53D2v2__48082__Ppl      149  KRRVEAHMFSPRSIRALEDTARVHFQVLVRQWDALCAPTGKTGRGSAEGTL-GTISWKVHGDRVWFDCMP  217
CYP53D5__46728__Ppl        134  KRRVEAHMFSPQSIRTLESTVSVHFHALVDQWDALCAHIQKAGSGGAEGII-GSVSWKVHESRVWFDCMP  202
CYP53D3__60352__Ppl        136  KRRVEAHMFSPQSIRTLESTVSVHFHALEDQWDALCAHIQKAGSGGAEGII-GSVSWKVHDSRVWFD---  201
CYP53C__112429__Csu        133  KRRVVAHLFAPKTVRMFEGALHKYIGQLVQQWDGMYKNVETALPGTATAGKAGDMSWIVRDGRVWFDCMP  202
CYP53C__151209__Csu        136  KRRVVSHLFAPKTVKAFEPAVQNYVGQLVRQWDRLCKN-ADAQSVSVMSGVLGSMTWRAYDGCVWFDCMP  204
CYP53C__118598__Csu        149  KQRMVSHLFAPKTVRLFEEDVQKYVGQLVAQWDDMC---ARAKDGVVLTGHNGAMEWSTREGKVWFDCMP  215
CYP53H3__318972__Bad       129  KRKVVSNAFAQKSVLEFEPVVKQYVGAVLKHWSHMCNAASSGDGGIIGDMK-----WTAQGGRATFNVIK  193
CYP53H7__358536__Bad       133  KRKALAHTFSPKSVLEFEPIIWEYLGAVIRRWDQMCTAASQGRGGVVGEMT-----WSSEGGCAVFDAVK  197
CYP53H6__142452__Bad       132  KRKSLAHTFALKTVLEFEPVVKEYVGSIIKKWDRMCAAATKGEGGTIGEMT-----WISQGGRAVFDTLK  196
CYP53H4__160054__Bad       132  KRKILAHTFSQKTTLEFEPVVRQYIGDMFKQWDRMCTAAVMGKGGVIGEMP-----WKDQDGRAEFDTLK  196
CYP53H2__55123__Bad        132  KRKIVAHTFARKTVLEFEPIVQRYIHAVVRQWEHMCAVAADGKGGVIGNAS-----WTSQDRRAVFNTLK  196
CYP53H5__65034__Bad        129  KRKILSHTFSQKSTLEFEPMMGQHIGDLIRQWDYMCAMAAEGKGGVVGETA-----WTSHEGRAVVDTLD  193
Cyp53A__101387__Pst        137  KRRNYSGAFAPKTIRSYTTTVHRFLEELLVKLDKRAALPGE-------------------PDKAPIDMLI  187
CYP53A__105834__Cpu        100  KRRLVSNIFAAKSLQDCTPFIRDIVDSFVVQLDRLAAKN------------------------EELNLLY  145
CYP53A__56813__Ade         119  KRRLLSQPFSYQSIRGFEGFMRESLGRFVRRLDAVCAGECFGDA---------------VRPGGAIDALL  173
CYP53__1116154__Fpi        131  AKKCISGPLTHK----LGHVLPDVVDEMIHSFNDIMPDAEH--------------------DWQTVPALE  176
CYP53NS__152212__Pca       142  QRRGLAPAFQPRTVHAQYPALHKYLLQFADTIDRSAAAR-----------------------GRAVDLAQ  188
CYP53NS__92916__Fme        139  QRKGLAPAFQPITVNDQYPMLQRYLTEFIEVID-AAARS-----------------------GSVIDLST  184
Consensus_aa:                   KR+hht.hFt.pol..hpshhp.hh..hhpphDphhs.s.pt.sGs..ps......Wpsp.s..hhshh.
Consensus_ss:                   hhhhhhhhh hhhhhhhhhhhhhhhhhhhhhhhhhhhh                           eehhh


Conservation:                   6   5  78 56 8677  7575    5
CYP53A15__ACF15219.1_Clu   174  W-FNYLAFDVIGDLAFGEPFGMLKKGRDEAEVARG-GKI------------------------TYAPAIE  217
CYP53C__1025718__Fpi_      196  W-YNYLAFDIIGDLAFGAPFGMLDACADAAPVAIS-HEKAMSSYGETD-----------TPEITYFPAVQ  252
Cyp53C__68781__Pst         190  W-YNYLAFDIIGDLAFGQSFGMLHACKDSAPVALS-QDEAMKAYGSAS-----------GYKVVSIPAVQ  246
CYP53C__80617__Gtr         193  W-YNYLAFDIIGDLAFGSPFGMLKACKDSAPVAVS-HADAMAAYGKDD----------SAVQVRSLPAVQ  250
CYP53C__127772__Cpu        216  W-YNYLAFDIIGDLAFGSPFGMIHSAKDSAPVAVS-HADAMSAYGSSA----------SNIKVVHIPAVQ  273
CYP53C__94174__Shi         198  W-YNYLAFDIIGDLAFGSPFGMLQAAKDSAPVAKS-AKDAIAAYGQDE----------AKVEVVHIPAVQ  255
CYP53c__27029__Wco         196  W-YNYLAFDIIGDLAFGAPFGMLQACADAAPVVVS-HADAMASYGKGD-----------APEVAYFPAVQ  252
CYP53C3__110015__Ppl       195  W-YNYLAFDIIGDLAFGAPFGMLHACADAAPVATE-HKDAMASYGADN-----------APKVTYFPAVQ  251
```

```
CYP53C__116910__Csu        194  W-WNYLAFDIIGDLAFGAPFGMLHACADSAPVAIS-HEAAMKNYGDDA-----------APEVEHFPAVQ  250
CYP53C__1179842__Sla       201  W-YNYLAFDIIGDLAFGSPFGMILNAKDSAPVAVS-QKDAMKSYGSES-----------TYEVIEIPAVQ  257
CYP53C__55106__Dsq         196  W-YNYLAFDIIGDLAFGAPFGMLDACKDSAPVAVS-HKAAMAAYGSSD-SSKE-------IQIEHFPAVQ  255
CYP53C4__GL08839-P1.1__Gl  196  W-YNYLAFDIIGDLAFGAPFGMLLACKDSAPVAVS-CEAAMASYGSAS-SSKE-------IQIEHFPAVQ  255
CYP53C4__47512__Gsp        196  W-YNYLAFDIIGDLAFGAPFGMLIACKDSAPVAVS-CEAAMASYGSAA-SSKE-------IQIEHFPAVQ  255
CYP53C__129211__Tve        198  W-YNYLAFDIIGDLAFGAPFGMLTSCKDSAPVAVS-QDDAMATYGKDA-----------AYKVEHFPAVQ  254
CYP53C9__27837__Pbr        197  W-YNYLAFDIIGDLAFGHPFGMLQACQDAAPVAVS-QEAAMAAYGEGK-----------QFEVTNIPAVR  253
CYP53C7__118978__Bad       200  W-YNYLAFDIIGDLAFGSPFGMLQACRDAAPVAVS-QEDAMAGYGGKQ------------CDVVYIPAVQ  255
CYP53C__183109__Pca        196  W-YNYLAFDIIGDLAFGAPFGMLLAARDAAPVAVN-HEQAMASYGKEK------------SEVQYIPAVQ  251
CYP53C2__130996__Pch       181  W-YNYLAFDIIGDLAFGAPFGMLLAARDAAPVAVD-HEQAMASYGKEK------------SEVQYIPAVQ  236
CYP53C__128292__Fme        203  W-FNYLAFDIIGDLAFGSPFGMILKGKDAAPVAKD-QKAAIAGYGRESASEKSA------CDVTELPAVQ  264
Cyp53C__37267__Tve         196  W-YNYLAFDIIGDLAFGAPFGMLTSGKDSAPIAVS-QVDAMAAYGQGG-----------TLKVKHVPAIQ  252
CYP53C__70450__Ade         191  CAYNYLAFDIIGDLAFGSPFGMLDACADSANAAVG-GVNAL---KDGK-----------PMQTVSVPAIR  245
CYP53C__52716__Dsp         199  W-YNYLAFDIIGDLAFGEPFGMINSGADSASVAIH-GDDPTHLASGEK-----------KLEIVRVPAVK  255
CYP53C__83844__Cpu         201  W-MNYLAFDIIGDLAFGQPFGMILKAKDSAPVAVS-QDAAMDSYGKE-------------CKVIEVPAVK  255
CYP53C__194303__Abi        199  W-MNYLAFDTIGDLAFGEPFGMLAAAKDMAVVPKD-QQSAMNSYGKET----------KEEDILTVPVIE  256
CYP53C__194181__Abi        203  W-ANYLAFDIIGDLAFGEPFGMLQAAKDSAVVPKD-QKSMMKSYGKED----------ASIEVMEIPAVQ  260
CYP53C__143663__Fme        199  W-FNYLAFDIIGDLAFGSPFGMVRNAKDAAPIAVD-RKSAMAQYGPVITDNRGLEK--PVIDVREVHAIS  264
CYP53C__130308__Fme        200  W-CNYLAFDIISDLAFALPFGMLRNAKDAALTAVD-QKAAMSENGQVNTDMQDIEK--PVVAVREVPAVK  265
CYP53C__24265__Fme         200  W-LNYLAFDIIGDLAFGAPFGMLRNAKDAAPTAVD-QKAAMSENGQVN--IQDLEK--PVVAVREVPAVK  263
CYP53C__149618__Fme        201  W-FHYLAFDVMSDLAFGASFGMVRNAKDAAPIAVD-QRAAMAQYQGTRVDSLDLEK--PSIDVKEVPAVT  266
CYP53C__94457__Fme         201  W-YNYLSFDIISDLAFGTPFGMIRKARDAVPVAID-HKAAMAQYGQIDTEYRDVKK--LVIDTREVPAIQ  266
CYP53C__154594__Fme        193  W-FNYLTFDIIGDLAFGAPFGMIRKGKDAAPVAVD-LKAAIAQYGQAGIDGQDLEK--PAIQVKEVPAVQ  258
CYP53C__115179__Fme        200  W-LYYMCFDIIGDLVLGAPFNMVHKGTDTVPVALE-PSAVIAQYGQSS-ITGSHDTEKPICAVKEAPAME  266
CYP53C1__5__Uma_           202  W-LNYFAFDTIGALAFGKTFGMLENGVDQAKVEYE-DANGNKQ-------------------VDYCSAVQ  250
CYP53B2__32280__Sro        199  W-FNALAFDVIGELAFGTPFGMVERDAADIVTITK-EDGT----------------------VIHAGGVQ  244
CYP53B3__28617__Pgr        195  W-FNYLAFDIIGDLAFGERFGMIERGADIAAVEKE-GKV------------------------IYLPAIQ  238
CYP53C__101826__Pca        169  W-LNFATFDVIGDLAVGAPFGMLEAGKDTALVPVS-EEQAMKSFGQQD-TDL---------EWATIPTIK  226
CYP53C__256510__Pca        186  W-FNFETFDIIGDLAFGASFGMLEAGKDTAPVPVY-TDQAMKSYGQKD-TDL---------EWSTAPAVQ  243
CYP53C__102576__Pca        198  W-LNFDTFDVIGDLAFGKPFGMLEAGKDAALVPVS-EEQAMKSFGRQD-TDL---------KWATIPAIK  255
CYP53C__212559__Pca        197  W-FNYETFDIIGDLAFGAPFGMVEAGKDTASVPVS-EKQAMKFYGQKG-AEI---------EWSTAPAIK  254
CYP53C__48859__Fpi         205  W-LNFWSFDTIGDLAFGLPFGMLKSGRDTAKVAKS-AEDALKAIDTVS-KGGDVLA----IEEEEIPYIE  267
CYP53C__86809__Fpi         205  W-LNFWSFDTIGDLAFGLPFGMLKSGRDTAKVAKS-AEEGFKAIDAMS-KGGDALV----VEEEEIPYIE  267
CYP53C__138909__Wco        208  W-FSYWSFDTIGDLAFGSPFGMLSAASDTVRVAKS-VKASLATFGTSS-NAEEFGF-----ETEEMAIEK  269
CYP53C__104840__Wco        210  W-FNYWSFDTIGDLAFGAPFGMLLSAKDTVRVATS-VKAGMAAFGTSS-TTGKFTL-----ETEEIPATK  271
CYP53C__77097__Wco         214  W-FNYWSFDTIGDLAFGAPFGMLLAAKDTARVAKS-VKAGLATFGTVS-RTGEFAF-----ETEEIPVTK  275
CYP53C__138864__Wco        208  W-FSYWSFDTIGDLAFGSPFGMLSAANDTVRVAKS-VKASLATFGTSS-NAGEFGF-----ETEEMAIEK  269
CYP53C__104855__Wco        208  C-----------DLAFGAPFGMILAAKDTARFAKS-VMAGMAAFGTSS-KTSEYAF-----ETDETPVTK  259
CYP53C__154264__Wco        188  --FNYWSFDTIGDLAFGDPFGMILAAKDTARSAKS-VKASLETFSTSS-NTEKLAF-----ETEELPVTK  248
CYP53c__138853__Wco        208  W-FNYWSFDTIGDLAFGAPFGMILAAKDTARVAKS-VKASLATFGTPS-QTGKFAF-----ETEELPVIK  269
CYP53C__154237__Wco        110  --FNYWSFDIIGDLAFGDPFGMILAAKDTARSAKS-VNASLATFSTSS-NTKKFAF-----EMEELPVTE  170
CYP53D1__108845__Ppl       218  W-FTFWSFDTISDLAFGHPFGMLEAAKDTAKISKS-NIKGMQAISQGN-SHSDEAE----LELEEIPAIE  280
CYP53D6__54877__Ppl        220  W-FSYWSFDTIGDLAFGHPFGMLETGKDVAQIAKS-NARGMQAIAQGT-SDSEKAT----LELVDIPAIE  282
CYP53D4__55859__Ppl        191  W-FSFWSFDTIGDLAFGHPFGMLETGKDTAQTVKS-DVRGMEAIAQAT-SNSEKTK----LELVDIPAIE  253
CYP53D2v1__56013__Ppl      218  W-FNFWSFDTISDLAFGRPFGMLEAAKGSAHVSKS-NTKSVQAVSQDT-SHSNEAQ----SELLEIPAME  280
CYP53D2v2__48082__Ppl      218  W-FNFWSFDTISDLAFGRPFGMLEAAKGSAHVSKS-NTKSVQAVSQDT-SHSDEAQ----SELLEIPAME  280
CYP53D5__46728__Ppl        203  W-FMFWSFDSIADLSFGRPFGMLVSAKDVVRIPKS-NASGIQAIAEAA-SHSEKTE----LEMADVPLIE  265
CYP53D3__60352__Ppl        202  W-FMFWSFDTIADLSFGRPFGMLVSAKDVVRIPKS-NASGIQAIAEAA-SHSKKTE----LEMVEVPLIE  264
CYP53C__112429__Csu        203  W-LNFLAFDTIGDLAFGSPFGMLVSGKDTARIAKS-LKAAMQTLGSTP-SATEKPS---TIEEEDIPAIS  266
CYP53C__151209__Csu        205  W-LNFLSFDTIGDLAFGKAFGMVESGKDIARVAKD-YTDAMRTYNAKQ-ELPEWTP---AYEEEEIPAIS  268
CYP53C__118598__Csu        216  W-FNLLAFDTISDLAFGSPFGMLIAGRDTARVARS-VDIAMKNLGVAQ-TAQESDR---IYEEEDIPAIS  279
```

```
CYP53H3__318972__Bad       194  W-YNFMVFDIIGDLVFRAPFGMTEHGTDIARIAKN-RDHAMASYDSGE-VKL---------EYDTVNAVQ  251
CYP53H7__358536__Bad       198  W-YNFMVFDIIGHLVFRHPFGMTERATEMTLIVKQGRDDAMELQDKPG-REL---------EYTSIPAVQ  256
CYP53H6__142452__Bad       197  W-YNFMVFDIIGDLVFRAPFGMTERATEIAVIAKR-RDKAIESYETSE-QKL---------EYTTLPAVQ  254
CYP53H4__160054__Bad       197  W-YNFMVFDIIGDLVFRAPFSMTERGTDMARIVKR-PEKAMSSYESVD-TKL---------EYDTINAIE  254
CYP53H2__55123__Bad        197  W-YNFMVFDIVGDLVFRNPFGMTERGSDMALIAKH-PDQVMASYNSIT-EEKI--------QYDAVNAVD  255
CYP53H5__65034__Bad        194  W-YSFVIFDVIGELVFRIPFGMTNRGSDETLIVKH-PNQTMALDESSS-TKT---------EHDSVRAVQ  251
Cyp53A__101387__Pst        188  W-SNYLVFDIMSTLAFGTPLGMLEKESDVLQAGSP-------------------------------KGAIE  226
CYP53A__105834__Cpu        146  W-FHFLAFDVLSDLAFGQRIGMVEKGSDAVTVQKR-DGSV-----------------------STENAIA  190
CYP53A__56813__Ade         174  W-FNYLAFDIISDLAFGEPLGMVNKGSDLLPAERK-DGTI-----------------------FEEHAAA  218
CYP53__1116154__Fpi        177  T-MIKIIARVTNRVFVGMPFCR---NEMLL-----------------------------------ETAVE  207
CYP53NS__152212__Pca       189  L-HVLLTLDFVGEVAFGAELRAVRDG-AACRILQI-FHAV------------------------------  225
CYP53NS__92916__Fme        185  L-HVLLTLDFVGEVAFGAELNALRDG-ASCRILQI-FHDI------------------------------  221
Consensus aa:                   h.hshhsFDhlt-LtFG.sFGMl..tps.h.l..p....sh..hsp.p.............ch.phstlp
Consensus ss:                   h hhhhhhhhhhhhh             hhhhhhh hhhhhhhhhhh               hhhhhhhh


Conservation:                               6  6                 5                         5   6
CYP53A15__ACF15219.1_Clu   218  VLNRRGEVSGTVGIFPA-IKPYA-----KYFPDPFFSQG-MKAVENLA---------GIAIARVNARLEK  271
CYP53C__1025718__Fpi_      253  ILNDRGEYSASLGVLPPHWRPIV-----KLL--PWYRKG-NKAVQRLA---------GIAIAQVAKRLAM  305
Cyp53C__68781__Pst         247  ILNDRGEFSASIGVLPPAWRPFV-----KNLI-PWYRNG-SKAVKNLA---------GLAVAAVAKRLDR  300
CYP53C__80617__Gtr         251  ILNDRGEYSASMGVLPVWFRPVV-----QRLH-PWYRNG-NKAVKDLA---------GLAVAAVAKRLRN  304
CYP53C__127772__Cpu        274  ILNDRGEYSAAMGVLPPAIRPFM-----QRFV-PWYRKG-GKAVRNLA---------GIAVAAVAKRLNE  327
CYP53C__94174__Shi         256  ILNDRGEFSASMGVLPPWLRPYV-----KRYI-PWFSKG-DQAVKNLA---------GLAIAAVSKRLNQ  309
CYP53c__27029__Wco         253  VLNDRGEFSASLGVLPPHWRPLV-----VRFI-PWYRNG-NKAVKRLA---------GIAIAAVAKRLTA  306
CYP53C3__110015__Ppl       252  VLNDRGEYSASMGVLPPHWRPLV-----VRFI-PWYRNG-GKAVKRLA---------GIAIAAVSKRLTA  305
CYP53C__116910__Csu        251  VLNSRGEYSASMGVLPPHWRPLA-----KRI--PWFRRG-NQAVQRLA---------GIAVAAVAKRLSA  303
CYP53C__1179842__Sla       258  ILNDRGEFSASMGVLPPHWRPLV-----RLL--PWYRKG-GKAVKNLA---------GLAVAAVAKRLTT  310
CYP53C__55106__Dsq         256  VLNDRGEYSAAMGVLPPHWRPLA-----KKI--PWYSKG-NQAVQKLA---------GIAVAAVAQRFAN  308
CYP53C4__GL08839-P1.1__Gl  256  VLNDRGEYSASMGVLPPHWRPLA-----KRI--PWFAKG-NQAVQRLA---------GIAVAAVAQRLGS  308
CYP53C4__47512__Gsp        256  VLNDRGEYSASMGVLPPHWRPLA-----KRI--PWYARG-NQAVQRLA---------GIAVAAVAQRMAS  308
CYP53C__129211__Tve        255  VLNDRGEYSASMGVVPPRWRPLV-----KRL--PWYNKG-NQAVQRLA---------GIAIAAVARRLSV  307
CYP53C9__27837__Pbr        254  ILNDRGMFSASLGVLPPWMRPLA-----KQL--PWFKKG-NAAVKTLA---------GIAVAAVARRLAT  306
CYP53C7__118978__Bad       256  ILNDRGNFSASLGVLPPWMRPIV-----KQL--PWFKKG-QKAVKDLA---------GIAIAAVAKRLTT  308
CYP53C__183109__Pca        252  VINDRGMYSASLGVLAPWMRPIV-----KLF--PWFRQG-QQAVKLLA---------GIAVAAVSQRLTT  304
CYP53C2__130996__Pch       237  VINDRGTYSASLGVLPPWMRPIV-----KLF--PWFRRG-QKAVKQLA---------GIAVAAVAQRLTT  289
CYP53C__128292__Fme        265  VLNDRGEYSASMGVLPPWWRPFV-----RRI--PWYANG-NRAVKNLA---------GLAVAAVAKRLAN  317
Cyp53C__37267__Tve         253  VINDRGEYAASVGVLPPHWRPFV-----KRL--PWYNTG-DKAVQNLT---------GMAIAAVARRMEE  305
CYP53C__70450__Ade         246  ILNERGEFSATMGVLAPWMRPLV-----LKL--PWFARG-LSAVRALA---------GLAIAAVGRRLAE  298
CYP53C__52716__Dsp         256  ILNDRGEYSASMGCLPIWIRPYA-----KKI--PWYAKG-NQAVKNLA---------GIAIAAVDKRLAT  308
CYP53C__83844__Cpu         256  ILNDRGDYNATLGTMPPWVRPYV-----RKL--PWFSQG-SEAAASVA---------GMAVAAVSRRLTT  308
CYP53C__194303__Abi        257  AFNNRGEFNLVMGSLPLHWRPLA-----RRL--PGLAQG-SRDFKTVA---------GIAVAAASKRLSS  309
CYP53C__194181__Abi        261  ILNGRGEFSLTMGTLPPYWRPIA-----RRL--PGFRQG-AQDVKNLA---------GIAIAAVAKRLAT  313
CYP53C__143663__Fme        265  VLENRMRLSAQMGVLPAWWRPIV-----RQL--PRFAQG-VQNSKDLV---------DLAVAAVAKRMAY  317
CYP53C__130308__Fme        266  VLNGRSEYSASMGVLPPWWRPIV-----RLL--PWYADG-SQDVEDLA---------GLAVAAVAKRLAI  318
CYP53C__24265__Fme         264  VLNGRSEYSASMGVLPPRWRPIA-----RLL--PWYAEG-SKDVEDLA---------GLAVAAVAKRLAI  316
CYP53C__149618__Fme        267  MLNAHIKVSARMAAVPPRWRPIL-----QRL--PCFARD-MRASEDLV---------ALAVAAVARRLVF  319
CYP53C__94457__Fme         267  VVNEQGEGVAQIAAFPPLWVPFL-----RCL--PRFAKG-MRRVEDFI---------GLVVLAVANRLAF  319
CYP53C__154594__Fme        259  ILNDRTFLASHQAAFPKPLRPLL-----ALL--PQYAEM-AKHSDEFI---------GFAVAAVAKRLVF  311
CYP53C__115179__Fme        267  LMNGRSAVIASLGVLPPWWRPIA-----SLF--PWYARG-NRDVGDLA---------GFATLAISKRLAR  319
CYP53C1__5__Uma_           251  IINERGEFSGTMGLAPVWMRPYL-----IKL--PWFSSR-LKSVKKLT---------GIALARVNDRLQN  303
CYP53B2__32280__Sro        245  ILNMRGEYSATLGCLPPWSRKYM-----KYID-PWFARG-LESVKNLT---------GIARTRVNDRLEK  298
```

```
CYP53B3__28617__Pgr         239  ILNERGEFSATQGQFDHIKFSKKKKEKKKYID-PWFSRG-AASVQNLT---------GIATNQVNLRISQ  297
CYP53C__101826__Pca         227  LLNETVPWIFFLGCLP-----------------PQARFL-MSKLQSFNAGASRKLFQKLGVAAVSKRLSS  278
CYP53C__256510__Pca         244  ILNEAIPWFFFLGCLP-----------------PQARRL-VSTLQSFNAGGSRNLIGKIAVAAVSKRLTS  295
CYP53C__102576__Pca         256  LLNETVPWTFFLGCLP-----------------PQARFL-MSKLPSFNAGTSRKLFVKLAVATVSKRLAS  307
CYP53C__212559__Pca         255  IVNEAVPWVFFLGCLP-----------------PHVRPL-LSKLRSFTTKASARALVKIAVAAVSKRLAT  306
CYP53C__48859__Fpi          268  YQSARAETDACLAWLPPIWVRIL-----GKL--PMFSVH-ALTGQKLA---------ALSIMAVARRIAD  320
CYP53C__86809__Fpi          268  YLSARAERNACLAWLPPVWARVV-----LTL--PAFSGY-ALTGRKLA---------ALSIMAVARRLAN  320
CYP53C__138909__Wco         270  VLNERGHLITILGWLPEYWRRIV-----QML--PAYRFG-MEAAPKMA---------GLAVAAVGKRLNN  322
CYP53C__104840__Wco         272  LLNKRAKLVTTLGWFPKYWQPII-----ELL--PPFRAG-REATPKLA---------GLAVAAVAKRLSN  324
CYP53C__77097__Wco          276  LLNKRAELVAILGWLPKYWQSIV-----GTL--AVFSGG-SNASPKLA---------GLAVASVAKRLSN  328
CYP53C__138864__Wco         270  MLSERGHLISILGWLPEYWRRIV-----QML--PAYRFG-TEAAPKMA---------GLAVAAVGKRLNN  322
CYP53C__104855__Wco         260  LMAERADLVATIGWLPEYWQPIV-----RML--PAFRGG-RKSTPQLA---------GLAVAAVAKRLSK  312
CYP53C__154264__Wco         249  IMGQRGELVAMLGWLPEYVRPII-----LMM--PGFRSN-LQAIPKVA---------GLAVAAVAKRMNN  301
CYP53c__138853__Wco         270  IVGQRAELVAMFGWLPEYVWPII-----LMM--PGFRGS-RRAIPQVS---------GLAVAAVAKRMNN  322
CYP53C__154237__Wco         171  IIGQRGELITMLGWLPKYVRPII-----LMM--PGFRSN-LQAIPKVA---------GSAVTAVAKRMND  223
CYP53D1__108845__Ppl        281  MLAEHLDIIVSLAFLPAWLQPIV-----GRL--PSVRYG-YDAAPKLA---------GLAVAAVANRFAS  333
CYP53D6__54877__Ppl         283  VLTARADALFVVAYLPPWAQKIV-----GRL--PSFRSG-YAAAPKLA---------GMAVAAVANRLAS  335
CYP53D4__55859__Ppl         254  ALTARADTLFVVAYLPAWLQPIV-----GHL--PSLQSG-YNAAPKLA---------GLAVAAVANKFAS  306
CYP53D2v1__56013__Ppl       281  VLSELLDFTVALAYLPAWVQPVF-----GRL--PMFRDG-YDAAPKLA---------NLSLTAVANRVAS  333
CYP53D2v2__48082__Ppl       281  VLSELLDFTVALAYLPAWVQPVF-----GRL--PMFRDG-YDAAPKLA---------NLSLTAVANRVAS  333
CYP53D5__46728__Ppl         266  VLEIRGKTVAALAYLPAWAQPII-----GRL--PGFREG-YGAIPKLN---------GIAIAAVANRLRS  318
CYP53D3__60352__Ppl         265  VLVKRGKTIAALAYLPAWAQPII-----GRL--PGFREG-YGAIPKLN---------GIAIAAVADRLRS  317
CYP53C__112429__Csu         267  SINRRSEFLIAFASLPAWIRPIV-----KRL--PMASDG-MAATREIM---------SMAVTTVSRRVRT  319
CYP53C__151209__Csu         269  SLKERSKYMIFVGTLPKSLRGIM-----RFL--RSRDHQ-VMALRRIM---------SMATSSVARRIRA  321
CYP53C__118598__Csu         280  ANNTRSDFLMFLAYLPEWLRPIV-----VRL--PLFDDG-VAAAGKIM---------SMAVTTVLRRLHA  332
CYP53H3__318972__Bad        252  ALLSQNKFLTVVAQLPAYLRPLL-----QSVPLPWVRSG-AEAFQKFS---------TLSVTAVANSQQY  306
CYP53H7__358536__Bad        257  SINTRSTFLATMGMLPKSWRPLL-----RKLPLSWFNGG-HLASEFML---------SLAMTAVSHRLQH  311
CYP53H6__142452__Bad        255  AINKRGTFLATMGMLPKYWWPLL-----RKLPLSWFSAG-HAAAEMML---------TLAITAVAHRSQH  309
CYP53H4__160054__Bad        255  AVNARSAFLITLGVLPALWRPLI-----KKLPLPWVRAG-TEAFQKIT---------KLTVTVVADRSHH  309
CYP53H2__55123__Bad         256  AVTALNVVLTAVGMLRPFWRPFI-----LKLPMPWIRAG-NEGVAKIA---------MMAVAAVAQCSQH  310
CYP53H5__65034__Bad         252  MMNERSALMVSLGMLPKYWRPLI-----ERLPMEWIRRG-NAGVEKMS---------SLVMSAIADGSKN  306
Cyp53A__101387__Pst         227  NTDVREHYLTVIGWAPAL-AYIA-----RLIPDPFFQKG-SKSSDELR---------DIARMCIKQRLAS  280
CYP53A__105834__Cpu         191  LVDEREHLGAVLGVHPSF-KFWS-----KFLPDPFFIQG-RKSSDGLV---------DFARRQVSRRIDN  244
CYP53A__56813__Ade          219  LIDQRGRTAAVVGLMPSI-EDIT-----KKLPIPFITAG-YKSTESLS---------RIAVRCVKHRIQS  272
CYP53__1116154__Fpi         208  FAKDVMKTKFIVNLFPDVLKPIV----GHRL--PWTTARRRKMAEILG---------DTVRERRRQMLEY  262
CYP53NS__152212__Pca        226  -----LPELMKCGLFPL--RAKV-----PVF--ESTRAM-HRAIAELR---------GMARAAVEDARRA  271
CYP53NS__92916__Fme         222  -----LPELMKCGLFPL--RSQI-----PIM--ESTRRM-HRSIKELR---------GMAHVAVKNAR--  265
Consensus_aa:                    hhs.p..h.h.hthhP.hhpshh.......h..P.hp....psh.pl..........shAhhsV.pph..
Consensus_ss:                    hhhhhhhhhhhhhhhhhhhhhhh     hh   hhhhhh hhhhhhhh          hhhhhhhhhhhh


Conservation:                                 8 8  7           5         66 55      5 56 6656 5 55  5
CYP53A15__ACF15219.1_Clu    272  PS--------DRVDLLARLMEGRDEN----GNKL---GREELTAE-ALTQLIAGSDTTSNTSCALLYHCL  325
CYP53C__1025718__Fpi_       306  HT--------DRSDLLGKLQEGKDDE----GNPM---GREELTAE-ALTQLIAGSDTTSNSSCAITYHLA  359
Cyp53C__68781__Pst          301  DTLN---GGSDRVDLLAKLQQGKDDE----GKPM---GREELTAE-ALTQLIAGSDTTSNSSCAITYHLA  359
CYP53C__80617__Gtr          305  PT--------DRVDLLSKLQEGKDDE----GKPM---GREELTAE-ALTQLIAGSDTTSNSSCAITYYLA  358
CYP53C__127772__Cpu         328  PS--------DRVDLLRRLQEAKDDE----GNPM---GREELTAE-ALTQLIAGSDTTSNSSCAITYYLA  381
CYP53C__94174__Shi          310  PT--------YRVDLLSKLQEGKDDE----GRPM---GREELTAE-ALTQLIAGSDTTSNTSCAITYYLA  363
CYP53c__27029__Wco          307  PT--------DRSDLLAKLQEGKDDD----GNPM---GREELTAE-ALTQLIAGSDTTSNSSCAITYYLA  360
CYP53C3__110015__Ppl        306  PT--------DRADLLGKLQEGKDDE----GNPM---GREELTAE-ALTQLIAGSDTTSNSSCALTYHLA  359
CYP53C__116910__Csu         304  PS--------DRTDLLSKLQEGKDDE----GKLM---GKPELTAE-ALTQLIAGSDTTSNSSCAITYHLA  357
```

```
CYP53C__1179842__Sla       311  PT--------DRVDLLSKLQEGRDDE----GKLM---GREELTAE-ALTQLIAGSDTTSNSSCAITYYLA  364
CYP53C__55106__Dsq         309  PS--------DRADLLSKLQEGRDDN----GDPM---GREELTAE-ALTQLIAGSDTTSNSSCALTYWLA  362
CYP53C4__GL08839-P1.1__Gl  309  PS--------DRVDLLAKLQEGRDDN----GDPM---GREELTAE-ALTQLIAGSDTTSNSSCAITYWLA  362
CYP53C4__47512__Gsp        309  PS--------DRVDLLAKLQEGRDDN----GDPM---GREELTAE-ALTQLIAGSDTTSNSSCAITYWLA  362
CYP53C__129211__Tve        308  PE-------SDRHDLLEKLQEGRDDN----GDPM---GRAELTAE-ALTQLIAGSDTTSNSSCAITYYLA  362
CYP53C9__27837__Pbr        307  PV--------DRVDLLGKLQDGRDDE----GNPM---GREELTAE-ALTQLIAGSDTTSNSSCAITYHLA  360
CYP53C7__118978__Bad       309  PS--------DRTDLLGKLQQGRDDE----GNPM---GRPELTAE-ALTQLIAGSDTTSNSSCAITYHLA  362
CYP53C__183109__Pca        305  PT--------DRVDLLGKLQGGRDDD----GNLM---GKEELTAE-ALTQLIAGSDTTSNSSCAITYYLA  358
CYP53C2__130996__Pch       290  PT--------DRVDLLGKLQEGRDDD----GNLM---GKEELTAE-ALTQLIAGSDTTSNSSCAITYYLA  343
CYP53C__128292__Fme        318  PT--------DRTDLLSKLQEGKDDE----GRPM---GREELTAE-ALTQLIAGSDTTSNSSCAITYHLA  371
Cyp53C__37267__Tve         306  SD-------SDHRDLLAKLREARDED----GNPM---GREELTAE-ALAQLVAGSDTTSNSSCAITYYLA  360
CYP53C__70450__Ade         299  PS--------DRNDLLAKLQDAKDDD----GMPM---GPEELTAE-ALTQLIAGSDTTSNSSCAIAYYVA  352
CYP53C__52716__Dsp         309  PT--------DRVDLLARLQQGKDEQ----GNLM---ARSELTAE-ALAQLIAGSDTTSNSSCAITYWLA  362
CYP53C__83844__Cpu         309  PT--------DRVDILSKLQQGKDEN----GEIM---GPEELTAE-ALTHLVAGSDTTANSSCAIIYYLA  362
CYP53C__194303__Abi        310  ST--------DRIDLMSKLQNSRDSN----GNPM---SREEMTAE-ALTLLVAGSDTSSNACAAFLYHVA  363
CYP53C__194181__Abi        314  PT--------DRNDLLSNLQAGRDSE----GKPL---GREELTAE-ALTLLIAGSDTTSNSTCAILYYLA  367
CYP53C__143663__Fme        318  PT--------QRDDILSKLQQSRDEY----GRPL---TQEDLTTD-AITQLVAGSDTISISSCGIAYHLA  371
CYP53C__130308__Fme        319  PT--------DRTDLLSKLQQGRHED----GRPL---NREELTAD-ALTQLIAGSDTTANSSCAVLYHII  372
CYP53C__24265__Fme         317  PT--------DRADILSKLQQGRHED----GSPM---SREELTAD-ALTVLIAGSDTTSNSTCALMYYIT  370
CYP53C__149618__Fme        320  PA--------ERIDVLSKLQETEDEH----GRVS---NMEDLTTD-AFTQLVAGSDTVSSTACGIAHCVA  373
CYP53C__94457__Fme         320  PT--------ERVDILSKLQQGKGED----GVPL---TKEELTSE-ALVQLIAGSDTTSNTTCAITYYVA  373
CYP53C__154594__Fme        312  PT--------ERVDILSKLQQSKDEN----GNPQ---SREDLTTD-GITQLVAGSDTVANTSCGITYHIA  365
CYP53C__115179__Fme        320  PT--------EPLGLLSALLELKDDE----GKPL---SKEQLSAD-GLLLLIAGSDMVANPTCAVLYQII  373
CYP53C1__5__Uma_           304  GS--------EREDLLAKLQAAKDDR----GEPM---GKMELTAE-ALTQLIAGSDTTSNTSCAIVYHLA  357
CYP53B2__32280__Sro        299  GA-------LDRKDILSHLQAGRDEN----GQPM---SKDELTME-ALTQLIAGSDTTSNSSCAILFQIV  353
CYP53B3__28617__Pgr        298  TG-------QSRRDLLARLQTGQDAD----GNPM---GKDELIAE-ALTQLIAGSDTTSNSSCAILWWVV  352
CYP53C__101826__Pca        279  EA--------TRRDFLSQLVAARDDE----GKPL---SAQELTSE-ALNLIIAGSDTTSSSIGAIIYHIA  332
CYP53C__256510__Pca        296  EV--------TRRDFLSHLVAAHDDQ----GRPL---SQQELTSE-AISLIVAGSDTTSTSIAAITYHVA  349
CYP53C__102576__Pca        308  EA--------TRRDFLSHLVAARNDE----GKPL---SAQELTAE-ALNLIVGGSDTTSSSIGVVIYHVA  361
CYP53C__212559__Pca        307  GV--------TRRDFLSHLIAVRDDQ----GRPL---TEQELTSE-AISLIVAGSDTTSSSIAAIAYHVA  360
CYP53C__48859__Fpi         321  PN--------PREDMLQKLLEARDEE----GKPL---SPQEMSAE-AFVLIIAGSDTIANTTCGTTYYLA  374
CYP53C__86809__Fpi         321  PN--------PREDMLQKLLEARDEE----GKPL---SPQEMSSE-AFLLIIAGSDTIANTTCGTTYYLA  374
CYP53C__138909__Wco        323  PA--------AREDMLNRLLDARDEN----GKPM---SPEELSAE-AFVLIIAGADTTANTSCATTYYLA  376
CYP53C__104840__Wco        325  PD--------AREDMLNRLLDARDEN----GEPL---SPEELSAE-AWLLIIAGADTVANTSCATTYYLA  378
CYP53C__77097__Wco         329  PQ--------AREDMLNRLLDARDDN----GEPM---SPEELSAE-AMTLIIAGADTVANTSCATTYYLA  382
CYP53C__138864__Wco        323  PA--------AREDMLNRLLDARDEN----GKPM---SPEELSAD-AFQLIVAGADTTANTSCATTYYLA  376
CYP53C__104855__Wco        313  PD--------AREDMLNRLLNARDED----DEPL---SREELSAE-AAMLIAAGADTVANTSCATTYYLA  366
CYP53C__154264__Wco        302  PD--------ARADMLNRLLDA-----------------------------PGAITVANTSCATTYYLA  333
CYP53c__138853__Wco        323  PD--------ARADMLNKLLDARDEN----GEPM---SPEELSSE-AFLLIVAGSDTVSNTSCATTYYLA  376
CYP53C__154237__Wco        224  LD--------AHADMLNRLLDARDEN----GEPM---SPEELSSE-ASLIIVAGAITVANTSCAITYYLA  277
CYP53D1__108845__Ppl       334  KTK------IDRADMLSELLRGRDED----GKPY---GPEELSAE-AELLIIAGGDTTANSSCATTYHLA  389
CYP53D6__54877__Ppl        336  QY--------DRADMLSKLLQGRDED----GKPY---SPEELSAE-AWVLIIAGGDTTANSSCALTYHLA  389
CYP53D4__55859__Ppl        307  KT--------DRADMLSKLLEGRDKN----GNLY---GPEELSAE-TWLLIIAGGDTTANTSCATTYYLA  360
CYP53D2v1__56013__Ppl      334  QT--------DRADMLSELLRGRDEE----GKPY---GLEELSTE-AELLIIAGGDTTANTSCATAYYIA  387
CYP53D2v2__48082__Ppl      334  QT--------DRADMLSELLRGRDEE----GKPY---GLEELSTE-AELLIIAGGDTTANTSCATAYYIA  387
CYP53D5__46728__Ppl        319  PN--------GRADMLTKLLEGRDGD----GHSY---SPQELSAE-ARTLIAAGGDTTASASCAITYYIA  372
CYP53D3__60352__Ppl        318  PN--------GRADMLTKLLEGRDGE----GYRY---GPQELSAE-AKTLIAAGGDTTASASCAITYYIA  371
CYP53C__112429__Csu        320  LYDGD-AEERQRPDFLTKLLEGRDEE----GSPL---SPDELSSE-AQTLLIAGSDTISNSTCAIVYWIA  380
CYP53C__151209__Csu        322  GRE------QDRDDFLARLLQARDDD----GNPL---SPDELSSE-AQTLLTAGADTISNSTCATVFWIA  377
CYP53C__118598__Csu        333  LSSDSGDEKKNYEDFLIKLLQGHNDD----GNRM---GPEELTSE-AQVLLIAGSDTISNSTCATVYWVA  394
CYP53H3__318972__Bad       307  PA--------PRNDILGRYFEATDEK----GQKM---GNHELSSE-AVSLLIAGTDTTSNSAAALTFYLA  360
```

```
CYP53H7__358536__Bad         312 QT--------TFDDILGKYLEATDDR----GQKM---NDEELIAE-ALTLLIGGTDTTSSTVAALTFYLA 365
CYP53H6__142452__Bad         310 NA--------IRSDILGKYMEARDDR----GQQL---DNRELSSE-ALTLLIGGTDTTSNSAAALTFYLA 363
CYP53H4__160054__Bad         310 SA--------TRNDILAKYFDATDEN----GQKL---DAQELSSE-AITLLIAGTDTTSNSVAAMTFYIA 363
CYP53H2__55123__Bad          311 PA--------SRNDILAKYFDAIDDR----GEKM---HDLELSAE-AVGLLIAGTDTTSNSLGALTFYLA 364
CYP53H5__65034__Bad          307 SS--------QRNDILSKYFNATDED----GRKM---GISELYTE-ALVLLAGADTTAHSLAALTFYLA 360
Cyp53A__101387__Pst          281 GSD------DAKSDILGHLIAAHMEY----KNHL---DVEELTSE-ALTLLIAGTDATSNAITAIIHALS 336
CYP53A__105834__Cpu          245 RL--------QRNDILDKLIRARVAD----DQEIVGENFADLVAE-TVTLLIAGSDTTSNSETAIMHLLF 301
CYP53A__56813__Ade           273 GV--------TRDDMLERLIDGVREK---QGGEV---SEEEVVTE-AMLLLTAGADTTANSLTAILYFIL 327
CYP53__1116154__Fpi          263 GTDY----EGKPDDYLTWVVEEDLKN---RGKGE---SIDGVMEV-IAASNFAAIHTSSMAMAHALYYLC 321
CYP53NS__152212__Pca         272 H-------------------EDSQEKDCGAEKGK---KIFEILAH-HVPRRRRRSHGTHDDFCRG-HRQL 317
CYP53NS__92916__Fme          266 ---------------------SSEEKSAVQPGSK---RIYEILAQLSVNLKLLHVLVT---FLPL-LFQL 307
Consensus aa:                    .s........+.DhLs+Lbptpp-p....Gp.h...s.pEl.s-.thhblhAGscThtss.tthh@@lh
Consensus ss:                                  hhhhhhhhh               hhhhhhh hhhhhh   hhhhhhhhhhhhhhh


Conservation:                           68  778  5                          7 77 565 9 69  5  5 7868  5
CYP53A15__ACF15219.1_Clu     326 QHPEVVQKLQNELDAALPNPD------------AVPSYAQVKDLPYVDAVIKETMRIHSTSSLGLPRVIP 383
CYP53C__1025718__Fpi_        360 ANPMVQQKLQRELDEALGNDD-----------DPVSTFEQVKRLPYLEAVINEGLRLHSTSGIGLPRLVP 418
Cyp53C__68781__Pst           360 ANPNVQAKLHAELDEALGTDD-----------DPVAIFDQVKRLTYLQAVIDETLRIHSTSGIGLPRIVP 418
CYP53C__80617__Gtr           359 LHPRVQEKLQAELDEALGNDD-----------DPVSTFEQVKRLKYLEAVINEALRVHSTSGIGLPRVVP 417
CYP53C__127772__Cpu          382 LHPEIQTKLQRELDDALGTDD-----------DPVSTFDAVKRLPYLDSVINEALRLHSTSSIGLPRIAP 440
CYP53C__94174__Shi           364 ANPAVQEKLHVELDAALGNED-----------DPASTFEQTKNLKYLQAVIDESIRLHSTSGIGLPRIAP 422
CYP53c__27029__Wco           361 ANPLVQQKLQRELDEALGNDD-----------DPVAMYEQVKRLPYLEAVINEGLRLHSTSGIGLPRIVP 419
CYP53C3__110015__Ppl         360 ANPRVQQKLQRELDEALGSDD-----------DPVATYEQVKRLPYLEAVVNEALRVHSTSGIGLPRVVP 418
CYP53C__116910__Csu          358 ANPHVQEKLQAELDAALGDG------------DPVATFDQVKRLPYLEAVINEALRIHSTSGIGLPRIVP 415
CYP53C__1179842__Sla         365 QNPDAQEKLQKELDEALGDDD-----------HPVSTFEQVKRLPYLEAVINEALRVHSTSSIGLPRIVP 423
CYP53C__55106__Dsq           363 KNQAAQRKLQQELDAALGSDD-----------DPVASYEQVKRLPYLEAVINEALRIHATSGIGLPRLVP 421
CYP53C4__GL08839-P1.1__Gl    363 RNQAAQRKLQAELDGALGSASD---------DDSIASFEDVKRLPYLEAVINEALRIHATSGIGLPRLVP 423
CYP53C4__47512__Gsp          363 RNPAAQRKLQAELDGALG--SN---------DDPIASFEDVKRLPYLDAVINEALRIHATSGIGLPRLVP 421
CYP53C__129211__Tve          363 KYQHVQEKLQKELDDALGGED-----------DSVASYEQVKRLPYLDAVINEALRIHATSGIGLPRLVP 421
CYP53C9__27837__Pbr          361 KNPDVQKKLQAELDEVLGNDD-----------DPVSTYEEVKKLAYLQAVIDEALRIHSTSGIGLPRVVP 419
CYP53C7__118978__Bad         363 KNPEVQRRLQKELDDALGAHAD----------EPVVTFEDVKRLPYLQAVIDEALRIHSTSGVGLPRLVP 422
CYP53C__183109__Pca          359 KYPDVQRKLQQELDEVLGYDD-----------EPVSTYDQVKKLTYLPAVIDEALRVHSTSGVGLPRVVP 417
CYP53C2__130996__Pch         344 KYPDAQRKLQQELDEALGSDD-----------EPVSTFDQVKRLPYLQAVIDEALRIHSTSGIGLPRLVP 402
CYP53C__128292__Fme          372 HNPHVLKRLQQELDTALAGED-----------DPVATFQQVKSLPYLDAVINEVLRIHSTSGIGLPRLVP 430
Cyp53C__37267__Tve           361 KHQRVQEKLQQELDEALASEE-----------DEVALFERVKHLPYLEAVINEALRIHSTAGVGLPRVVP 419
CYP53C__70450__Ade           353 RYPRVQLKLQQELDAALPN-------------DGVTTYEQVKRLPYLTAVINEGLRLHSTSAMGLPRIVP 409
CYP53C__52716__Dsp           363 KYPDAQRKLQKELDEALGDD------------EDVPTYEQLKRLRYLDAVVNEGLRIHSTSSLGLPRIVP 420
CYP53C__83844__Cpu           363 AYPHVQEKLQKELDEALGSED-----------EPVTTYEQVKRLTYLEVVILEVLRLHSTIGLGLPRMAP 421
CYP53C__194303__Abi          364 ANPSVQDKLHQELDEQLGTED-----------ELVATAEQIKRLTYLEACINEALRIQSVSGIGLPRVVP 422
CYP53C__194181__Abi          368 RNRGAQEKLQKELDEHLGTEN-----------EFTATEAQVKNLPYLDACINEGLRLHSTSSVGLPREVP 426
CYP53C__143663__Fme          372 ANPDVQSKLQKEIDDALGGFD-----------DPMVTYAQIKHLQYLEAVINEGLRVHPTPGLGLPRVVP 430
CYP53C__130308__Fme          373 SSPRVQAKLQKELDEALASLD-----------DPVASYDLVKHLPYLDAVIHEGLRVHSTSGNGLPRLVP 431
CYP53C__24265__Fme           371 SNPRVQAKLQKELDEALVSFD-----------DPVTSYDLVNHLPYLDAVIHEGLRVHSTLGVGLPRLVP 429
CYP53C__149618__Fme          374 ANSRVRAKLQQELDVVFGGSY-----------DPVATYAQIKRLPYLEAVIIEGLRVHSTSGLGLPRTVP 432
CYP53C__94457__Fme           374 ANPHVQTKLQKELDNALGHSE-----------NHVATYSQIKQLSYLDAVVNEGLRVHSTVGIGLPREVP 432
CYP53C__154594__Fme          366 SNPCVQAKLQAELDDALGKDME----------DPVVTYAQIKNLPYLEAVLNEGQRVYSTAALGLQRIVP 425
CYP53C__115179__Fme          374 ANPPVQAKLQKELDDALGAPSP--------SDDSVSTYSQINHLPYLEAVINEALRVHPMVGLGLPRVVP 435
CYP53C1__5__Uma_             358 THPDKMRKLQAELDRELEHAE------------EVPLHADVQELPYLQAVLSESLRYHSTSAIGLPRVIP 415
CYP53B2__32280__Sro          354 STPHAHKKLQQELDEAFSGKG----------MSGVLEYEDVKALPYLGACINEALRRHSTSGIGLPRIMM 413
CYP53B3__28617__Pgr          353 KHPEVHKRLMEELDEHLGTE------------EGVISYADCKELKYLNACINETLRIHSTSSIGLPRILP 410
```

```
CYP53C__101826__Pca          333  RNRDVQERLQKALDDVLGVPNSM-----FSTDEVVASFDLVKNLTYLQDVINEGLRLHSTVGVGLPREVP  397
CYP53C__256510__Pca          350  RTQDVQAKLQEELDDALGVPDAS-----SNADNVVAPFDLVKNLAYLQDVINEGLRLHSTIGVGLPREVP  414
CYP53C__102576__Pca          362  RNRDVQERLQKELDDVLGVPNST-----FSTDEVVAPFELVKNLTYLQDVINEGLRLHSTVGVGLPREVP  426
CYP53C__212559__Pca          361  RNQDVQAKLQAELDDVLGAPGSD-----SSTDDVVAPFDRVKNLTYLQDVINEGLRVHSTLGAGLPREVP  425
CYP53C__48859__Fpi           375  RDKRVQAKLQAELDGALASVD-----------SEVAPYDTVKDLPYLDAVIHEGQRLYSTIGAGLPREVP  433
CYP53C__86809__Fpi           375  RDKRVQAKLQAELDGALASVD-----------SEVVPYDAVKDLPYLDAVIHEGQRLHSTVGAGLPREVP  433
CYP53C__138909__Wco          377  RDQRVQAKLQAELDEALKSID-----------SAVAPYDAVKNLPYLDAVINEGLRLHATIGAGLPRVVP  435
CYP53C__104840__Wco          379  RDQRVQTKLQAELDEALKSID-----------SAVAPYDAIKHLPYLDAVVNEGLRLHATVGAGLPRVVP  437
CYP53C__77097__Wco           383  RNQRVQAKLQAELDEALKAVD-----------SEVALYDAVKYLPYLDAVVNEGLRLHATIGAGLPRVVP  441
CYP53C__138864__Wco          377  RDQRVQAKLQAELDEALKSID-----------SAVAPYDAIQNLPYLDAVVNEGLRLYTTVGAGLPRVVP  435
CYP53C__104855__Wco          367  RDQRVQAKLQAELDEALKSVD-----------SVAAPYDAIKHLPYLDAVVNEGLRLHATIGAGLPRVVP  425
CYP53C__154264__Wco          334  RDQRVQAKLQTELDDALKAVD-----------SVVAPHGAIKHLPYLDAVVNEGLRLHSPVGAGLPRVVP  392
CYP53c__138853__Wco          377  RDQRVQAKLQAELDDALKAVD-----------SVVAPHDAIKHLPYLDAVVNEGLRLHSAVGAGLPRVVP  435
CYP53C__154237__Wco          278  RDQRVQAKLQAELDDALKADD-----------SIVAPHDAIKHLPYLDAVVNEGLRLDS----------P  326
CYP53D1__108845__Ppl         390  RNPRIRAKLQAELDAALEGID-----------SDVAPYDAVKDLPYLDAVINEGLRLHSTIGAGLPRVVP  448
CYP53D6__54877__Ppl          390  RNPRVQAKLQAELDAALDGID-----------SDVAPYDAVKDLPYLDAVINEGLRLHSTVGGGLPRVVP  448
CYP53D4__55859__Ppl          361  RNPRIQAKLQAELDTALDGID-----------SDVASYDAVKDLPYLGAVINEGLRLHATVGVGLPCVVP  419
CYP53D2v1__56013__Ppl        388  RDLQIQAKLQAELDVALDGVE-----------SDVAPYDAVKDLPYLDAVINEGLRLHSTIGAGLPRVVP  446
CYP53D2v2__48082__Ppl        388  RDLQIQAKLQAELDVALDGVE-----------SDVAPYDAVKDLPYLDAVINEGLRLHSTIGAGLPRVVP  446
CYP53D5__46728__Ppl          373  RDPRIQAKLQAELDAALDGTG-----------SEIAPYGAVKVLPYLEAVVNEGLRLHSGVGAGLPRVVP  431
CYP53D3__60352__Ppl          372  RDPRIQAKLQAELDAALDGIG-----------SEIAPYGTVKVLPYLEAVVNEGLRLHSGVGAGLPRVVP  430
CYP53C__112429__Csu          381  RNPDVQKKLQAELDAALADAG----------EGPIAPVEKTERLLYLNAVIDEGLRVHSTVGANLPRVVG  440
CYP53C__151209__Csu          378  RAPPVKARLQAELDATLGISST-------DLGSPVAPIDKIEHLPYLNAVIDEALRIHSTVAAGLPREVG  440
CYP53C__118598__Csu          395  RHLNVQRNLQSELDGALADVSS-------DEDSFVAPIDKIDNLPYLNAVVDEGLRVHSAVGANLPRTVG  457
CYP53H3__318972__Bad         361  HNPAAQAKLQVELDKALGPPCAL----DGDNDPAIVSYDQVKNLSYLHDVVNEGLRLFSAVGLGLPRVVP  426
CYP53H7__358536__Bad         366  QNPVVQAKLQKELDEAFGNPSAVAANDDDDGQPNLVKYERIKNLTHLQDVVNEGLRLFSTIGLGLPRVVP  435
CYP53H6__142452__Bad         364  QNTIVQARLQKELDEALGEPVYGD---EDAERPVLVAYELIKNLPYLQDAVNEGLRLFSTIGLGLPRVVP  430
CYP53H4__160054__Bad         364  HNPVVQARLQAELDEALGCPVCE-----SREEPVLATYEQIKGLSYLQDVVNEGLRVFSTVGLGLPRIVP  428
CYP53H2__55123__Bad          365  QHPSAQSKLQAELDGALGAPHAF-----GNDDVESVSYENIKNLQYLQDVVNEGLRLFSIAGFGLPRIVP  429
CYP53H5__65034__Bad          361  QCPAAQTKLQVELDEALGWPNAV----DDGNRPTIASYDSVKNLPYLQDVVNEGLRLFSAVGVGLPRVVP  426
Cyp53A__101387__Pst          337  VNPRPLAKLREELDEALSPGG------------LQGPTSDLNDLPYLNACIHEAIRLHSPTGMGLPRIVP  394
CYP53A__105834__Cpu          302  TNPRVYNKLIGILEEAVDEE--------------LPTADHVRDIPYLDAVINEGLRYHATTAIGLHRAVH  357
CYP53A__56813__Ade           328  TRPDVYKKLMAELDSINAPTAELD---TGTTIDGLPTHDQVKNLPYLNAVIEEGLRLFATNAFGLPRVSS  394
CYP53__1116154__Fpi          322  AMPQYIKPLKQEAEEKIKEH--------------GWTKTAMDAMWKTDSFFKESLRLNGVNHLSLFRKSM  377
CYP53NS__152212__Pca         318  RNPAIHRKLRDELDALLPADC------------VVPSIEQVSRLPYLRLVIKETLRYNGP-GFGTFRYTP  374
CYP53NS__92916__Fme          308  RNPEILAKVRTELDEVLPPDS------------EIPTVEQASRLRYLHLVIKETLRYNGP-GFGTFRYTS  364
Consensus_aa:                     .p..hb.+Lp.ELD.hL...s...........s.lsshp.lcpL.YLpsVlpEtLRlpthsthGL.R.hs
Consensus_ss:                     h hhhhhhhhhhhhhh                     hhhhh  hhhhhhhhhhhhh   hh


Conservation:                         6    7  6  8  5757 5  65    78 7   759698
CYP53A15__ACF15219.1_Clu     384  P-GPGVTILGRHFPQGTVLSVPAYTIHHSTEIWGPDADTFRPERWE----KVTE------------QQKA  436
CYP53C__1025718__Fpi_        419  E--GGLTVCGRFFPEGTVLSVPSYTIHRDQDVWGSDADAFRPERWF----EQDE-----------KAIQK  471
Cyp53C__68781__Pst           419  AGSGGMHVAGHFFPEGTVLSVPTYTIHRDKEVWGEDVEVFRPERFL----EGDQ-----------AVIQK  473
CYP53C__80617__Gtr           418  E--GGLTVLGRTFPEGTIMSVPTYTIHRYEEVWGPDVDEFRPERWF----EIDQ-----------AQINK  470
CYP53C__127772__Cpu          441  E--GGLALRGLWFPPGAILSVPSYTIHRDAGVWGADTEAFRPERWA----EEERRD----------AVQR  494
CYP53C__94174__Shi           423  E--GGLTVCGKYFPEGTILSVPSYTIHRDVDVWGYDVEAFRPERWF----ERDA-----------EMIQK  475
CYP53c__27029__Wco           420  E--GGLTVRGQFFPEGTVLSVPSYTIHRDREVWGADVDAFRPERWM----ELDK-----------NAVQK  472
CYP53C3__110015__Ppl         419  E--GGLSVCGRFFPAGTVLSVPTYTVHRDAETWGADVDAFRPERWE----ERDK-----------NAVQK  471
CYP53C__116910__Csu          416  Q--GGLTAAGQYFPEGTVLSVPTYTVHRDKEAWGEDADLFRPERWF----EHDE-----------KTLQR  468
CYP53C__1179842__Sla         424  E--GGLIVQGQHFPQGAVLSVPSYTIHRDTTVWGADPDQFRPERWF----ECDH-----------AAIQK  476
```

```
CYP53C__55106__Dsq         422  E--GGLTVCGKFFPEGTVLSVPTYTIHRDKAVWGEDVDDFRPERWF----EQDK-----------NLVQK  474
CYP53C4__GL08839-P1.1__Gl  424  E--GGLTVCGRFFPEGTVLSVPTYTIHRDREVWGEDVDAFRPERWF----ERDK-----------NLVQQ  476
CYP53C4__47512__Gsp        422  E--GGLTVCGRFFPEGTVLSVPTYTIHRDREVWGEDVDAFRPERWF----ERDK-----------NLIQK  474
CYP53C__129211__Tve        422  A--GGLEVCGRWFPEGAVLSVPTYTIHRDKAVWGDDVEEFRPERWF----EQDK-----------VAVQK  474
CYP53C9__27837__Pbr        420  E--GGLTVCGQFFPEGTVLSVPTYTIHRDTHVWGDDVETFRPERWF----EQDD-----------KLIQK  472
CYP53C7__118978__Bad       423  E--GGLTVCGQYFQEGTVLSVPTYTIHRDKEIWGEDCEAFRPERWF----EQDK-----------NGIQK  475
CYP53C__183109__Pca        418  E--GGMTVCGRTFPEGTILSVPTYTIHRDEEVWGKDVEVFRPERWF----SQDK-----------NEVQK  470
CYP53C2__130996__Pch       403  K--GGMTVCGRFFPEGTVLSVPTYTIHRDEEVWGKDPEVFRPERWF----EQDK-----------NAVQK  455
CYP53C__128292__Fme        431  E--GGLTVCGKTFPEGTVLSVPTYTIHRDKEVWGEDVEAMRPERWL----EGDQ-----------AAIQK  483
Cyp53C__37267__Tve         420  A--GGLEVCGKFFPEGTVLSVPGYTIHRDKAVWGDDADEFRPERWF----GKDK-----------AALQK  472
CYP53C__70450__Ade         410  E--GGLTVAGRFFTEGSILSVPSYTIHRDPEVWGEDFDKFRPERWS----EGDQ-----------TLIQK  462
CYP53C__52716__Dsp         421  E--GGLEVSGIHFPAGSVLSVPSYTIHRDTAIWGPDPDIYRPERWF----EQDA-----------EGIQA  473
CYP53C__83844__Cpu         422  E--GGLTVHGTYFPEGTILSVPTYTLHRDKRVWGDDPEIFRPERWF----EENS-----------AKMHK  474
CYP53C__194303__Abi        423  E--GGLEVLGNFFPEGTVLSVPSYSVHRDTKSWGDDTETYRPERWF----ERDQ-----------AAMNK  475
CYP53C__194181__Abi        427  E--GGMMVCGQFFAEGTVLSVPSYTIHRDRGVWGEDFEAYRPERWF----ERDQ-----------TLMQK  479
CYP53C__143663__Fme        431  E--GGLNVCGKWFPEGTILSVPTYTIHRNTGVWGEDANVFRPERWF----EGDQ-----------AAMQK  483
CYP53C__130308__Fme        432  E--GGLTVCGKWFPAGTVLSAPTYTIHRDPKVWGEDADVFRPERWL----ERGQ-----------ATLLK  484
CYP53C__24265__Fme         430  E--GGLTVCGKWFPEGTVLSAPTYTIHRDPKVWGEDADVFRPERWL----ERDH-----------ATLLK  482
CYP53C__149618__Fme        433  N--GGLIVCGKWFSEGTVLSVPTYTIHRDPIVWGEDADAFRPDRWF----ERDQ-----------TILQK  485
CYP53C__94457__Fme         433  E--GGITVLGKSFPEGTVLSVPIYTIHRDPKVWGKDVDSFRPERWI----EGDK-----------AAMQK  485
CYP53C__154594__Fme        426  E--GGLTISGKWFPEGTIVSVPTYTIHRDPKVWGEDVDVFRPERWL----EGDH-----------SAMSK  478
CYP53C__115179__Fme        436  A--SGLTVCGKHFPEGTVLSVPTYTIHRDKEVWGEDADTFRPERWF----EGDK-----------STMQK  488
CYP53C1__5__Uma_           416  A--GGATVCGQQFPSGTILSVPAYTLHRDKSVFGADAEEYNPDRWL----APNAK----------RDFEK  469
CYP53B2__32280__Sro        414  ---DDTEVLGEVFPKGTVLSVPSYTIHWSTEFWGPDAAEFKPERWL----ESEEKTR---------QLEK  467
CYP53B3__28617__Pgr        411  ---QTVSFKGHILPKGLVCSVPTFEIHHDPDVWG-DPFTFRPERWL----EPNA-----------KDREK  461
CYP53C__101826__Pca        398  E--GGMTVAGKTLLAGTHVSCPTYTLHRLKSIWGDDADEFNPDRWT----RGDR-----------NMMLK  450
CYP53C__256510__Pca        415  E--EGLTVAGKALLPGTHVSCPLYTLHRLKSIWGDDADEFNPDRWA----RGDR-----------KAMLK  467
CYP53C__102576__Pca        427  E--GGMTVAGKALLPGTHVSCPTYTLHRLKSIWGDDADEFNPDRWT----RGDR-----------NMMLK  479
CYP53C__212559__Pca        426  E--GGLTVAGKTLLAGTHVSCPSYTLHRLKSIWGDDADQFNPDRWT----LGDR-----------NAMLK  478
CYP53C__48859__Fpi         434  A--GGATILGHHFKEGTTISVPIYRLHRDESTWGPDAAEFRPERWI----EASPERKK--------LMMD  489
CYP53C__86809__Fpi         434  T--GGATILGHHFREGITLSVPIYRLHRDESIWGPDAAEFRPERWI----EASPERKK--------LMMD  489
CYP53C__138909__Wco        436  E--GGLTVLGHTFKEGTWVSVPVYHLHRDESIWGENASEFYPERWI----EASGDRKK--------AMLD  491
CYP53C__104840__Wco        438  E--GGLTVLGHTFKEGTWVSVPVYHLHRDESIWGENANEFYPERWI----EASGDRKK--------AMLD  493
CYP53C__77097__Wco         442  E--GGITVLGHTFKEGTCVSVPIYYLHRDESIWGANATEFYPERWL----DATGERKK--------AMLD  497
CYP53C__138864__Wco        436  E--GGLTVLGHTFKEGTWVSVPIYRLHQDESIWGENVNEFYPERWI----EASGDRKK--------AMLD  491
CYP53C__104855__Wco        426  E--GGLTMLGHTFKEGTWVSVPVYHLHRDESIWGENASEFYPERWI----EASGERKK--------AMLD  481
CYP53C__154264__Wco        393  E--GGMTVLGHTFKEGTWVSVPTYHLHRDENIWGENASEFYPERWI----EASGDQKK--------AMLD  448
CYP53c__138853__Wco        436  E--GGMTVLGHTFKEGTWVSVPIYHLHRDENIWGENASVFYPERWI----EASGDQKK--------AMLD  491
CYP53C__154237__Wco        327  A--PGSAYLR-----------------------GENASEFYPERWV----EASGDQKK--------AMLD  359
CYP53D1__108845__Ppl       449  P--GGLTVLGQHLKEGTVVSSPLYCLHRNEVVWGENAHEFYPERWL----EASADAKK--------EMMR  504
CYP53D6__54877__Ppl        449  A--GGLTVLGQHLKEGTVVSSPIYTLHTNEVVWGENAHEFYPERWL----EASADAKK--------EMMR  504
CYP53D4__55859__Ppl        420  P--GGLTVLGHHLKEGSVVSSPIYSLQRSEAVWGENAREFYPERWL----EASADAKK--------EMMR  475
CYP53D2v1__56013__Ppl      447  S--GGMTVLGQHLKEGTVVSSPIYTLHRNEAVWGKNAYEFYPERWL----EASADAKK--------EMMQ  502
CYP53D2v2__48082__Ppl      447  S--GGMTVLGQHLKEGTVVSSPIYTLHRNEAVWGKNACEFYPERWL----EASADAKK--------EMMQ  502
CYP53D5__46728__Ppl        432  A--GGMTILGHHLMEGTVVSSPIYTLHRSKAVWGANADEFYPERWI----DASADTKK--------EMMS  487
CYP53D3__60352__Ppl        431  A--GGMTILGHHLMEGTVVSSPIYTLHRSKAVWGANADEFYPERWI----DASADTKK--------EMMS  486
CYP53C__112429__Csu        441  P--EGVTILGHTFTEGTVVSVPAYSTHRDENIWGHDAEFFRPERWL----EADKEKRD--------AMNK  496
CYP53C__151209__Csu        441  P--GGLNVLGHFFPEGAVLSVPTYSAHRDESIWAPDPDAYRPERWI----EADKEKRE--------AMNK  496
CYP53C__118598__Csu        458  P--EGATVLGHSFQEGTILSVPAYSAHRDEQVWGLDCEEFRPERWL----EADREQQE--------LMKK  513
CYP53H3__318972__Bad       427  E--SGLTVLGRTFAPGTVVSVPTHVLHHDKTIWGDDAESFNPDRWT----EGDK-----------TAMME  479
CYP53H7__358536__Bad       436  D--GGLTVLGNTLAPGTVVSVPTYIIHHDEEIWGNDAWSFNPNRWQ----TRDK-----------DTMSK  488
```

```
CYP53H6__142452__Bad        431  E--SGLVVLGEAFTPGTVVSVPTYVTHHDEAIWGEDSWAFNPERWQ----TGDK-----------AVMAK  483
CYP53H4__160054__Bad        429  D--GGLTVLGRTLSPGTVVSVPTYVLNRDKSIWGDDAEYFNPDRWA----NGNK-----------AAMSK  481
CYP53H2__55123__Bad         430  E--GGLTILGHHFAPGAVISVPLYVVHRDKSVWGDDAEVFNPDRWA----AGDR-----------VAMTK  482
CYP53H5__65034__Bad         427  E--GGLTILGRTLPPGTVVSVPAYVVHRDQAAWGDDVESFNPDRWA----KGDK-----------TGMMR  479
Cyp53A__101387__Pst         395  E--GGLTYRDYYFPPGTDVSVPTWTMSRDRAAWGEDADVFRPERWI----EDP-------------SLTK  445
CYP53A__105834__Cpu         358  E--KGAMFGGKYFPPGTEMSVPAWTIQHDPEIWG-DPEVFRPERWI----ENP-------------DLKK  407
CYP53A__56813__Ade          395  R--EGFELDGWTIPAGVEVSAPAYTIQRDPRIWGPDADDYRPERWI----DETD-----------SGLKK  447
CYP53__1116154__Fpi         378  K--DVVLSNGTVIPAGTIVVATSTGTHLQEALY-KDAAEFRPFRFS----DVREKGGADAQKQQFHIPTA  440
CYP53NS__152212__Pca        375  ---ADVEIEGVVLPANTTLALWNPQVHRCPNVWGADADTFRPERWM----STQGEGNE------KAALPG  431
CYP53NS__92916__Fme         365  ---KDVEINGVTLPANTTLALWNPQVHRDPKLWGPDSDEFRPERWLVSTGSTSSEFSR------FIPPPG  425
Consensus_aa:                    ...sshph.Gphh..GThlShPs@plH+s..lWG.Dhp.FpP-RWh....c.s..............h.p
Consensus_ss:                         eee  eee    eeeeehhhh   hhh    hh


Conservation:                     5 588 96869 86 69  66    65 5 57   6                               5
CYP53A15__ACF15219.1_Clu    437  AFIPFSYGPRACVGRNVAEMELALIVATVFRRYEFELRQGE--------------------METRE-GFL  485
CYP53C__1025718__Fpi_       472  TFNPFSFGPRSCVGRNLASMELLIILSSILRRYHFVLEHPEQG------------------LDTKE-GFL  522
Cyp53C__68781__Pst          474  TFNPFSFGPRACVGRNLANMELLIIIASILRRYHFVLEHPEKP------------------FDTRE-GFL  524
CYP53C__80617__Gtr          471  AFNPFSYGPRACVGRNLASMELMIIVSSIFRRYHFVLEEPEKK------------------FETRE-GFL  521
CYP53C__127772__Cpu         495  AFNPFSFGPRACVGRNLASMELLVIVSSILRRYTFVLEDAAKP------------------FDTRE-GFL  545
CYP53C__94174__Shi          476  AYNPFSFGPRACVGRNLASMELLIIISSILRRYDFVLEDPTKP------------------FATKE-GFL  526
CYP53c__27029__Wco          473  TFNPFSFGPRSCVGRNLASMELLIIIGSILRRYHFVLEDADKK------------------FDTRE-GFL  523
CYP53C3__110015__Ppl        472  AFNPFSFGPRSCVGRNLASMELLIIIASILRRYHFVLEEPKHK------------------LETKE-GFL  522
CYP53C__116910__Csu         469  AFNPFSFGPRSCVGRNLANLELLIIIASILHRYHFVLEDPEKH------------------FDTRE-GFL  519
CYP53C__1179842__Sla        477  TFNPFSFGPRACVGRNLASMELLIIISSILRRYHFVLADPEKP------------------FDTRE-GFL  527
CYP53C__55106__Dsq          475  TFNPFSFGPRSCVGRNLANLELLVIVASIFRRYHFVLENPNAQ------------------LETRE-GFL  525
CYP53C4__GL08839-P1.1__Gl   477  AFNPFSFGPRSCVGRNLANLELLVIVASIFRRYEFVLEDPTAE------------------LDTRE-GFL  527
CYP53C4__47512__Gsp         475  TFNPFSFGPRSCVGRNLANLELLVIVASIFRRYEFVLEDPAAE------------------LDTRE-GFL  525
CYP53C__129211__Tve         475  TFNPFSFGPRSCVGRNLANLELLVIVASIFRRYHFVLEDPSAP------------------LATNE-GFL  525
CYP53C9__27837__Pbr         473  TYNPFSYGPRSCVGRNLASMELLIIISSILRRYEFVLENPSKP------------------LETLE-GFL  523
CYP53C7__118978__Bad        476  TFNPFSFGPRSCVGRNLANMELLIIVASILHRYDFVLADPEKP------------------FDTAE-GFL  526
CYP53C__183109__Pca         471  TFNPFSFGPRSCVGRNLASMELLIIISSILRRYDFVLEEPDKP------------------FDTME-GFL  521
CYP53C2__130996__Pch        456  TYNPFSFGPRSCIGRNLANMELLIIVSSILRRYDFVLEDPDKP------------------FDTME-GFL  506
CYP53C__128292__Fme         484  TFNPFSFGPRACVGRNLASMELLIIIASIFRRYEFVLEKPDEQ------------------FDTRE-GFL  534
Cyp53C__37267__Tve          473  AFAPFSVGPRSCVGRNLAHLELLTFVASIFRRYSFVLENPDEP------------------LPTNE-GFL  523
CYP53C__70450__Ade          463  TFNPFSWGPRACVGRNLAMMELLLIVSTTFRRYHLVLESDDEA------------------SQTRE-GFL  513
CYP53C__52716__Dsp          474  TFNAFSFGPRACVGKNLASMELLLIVATIFHRYEFALLSQDQP------------------LETRE-GFL  524
CYP53C__83844__Cpu          475  AFNTFSFGPRACVGRNLANLELLIIVSSLLRRYDFVLKNPGDA------------------LGTCE-GFL  525
CYP53C__194303__Abi         476  AFNPYSVGPRSCVGRNLAAMELSIILASIMRRYEFVLKDEDKP------------------LVISE-GFL  526
CYP53C__194181__Abi         480  TFNPFSYGPRACVGRNLASMELLIILASIMRRYDIVLEDPDLI------------------LDTRE-GFL  530
CYP53C__143663__Fme         484  VFNAFSFGPRACIGRNLAMMELYIIIASIFHRYELILEEPNKP------------------LEIHE-AFM  534
CYP53C__130308__Fme         485  AFNTFSYGPRACIGRNVATMELFIIVSSIFRRYEFVLEEPHKP------------------LEVHE-GFI  535
CYP53C__24265__Fme          483  VFNTFSYGPRACIGRNVATMELFIFISSIFRRYDLVLEEQNKP------------------LEVHE-GFI  533
CYP53C__149618__Fme         486  AFSPFSFGPRACIGRELAIMELCIFVSSIFHRYDLELEAPDKP------------------LTIRE-DFI  536
CYP53C__94457__Fme          486  VFSPFSVGPRACTGRNLALMSLHIFIASIFRRYDIVLEQPDKP------------------LEVHD-AFA  536
CYP53C__154594__Fme         479  TLNTFSIGPRACVGRNLALLELHIFIASIFYRYELVLEEPDKP------------------LETHE-GFI  529
CYP53C__115179__Fme         489  VFNAYSYGLRACAGRVLANVELQIFISSIFRRYEFVLEEPENP------------------VEVFE-GFI  539
CYP53C1__5__Uma_            470  AFIPFSVGPRACVGRNVAMMELSILIAAIFRRYDIVLAEPDKP------------------LDTFE-GFL  520
CYP53B2__32280__Sro         468  QLNVFSFGPRSCVGRNVAMIELFCFMATLVYRYDFKLVDPNQK-----------------ELEVVE-GFL  519
CYP53B3__28617__Pgr         462  AFMPFSCGPRSCIGRNLAMMELYMITSTIFKRYEFALVDPDLA-----------------ELETRE-GFL  513
CYP53C__101826__Pca         451  YFAPFSIGPRACIGRNLAMMEMTICIATMFHRYRLVLASPDQQ------------------LECSE-GFV  501
```

```
CYP53C__256510__Pca        468  YFAPFSTGPRACVGRNLATMEMTICIATIFHRYRVVLASPDQQ------------------LECHE-GLV  518
CYP53C__102576__Pca        480  YFAPFSIGPRACIGRNLAMMEMTICIATIVHRYRLVLANPDQQ------------------LECSE-GFV  530
CYP53C__212559__Pca        479  YFSPFSVGPRACIGRNLAMMELTICIATIFHRYRVVLASPDQR--------------------VSQ-LFN  527
CYP53C__48859__Fpi         490  AFAPFSVGPRACIGRSLAIMQLHIIVATLFHRYDFTLQSDEP-------------------LRVRD-SLA  539
CYP53C__86809__Fpi         490  AFAPFSVGPRACIGRSLAIMQLHIIVATLLRRYDFALQSDEP-------------------LRVRD-SFA  539
CYP53C__138909__Wco        492  AFAPFSMGPRSCIGRNLALMQLHIVLSTLFHRFDFVLESDDP-------------------LPVQD-SFV  541
CYP53C__104840__Wco        494  AFAPFSVGPRACIGRNLALMQLHIVLSTLFHRFDFVLESHDP-------------------LPVQD-SFV  543
CYP53C__77097__Wco         498  AFTPFSVGPRACIGRNLALMQLHIVIATLFRRFNVVLESDYS-------------------LPVQD-SFV  547
CYP53C__138864__Wco        492  AFAPFSMGPRSCIGRNLALMQLHIVLSTLFHRFDFVLESDDP-------------------LHVQD-SLI  541
CYP53C__104855__Wco        482  AFAPFSVGPRACIGRNLALMQLHKTLATLFYRFSFVLESSDPVRLRSCIRFCSTADSVRNQLPVQD-GFA  550
CYP53C__154264__Wco        449  AFVPFSIGPRACIGRNLALMQLHIVLATLFHRFNVVLESDDP-------------------VRSQA-YAL  498
CYP53c__138853__Wco        492  AFVPFSIGPRACIGRNLALMQLHIVLATLFRRFNVVLESDDP-------------------LPVQD-SFG  541
CYP53C__154237__Wco        360  AFVPFSIGPRACIGCNLASMQLHTVLATLFHRFNVVLESDDP-------------------VRSQV----  406
CYP53D1__108845__Ppl       505  SFAPFSVGPRACLGRNLALQQMHIVLATIFHRYNLVLESDAP-------------------LPLRD-GFV  554
CYP53D6__54877__Ppl        505  SFVPFSAGPRACLGRNLALQQLHVMFATIFRRYSFALENDAQ-------------------LTIQE-SFV  554
CYP53D4__55859__Ppl        476  SFAPFSVGPRACLGRNLALQQLYIMFATLFRRYDFVLENDAP-------------------LAVQD-RLV  525
CYP53D2v1__56013__Ppl      503  SFAPFSVGPRACLGRSLALQQLHILLATIFHRYSLVLENNAPA-----------------QLPLRD-GFA  554
CYP53D2v2__48082__Ppl      503  SFAPFSMGPRACLGRSLALQQLHILLATIFHRYSLVLENNAPA-----------------QLPLRD-GFA  554
CYP53D5__46728__Ppl        488  SFAPFSIGLRACIGRNLAMQQLQIVTATIFRRYSIVLQDGAP-------------------LEIQD-SFG  537
CYP53D3__60352__Ppl        487  SFAPFSIGLRACIGRNLAMQQLQIVTATIFRRYSIVLQDDAP-------------------LEIQD-SFG  536
CYP53C__112429__Csu        497  AFVPFSVGPRACVGRNLAKMQLLVNIATVFRLYDIVLENPDLP------------------LPVHD-NFI  547
CYP53C__151209__Csu        497  AFIPFSVGPRACVGRNLAKIQLLINVATIFRCYDVVLEKPHDP------------------MPVHD-DFT  547
CYP53C__118598__Csu        514  AFIPFSVGPRACVGRNLAKMQLLINIATIFRLYKVVLADQDKE------------------LEVFD-NFV  564
CYP53H3__318972__Bad       480  AFAPFSVGPRACIGRNLALMELLIVVANVFYYFDAAPTSSDQP------------------MEIWD-GFL  530
CYP53H7__358536__Bad       489  AFAPFSLGPRACIGRNLAFMELLLVVANIFHRFEASSPYPGRE------------------AHIHD-GLP  539
CYP53H6__142452__Bad       484  AFAPFSVGPRACLGRNLALMELLLVVANVFHRFEVRLADPAQT------------------MEITD-GFL  534
CYP53H4__160054__Bad       482  AFAPFSTGPRACIGRNFAFMELLLVIANVFHRYEVVPGQSEPT------------------MEVRE-GFL  532
CYP53H2__55123__Bad        483  AFAPFSIGPRACIGRNLAFMELLLVVANIFHRFEAKIVSAEQV------------------MEVRD-GFL  533
CYP53H5__65034__Bad        480  AFAPFSIGPRACIARNLASMEMLLFVANVFHRYEAKLASQLQ-------------------MDIHD-SFT  529
Cyp53A__101387__Pst        446  YFMAFSSGTRGCLGKSLAILELKMIVATLLQRYDITPQSLV--------------------LQTTE-KLM  494
CYP53A__105834__Cpu        408  YLMTFGKGPRACLGRHLAYMEMRLVLSTVLLRYDLQLKSQV--------------------METTE-GFM  456
CYP53A__56813__Ade         448  HMLTFGMGPRACIGKNLAYVQMQLALATSLLRYEFLLSPKAQ-------------------LRSIE-GFM  497
CYP53__1116154__Fpi        441  EYIAFGHGKHACSGRWFAAAEVKAILAYILLNYDFKLEKPGGRP---------------ENMNLGP-SIL  494
CYP53NS__152212__Pca       432  SYFPFSYGPRKCMGEGLAMLEMSLTLATLFKRYDLELEPGFE-------------------MDFQPSFTL  482
CYP53NS__92916__Fme        426  SYFPFSYGPRKCLGEGLAILEMSLTLATLFKRYDLKLQEGFV-------------------MEFLPSFTL  476
Consensus aa:                   .@.sFShGPRtClGRsLA.hph.hhltolhpRYch.L.ps.....................hph...shh
Consensus ss:                                  hhhhhhhhhhhhhhhhhheeeee                             eee


Conservation:                          5    7
CYP53A15__ACF15219.1_Clu   486  RKPLALQVGMRKRSFA----------  501
CYP53C__1025718__Fpi_      523  RKPVECRVGIKRRTV-----------  537
Cyp53C__68781__Pst         525  RKPVECKVGIKRRSA-----------  539
CYP53C__80617__Gtr         522  RKPVECRVGIKRRN------------  535
CYP53C__127772__Cpu        546  RKPVECRVGIRRRV------------  559
CYP53C__94174__Shi         527  RKPVDCKIGIKRRNL-----------  541
CYP53c__27029__Wco         524  RKPVECKVGMKRRSL-----------  538
CYP53C3__110015__Ppl       523  RKPVACKVGLRRRSA-----------  537
CYP53C__116910__Csu        520  RKPVECRVGIKRRHD-----------  534
CYP53C__1179842__Sla       528  RKPQECRVGIKRRQT-----------  542
CYP53C__55106__Dsq         526  RKPVECKVGMKQRHT-----------  540
```

```
CYP53C4__GL08839-P1.1__Gl  528  RKPVECKVGMKRRNV-----------  542
CYP53C4__47512__Gsp        526  RKPVECKVGMKRRNA-----------  540
CYP53C__129211__Tve        526  RKPLKCIVGMKRRNV-----------  540
CYP53C9__27837__Pbr        524  RKPVDCVVGIRRRSL-----------  538
CYP53C7__118978__Bad       527  RKPVDCQVGIKKRAN-----------  541
CYP53C__183109__Pca        522  RKPVECLVGIKRRSL-----------  536
CYP53C2__130996__Pch       507  RKPVECVVGIRRRTL-----------  521
CYP53C__128292__Fme        535  RKPLRCRVGMRRREL-----------  549
Cyp53C__37267__Tve         524  RKPLKCNVGMQRRNV-----------  538
CYP53C__70450__Ade         514  RKPVLCRIGLKRRSPV----------  529
CYP53C__52716__Dsp         525  RKPVSCYVGMKRRST-----------  539
CYP53C__83844__Cpu         526  RKPTDCWVGLRRRAL-----------  540
CYP53C__194303__Abi        527  RKPLSVDLGIKRRDV-----------  541
CYP53C__194181__Abi        531  RKPLACRVGIKRRDI-----------  545
CYP53C__143663__Fme        535  RKPVACHVGLKRRGA-----------  549
CYP53C__130308__Fme        536  RKPMACRVGMKRRNV-----------  550
CYP53C__24265__Fme         534  RKPMACRVGMKRRNV-----------  548
CYP53C__149618__Fme        537  RKPVACRVGMKRRNI-----------  551
CYP53C__94457__Fme         537  RKPNSCRIGLKRRDV-----------  551
CYP53C__154594__Fme        530  RKPLTCRVGMKRRDV-----------  544
CYP53C__115179__Fme        540  MRPKSCRVGMKRRAI-----------  554
CYP53C1__5__Uma_           521  RKPVKLEVGLKRRN------------  534
CYP53B2__32280__Sro        520  RKPTGCQIGFKLRDATQ---------  536
CYP53B3__28617__Pgr        514  RKV---GFSLQQKT------------  524
CYP53C__101826__Pca        502  SKPKDVYVGMQRRV------------  515
CYP53C__256510__Pca        519  RKPNSVPVGMRRRV------------  532
CYP53C__102576__Pca        531  RKPKNVHVGMQRRL------------  544
CYP53C__212559__Pca        528  VLDKDVG-------------------  534
CYP53C__48859__Fpi         540  RRPLECMVGITRRKGELGYGSFEL--  563
CYP53C__86809__Fpi         540  RRPQECMVGITRRK------------  553
CYP53C__138909__Wco        542  RKPKRCMIGIKSRKL-----------  556
CYP53C__104840__Wco        544  RKPKRCVIGIKSRKL-----------  558
CYP53C__77097__Wco         548  RKPRWCAIGIKLRKL-----------  562
CYP53C__138864__Wco        542  RKPKWCMIGIKSRKL-----------  556
CYP53C__104855__Wco        551  RKPRRCMIGIRSRKL-----------  565
CYP53C__154264__Wco        499  YVAGHAAMGIEAVMLRQKEF------  518
CYP53c__138853__Wco        542  RRPKQCMVGIKPRNL-----------  556
CYP53C__154237__Wco             --------------------------
CYP53D1__108845__Ppl       555  RKPKNCVVGVQRRK------------  568
CYP53D6__54877__Ppl        555  RKPKNCFVGVQRRK------------  568
CYP53D4__55859__Ppl        526  QKSKECIIGVQRRKGLVSRQ------  545
CYP53D2v1__56013__Ppl      555  RKPMKCIVGVQRRK------------  568
CYP53D2v2__48082__Ppl      555  RKPMKCIVGVQRRK------------  568
CYP53D5__46728__Ppl        538  RKPKKCIIGVKRRDLKVL--------  555
CYP53D3__60352__Ppl        537  RKPKKCIIGVKRRDLKVL--------  554
CYP53C__112429__Csu        548  RKPLNCHVGVKRRS------------  561
CYP53C__151209__Csu        548  RKVIECFIGLKRREI-----------  562
CYP53C__118598__Csu        565  RKPLNCRVGMQRRDLSSKREWSI---  587
CYP53H3__318972__Bad       531  RQAFKCDVSLKSRGVA----------  546
CYP53H7__358536__Bad       540  SKPLSSVIALRRRSGV----------  555
CYP53H6__142452__Bad       535  RKPVSSVVALKRRVSA----------  550
```

```
CYP53H4__160054__Bad       533  RKPLASTVAFKRRGAAL---------  549
CYP53H2__55123__Bad        534  RQPLSSVVSVKRRGVF----------  549
CYP53H5__65034__Bad        530  RRPLASIVALKRRGL-----------  544
Cyp53A__101387__Pst        495  HKPTHDWVRLRRRNVD----------  510
CYP53A__105834__Cpu        457  HKPNEMFVRLSRRERKGAAVQAQAQA  482
CYP53A__56813__Ade         498  HKPVELWVGIRRRHQPTA--------  515
CYP53__1116154__Fpi        495  PHP-RAKVMFRKRKASRA--------  511
CYP53NS__152212__Pca       483  CSRNGLPVYARLRK------------  496
CYP53NS__92916__Fme        477  CSKNGLPVTARVRAQV----------  492
Consensus_aa:                   .+P..h.lshcpR.............
Consensus_ss:                   e    eeeeeee
```

Fig. S1D

Colored PROMALS3D alignment (sequences in aligned order)

```
Conservation:                           9
CYP53C__1025718__Fpi_       1  ----------MS-----------------------------SVVDQLTG--------LPVAAWA--GLVV   21
Cyp53C__68781__Pst          1  ----------MVL--------------------------------------------SDPLTLA--GLGL   14
CYP53C__80617__Gtr          1  ------------------------------------------MLSGILN--------ADVSNIL--ILLV   18
CYP53C__127772__Cpu         1  ----------M----------DSVIATLKDLVPINLNFDFDALLDRLRS--------ISPSQLA--AGVP   40
CYP53C__94174__Shi          1  ----------MLA----------------------------SLVNAVTN--------VDGKTCL--VAVP   22
CYP53c__27029__Wco          1  ----------M------------------------------SGLLAPLN--------LHPAALL--LLIP   20
CYP53C3__110015__Ppl        1  ----------M------------------------------SAPAALFT--------SNLVYGL--AVIP   20
CYP53C__116910__Csu         1  ----------M--------------------------------EFLHLS--------FDWTTAL--LVLA   18
CYP53C__1179842__Sla        1  ----------M------NTILQ-------------------LNPFQDFH--------FSFTTAL--AGVP   25
CYP53C__55106__Dsq          1  ----------M------------------------------SLVDRLLN--------SEPATWA--VVGF   20
CYP53C4__GL08839-P1.1__Gl   1  ----------M------------------------------SLLDPLLA--------FDFATWA--VIGL   20
CYP53C4__47512__Gsp         1  ----------M------------------------------SFLNPLLA--------LDFATWA--ALGL   20
CYP53C__129211__Tve         1  ----------M------------------------------ALLGILSS--------LDGPSWA--ALVF   20
CYP53C9__27837__Pbr         1  ----------MS-----------------------------SLLTSFSL--------DNVTNLL--LVIP   21
```

```
CYP53C7__118978__Bad          1  ----------M-------AVLD-------------------YVLSPLES--------LGLASSL--LIIP   24
CYP53C__183109__Pca           1  ----------M------------------------------AILEVLAQ--------LNLTSWL--VLIP   20
CYP53C2__130996__Pch          1  ----------M------------------------------AVIEALTQ--------LDLKSWL--LLIP   20
CYP53C__128292__Fme           1  ----------M-----GFTGVS-------------------TLTNALAR--------VDLISLA--VAVP   26
Cyp53C__37267__Tve            1  ----------M------------------------------AIFELLAS--------LDLPSWA--AIVL   20
CYP53C__70450__Ade            1  ----------M---------------------------------EDLLR--------LAV-YLA--LAGS   16
CYP53C__52716__Dsp            1  ----------M------SSLLD-------------------YFPTPFSS--------NALLLYG--GLAV   25
CYP53C__83844__Cpu            1  ----------M-------DVQQ-------------------LIQHLPQN--------VDLTSAA--AASF   24
CYP53C__194303__Abi           1  ----------M--------IIE-------------------TFKNFVAQ--------ADTTTLA--CAIP   23
CYP53C__194181__Abi           1  ----------M----ILDRLSD-------------------TFLELYDK--------LDLQIVA--FAVP   27
CYP53C__143663__Fme           1  ----------M-----ILIILR-------------------VFVDIGY-----------RIWLF--LPWI   23
CYP53C__162664__Fme           1  MTAVILSQSTM-----LPNILG-------------------ITVHSVF-----------LFFTL--LGVL   33
CYP53C__154594__Fme           1  --------------------MQ-------------------MISNISP-----------WYFCL--LPAA   18
CYP53C__149618__Fme           1  ----------MSM---SSGIVQ-------------------ELLNVTF-----------WLWFF--LLAI   25
CYP53C__130308__Fme           1  ----------M-----IASIFY-------------------STFFTKYL----------WICAG--LTAG   24
CYP53C__24265__Fme            1  ----------M-----ILKILY-------------------SVFFTNYL----------WLCTG--LTAA   24
CYP53C__94457__Fme            1  ----------M-----VPNIAH-------------------NFL-LGYS----------WILLL--LPAI   23
CYP53C__115179__Fme           1  ----------MLP----------------------------NILDALAQ--------LNTSQLCASALAV   24
CYP53C1__5__Uma_              1  ----------MVETDLVPRIGA-------------------AIQWSV----------ESPAHV---VITL   28
CYP53C__48859__Fpi            1  ----------MS----IVQQLT-------------------NFTRG-----------NPLLVL---AALP   23
CYP53C__86809__Fpi            1  ----------MS----IVQQLI-------------------DFTRG-----------NPLLVL---AALP   23
CYP53C__138909__Wco           1  ----------MSLLRDITVQAL-------------------PFVD------------KLIFWL---ALIP   26
CYP53C__104840__Wco           1  ----------MPLIGDITVQTL-------------------PFVG------------ISTIWL---ALIS   26
CYP53C__77097__Wco            1  ----------MSLVDDTTAHVP-------------------SY---------------PAFWI---ALIA   23
CYP53C__138864__Wco           1  ----------MSLLRDITVQAL-------------------SFVD------------KLIFWL---ALIP   26
CYP53C__104855__Wco           1  ----------MSLLGDIAPQTL-------------------DYVG------------KLTFWL---ASIP   26
CYP53C__154264__Wco           1  ----------MPLLASITAYAL-------------------DYMC------------MPTFWL---ALIP   26
CYP53c__138853__Wco           1  ----------MPLLDRITTHAL-------------------DYVC------------MPTFWL---ALIP   26
CYP53C__154237__Wco           1  ----------M-----------------------------------------------------------    1
CYP53C__112429__Csu           1  ----------M-----ILDVVH-------------------SVLERP----------L--LLV---VLLP   21
CYP53C__151209__Csu           1  ----------MLPAE-LVDCLP-------------------GS---------------LPLLF---GLFL   22
CYP53C__118598__Csu           1  ----------MDWRM-MIDGFT-------------------TLTSRETYTFSGFVSILLPLLF---LCLS   37
CYP53C__101826__Pca              ----------------------------------------------------------------------
CYP53C__256510__Pca           1  ----------MEQVRSLLP----------------------QLD--------------LKLAS---VLLV   21
CYP53C__212559__Pca           1  ----------MEQTHFLLP----------------------QLD--------------LRLTA---GLLA   21
CYP53C__102576__Pca           1  ----------MEQSHFILP----------------------QLD--------------LKLIT---AILA   21
CYP53C__212558__Pca           1  ----------MEQYHFILP----------------------QID--------------LKSIA---VIRA   21
CYP53__1116154__Fpi           1  ----------MPS---LPLG---------------------LKIPQ-----------LSALEIC--G---   20
Consensus_aa:                    ..........M...............................h.................h.h....hh.
Consensus_ss:                                    hhhhhh                   hhhhhh            hhhh   hhhh


Conservation:                                                99     8 55  95      5  6  7   8 5 8
CYP53C__1025718__Fpi_        22  A--AVVLVHLVPLIT--DPYQVRSY--PGPFL--AKISDAWLGWVAAQGHRSEVVHELHQKHG-------   76
Cyp53C__68781__Pst           15  A--AVVVAHIGAYLL--DPHNIRDI--PGPTL--AKFSDAWLGWVAAKGHRSEVVHEMHAQYG-------   69
CYP53C__80617__Gtr           19  A--VVAVAHIVPYFT--DPHAIKSY--PGPWL--AKFSDAWLGKVSAQGHRSEVVHDLHKKYG-------   73
CYP53C__127772__Cpu          41  V--CLLLYFLVPWLW--DPYHQRSI--PGPFL--AKFSNAWLGWVSAHGHRSEIVHELHKKYG-------   95
CYP53C__94174__Shi           23  V--LLVLAHVIPWLV--DVHGIRSY--PGPFW--ARFTDLWLGWVAAQGHRSEVVHEMHNKYG-------   77
CYP53c__27029__Wco           21  A--AVLAVHFVPYLL--DPHGIRSY--PGPFL--AKLSDAWLGWVAAKGHRSEVVHQLHQRYG-------   75
CYP53C3__110015__Ppl         21  V--AVLLVHFVPYLL--DPHGIRAY--PGPFL--ARLSDIWLGWIAAQGHRSETVHELHKQYG-------   75
CYP53C__116910__Csu          19  V--GVALVHVVPWLL--DPHGIRSY--PGPLL--AKFSDAWLGWVAAQGHRSEVVHELHKKYG-------   73
```

```
CYP53C__1179842__Sla        26  I--IFILVHVFPYLA--DPFKQRAI--PGPLL--AKFSDAWLGWVSSQGHRSEVVHKMHLKYG-------  80
CYP53C__55106__Dsq          21  T--AVLLIHFVPYLA--DPHHIREY--PGPLL--AKLSDIWLGYVAAQGHRSERVHELHKQYG-------  75
CYP53C4__GL08839-P1.1__Gl   21  S--AIVLFHLVPYLV--DSHHIRGY--PGPLL--AKFSDVWLGYVAAQGHRSEQVHELHKQYG-------  75
CYP53C4__47512__Gsp         21  A--AIVLFHLVPYLV--DSHHIRGY--PGPLL--AKFSDVWLGYVAAQGHRSEKVHELHEQYG-------  75
CYP53C__129211__Tve         21  A--AVVLVHVVPYLL--DPHGFRSY--PGPFL--AKLSDFWLGKVAADGHRSERVHELHEIYGN------  76
CYP53C9__27837__Pbr         22  G--LLVLGHVVFYLV--DPYKIRSY--PGPLL--ARFSDLWLGRVAAEGHRSEIVHKLHQKYG-------  76
CYP53C7__118978__Bad        25  C--LVLAGHFIYFVI--DPHRIRSY--PGPLL--AKLTDAWLGYVAAHGHRSEVVHGLHQKYG-------  79
CYP53C__183109__Pca         21  A--LAIAAHVVHWLL--DPHGIRSY--PGPLL--ARFSDAWLGYVAAQGHRSEVVHDLHKKHG-------  75
CYP53C2__130996__Pch        21  A--LAIVAHILIWLL--DPHGIRSY--PGPLL--AKFSDAWLGYVAAQGHRSEVVHDLHKQYG-------  75
CYP53C__128292__Fme         27  L--VLILGLIVPYFV--DPHCIRNNGITGPLS--ARFSDAWLGWVAAQGHRSEVVHEMHKKYG-------  83
Cyp53C__37267__Tve          21  A--AVVLVHLVPYVL--DPHGIRAY--PGPFW--AKLTDLWLGKIAADGHRSERVHDLHKKYG-------  75
CYP53C__70450__Ade          17  I--GTTLFVLVPYFK--DEHGVRDI--PGPLA--AHLSNLWLAYWSSQGKRSEMVHEQHLKCG-------  71
CYP53C__52716__Dsp          26  L--LLVASHLVPWVA--DPFGYRKKHIPGPFL--AQLSDVWLARVAAQGHRSEIIHGLHQKYG-------  82
CYP53C__83844__Cpu          25  A--AFLAVHLGPYAW--DRYHLKSI--PGPFW--AKFSDAWLAWVAANGHRSEEVHKLHEKLG-------  79
CYP53C__194303__Abi         24  A--AVVLFHVIPWLT--DSHSLRKY--PGPFF--AKFSDFWLAFTSRGGRRSEIIHDYHKKFG-------  78
CYP53C__194181__Abi         28  G--TFLAFHLLPWLW--DPHGLRAY--PGPFI--AKFSDIWLTCVSKGAHRSELVHEAHLKYG-------  82
CYP53C__143663__Fme         24  F--LVVHLIPYLVDR--YHIRRNGI--TGPSL--ARFSDAWLGWVVANGRQSEVVHEMHKKFG-------  78
CYP53C__162664__Fme         34  V--LVVHLVPYFIDP--YHIRSNDI--KGPSL--AGFSGTWLGWVAVSGHQSIVVHQLHKKFG-------  88
CYP53C__154594__Fme         19  V--LVLLVVPYFIDP--YWIRRNEV--RGPFL--ASLTSLWFGWNATRGHLSQVVHDLHKKFG-------  73
CYP53C__149618__Fme         26  I--FAVHLDAYFIDS--HRIRRNGI--SGPFL--ARFSGAWLGWVVFQGRQSEVVHSLHKKFG-------  80
CYP53C__130308__Fme         25  F--LVVHVVPYLLDK--HHIRRNGI--TGPFL--AMFSDAWLGWVAAQGRRAEVVHEKHKKYG-------  79
CYP53C__24265__Fme          25  F--FLVHVVPYLFDK--HHIRRNGI--SGPLL--ARFSDAWLGWVAAQGPRSEVVHQIHKKYG-------  79
CYP53C__94457__Fme          24  I--LVVHLVSYFVDS--KHIRRNDI--PGPTL--AKVSGSWLGRVALEGRQSEVVHELHKKFG-------  78
CYP53C__115179__Fme         25  A--AVV--YLQPYLVDSHFIRRNGI--TGPFF--ARFSDAWLGWVAAHGNRSVVVHKLHKKYG-------  79
CYP53C1__5__Uma_            29  L--GAVVLFHVVPYI--TNTACIKY--PGPFF--AKFTDFWLLRTALIGHRFEEVHKQHQKYG-------  83
CYP53C__48859__Fpi          24  V--VLVVAKVVHYLV--DSSDLRSY--PGPFL--AKFTDAWIFWTVSRNRWSRSVEDAHIKYG-------  78
CYP53C__86809__Fpi          24  V--VLVVAKVVHYLA--DSSDLRSY--PGPFL--AKFTDAWIFWTVSSNRWSHSVEDAHIKYG-------  78
CYP53C__138909__Wco         27  I--ISIAVYTVPYLL--DPLGLRAF--PGPIL--AKFTIGWLPWIVSQNRWSLTVNRLHQKYG-------  81
CYP53C__104840__Wco         27  ITGIVTVICVVPYLL--DSLGLRAF--PGPTL--AKFASGWLPWVISQNRWSVTVGRLHEKYG-------  83
CYP53C__77097__Wco          24  I--TVFAVQFVPYLL--DPLGLSSF--PGPVL--AKFSNVWLPWIVSQNRWSVTVDQLHRKYGKTLIYCG  85
CYP53C__138864__Wco         27  I--IGIAMYIVPYFL--DPLGLRAF--PGPIL--AKFTIGWLPWVVSQNRWSLTVNRLHQKYG-------  81
CYP53C__104855__Wco         27  I--VIIAAHIVSYLL--DPLGLRAF--PGPIF--ARFTSGWLPWIISQNRWSVTVDRLHQKYG-------  81
CYP53C__154264__Wco         27  A--VLTTVYIVPYLL--DPLGLRTF--PGPIF--AKFTSGWLPWVISQNRWSAIVDSLHRKDG-------  81
CYP53c__138853__Wco         27  A--VLTTVYIVPYLL--DPLGLRTF--PGPIF--AKFTSGWLPWVVSQNRWSVAVDSLHRKYG-------  81
CYP53C__154237__Wco          2  -------------------------------------------------------------YG-------   3
CYP53C__112429__Csu         22  S--VLLAVALVAHLR--DPHHLRSY--PGPFL--ASLTDLWLAYKVWVGDRSPGVHELHKKHG-------  76
CYP53C__151209__Csu         23  T--ALLAVFFAPYIL--DRHRLRSY--PGPFL--ARFSDLWLASQVWKSHRSEEVHRLHKKYGK------  78
CYP53C__118598__Csu         38  I--TLLLFPCVTYLL--DFYGLRSY--PGPFL--AKFTDLWLAYKVWEGNRSPDIHLLHKKHG-------  92
CYP53C__101826__Pca          1  ---------MIPFLV--DKYGLRGY--PGPLV--AKFSSLWLASKAHKGKTTSAVHALHQKYG-------  48
CYP53C__256510__Pca         22  A--APLFAWAVPFLV--DKHGLMAF--PGPLL--AKFSSLWFALKAYKGTTSLTVHALHERYG-------  76
CYP53C__212559__Pca         22  A--ALLAAWVVPFLI--DRYRLKGI--PGPLL--AKFSCVWLASKAYKGTMSSAVHVLHEKYG-------  76
CYP53C__102576__Pca         22  A--SLLAAWIIPFLV--DKYRLRGY--PGPLL--AKFSGFWLASKAYKGTTTSAVYALHQKYE-------  76
CYP53C__212558__Pca         22  A--ALLAAWVIPFFV--DKYGLRGY--PGPLL--AEFSGFWLASQAYKGTTTSAVHALHQKYG-------  76
CYP53__1116154__Fpi         21  ---IVTALFLVSYLR-RRSDPIHAIPTVGPSWPLLSYLGAWRYFRDAKG----MILEGCSKY--------  74
Consensus_aa:                   h..hlhhhhhh.@h...c.h.hpsh..sGPhh..Ap@oshWls..s.ptp.S..Vc.hHpK@G.......
Consensus_ss:                   h  hhhhhhhhhhh                     h  hhhhhh    hhhhhhhhhhhh


Conservation:                      5567576   6686   6 86   6855   5 5 85   85 5          7  556   755776 57
CYP53C__1025718__Fpi_       77  --KFVQIAPN---HVSVSDPDALQVIYAHGNGTL-KSTFYDAFVSI---QRGLFNTRSRPEHARKRKIVS  137
Cyp53C__68781__Pst          70  --PVVRIAPN---HVSIAEPQALQIVYAHGNGSL-KSNFYDAFVSI---QRGLFNTRNRADHARKRKIVS  130
```

```
CYP53C__80617__Gtr          74  --TFVRLAPN---HLSIADPEALQTVYAHGNGSL-KSDFYDAFVSI---RRGLFNTRDRAEHARKRKIVS  134
CYP53C__127772__Cpu         96  --PVVRIAPN---HVSVADPEALQVVYAHGNGSL-KSDFYDAFVSI---HRGLFNTRDRQQHARKRKIVS  156
CYP53C__94174__Shi          78  --PIVRIAPN---HISISDPEALQIVYAHGNGSL-KSNFYDAFVSI---HRGLFNTRDRAAHARKRKIVS  138
CYP53c__27029__Wco          76  --TFVRIAPN---HVSISDPDALSEVYAHGNGTM-KSNFYDAFVSI---QRGLFNTRSRPEHARKRKIVS  136
CYP53C3__110015__Ppl        76  --TFVRIAPN---HVSISDPEAIQYVYAHGNGTT-KSNFYDAFVSI---RRGLFNTRSRPEHARKRKIVS  136
CYP53C__116910__Csu         74  --TFVRLAPN---HVSISDPDAIQIVYAHGNGSL-KSNFYDAFVSI---QRGLFNTRSRPEHARKRKIVS  134
CYP53C__1179842__Sla        81  --TFVRIAPN---HVSVADPDALQVVYAHGNGSL-KANFYDAFVSI---QRGLFNTRNRNEHARKRKIVS  141
CYP53C__55106__Dsq          76  --TFVRIAPN---HLSISDPEALQVVYGHGTGTL-KSDFYDAFVSI---QRGLFNTRSRVQHARKRKIVS  136
CYP53C4__GL08839-P1.1__Gl   76  --TFVRIAPN---HLSISDPDALQVVYGHGTGTL-KSTFYDAFVSI---QRGLFNTRSRVQHARKRKIVS  136
CYP53C4__47512__Gsp         76  --TFVRIAPN---HLSISDPDALQIVYGHGTGTL-KSTFYDAFVSI---QRGLFNTRSRVQHARKRKIVS  136
CYP53C__129211__Tve         77  -WTFVRIAPN---HLSIADPDALQIVYGHGTGTL-KSDFYDAFVSI---QRGLFNTRSRTDHARKRKIVS  138
CYP53C9__27837__Pbr         77  --TFVRLAPN---HVSVSDPDALQVVYAHGNGTL-KANFYDAFVSI---QRGLFNTRSRPEHARKRKIVS  137
CYP53C7__118978__Bad        80  --KFVRIAPN---HVSIADPDALPIVYGHGNGTL-KSNFYDAFVSI---ERGLFNTRSRHEHARKRKIVS  140
CYP53C__183109__Pca         76  --TFVRLAPN---HVSISDPDALQVVYGHGTGTL-KSDFYDAFVSI---QRGLFNTRSRPEHARKRKIVS  136
CYP53C2__130996__Pch        76  --TFVRIAPN---HLSIADPDALQVVYGHGTGTL-KSNFYDAFVSI---QRGLFNTRSRSEHARKRKIVS  136
CYP53C__128292__Fme         84  --TFVRLAPN---HVSISDPAALQIVYAHGNGTL-KSSFYDAFVSI---RRGLFNTRSRPEHTRKRKIVS  144
Cyp53C__37267__Tve          76  --PFVRIAPN---HLSISDPDALPVVYGHGTGTL-KSDFYDAFVSV---QRGLFSTRSRPEHTRKRKIVA  136
CYP53C__70450__Ade          72  --KLVRIAPN---HISVNDPDALPIVYGHGTGTL-KSEFYDAFVSI---QRGLFNTRSRTQHTRKRKIVS  132
CYP53C__52716__Dsp          83  --KVVRIAPN---HISLSEPGALQIVYAHGNGAL-KSDFYEAFVSI---RKNIFSTRDRAEHTRKRKIVS  143
CYP53C__83844__Cpu          80  --PVVRIAPN---HISIADPDALQIIYAHGSNTLKKSNFYDAFVSI---RRAIFNTREKADHARKRKIVA  141
CYP53C__194303__Abi         79  --PVVRIAPN---HVSISDPDALNAVYGHGTGTL-KSEFYDAFVAM---DRGLFNVRDRHDHTRKRKVIS  139
CYP53C__194181__Abi         83  --PVVRIAPN---HLSIANPEALQIVYAHGNGAL-KSIFYDAFVSI---RRGLFNVRDRNEHTRKRKIVS  143
CYP53C__143663__Fme         79  --PVVRLAPN---HVSISDPGALHVIYGHGSGLL-KSGYYEPFTAV---RPSIFSTRSREVHSKKRKIIS  139
CYP53C__162664__Fme         89  --TFLRLAPN---HVSISDVEALQAVYGQGSGTL-KSNYYDAFVAF---RPSIFETRSKAEHSRKRKAIA  149
CYP53C__154594__Fme         74  --TFVRLSPN---HVSISDPDALQTIYGHGKGLM-KSDYYDAFKGL---RPSIFSTRDRAFHAWKRKAIS  134
CYP53C__149618__Fme         81  --TFVRLSPN---HVSISDPDALRLVYGRGNGAL-KSDYYDAFLAV---RPSIFTTRSKEEHARKRTAIA  141
CYP53C__130308__Fme         80  --KFVRLAPN---HVSIADPEAIGDIYGHGNGTL-KTDFYDAFISI---GVTVFTTRSREEHTRKRKVIS  140
CYP53C__24265__Fme          80  --KFVRLAPN---HVSIAYPEAIGEIYGHGNGTL-KTDFYDAFLSI---DRTIFTTRSREEHTRKRKVIA  140
CYP53C__94457__Fme          79  --TFVRLSPN---HVSISDPEALQVVYGHGNGML-KSEYYDAFAAP-NLRRSVFDTRSREEHARKRKAIS  141
CYP53C__115179__Fme         80  --LFVRLAPN---HVSISDPEALHIVYGHGSGTL-KSDYYDAFLAI---RHTVLTTRDREDHSMKRKLVA  140
CYP53C1__5__Uma_            84  --KFVRIAPN---HVSIADHEALQPIYGHGTGTL-KPAYYDAFVPPRPFPRGLFNTRDRAEHTRKRKIVS  147
CYP53C__48859__Fpi          79  --PIVRIAPN---HISIDDPKALAMVYGHSTGFM-KSNWYDIFAAF--SVSNIFDTRSRSEHARKRRMEA  140
CYP53C__86809__Fpi          79  --PIVRIAPN---HISIADPKALATVYGHSTGFT-KANWYNAFSEF--AAKNIFNTRSRSEHARKRRMEA  140
CYP53C__138909__Wco         82  --IFVRLSPN---YVSIAHPAALSAVYGHSSGAT-KAPYYEVFGDF--RARNLFNILSRPEHARRRRLEA  143
CYP53C__104840__Wco         84  --TFVRLAPN---HVSIAHPAALSAVYGHSSGAL-KAPFYDASGNF--KARNMFNTRSRSEHARKRRSES  145
CYP53C__77097__Wco          86  AGTFVRLAPN---HVSIAHPAALPAVYGHSSGTL-KAPLYDVFGPF--RARSIFSTRSRTEHARKRRIES  149
CYP53C__138864__Wco         82  --IFVRLSPN---HVSIAHPAALSAVYGYSSGVT-KAPYYDVFGDF--RAKNLFNIISRTEHTRKRRIVS  143
CYP53C__104855__Wco         82  --TFVRLSPN---HVSIAHPAALPAVYGHGSGAP-KAPYYDGFVNF--KSRNMFNTLSRSEHARKRRIES  143
CYP53C__154264__Wco         82  --IFVRLSPN---HISIAHPVALPAVYGHSSGAL-KAPFYDAFASF--KTRNMFNTLSRTEHTRKRRIES  143
CYP53c__138853__Wco         82  --IFVRLSPN---YVSIAHPVALPAVYGHSSGAL-KAPFYDALSGF--KTRNMFNTLSRTEHARKRRIES  143
CYP53C__154237__Wco          4  --IFVCLSPN---HISIVHPVALPAVYGHSSGAL-KAPFYDAFASF--KTRNMFNTISRTGHTRKRRIES   65
CYP53C__112429__Csu         77  --TFLRIGPN---HISIASPAALGVIYSHSHPLL-KSDFYDGLATF--SAPGTFTVRDRVAHARKRRVVA  138
CYP53C__151209__Csu         79  -CRFLRIGPN---HVSVADPAAIPILYSHSNPLM-KSDFYDGFTTF--RTPGIFVERDRVAHARKRRVVS  141
CYP53C__118598__Csu         93  --PFMRIGPN---HISVVSPAAISTIYSHIDPLP-KSAFYDGLATF--SVPDIFTTRDRVTHGRKQRMVS  154
CYP53C__101826__Pca         49  --PFVRISSK---HVSIADPEALQAIYGHNSGAL-KTDFYDAFVAF---RHNIFTSRSRLEHSRKRKYTA  109
CYP53C__256510__Pca         77  --PFVRLSPQ---HVSIADPEALRAIYGHSSGTL-KTELYDAFVTF--------------FLARKRKYTA  126
CYP53C__212559__Pca         77  --PFVRISPK---YVSIADPEALQAIYGYSSGAL-KTELYDAFVFF---RPTMFSTRSRLEHSRKRKYTA  137
CYP53C__102576__Pca         77  -GPFVRISPK---YVSIADPEALQAIYGHTSGTL-KTDFYDAFVTF---RRNIFTSRSRLEHSRKRKYTS  138
CYP53C__212558__Pca             ----------------------------------------------------------------------
CYP53__1116154__Fpi         75  --EVFKIPLSDQWLVVVSGRDMNDELRKYPDDTM-SALEAQKWVV-----QTEYTLGNNNPDATAKKCIS  136
Consensus aa:                   ...hlRltPs...HlSltpP.Al.hlYt+tsshh.Kt.@Y-s@hsh.....shFsh.sR..HhRKR+hht
```

```
Consensus_ss:                      eeee      eeee  hhhhhhhhh           hhhhh        eee    hhhhhhhhhh

Conservation:                     66 7 7  685        5  566 5   5  5  5           9    6 56 6 666  55
CYP53C__1025718__Fpi_      138  HIFSQKSVLEFEPYTRMHIKKLMNQWDRLYDLAMKGGSGEEGE-G-----WQGRDGRLWLDILPW-YNYL  200
Cyp53C__68781__Pst         131  AIFSMKNVLEFEPHVREYVGLLIKQWDRLCAEAVKGGSGDEGEGG-----WRGESGRLWLDCLPW-YNYL  194
CYP53C__80617__Gtr         135  HIFSQKSVLEFEPHVRLYVRQFIEQWDRLCGLAAKGERGEEGNG------WEGREGRLWLDCLPW-YNYL  197
CYP53C__127772__Cpu        157  GIFSQKNVLEFEPHVRLYVGQLMEQWDRLCARAEKGESGDEGEGG-----WQGRGGKLWLDCLPW-YNYL  220
CYP53C__94174__Shi         139  HIFSQKNVLEFEPHVREYVKSLIAQWDRLYDLAVNGESGTEGEGG-----WVGREGRLWLDCLPW-YNYL  202
CYP53c__27029__Wco         137  HIFSQKSVLEFEPYTRQHVGALFKQWDRMCELGTKGLFGEEGEGG-----WHGRDGRVWFDCLPW-YNYL  200
CYP53C3__110015__Ppl       137  HIFSMKSVMEFEPYTRMHVAQLLKQWDRLYELGIKGASGEEGEG------WKGRDGRVWLDCLPW-YNYL  199
CYP53C__116910__Csu        135  HIFSQKNVLEFEPHVREHIRTLISQWDRLYELGKKGLSGTEGEGG-----WQGKNGRVWLDCLPW-WNYL  198
CYP53C__1179842__Sla       142  HIFSQKNVLEFEPNVRLYVGQLISQWDRLYDSAAKGASGTEGEGG-----WFGKDGRLWLDSLPW-YNYL  205
CYP53C__55106__Dsq         137  NIFAQKNVLDFEPHVRQHLANLFRQWDKLCEGGKNGLSGDEGEGG-----WQGRDGRVWYDCLPW-YNYL  200
CYP53C4__GL08839-P1.1__Gl  137  NIFAQKNVLEFEPHVREHLGTLFQQWDKLCDGGKKGLSGTEGEGG-----WHGGEGRVWYDCLPW-YNYL  200
CYP53C4__47512__Gsp        137  NIFAQKNVLEFEPHVRQHLGTLFQQWDKLCDGGKKGLSGTEGEGG-----WHGSDGRVWYDCLPW-YNYL  200
CYP53C__129211__Tve        139  HIFSQKNVLEFEPHVRVHLIQLFKQWDRLCAGGARGEAGDEGEGG-----WRGRDGRVWYDCLPW-YNYL  202
CYP53C9__27837__Pbr        138  HIFSQKSVLEFEPNTRLYVRQLIAQWDRLCELGAKGLSGDEGEGG-----WKGRNGRVWLDCLPW-YNYL  201
CYP53C7__118978__Bad       141  HIFSQKSVLEFEPNVRTYVGQLIGKWDRLYENGAKGLSGDEGEGG-----WTGRNGRVWLDCLPW-YNYL  204
CYP53C__183109__Pca        137  HIFSQKSVLEFEPHVRLYVNQLIRQWDRLYEAGAKGLSGDDGESG-----WTGRNGRVWLDCLPW-YNYL  200
CYP53C2__130996__Pch       137  HIFSQKSVLEFEPHVRLYVKQLIQQWDRLYEAGAKGL--------------------VWLDCLPW-YNYL  185
CYP53C__128292__Fme        145  HIFSQKSVLEFEPHIRLHVGELFTQWDKLCDGGKRGLKGTEGDG------WEGHDGWVWFDCLPW-FNYL  207
Cyp53C__37267__Tve         137  HSFSQKSVHEFEPHVRENLSKLFKQWDTLCEGGAKGLSGNEGEGG-----WQGREGRVWYDCLPW-YNYL  200
CYP53C__70450__Ade         133  HVFSQKNVLGFEPNLHSALSRFVSQWDRMCAAGVKGGRGNE-QDG-----WHGDGQRVWMDCLPCAYNYL  196
CYP53C__52716__Dsp         144  HIFSQKSVLEFEPYIRQALGKLVKQWDSLLSDDRKLASHRLR---------PNENGTAWFDCLNW-YNYL  203
CYP53C__83844__Cpu         142  NTFSQKNVIEFEPRVRIYVGQIIDQWDRLSKLAAADGSGDEGESG-----WYGKDERLWLDVLPW-MNYL  205
CYP53C__194303__Abi        140  HIFAQKSVVAFEPKIAIYVTQLLNQWDRLYDMAVKGGSGNEGEGG-----WKGKDGKLYLDILPW-MNYL  203
CYP53C__194181__Abi        144  HIFSQKNVLEFEPHIRMYVAQLQNQWDRLYDMAVKGMSGNDGEGG-----WEGRDGRLWLDCLPW-ANYL  207
CYP53C__143663__Fme        140  HVFSQKSVLEFEPFVHLHLAELFEHWDKMCDGGKEGLSGTESEGG-----WKRRGGQAWFDIMPW-FNYL  203
CYP53C__162664__Fme        150  HVFSQKSVAEFEPFVRLHLAELFEQWDKMCDGGKKGISGAEGEDG-----WNGHDGRVWFNVAPW-FNYL  213
CYP53C__154594__Fme        135  HIFSPKSVIDFEPYIHLHLTELFEQWDKLYDGGKRGLSGVEGEG------WNGRQGRVWFNIMPW-FNYL  197
CYP53C__149618__Fme        142  HAFSQKSVLEFEPYIRLHVAELFNQWDRMCNRGKNGLSGTEGEGG-----WIGQGGRVWFDIMPW-FHYL  205
CYP53C__130308__Fme        141  HGFSQKSVSEFEPYIRLHVSELFEQWDELYDGGRKGLTGVEGEGG-----WKGHDGRVWFNAMPW-CNYL  204
CYP53C__24265__Fme         141  HGFSQKSISQLEPYIRLHVAELFEKWDKLYDGGRKGLTGVEGHNS-----WEGHDGRVWFNAMPW-LNYL  204
CYP53C__94457__Fme         142  HIFSQKSVLEFEPYIHTHLTDFFKQWDKLCDGGRKGFSGIEGEGG-----WKGHDGRVWFNAMPW-YNYL  205
CYP53C__115179__Fme        141  PIFSQKSVLGFEPCVHSHVTELFEQWDKLCDGGKQGLTGNAGKGG-----WKGRDGRVWFDALPW-LYYM  204
CYP53C1__5__Uma_           148  HTFAPKTIVAFEPFIRREVQLLLERWDEFCDKATKDNTEGP----------RGIKGRAWLDSLMW-LNYF  206
CYP53C__48859__Fpi         141  HMFAPQSIRAVEPISHSHVNELLRQWDGLISRVAKAQQGGPNGGHIGATTWNVQDGRVWIDCMPW-LNFW  209
CYP53C__86809__Fpi         141  HMFAPQSIRAVEPISHAHVNELLRQWDGLISRVAKAQQGGPNGGHIGATTWNVQDGRVWIDCMPW-LNFW  209
CYP53C__138909__Wco        144  HMFSLQSVRALEINARVHHNNLVNQWERLYSYVKQAEKGGIREDKLGASAWRVEDGRVWFDCMPW-FSYW  212
CYP53C__104840__Wco        146  HMFSPQSVRALEGTARVHHGNFVNQWGKLYSYVAQAKSGEAKEGKLGACAWKVEDGRVWFNCMPW-FNYW  214
CYP53C__77097__Wco         150  PMFSPQSVRALEGTTRVHHSDLASQWDNLYSYVKQAENGGSREGMLGESAWKVEDGRVWFDCMPW-FNYW  218
CYP53C__138864__Wco        144  PMFSQQSVRALEINARVHHNNLVSQWERLYSYVKQAEKGGIREDKLGASAWRVEDGRVWFDCMPW-FSYW  212
CYP53C__104855__Wco        144  HMFSPQSVRALEGTASVHHGNLVSQWDKLYSYVKQAEGGGAKEGQLGASVWKVEGGRVWFDCMPC-----  208
CYP53C__154264__Wco        144  QIFSQQSVRELEGTARVHHIDLVSQWDKLYSYVKRAESGAAREG----------------------FNYW  191
CYP53c__138853__Wco        144  HMFSPQSVRALEGIARMHHNDLVSQWDKLYSYVKRAESGAAREGSLGECAWKVKDGRVWLDCMPW-FNYW  212
CYP53C__154237__Wco         66  QIFSQQSVRELEGTTRVHHIDLVSQWDKLYSYVKRAESDGVREG----------------------FNYW  113
CYP53C__112429__Csu        139  HLFAPKTVRMFEGALHKYIGQLVQQWDGMYKNVETALPGTATAGKAGDMSWIVRDGRVWFDCMPW-LNFL  207
CYP53C__151209__Csu        142  HLFAPKTVKAFEPAVQNYVGQLVRQWDRLCKN-ADAQSVSVMSGVLGSMTWRAYDGCVWFDCMPW-LNFL  209
CYP53C__118598__Csu        155  HLFAPKTVRLFEEDVQKYVGQLVAQWDDMC---ARAKDGVVLTGHNGAMEWSTREGKVWFDCMPW-FNLL  220
CYP53C__101826__Pca        110  YAMSMKGIMEFEPNVREYQHMLVRQLDTLCAVGAQGIDGVLGSCP-----WTARDGWVLFDCMPW-LNFA  173
```

```
CYP53C__256510__Pca        127  HAMSVKGIMQFEPNVREHQQMLVKRLDTLCTVGAQGVDGVLGSCP-----WAARDGWVWFDCMPW-FNFE  190
CYP53C__212559__Pca        138  HAMSMKYIMEFEPNVREHHHMLVKQLDTLCAAGAQGKDGILGTRP-----WTARDGWAWFDCMPW-FNYE  201
CYP53C__102576__Pca        139  HAMSMKGITEFEPNVREYQHMLLKQLDTLCAVGAQGIDGVLGSCP-----WTTRDGWVLFDCMPW-LNFD  202
CYP53C__212558__Pca         77  ------------------------------------------------------------------LNFG   80
CYP53__1116154__Fpi        137  GPLTHK----LGHVLPDVVDEMIHSFNDIMPDAEH-------------------DWQTVPALET-MIKI  181
Consensus_aa:                   .hht.psl..hEs.hp.hh.plhpp@Dclhs.s.pt.sGs..pt......WpscsG.h@hsthPW.hN@h
Consensus_ss:                   hhh hhhhhhhhhhhhhhhhhhhhhhhhhhhhhh hhhhhhhhhh              eehhhh hhhh


Conservation:                    8866678768 59796    8 6  5                                  55
CYP53C__1025718__Fpi_      201  AFDIIGDLAFGAPFGMLDACADAAPVAISHEKAMSSYGETDT------------PEITYFPAVQILNDRG  258
Cyp53C__68781__Pst         195  AFDIIGDLAFGQSFGMLHACKDSAPVALSQDEAMKAYGSASG------------YKVVSIPAVQILNDRG  252
CYP53C__80617__Gtr         198  AFDIIGDLAFGSPFGMLKACKDSAPVAVSHADAMAAYGKDDS-----------AVQVRSLPAVQILNDRG  256
CYP53C__127772__Cpu        221  AFDIIGDLAFGSPFGMIHSAKDSAPVAVSHADAMSAYGSSAS-----------NIKVVHIPAVQILNDRG  279
CYP53C__94174__Shi         203  AFDIIGDLAFGSPFGMLQAAKDSAPVAKSAKDAIAAYGQDEA-----------KVEVVHIPAVQILNDRG  261
CYP53c__27029__Wco         201  AFDIIGDLAFGAPFGMLQACADAAPVVVSHADAMASYGKGDA------------PEVAYFPAVQVLNDRG  258
CYP53C3__110015__Ppl       200  AFDIIGDLAFGAPFGMLHACADAAPVATEHKDAMASYGADNA------------PKVTYFPAVQVLNDRG  257
CYP53C__116910__Csu        199  AFDIIGDLAFGAPFGMLHACADSAPVAISHEAAMKNYGDDAA------------PEVEHFPAVQVLNSRG  256
CYP53C__1179842__Sla       206  AFDIIGDLAFGSPFGMILNAKDSAPVAVSQKDAMKSYGSEST------------YEVIEIPAVQILNDRG  263
CYP53C__55106__Dsq         201  AFDIIGDLAFGAPFGMLDACKDSAPVAVSHKAAMAAYGSSDS-SKE--------IQIEHFPAVQVLNDRG  261
CYP53C4__GL08839-P1.1__Gl  201  AFDIIGDLAFGAPFGMLLACKDSAPVAVSCEAAMASYGSASS-SKE--------IQIEHFPAVQVLNDRG  261
CYP53C4__47512__Gsp        201  AFDIIGDLAFGAPFGMLIACKDSAPVAVSCEAAMASYGSAAS-SKE--------IQIEHFPAVQVLNDRG  261
CYP53C__129211__Tve        203  AFDIIGDLAFGAPFGMLTSCKDSAPVAVSQDDAMATYGKDAA------------YKVEHFPAVQVLNDRG  260
CYP53C9__27837__Pbr        202  AFDIIGDLAFGHPFGMLQACQDAAPVAVSQEAAMAAYGEGKQ------------FEVTNIPAVRILNDRG  259
CYP53C7__118978__Bad       205  AFDIIGDLAFGSPFGMLQACRDAAPVAVSQEDAMAGYGGKQ-------------CDVVYIPAVQILNDRG  261
CYP53C__183109__Pca        201  AFDIIGDLAFGAPFGMLLAARDAAPVAVNHEQAMASYGKEK-------------SEVQYIPAVQVINDRG  257
CYP53C2__130996__Pch       186  AFDIIGDLAFGAPFGMLLAARDAAPVAVDHEQAMASYGKEK-------------SEVQYIPAVQVINDRG  242
CYP53C__128292__Fme        208  AFDIIGDLAFGSPFGMILKGKDAAPVAKDQKAAIAGYGRESASEKSA-------CDVTELPAVQVLNDRG  270
Cyp53C__37267__Tve         201  AFDIIGDLAFGAPFGMLTSGKDSAPIAVSQVDAMAAYGQGGT------------LKVKHVPAIQVINDRG  258
CYP53C__70450__Ade         197  AFDIIGDLAFGSPFGMLDACADSANAAVGGVNAL---KDGKP------------MQTVSVPAIRILNERG  251
CYP53C__52716__Dsp         204  AFDIIGDLAFGEPFGMINSGADSASVAIHGDDPTHLASGEKK------------LEIVRVPAVKILNDRG  261
CYP53C__83844__Cpu         206  AFDIIGDLAFGQPFGMILKAKDSAPVAVSQDAAMDSYGKE--------------CKVIEVPAVKILNDRG  261
CYP53C__194303__Abi        204  AFDTIGDLAFGEPFGMLAAAKDMAVVPKDQQSAMNSYGKETK-----------EEDILTVPVIEAFNNRG  262
CYP53C__194181__Abi        208  AFDIIGDLAFGEPFGMLQAAKDSAVVPKDQKSMMKSYGKEDA-----------SIEVMEIPAVQILNGRG  266
CYP53C__143663__Fme        204  AFDIIGDLAFGSPFGMVRNAKDAAPIAVDRKSAMAQYGPVIT-DNRGLEKP--VIDVREVHAISVLENRM  270
CYP53C__162664__Fme        214  GFDITGDLAFGFPFGMVCNASDTVQITVDQKASMDKYGQKDA-DQGSGKSAI-ETETKEIQAVKVLNNRT  281
CYP53C__154594__Fme        198  TFDIIGDLAFGAPFGMIRKGKDAAPVAVDLKAAIAQYGQAGI-DGQDLEKP--AIQVKEVPAVQILNDRT  264
CYP53C__149618__Fme        206  AFDVMSDLAFGASFGMVRNAKDAAPIAVDQRAAMAQYQGTRV-DSLDLEKP--SIDVKEVPAVTMLNAHI  272
CYP53C__130308__Fme        205  AFDIISDLAFALPFGMLRNAKDAALTAVDQKAAMSENGQVNT-DMQDIEKP--VVAVREVPAVKVLNGRS  271
CYP53C__24265__Fme         205  AFDIIGDLAFGAPFGMLRNAKDAAPTAVDQKAAMSENGQVNI---QDLEKP--VVAVREVPAVKVLNGRS  269
CYP53C__94457__Fme         206  SFDIISDLAFGTPFGMIRKARDAVPVAIDHKAAMAQYGQIDT-EYRDVKKL--VIDTREVPAIQVVNEQG  272
CYP53C__115179__Fme        205  CFDIIGDLVLGAPFNMVHKGTDTVPVALEPSAVIAQYGQSSI--TGSHDTEKPICAVKEAPAMELMNGRS  272
CYP53C1__5__Uma_           207  AFDTIGALAFGKTFGMLENGVDQAKVEYEDANGNKQV--------------------DYCSAVQIINERG  256
CYP53C__48859__Fpi         210  SFDTIGDLAFGLPFGMLKSGRDTAKVAKSAEDALKAIDTVSK--GGDVLA----IEEEEIPYIEYQSARA  273
CYP53C__86809__Fpi         210  SFDTIGDLAFGLPFGMLKSGRDTAKVAKSAEEGFKAIDAMSK--GGDALV----VEEEEIPYIEYLSARA  273
CYP53C__138909__Wco        213  SFDTIGDLAFGSPFGMLSAASDTVRVAKSVKASLATFGTSSN--AEEFGF-----ETEEMAIEKVLNERG  275
CYP53C__104840__Wco        215  SFDTIGDLAFGAPFGMLLSAKDTVRVATSVKAGMAAFGTSST--TGKFTL-----ETEEIPATKLLNKRA  277
CYP53C__77097__Wco         219  SFDTIGDLAFGAPFGMLLAAKDTARVAKSVKAGLATFGTVSR--TGEFAF-----ETEEIPVTKLLNKRA  281
CYP53C__138864__Wco        213  SFDTIGDLAFGSPFGMLSAANDTVRVAKSVKASLATFGTSSN--AGEFGF-----ETEEMAIEKMLSERG  275
CYP53C__104855__Wco        209  ------DLAFGAPFGMILAAKDTARFAKSVMAGMAAFGTSSK--TSEYAF-----ETDETPVTKLMAERA  265
CYP53C__154264__Wco        192  SFDTIGDLAFGDPFGMILAAKDTARSAKSVKASLETFSTSSN--TEKLAF-----ETEELPVTKIMGQRG  254
```

```
CYP53c__138853__Wco        213  SFDTIGDLAFGAPFGMILAAKDTARVAKSVKASLATFGTPSQ--TGKFAF-----ETEELPVIKIVGQRA  275
CYP53C__154237__Wco        114  SFDIIGDLAFGDPFGMILAAKDTARSAKSVNASLATFSTSSN--TKKFAF-----EMEELPVTEIIGQRG  176
CYP53C__112429__Csu        208  AFDTIGDLAFGSPFGMLVSGKDTARIAKSLKAAMQTLGSTPS--ATEKPST---IEEEDIPAISSINRRS  272
CYP53C__151209__Csu        210  SFDTIGDLAFGKAFGMVESGKDIARVAKDYTDAMRTYNAKQE--LPEWTPA---YEEEEIPAISSLKERS  274
CYP53C__118598__Csu        221  AFDTISDLAFGSPFGMLIAGRDTARVARSVDIAMKNLGVAQT--AQESDRI---YEEEDIPAISANNTRS  285
CYP53C__101826__Pca        174  TFDVIGDLAVGAPFGMLEAGKDTALVPVSEEQAMKSFGQQDT--DLEW---------ATIPTIKLLNETV  232
CYP53C__256510__Pca        191  TFDIIGDLAFGASFGMLEAGKDTAPVPVYTDQAMKSYGQKDT--DLEW---------STAPAVQILNEAI  249
CYP53C__212559__Pca        202  TFDIIGDLAFGAPFGMVEAGKDTASVPVSEKQAMKFYGQKGA--EIEW---------STAPAIKIVNEAV  260
CYP53C__102576__Pca        203  TFDVIGDLAFGKPFGMLEAGKDAALVPVSEEQAMKSFGRQDT--DLKW---------ATIPAIKLLNETV  261
CYP53C__212558__Pca         81  AFDVIGDLAFGAPFGMLEAEKDTVPVPVSEEQAMKSYGQKDM--DLEW---------STLPAIKLLNETI  139
CYP53__1116154__Fpi        182  IARVTNRVFVGMPFC---RNEMLLETA-------------------------------------VEFAKDVM  213
Consensus aa:                   sFDhIGDLAFG.PFGMl..tcDsh.hs.s.p.th..@tp.p..............ch.phPhlphhspp.
Consensus ss:                   hhhhhhhhh             hhhhhhhhhhhhhhhhhhhh  hhhh       hhhhhhhhhhhhhhh

Conservation:                         6  8    5      6                5   6  85             5 8 8  8
CYP53C__1025718__Fpi_      259  EYSASLGVLPPHWRPIVKL-LPWYRKGNKAVQR-LAGIAIAQVAKRLAMHTD--------RSDLLGKLQE  318
Cyp53C__68781__Pst         253  EFSASIGVLPPAWRPFVKNLIPWYRNGSKAVKN-LAGLAVAAVAKRLDRDTLNG---GSDRVDLLAKLQQ  318
CYP53C__80617__Gtr         257  EYSASMGVLPVWFRPVVQRLHPWYRNGNKAVKD-LAGLAVAAVAKRLRNPTD--------RVDLLSKLQE  317
CYP53C__127772__Cpu        280  EYSAAMGVLPPAIRPFMQRFVPWYRKGGKAVRN-LAGIAVAAVAKRLNEPSD--------RVDLLRRLQE  340
CYP53C__94174__Shi         262  EFSASMGVLPPWLRPYVKRYIPWFSKGDQAVKN-LAGLAIAAVSKRLNQPTY--------RVDLLSKLQE  322
CYP53c__27029__Wco         259  EFSASLGVLPPHWRPLVVRFIPWYRNGNKAVKR-LAGIAIAAVAKRLTAPTD--------RSDLLAKLQE  319
CYP53C3__110015__Ppl       258  EYSASMGVLPPHWRPLVVRFIPWYRNGGKAVKR-LAGIAIAAVSKRLTAPTD--------RADLLGKLQE  318
CYP53C__116910__Csu        257  EYSASMGVLPPHWRPLAKR-IPWFRRGNQAVQR-LAGIAVAAVAKRLSAPSD--------RTDLLSKLQE  316
CYP53C__1179842__Sla       264  EFSASMGVLPPHWRPLVRL-LPWYRKGGKAVKN-LAGLAVAAVAKRLTTPTD--------RVDLLSKLQE  323
CYP53C__55106__Dsq         262  EYSAAMGVLPPHWRPLAKK-IPWYSKGNQAVQK-LAGIAVAAVAQRFANPSD--------RADLLSKLQE  321
CYP53C4__GL08839-P1.1__Gl  262  EYSASMGVLPPHWRPLAKR-IPWFAKGNQAVQR-LAGIAVAAVAQRLGSPSD--------RVDLLAKLQE  321
CYP53C4__47512__Gsp        262  EYSASMGVLPPHWRPLAKR-IPWYARGNQAVQR-LAGIAVAAVAQRMASPSD--------RVDLLAKLQE  321
CYP53C__129211__Tve        261  EYSASMGVVPPRWRPLVKR-LPWYNKGNQAVQR-LAGIAIAAVARRLSVPES-------DRHDLLEKLQE  321
CYP53C9__27837__Pbr        260  MFSASLGVLPPWMRPLAKQ-LPWFKKGNAAVKT-LAGIAVAAVARRLATPVD--------RVDLLGKLQD  319
CYP53C7__118978__Bad       262  NFSASLGVLPPWMRPIVKQ-LPWFKKGQKAVKD-LAGIAIAAVAKRLTTPSD--------RTDLLGKLQQ  321
CYP53C__183109__Pca        258  MYSASLGVLAPWMRPIVKL-FPWFRQGQQAVKL-LAGIAVAAVSQRLTTPTD--------RVDLLGKLQG  317
CYP53C2__130996__Pch       243  TYSASLGVLPPWMRPIVKL-FPWFRRGQKAVKQ-LAGIAVAAVAQRLTTPTD--------RVDLLGKLQE  302
CYP53C__128292__Fme        271  EYSASMGVLPPWWRPFVRR-IPWYANGNRAVKN-LAGLAVAAVAKRLANPTD--------RTDLLSKLQE  330
Cyp53C__37267__Tve         259  EYAASVGVLPPHWRPFVKR-LPWYNTGDKAVQN-LTGMAIAAVARRMEESDS-------DHRDLLAKLRE  319
CYP53C__70450__Ade         252  EFSATMGVLAPWMRPLVLK-LPWFARGLSAVRA-LAGLAIAAVGRRLAEPSD--------RNDLLAKLQD  311
CYP53C__52716__Dsp         262  EYSASMGCLPIWIRPYAKK-IPWYAKGNQAVKN-LAGIAIAAVDKRLATPTD--------RVDLLARLQQ  321
CYP53C__83844__Cpu         262  DYNATLGTMPPWVRPYVRK-LPWFSQGSEAAAS-VAGMAVAAVSRRLTTPTD--------RVDILSKLQQ  321
CYP53C__194303__Abi        263  EFNLVMGSLPLHWRPLARR-LPGLAQGSRDFKT-VAGIAVAAASKRLSSSTD--------RIDLMSKLQN  322
CYP53C__194181__Abi        267  EFSLTMGTLPPYWRPIARR-LPGFRQGAQDVKN-LAGIAIAAVAKRLATPTD--------RNDLLSNLQA  326
CYP53C__143663__Fme        271  RLSAQMGVLPAWWRPIVRQ-LPRFAQGVQNSKD-LVDLAVAAVAKRMAYPTQ--------RDDILSKLQQ  330
CYP53C__162664__Fme        282  NFDAHAGSFPPRFRPFMRR-LPRFSECLQSSED-LAGFTVTAVARRLAFPSY--------RTDILSKLQQ  341
CYP53C__154594__Fme        265  FLASHQAAFPKPLRPLLAL-LPQYAEMAKHSDE-FIGFAVAAVAKRLVFPTE--------RVDILSKLQQ  324
CYP53C__149618__Fme        273  KVSARMAAVPPRWRPILQR-LPCFARDMRASED-LVALAVAAVARRLVFPAE--------RIDVLSKLQE  332
CYP53C__130308__Fme        272  EYSASMGVLPPWWRPIVRL-LPWYADGSQDVED-LAGLAVAAVAKRLAIPTD--------RTDLLSKLQQ  331
CYP53C__24265__Fme         270  EYSASMGVLPPRWRPIARL-LPWYAEGSKDVED-LAGLAVAAVAKRLAIPTD--------RADILSKLQQ  329
CYP53C__94457__Fme         273  EGVAQIAAFPPLWVPFLRC-LPRFAKGMRRVED-FIGLVVLAVANRLAFPTE--------RVDILSKLQQ  332
CYP53C__115179__Fme        273  AVIASLGVLPPWWRPIASL-FPWYARGNRDVGD-LAGFATLAISKRLARPTE--------PLGLLSALLE  332
CYP53C1__5__Uma_           257  EFSGTMGLAPVWMRPYLIK-LPWFSSRLKSVKK-LTGIALARVNDRLQNGSE--------REDLLAKLQA  316
CYP53C__48859__Fpi         274  ETDACLAWLPPIWVRILGK-LPMFSVHALTGQK-LAALSIMAVARRIADPNP--------REDMLQKLLE  333
CYP53C__86809__Fpi         274  ERNACLAWLPPVWARVVLT-LPAFSGYALTGRK-LAALSIMAVARRLANPNP--------REDMLQKLLE  333
```

```
CYP53C__138909__Wco          276   HLITILGWLPEYWRRIVQM-LPAYRFGMEAAPK-MAGLAVAAVGKRLNNPAA--------REDMLNRLLD  335
CYP53C__104840__Wco          278   KLVTTLGWFPKYWQPIIEL-LPPFRAGREATPK-LAGLAVAAVAKRLSNPDA--------REDMLNRLLD  337
CYP53C__77097__Wco           282   ELVAILGWLPKYWQSIVGT-LAVFSGGSNASPK-LAGLAVASVAKRLSNPQA--------REDMLNRLLD  341
CYP53C__138864__Wco          276   HLISILGWLPEYWRRIVQM-LPAYRFGTEAAPK-MAGLAVAAVGKRLNNPAA--------REDMLNRLLD  335
CYP53C__104855__Wco          266   DLVATIGWLPEYWQPIVRM-LPAFRGGRKSTPQ-LAGLAVAAVAKRLSKPDA--------REDMLNRLLN  325
CYP53C__154264__Wco          255   ELVAMLGWLPEYVRPIILM-MPGFRSNLQAIPK-VAGLAVAAVAKRMNNPDA--------RADMLNRLLD  314
CYP53c__138853__Wco          276   ELVAMFGWLPEYVWPIILM-MPGFRGSRRAIPQ-VSGLAVAAVAKRMNNPDA--------RADMLNKLLD  335
CYP53C__154237__Wco          177   ELITMLGWLPKYVRPIILM-MPGFRSNLQAIPK-VAGSAVTAVAKRMNDLDA--------HADMLNRLLD  236
CYP53C__112429__Csu          273   EFLIAFASLPAWIRPIVKR-LPMASDGMAATRE-IMSMAVTTVSRRVRTLYDGDA-EERQRPDFLTKLLE  339
CYP53C__151209__Csu          275   KYMIFVGTLPKSLRGIMRF-LRSRDHQVMALRR-IMSMATSSVARRIRAGREQ------DRDDFLARLLQ  336
CYP53C__118598__Csu          286   DFLMFLAYLPEWLRPIVVR-LPLFDDGVAAAGK-IMSMAVTTVLRRLHALSSDSGDEKKNYEDFLIKLLQ  353
CYP53C__101826__Pca          233   PWIFFLGCLPPQARFLMSK-LQSFNAG-ASRKL-FQKLGVAAVSKRLSSEAT--------RRDFLSQLVA  291
CYP53C__256510__Pca          250   PWFFFLGCLPPQARRLVST-LQSFNAG-GSRNL-IGKIAVAAVSKRLTSEVT--------RRDFLSHLVA  308
CYP53C__212559__Pca          261   PWVFFLGCLPPHVRPLLSK-LRSFTTK-ASARA-LVKIAVAAVSKRLATGVT--------RRDFLSHLIA  319
CYP53C__102576__Pca          262   PWTFFLGCLPPQARFLMSK-LPSFNAG-TSRKL-FVKLAVATVSKRLASEAT--------RRDFLSHLVA  320
CYP53C__212558__Pca          140   PRTSFLGCLPP-----------------------------------------------------------  150
CYP53__1116154__Fpi          214   KTKFIVNLFPDVLKPIVGHRLPWTTARRRKMAEILGDTVRERRRQMLEYGTDYE----GKPDDYLTWVVE  279
Consensus_aa:                      .h.h.hthhP.hh+Plh...lP.@....ps..p.lhshtlhsVscRl..ss.........+.DhLs+L.p
Consensus_ss:                      hhhhhhhhhhhhhhhhhhh hhhhhhhhhhhhh hhhhhhhhhhhhhh               hhhhhhh


Conservation:                        5 5 8      566566   6 7867857 5 5 5  6 55       596 878  6
CYP53C__1025718__Fpi_        319   GKD-DEGNPMGREELTAEALTQLIAGSDTTSNSSC-AITYHLAANPMVQQKLQRELDEALGND------D  380
Cyp53C__68781__Pst           319   GKD-DEGKPMGREELTAEALTQLIAGSDTTSNSSC-AITYHLAANPNVQAKLHAELDEALGTD------D  380
CYP53C__80617__Gtr           318   GKD-DEGKPMGREELTAEALTQLIAGSDTTSNSSC-AITYYLALHPRVQEKLQAELDEALGND------D  379
CYP53C__127772__Cpu          341   AKD-DEGNPMGREELTAEALTQLIAGSDTTSNSSC-AITYYLALHPEIQTKLQRELDDALGTD------D  402
CYP53C__94174__Shi           323   GKD-DEGRPMGREELTAEALTQLIAGSDTTSNTSC-AITYYLAANPAVQEKLHVELDAALGNE------D  384
CYP53c__27029__Wco           320   GKD-DDGNPMGREELTAEALTQLIAGSDTTSNSSC-AITYYLAANPLVQQKLQRELDEALGND------D  381
CYP53C3__110015__Ppl         319   GKD-DEGNPMGREELTAEALTQLIAGSDTTSNSSC-ALTYHLAANPRVQQKLQRELDEALGSD------D  380
CYP53C__116910__Csu          317   GKD-DEGKLMGKPELTAEALTQLIAGSDTTSNSSC-AITYHLAANPHVQEKLQAELDAALGDG-------  377
CYP53C__1179842__Sla         324   GRD-DEGKLMGREELTAEALTQLIAGSDTTSNSSC-AITYYLAQNPDAQEKLQKELDEALGDD------D  385
CYP53C__55106__Dsq           322   GRD-DNGDPMGREELTAEALTQLIAGSDTTSNSSC-ALTYWLAKNQAAQRKLQQELDAALGSD------D  383
CYP53C4__GL08839-P1.1__Gl    322   GRD-DNGDPMGREELTAEALTQLIAGSDTTSNSSC-AITYWLARNQAAQRKLQAELDGALGSAS----DD  385
CYP53C4__47512__Gsp          322   GRD-DNGDPMGREELTAEALTQLIAGSDTTSNSSC-AITYWLARNPAAQRKLQAELDGALG--S----ND  383
CYP53C__129211__Tve          322   GRD-DNGDPMGRAELTAEALTQLIAGSDTTSNSSC-AITYYLAKYQHVQEKLQKELDDALGGE------D  383
CYP53C9__27837__Pbr          320   GRD-DEGNPMGREELTAEALTQLIAGSDTTSNSSC-AITYHLAKNPDVQKKLQAELDEVLGND------D  381
CYP53C7__118978__Bad         322   GRD-DEGNPMGRPELTAEALTQLIAGSDTTSNSSC-AITYHLAKNPEVQRRLQKELDDALGAHA-----D  384
CYP53C__183109__Pca          318   GRD-DDGNLMGKEELTAEALTQLIAGSDTTSNSSC-AITYYLAKYPDVQRKLQQELDEVLGYD------D  379
CYP53C2__130996__Pch         303   GRD-DDGNLMGKEELTAEALTQLIAGSDTTSNSSC-AITYYLAKYPDAQRKLQQELDEALGSD------D  364
CYP53C__128292__Fme          331   GKD-DEGRPMGREELTAEALTQLIAGSDTTSNSSC-AITYHLAHNPHVLKRLQQELDTALAGE------D  392
Cyp53C__37267__Tve           320   ARD-EDGNPMGREELTAEALAQLVAGSDTTSNSSC-AITYYLAKHQRVQEKLQQELDEALASE------E  381
CYP53C__70450__Ade           312   AKD-DDGMPMGPEELTAEALTQLIAGSDTTSNSSC-AIAYYVARYPRVQLKLQQELDAALPN--------  371
CYP53C__52716__Dsp           322   GKD-EQGNLMARSELTAEALAQLIAGSDTTSNSSC-AITYWLAKYPDAQRKLQKELDEALGDD-------  382
CYP53C__83844__Cpu           322   GKD-ENGEIMGPEELTAEALTHLVAGSDTTANSSC-AIIYYLAAYPHVQEKLQKELDEALGSE------D  383
CYP53C__194303__Abi          323   SRD-SNGNPMSREEMTAEALTLLVAGSDTSSNACA-AFLYHVAANPSVQDKLHQELDEQLGTE------D  384
CYP53C__194181__Abi          327   GRD-SEGKPLGREELTAEALTLLIAGSDTTSNSTC-AILYYLARNRGAQEKLQKELDEHLGTE------N  388
CYP53C__143663__Fme          331   SRD-EYGRPLTQEDLTTDAITQLVAGSDTISISSC-GIAYHLAANPDVQSKLQKEIDDALGGF------D  392
CYP53C__162664__Fme          342   SKD-EHGKPFSKEEVTSDAQTMLIAGSDTVSNTSS-ATAYYIAKNPGVQIRLQKELDEAFAAS------D  403
CYP53C__154594__Fme          325   SKD-ENGNPQSREDLTTDGITQLVAGSDTVANTSC-GITYHIASNPCVQAKLQAELDDALGKDM-----E  387
CYP53C__149618__Fme          333   TED-EHGRVSNMEDLTTDAFTQLVAGSDTVSSTAC-GIAHCVAANSRVRAKLQQELDVVFGGS------Y  394
CYP53C__130308__Fme          332   GRH-EDGRPLNREELTADALTQLIAGSDTTANSSC-AVLYHIISSPRVQAKLQKELDEALASL------D  393
```

```
CYP53C__24265__Fme         330  GRH-EDGSPMSREELTADALTVLIAGSDTTSNSTC-ALMYYITSNPRVQAKLQKELDEALVSF------D  391
CYP53C__94457__Fme         333  GKG-EDGVPLTKEELTSEALVQLIAGSDTTSNTTC-AITYYVAANPHVQTKLQKELDNALGHS------E  394
CYP53C__115179__Fme        333  LKD-DEGKPLSKEQLSADGLLLLIAGSDMVANPTC-AVLYQIIANPPVQAKLQKELDDALGAPSP---SD  397
CYP53C1__5__Uma_           317  AKD-DRGEPMGKMELTAEALTQLIAGSDTTSNTSC-AIVYHLATHPDKMRKLQAELDRELEHA-------  377
CYP53C__48859__Fpi         334  ARD-EEGKPLSPQEMSAEAFVLIIAGSDTIANTTC-GTTYYLARDKRVQAKLQAELDGALASV------D  395
CYP53C__86809__Fpi         334  ARD-EEGKPLSPQEMSSEAFLLIIAGSDTIANTTC-GTTYYLARDKRVQAKLQAELDGALASV------D  395
CYP53C__138909__Wco        336  ARD-ENGKPMSPEELSAEAFVLIIAGADTTANTSC-ATTYYLARDQRVQAKLQAELDEALKSI------D  397
CYP53C__104840__Wco        338  ARD-ENGEPLSPEELSAEAWLLIIAGADTVANTSC-ATTYYLARDQRVQTKLQAELDEALKSI------D  399
CYP53C__77097__Wco         342  ARD-DNGEPMSPEELSAEAMTLIIAGADTVANTSC-ATTYYLARNQRVQAKLQAELDEALKAV------D  403
CYP53C__138864__Wco        336  ARD-ENGKPMSPEELSADAFQLIVAGADTTANTSC-ATTYYLARDQRVQAKLQAELDEALKSI------D  397
CYP53C__104855__Wco        326  ARD-EDDEPLSREELSAEAAMLIAAGADTVANTSC-ATTYYLARDQRVQAKLQAELDEALKSV------D  387
CYP53C__154264__Wco        315  A-----------------------PGAITVANTSC-ATTYYLARDQRVQAKLQTELDDALKAV------D  354
CYP53c__138853__Wco        336  ARD-ENGEPMSPEELSSEAFLLIVAGSDTVSNTSC-ATTYYLARDQRVQAKLQAELDDALKAV------D  397
CYP53C__154237__Wco        237  ARD-ENGEPMSPEELSSEASLIIVAGAITVANTSC-AITYYLARDQRVQAKLQAELDDALKAD------D  298
CYP53C__112429__Csu        340  GRD-EEGSPLSPDELSSEAQTLLIAGSDTISNSTC-AIVYWIARNPDVQKKLQAELDAALADAG-----E  402
CYP53C__151209__Csu        337  ARD-DDGNPLSPDELSSEAQTLLTAGADTISNSTC-ATVFWIARAPPVKARLQAELDATLGISST--DLG  402
CYP53C__118598__Csu        354  GHN-DDGNRMGPEELTSEAQVLLIAGSDTISNSTC-ATVYWVARHLNVQRNLQSELDGALADVSS--DED  419
CYP53C__101826__Pca        292  ARD-DEGKPLSAQELTSEALNLIIAGSDTTSSSIG-AIIYHIARNRDVQERLQKALDDVLGVPNSMFSTD  359
CYP53C__256510__Pca        309  AHD-DQGRPLSQQELTSEAISLIVAGSDTTSTSIA-AITYHVARTQDVQAKLQEELDDALGVPDASSNAD  376
CYP53C__212559__Pca        320  VRD-DQGRPLTEQELTSEAISLIVAGSDTTSSSIA-AIAYHVARNQDVQAKLQAELDDVLGAPGSDSSTD  387
CYP53C__102576__Pca        321  ARN-DEGKPLSAQELTAEALNLIVGGSDTTSSSIG-VVIYHVARNRDVQERLQKELDDVLGVPNSTFSTD  388
CYP53C__212558__Pca        151  -----QGKPLSEQDLTSEALNLIVAGSDTTSRSFDWCYRLPCCAESGCPKRLQKELDDVLRVPNSTFNTD  215
CYP53__1116154__Fpi        280  EDLKNRGKGESIDGVMEVIAASNFAAIHTSSMAMA-HALYYLCAMPQYIKPLKQEAEEKIKEH-------  341
Consensus aa:                   t+c.-pGpsht.p-Lot-AhhbllAGtDThtpo.t.thhY@lt....hb.+LQ.ELDphL.........D
Consensus ss:                   hh         hhhhhhhhhhhhh   hhhhhhhh hhhhhhhh hhhhhhhhhhhhhh


Conservation:                              7 88 565 9 6955   5 7979 66   58    8  6  95 5868 8  975
CYP53C__1025718__Fpi_      381  DPVSTFEQVKRLPYLEAVINEGLRLHSTSGIGLPRLVP--EGGLTVCGRFFPEGTVLSVPSYTIHRDQDV  448
Cyp53C__68781__Pst         381  DPVAIFDQVKRLTYLQAVIDETLRIHSTSGIGLPRIVPAGSGGMHVAGHFFPEGTVLSVPTYTIHRDKEV  450
CYP53C__80617__Gtr         380  DPVSTFEQVKRLKYLEAVINEALRVHSTSGIGLPRVVP--EGGLTVLGRTFPEGTIMSVPTYTIHRYEEV  447
CYP53C__127772__Cpu        403  DPVSTFDAVKRLPYLDSVINEALRLHSTSSIGLPRIAP--EGGLALRGLWFPPGAILSVPSYTIHRDAGV  470
CYP53C__94174__Shi         385  DPASTFEQTKNLKYLQAVIDESIRLHSTSGIGLPRIAP--EGGLTVCGKYFPEGTILSVPSYTIHRDVDV  452
CYP53c__27029__Wco         382  DPVAMYEQVKRLPYLEAVINEGLRLHSTSGIGLPRIVP--EGGLTVRGQFFPEGTVLSVPSYTIHRDREV  449
CYP53C3__110015__Ppl       381  DPVATYEQVKRLPYLEAVVNEALRVHSTSGIGLPRVVP--EGGLSVCGRFFPAGTVLSVPTYTVHRDAET  448
CYP53C__116910__Csu        378  DPVATFDQVKRLPYLEAVINEALRIHSTSGIGLPRIVP--QGGLTAAGQYFPEGTVLSVPTYTVHRDKEA  445
CYP53C__1179842__Sla       386  HPVSTFEQVKRLPYLEAVINEALRVHSTSSIGLPRIVP--EGGLIVQGQHFPQGAVLSVPSYTIHRDTTV  453
CYP53C__55106__Dsq         384  DPVASYEQVKRLPYLEAVINEALRIHATSGIGLPRLVP--EGGLTVCGKFFPEGTVLSVPTYTIHRDKAV  451
CYP53C4__GL08839-P1.1__Gl  386  DSIASFEDVKRLPYLEAVINEALRIHATSGIGLPRLVP--EGGLTVCGRFFPEGTVLSVPTYTIHRDREV  453
CYP53C4__47512__Gsp        384  DPIASFEDVKRLPYLDAVINEALRIHATSGIGLPRLVP--EGGLTVCGRFFPEGTVLSVPTYTIHRDREV  451
CYP53C__129211__Tve        384  DSVASYEQVKRLPYLDAVINEALRIHATSGIGLPRLVP--AGGLEVCGRWFPEGAVLSVPTYTIHRDKAV  451
CYP53C9__27837__Pbr        382  DPVSTYEEVKKLAYLQAVIDEALRIHSTSGIGLPRVVP--EGGLTVCGQFFPEGTVLSVPTYTIHRDTHV  449
CYP53C7__118978__Bad       385  EPVVTFEDVKRLPYLQAVIDEALRIHSTSGVGLPRLVP--EGGLTVCGQYFQEGTVLSVPTYTIHRDKEI  452
CYP53C__183109__Pca        380  EPVSTYDQVKKLTYLPAVIDEALRVHSTSGVGLPRVVP--EGGMTVCGRTFPEGTILSVPTYTIHRDEEV  447
CYP53C2__130996__Pch       365  EPVSTFDQVKRLPYLQAVIDEALRIHSTSGIGLPRLVP--KGGMTVCGRFFPEGTVLSVPTYTIHRDEEV  432
CYP53C__128292__Fme        393  DPVATFQQVKSLPYLDAVINEVLRIHSTSGIGLPRLVP--EGGLTVCGKTFPEGTVLSVPTYTIHRDKEV  460
Cyp53C__37267__Tve         382  DEVALFERVKHLPYLEAVINEALRIHSTAGVGLPRVVP--AGGLEVCGKFFPEGTVLSVPGYTIHRDKAV  449
CYP53C__70450__Ade         372  DGVTTYEQVKRLPYLTAVINEGLRLHSTSAMGLPRIVP--EGGLTVAGRFFTEGSILSVPSYTIHRDPEV  439
CYP53C__52716__Dsp         383  EDVPTYEQLKRLRYLDAVVNEGLRIHSTSSLGLPRIVP--EGGLEVSGIHFPAGSVLSVPSYTIHRDTAI  450
CYP53C__83844__Cpu         384  EPVTTYEQVKRLTYLEVVILEVLRLHSTIGLGLPRMAP--EGGLTVHGTYFPEGTILSVPTYTLHRDKRV  451
CYP53C__194303__Abi        385  ELVATAEQIKRLTYLEACINEALRIQSVSGIGLPRVVP--EGGLEVLGNFFPEGTVLSVPSYSVHRDTKS  452
```

```
CYP53C__194181__Abi        389  EFTATEAQVKNLPYLDACINEGLRLHSTSSVGLPREVP--EGGMMVCGQFFAEGTVLSVPSYTIHRDRGV  456
CYP53C__143663__Fme        393  DPMVTYAQIKHLQYLEAVINEGLRVHPTPGLGLPRVVP--EGGLNVCGKWFPEGTILSVPTYTIHRNTGV  460
CYP53C__162664__Fme        404  DAVATYAQIKDLPYLAAVVNEGLRLHAPVGVGLQRVVP--KGGLTVCGKWFPEGTILSVPTYTIHRDPGV  471
CYP53C__154594__Fme        388  DPVVTYAQIKNLPYLEAVLNEGQRVYSTAALGLQRIVP--EGGLTISGKWFPEGTIVSVPTYTIHRDPKV  455
CYP53C__149618__Fme        395  DPVATYAQIKRLPYLEAVIIEGLRVHSTSGLGLPRTVP--NGGLIVCGKWFSEGTVLSVPTYTIHRDPIV  462
CYP53C__130308__Fme        394  DPVASYDLVKHLPYLDAVIHEGLRVHSTSGNGLPRLVP--EGGLTVCGKWFPAGTVLSAPTYTIHRDPKV  461
CYP53C__24265__Fme         392  DPVTSYDLVNHLPYLDAVIHEGLRVHSTLGVGLPRLVP--EGGLTVCGKWFPEGTVLSAPTYTIHRDPKV  459
CYP53C__94457__Fme         395  NHVATYSQIKQLSYLDAVVNEGLRVHSTVGIGLPREVP--EGGITVLGKSFPEGTVLSVPIYTIHRDPKV  462
CYP53C__115179__Fme        398  DSVSTYSQINHLPYLEAVINEALRVHPMVGLGLPRVVP--ASGLTVCGKHFPEGTVLSVPTYTIHRDKEV  465
CYP53C1__5__Uma_           378  EEVPLHADVQELPYLQAVLSESLRYHSTSAIGLPRVIP--AGGATVCGQQFPSGTILSVPAYTLHRDKSV  445
CYP53C__48859__Fpi         396  SEVAPYDTVKDLPYLDAVIHEGQRLYSTIGAGLPREVP--AGGATILGHHFKEGTTISVPIYRLHRDEST  463
CYP53C__86809__Fpi         396  SEVVPYDAVKDLPYLDAVIHEGQRLHSTVGAGLPREVP--TGGATILGHHFREGITLSVPIYRLHRDESI  463
CYP53C__138909__Wco        398  SAVAPYDAVKNLPYLDAVINEGLRLHATIGAGLPRVVP--EGGLTVLGHTFKEGTWVSVPVYHLHRDESI  465
CYP53C__104840__Wco        400  SAVAPYDAIKHLPYLDAVVNEGLRLHATVGAGLPRVVP--EGGLTVLGHTFKEGTWVSVPVYHLHRDESI  467
CYP53C__77097__Wco         404  SEVALYDAVKYLPYLDAVVNEGLRLHATIGAGLPRVVP--EGGITVLGHTFKEGTCVSVPIYYLHRDESI  471
CYP53C__138864__Wco        398  SAVAPYDAIQNLPYLDAVVNEGLRLYTTVGAGLPRVVP--EGGLTVLGHTFKEGTWVSVPIYRLHQDESI  465
CYP53C__104855__Wco        388  SVAAPYDAIKHLPYLDAVVNEGLRLHATIGAGLPRVVP--EGGLTMLGHTFKEGTWVSVPVYHLHRDESI  455
CYP53C__154264__Wco        355  SVVAPHGAIKHLPYLDAVVNEGLRLHSPVGAGLPRVVP--EGGMTVLGHTFKEGTWVSVPTYHLHRDENI  422
CYP53c__138853__Wco        398  SVVAPHDAIKHLPYLDAVVNEGLRLHSAVGAGLPRVVP--EGGMTVLGHTFKEGTWVSVPIYHLHRDENI  465
CYP53C__154237__Wco        299  SIVAPHDAIKHLPYLDAVVNEGLRLDS----------P--APGSAYLR----------------------  334
CYP53C__112429__Csu        403  GPIAPVEKTERLLYLNAVIDEGLRVHSTVGANLPRVVG--PEGVTILGHTFTEGTVVSVPAYSTHRDENI  470
CYP53C__151209__Csu        403  SPVAPIDKIEHLPYLNAVIDEALRIHSTVAAGLPREVG--PGGLNVLGHFFPEGAVLSVPTYSAHRDESI  470
CYP53C__118598__Csu        420  SFVAPIDKIDNLPYLNAVVDEGLRVHSAVGANLPRTVG--PEGATVLGHSFQEGTILSVPAYSAHRDEQV  487
CYP53C__101826__Pca        360  EVVASFDLVKNLTYLQDVINEGLRLHSTVGVGLPREVP--EGGMTVAGKTLLAGTHVSCPTYTLHRLKSI  427
CYP53C__256510__Pca        377  NVVAPFDLVKNLAYLQDVINEGLRLHSTIGVGLPREVP--EEGLTVAGKALLPGTHVSCPLYTLHRLKSI  444
CYP53C__212559__Pca        388  DVVAPFDRVKNLTYLQDVINEGLRVHSTLGAGLPREVP--EGGLTVAGKTLLAGTHVSCPSYTLHRLKSI  455
CYP53C__102576__Pca        389  EVVAPFELVKNLTYLQDVINEGLRLHSTVGVGLPREVP--EGGMTVAGKALLPGTHVSCPTYTLHRLKSI  456
CYP53C__212558__Pca        216  EVVASFDRVKNVAYLQDAINEGLRLHSTVGVGLPREVP--QGGLTIAGKALLPGTHVSCPSYTLHRLKSI  283
CYP53__1116154__Fpi        342  --GWTKTAMDAMWKTDSFFKESLRLNGVNHLSLFRKSM--KDVVLSNGTVIPAGTIVVATSTGTHLQEAL  407
Consensus aa:                   p.lhshp.lcpLsYLpthlpEtLRlcthsthGLPR.VP...sGhhlhGphh..GThlShPsYslHRpp.l
Consensus ss:                        hhhhh  hhhhhhhhhhhhh   hh              eee  eee    eeee hhhh   hhh

Conservation:                   78 8   669697                       6 688 97879 97568 567   5655 558 5
CYP53C__1025718__Fpi_      449  WGSDADAFRPERWF-EQDE-----------KAIQKTFNPFSFGPRSCVGRNLASMELLIILSSILRRYHF  506
Cyp53C__68781__Pst         451  WGEDVEVFRPERFL-EGDQ-----------AVIQKTFNPFSFGPRACVGRNLANMELLIIIASILRRYHF  508
CYP53C__80617__Gtr         448  WGPDVDEFRPERWF-EIDQ-----------AQINKAFNPFSYGPRACVGRNLASMELMIIVSSIFRRYHF  505
CYP53C__127772__Cpu        471  WGADTEAFRPERWA-EEER----------RDAVQRAFNPFSFGPRACVGRNLASMELLVIVSSILRRYTF  529
CYP53C__94174__Shi         453  WGYDVEAFRPERWF-ERDA-----------EMIQKAYNPFSFGPRACVGRNLASMELLIIISSILRRYDF  510
CYP53c__27029__Wco         450  WGADVDAFRPERWM-ELDK-----------NAVQKTFNPFSFGPRSCVGRNLASMELLIIIGSILRRYHF  507
CYP53C3__110015__Ppl       449  WGADVDAFRPERWE-ERDK-----------NAVQKAFNPFSFGPRSCVGRNLASMELLIIIASILRRYHF  506
CYP53C__116910__Csu        446  WGEDADLFRPERWF-EHDE-----------KTLQRAFNPFSFGPRSCVGRNLANLELLIIIASILHRYHF  503
CYP53C__1179842__Sla       454  WGADPDQFRPERWF-ECDH-----------AAIQKTFNPFSFGPRACVGRNLASMELLIIISSILRRYHF  511
CYP53C__55106__Dsq         452  WGEDVDDFRPERWF-EQDK-----------NLVQKTFNPFSFGPRSCVGRNLANLELLVIVASIFRRYHF  509
CYP53C4__GL08839-P1.1__Gl  454  WGEDVDAFRPERWF-ERDK-----------NLVQQAFNPFSFGPRSCVGRNLANLELLVIVASIFRRYEF  511
CYP53C4__47512__Gsp        452  WGEDVDAFRPERWF-ERDK-----------NLIQKTFNPFSFGPRSCVGRNLANLELLVIVASIFRRYEF  509
CYP53C__129211__Tve        452  WGDDVEEFRPERWF-EQDK-----------VAVQKTFNPFSFGPRSCVGRNLANLELLVIVASIFRRYHF  509
CYP53C9__27837__Pbr        450  WGDDVETFRPERWF-EQDD-----------KLIQKTYNPFSYGPRSCVGRNLASMELLIIISSILRRYEF  507
CYP53C7__118978__Bad       453  WGEDCEAFRPERWF-EQDK-----------NGIQKTFNPFSFGPRSCVGRNLANMELLIIVASILHRYDF  510
CYP53C__183109__Pca        448  WGKDVEVFRPERWF-SQDK-----------NEVQKTFNPFSFGPRSCVGRNLASMELLIIISSILRRYDF  505
CYP53C2__130996__Pch       433  WGKDPEVFRPERWF-EQDK-----------NAVQKTYNPFSFGPRSCIGRNLANMELLIIVSSILRRYDF  490
```

```
CYP53C__128292__Fme        461  WGEDVEAMRPERWL-EGDQ-----------AAIQKTFNPFSFGPRACVGRNLASMELLIIIASIFRRYEF  518
Cyp53C__37267__Tve         450  WGDDADEFRPERWF-GKDK-----------AALQKAFAPFSVGPRSCVGRNLAHLELLTFVASIFRRYSF  507
CYP53C__70450__Ade         440  WGEDFDKFRPERWS-EGDQ-----------TLIQKTFNPFSWGPRACVGRNLAMMELLLIVSTTFRRYHL  497
CYP53C__52716__Dsp         451  WGPDPDIYRPERWF-EQDA-----------EGIQATFNAFSFGPRACVGKNLASMELLLIVATIFHRYEF  508
CYP53C__83844__Cpu         452  WGDDPEIFRPERWF-EENS-----------AKMHKAFNTFSFGPRACVGRNLANLELLIIVSSLLRRYDF  509
CYP53C__194303__Abi        453  WGDDTETYRPERWF-ERDQ-----------AAMNKAFNPYSVGPRSCVGRNLAAMELSIILASIMRRYEF  510
CYP53C__194181__Abi        457  WGEDFEAYRPERWF-ERDQ-----------TLMQKTFNPFSYGPRACVGRNLASMELLIILASIMRRYDI  514
CYP53C__143663__Fme        461  WGEDANVFRPERWF-EGDQ-----------AAMQKVFNAFSFGPRACIGRNLAMMELYIIIASIFHRYEL  518
CYP53C__162664__Fme        472  WGEDADAFRPERWL-ECDQ-----------SVMQKAFNPFSIGPRACIGRNLALMELQLFASSIFHRYEI  529
CYP53C__154594__Fme        456  WGEDVDVFRPERWL-EGDH-----------SAMSKTLNTFSIGPRACVGRNLALLELHIFIASIFYRYEL  513
CYP53C__149618__Fme        463  WGEDADAFRPDRWF-ERDQ-----------TILQKAFSPFSFGPRACIGRELAIMELCIFVSSIFHRYDL  520
CYP53C__130308__Fme        462  WGEDADVFRPERWL-ERGQ-----------ATLLKAFNTFSYGPRACIGRNVATMELFIIVSSIFRRYEF  519
CYP53C__24265__Fme         460  WGEDADVFRPERWL-ERDH-----------ATLLKVFNTFSYGPRACIGRNVATMELFIFISSIFRRYDL  517
CYP53C__94457__Fme         463  WGKDVDSFRPERWI-EGDK-----------AAMQKVFSPFSVGPRACTGRNLALMSLHIFIASIFRRYDI  520
CYP53C__115179__Fme        466  WGEDADTFRPERWF-EGDK-----------STMQKVFNAYSYGLRACAGRVLANVELQIFISSIFRRYEF  523
CYP53C1__5__Uma_           446  FGADAEEYNPDRWL-APNA----------KRDFEKAFIPFSVGPRACVGRNVAMMELSILIAAIFRRYDI  504
CYP53C__48859__Fpi         464  WGPDAAEFRPERWI-EASP--------ERKKLMMDAFAPFSVGPRACIGRSLAIMQLHIIVATLFHRYDF  524
CYP53C__86809__Fpi         464  WGPDAAEFRPERWI-EASP--------ERKKLMMDAFAPFSVGPRACIGRSLAIMQLHIIVATLLRRYDF  524
CYP53C__138909__Wco        466  WGENASEFYPERWI-EASG--------DRKKAMLDAFAPFSMGPRSCIGRNLALMQLHIVLSTLFHRFDF  526
CYP53C__104840__Wco        468  WGENANEFYPERWI-EASG--------DRKKAMLDAFAPFSVGPRACIGRNLALMQLHIVLSTLFHRFDF  528
CYP53C__77097__Wco         472  WGANATEFYPERWL-DATG--------ERKKAMLDAFTPFSVGPRACIGRNLALMQLHIVIATLFRRFNV  532
CYP53C__138864__Wco        466  WGENVNEFYPERWI-EASG--------DRKKAMLDAFAPFSMGPRSCIGRNLALMQLHIVLSTLFHRFDF  526
CYP53C__104855__Wco        456  WGENASEFYPERWI-EASG--------ERKKAMLDAFAPFSVGPRACIGRNLALMQLHKTLATLFYRFSF  516
CYP53C__154264__Wco        423  WGENASEFYPERWI-EASG--------DQKKAMLDAFVPFSIGPRACIGRNLALMQLHIVLATLFHRFNV  483
CYP53c__138853__Wco        466  WGENASVFYPERWI-EASG--------DQKKAMLDAFVPFSIGPRACIGRNLALMQLHIVLATLFRRFNV  526
CYP53C__154237__Wco        335  -GENASEFYPERWV-EASG--------DQKKAMLDAFVPFSIGPRACIGCNLASMQLHTVLATLFHRFNV  394
CYP53C__112429__Csu        471  WGHDAEFFRPERWL-EADK--------EKRDAMNKAFVPFSVGPRACVGRNLAKMQLLVNIATVFRLYDI  531
CYP53C__151209__Csu        471  WAPDPDAYRPERWI-EADK--------EKREAMNKAFIPFSVGPRACVGRNLAKIQLLINVATIFRCYDV  531
CYP53C__118598__Csu        488  WGLDCEEFRPERWL-EADR--------EQQELMKKAFIPFSVGPRACVGRNLAKMQLLINIATIFRLYKV  548
CYP53C__101826__Pca        428  WGDDADEFNPDRWT-RGDR-----------NMMLKYFAPFSIGPRACIGRNLAMMEMTICIATMFHRYRL  485
CYP53C__256510__Pca        445  WGDDADEFNPDRWA-RGDR-----------KAMLKYFAPFSTGPRACVGRNLATMEMTICIATIFHRYRV  502
CYP53C__212559__Pca        456  WGDDADQFNPDRWT-LGDR-----------NAMLKYFSPFSVGPRACIGRNLAMMELTICIATIFHRYRV  513
CYP53C__102576__Pca        457  WGDDADEFNPDRWT-RGDR-----------NMMLKYFAPFSIGPRACIGRNLAMMEMTICIATIVHRYRL  514
CYP53C__212558__Pca        284  WGDDADEFNPDRWTRRGDR-----------NAILKYLAPFSIGPRACIGRNLVMMEMTICVATISHPYRL  342
CYP53__1116154__Fpi        408  Y-KDAAEFRPFRFS-DVREKGGADAQKQQFHIPTAEYIAFGHGKHACSGRWFAAAEVKAILAYILLNYDF  475
Consensus aa:                   WG.Dhp.FpP-RWh.-.s...........phh.ch@ssFShGPRAClGRsLA.hpl.lhltolh+pYch
Consensus ss:                       hh                       hh            hhhhhhhhhhhhhhheee


Conservation:                    9
CYP53C__1025718__Fpi_      507  VLEHPEQG----------------------------------------------------  514
Cyp53C__68781__Pst         509  VLEHPEKP----------------------------------------------------  516
CYP53C__80617__Gtr         506  VLEEPEKK----------------------------------------------------  513
CYP53C__127772__Cpu        530  VLEDAAKP----------------------------------------------------  537
CYP53C__94174__Shi         511  VLEDPTKP----------------------------------------------------  518
CYP53c__27029__Wco         508  VLEDADKK----------------------------------------------------  515
CYP53C3__110015__Ppl       507  VLEEPKHK----------------------------------------------------  514
CYP53C__116910__Csu        504  VLEDPEKH----------------------------------------------------  511
CYP53C__1179842__Sla       512  VLADPEKP----------------------------------------------------  519
CYP53C__55106__Dsq         510  VLENPNAQ----------------------------------------------------  517
CYP53C4__GL08839-P1.1__Gl  512  VLEDPTAE----------------------------------------------------  519
```

```
CYP53C4__47512__Gsp        510  VLEDPAAE--------------------------------------------------------------  517
CYP53C__129211__Tve        510  VLEDPSAP--------------------------------------------------------------  517
CYP53C9__27837__Pbr        508  VLENPSKP--------------------------------------------------------------  515
CYP53C7__118978__Bad       511  VLADPEKP--------------------------------------------------------------  518
CYP53C__183109__Pca        506  VLEEPDKP--------------------------------------------------------------  513
CYP53C2__130996__Pch       491  VLEDPDKP--------------------------------------------------------------  498
CYP53C__128292__Fme        519  VLEKPDEQ--------------------------------------------------------------  526
Cyp53C__37267__Tve         508  VLENPDEP--------------------------------------------------------------  515
CYP53C__70450__Ade         498  VLESDDEA--------------------------------------------------------------  505
CYP53C__52716__Dsp         509  ALLSQDQP--------------------------------------------------------------  516
CYP53C__83844__Cpu         510  VLKNPGDA--------------------------------------------------------------  517
CYP53C__194303__Abi        511  VLKDEDKP--------------------------------------------------------------  518
CYP53C__194181__Abi        515  VLEDPDLI--------------------------------------------------------------  522
CYP53C__143663__Fme        519  ILEEPNKP--------------------------------------------------------------  526
CYP53C__162664__Fme        530  VLEGVDKPEYACMPPNHVSISDPEALHVVYGHGSRTLKSDYYEVFNSVRPSIFSTRSRTEHARKRKAISH  599
CYP53C__154594__Fme        514  VLEEPDKP--------------------------------------------------------------  521
CYP53C__149618__Fme        521  ELEAPDKP--------------------------------------------------------------  528
CYP53C__130308__Fme        520  VLEEPHKP--------------------------------------------------------------  527
CYP53C__24265__Fme         518  VLEEQNKP--------------------------------------------------------------  525
CYP53C__94457__Fme         521  VLEQPDKP--------------------------------------------------------------  528
CYP53C__115179__Fme        524  VLEEPENP--------------------------------------------------------------  531
CYP53C1__5__Uma_           505  VLAEPDKP--------------------------------------------------------------  512
CYP53C__48859__Fpi         525  TLQSD-EP--------------------------------------------------------------  531
CYP53C__86809__Fpi         525  ALQSD-EP--------------------------------------------------------------  531
CYP53C__138909__Wco        527  VLESD-DP--------------------------------------------------------------  533
CYP53C__104840__Wco        529  VLESH-DP--------------------------------------------------------------  535
CYP53C__77097__Wco         533  VLESDYS---------------------------------------------------------------  539
CYP53C__138864__Wco        527  VLESD-DP--------------------------------------------------------------  533
CYP53C__104855__Wco        517  VLESS-DPVRLRSCIRF-----------------------------------------------------  532
CYP53C__154264__Wco        484  VLESD-DP--------------------------------------------------------------  490
CYP53c__138853__Wco        527  VLESD-DP--------------------------------------------------------------  533
CYP53C__154237__Wco        395  VLESD-DP--------------------------------------------------------------  401
CYP53C__112429__Csu        532  VLENPDLP--------------------------------------------------------------  539
CYP53C__151209__Csu        532  VLEKPHDP--------------------------------------------------------------  539
CYP53C__118598__Csu        549  VLADQDKE--------------------------------------------------------------  556
CYP53C__101826__Pca        486  VLASPDQQ--------------------------------------------------------------  493
CYP53C__256510__Pca        503  VLASPDQQ--------------------------------------------------------------  510
CYP53C__212559__Pca        514  VLASPDQR--------------------------------------------------------------  521
CYP53C__102576__Pca        515  VLANPDQQ--------------------------------------------------------------  522
CYP53C__212558__Pca        343  VLANPDQQ--------------------------------------------------------------  350
CYP53__1116154__Fpi        476  KLEKPGGRP-------------------------------------------------------------  484
Consensus_aa:                   lL.ps.p...............................................................
Consensus_ss:                   ee


Conservation:
CYP53C__1025718__Fpi_           ----------------------------------------------------------------------
Cyp53C__68781__Pst              ----------------------------------------------------------------------
CYP53C__80617__Gtr              ----------------------------------------------------------------------
CYP53C__127772__Cpu             ----------------------------------------------------------------------
CYP53C__94174__Shi              ----------------------------------------------------------------------
```

CYP53c__27029__Wco		--	
CYP53C3__110015__Ppl		--	
CYP53C__116910__Csu		--	
CYP53C__1179842__Sla		--	
CYP53C__55106__Dsq		--	
CYP53C4__GL08839-P1.1__Gl		--	
CYP53C4__47512__Gsp		--	
CYP53C__129211__Tve		--	
CYP53C9__27837__Pbr		--	
CYP53C7__118978__Bad		--	
CYP53C__183109__Pca		--	
CYP53C2__130996__Pch		--	
CYP53C__128292__Fme		--	
Cyp53C__37267__Tve		--	
CYP53C__70450__Ade		--	
CYP53C__52716__Dsp		--	
CYP53C__83844__Cpu		--	
CYP53C__194303__Abi		--	
CYP53C__194181__Abi		--	
CYP53C__143663__Fme		--	
CYP53C__162664__Fme	600	AFSLKSVLEFEPYIHIHITFFLGQLDKLCDGKLNIPLDTEDDMCWEGQGGRVSFDIMPWLNYLAFDIIGA	669
CYP53C__154594__Fme		--	
CYP53C__149618__Fme		--	
CYP53C__130308__Fme		--	
CYP53C__24265__Fme		--	
CYP53C__94457__Fme		--	
CYP53C__115179__Fme		--	
CYP53C1__5__Uma_		--	
CYP53C__48859__Fpi		--	
CYP53C__86809__Fpi		--	
CYP53C__138909__Wco		--	
CYP53C__104840__Wco		--	
CYP53C__77097__Wco		--	
CYP53C__138864__Wco		--	
CYP53C__104855__Wco		--	
CYP53C__154264__Wco		--	
CYP53c__138853__Wco		--	
CYP53C__154237__Wco		--	
CYP53C__112429__Csu		--	
CYP53C__151209__Csu		--	
CYP53C__118598__Csu		--	
CYP53C__101826__Pca		--	
CYP53C__256510__Pca		--	
CYP53C__212559__Pca		--	
CYP53C__102576__Pca		--	
CYP53C__212558__Pca		--	
CYP53__1116154__Fpi		--	
Consensus aa:		....	
Consensus ss:			

```
Conservation:
CYP53C__1025718__Fpi_                ----------------------------------------------------------------------
Cyp53C__68781__Pst                   ----------------------------------------------------------------------
CYP53C__80617__Gtr                   ----------------------------------------------------------------------
CYP53C__127772__Cpu                  ----------------------------------------------------------------------
CYP53C__94174__Shi                   ----------------------------------------------------------------------
CYP53c__27029__Wco                   ----------------------------------------------------------------------
CYP53C3__110015__Ppl                 ----------------------------------------------------------------------
CYP53C__116910__Csu                  ----------------------------------------------------------------------
CYP53C__1179842__Sla                 ----------------------------------------------------------------------
CYP53C__55106__Dsq                   ----------------------------------------------------------------------
CYP53C4__GL08839-P1.1__Gl            ----------------------------------------------------------------------
CYP53C4__47512__Gsp                  ----------------------------------------------------------------------
CYP53C__129211__Tve                  ----------------------------------------------------------------------
CYP53C9__27837__Pbr                  ----------------------------------------------------------------------
CYP53C7__118978__Bad                 ----------------------------------------------------------------------
CYP53C__183109__Pca                  ----------------------------------------------------------------------
CYP53C2__130996__Pch                 ----------------------------------------------------------------------
CYP53C__128292__Fme                  ----------------------------------------------------------------------
Cyp53C__37267__Tve                   ----------------------------------------------------------------------
CYP53C__70450__Ade                   ----------------------------------------------------------------------
CYP53C__52716__Dsp                   ----------------------------------------------------------------------
CYP53C__83844__Cpu                   ----------------------------------------------------------------------
CYP53C__194303__Abi                  ----------------------------------------------------------------------
CYP53C__194181__Abi                  ----------------------------------------------------------------------
CYP53C__143663__Fme                  ----------------------------------------------------------------------
CYP53C__162664__Fme             670  CVWISIRYVRNASNTAPVAVDQKACLARRSKIGADTDSFESEHIEVKEIERFSGRTPGEFTVAAVAKRLA  739
CYP53C__154594__Fme                  ----------------------------------------------------------------------
CYP53C__149618__Fme                  ----------------------------------------------------------------------
CYP53C__130308__Fme                  ----------------------------------------------------------------------
CYP53C__24265__Fme                   ----------------------------------------------------------------------
CYP53C__94457__Fme                   ----------------------------------------------------------------------
CYP53C__115179__Fme                  ----------------------------------------------------------------------
CYP53C1__5__Uma_                     ----------------------------------------------------------------------
CYP53C__48859__Fpi                   ----------------------------------------------------------------------
CYP53C__86809__Fpi                   ----------------------------------------------------------------------
CYP53C__138909__Wco                  ----------------------------------------------------------------------
CYP53C__104840__Wco                  ----------------------------------------------------------------------
CYP53C__77097__Wco                   ----------------------------------------------------------------------
CYP53C__138864__Wco                  ----------------------------------------------------------------------
CYP53C__104855__Wco                  ----------------------------------------------------------------------
CYP53C__154264__Wco                  ----------------------------------------------------------------------
CYP53c__138853__Wco                  ----------------------------------------------------------------------
CYP53C__154237__Wco                  ----------------------------------------------------------------------
CYP53C__112429__Csu                  ----------------------------------------------------------------------
CYP53C__151209__Csu                  ----------------------------------------------------------------------
CYP53C__118598__Csu                  ----------------------------------------------------------------------
CYP53C__101826__Pca                  ----------------------------------------------------------------------
CYP53C__256510__Pca                  ----------------------------------------------------------------------
CYP53C__212559__Pca                  ----------------------------------------------------------------------
CYP53C__102576__Pca                  ----------------------------------------------------------------------
```

```
CYP53C__212558__Pca              ----------------------------------------------------------------------
CYP53__1116154__Fpi              ----------------------------------------------------------------------
Consensus aa:                    ......................................................................
Consensus_ss:


Conservation:
CYP53C__1025718__Fpi_            ----------------------------------------------------------------------
Cyp53C__68781__Pst               ----------------------------------------------------------------------
CYP53C__80617__Gtr               ----------------------------------------------------------------------
CYP53C__127772__Cpu              ----------------------------------------------------------------------
CYP53C__94174__Shi               ----------------------------------------------------------------------
CYP53c__27029__Wco               ----------------------------------------------------------------------
CYP53C3__110015__Ppl             ----------------------------------------------------------------------
CYP53C__116910__Csu              ----------------------------------------------------------------------
CYP53C__1179842__Sla             ----------------------------------------------------------------------
CYP53C__55106__Dsq               ----------------------------------------------------------------------
CYP53C4__GL08839-P1.1__Gl        ----------------------------------------------------------------------
CYP53C4__47512__Gsp              ----------------------------------------------------------------------
CYP53C__129211__Tve              ----------------------------------------------------------------------
CYP53C9__27837__Pbr              ----------------------------------------------------------------------
CYP53C7__118978__Bad             ----------------------------------------------------------------------
CYP53C__183109__Pca              ----------------------------------------------------------------------
CYP53C2__130996__Pch             ----------------------------------------------------------------------
CYP53C__128292__Fme              ----------------------------------------------------------------------
Cyp53C__37267__Tve               ----------------------------------------------------------------------
CYP53C__70450__Ade               ----------------------------------------------------------------------
CYP53C__52716__Dsp               ----------------------------------------------------------------------
CYP53C__83844__Cpu               ----------------------------------------------------------------------
CYP53C__194303__Abi              ----------------------------------------------------------------------
CYP53C__194181__Abi              ----------------------------------------------------------------------
CYP53C__143663__Fme              ----------------------------------------------------------------------
CYP53C__162664__Fme         740  FPTERANVLSNLFLTKDEQGRLPSREDLTQDAITQLVAGSDTDSSKSVKACQAPDGAERCKGLPRVIGES  809
CYP53C__154594__Fme              ----------------------------------------------------------------------
CYP53C__149618__Fme              ----------------------------------------------------------------------
CYP53C__130308__Fme              ----------------------------------------------------------------------
CYP53C__24265__Fme               ----------------------------------------------------------------------
CYP53C__94457__Fme               ----------------------------------------------------------------------
CYP53C__115179__Fme              ----------------------------------------------------------------------
CYP53C1__5__Uma_                 ----------------------------------------------------------------------
CYP53C__48859__Fpi               ----------------------------------------------------------------------
CYP53C__86809__Fpi               ----------------------------------------------------------------------
CYP53C__138909__Wco              ----------------------------------------------------------------------
CYP53C__104840__Wco              ----------------------------------------------------------------------
CYP53C__77097__Wco               ----------------------------------------------------------------------
CYP53C__138864__Wco              ----------------------------------------------------------------------
CYP53C__104855__Wco              ----------------------------------------------------------------------
CYP53C__154264__Wco              ----------------------------------------------------------------------
CYP53c__138853__Wco              ----------------------------------------------------------------------
CYP53C__154237__Wco              ----------------------------------------------------------------------
CYP53C__112429__Csu              ----------------------------------------------------------------------
```

```
CYP53C__151209__Csu              ----------------------------------------------------------------------
CYP53C__118598__Csu              ----------------------------------------------------------------------
CYP53C__101826__Pca              ----------------------------------------------------------------------
CYP53C__256510__Pca              ----------------------------------------------------------------------
CYP53C__212559__Pca              ----------------------------------------------------------------------
CYP53C__102576__Pca              ----------------------------------------------------------------------
CYP53C__212558__Pca              ----------------------------------------------------------------------
CYP53__1116154__Fpi              ----------------------------------------------------------------------
Consensus_aa:                    ......................................................................
Consensus_ss:


Conservation:
CYP53C__1025718__Fpi_            ----------------------------------------------------------------------
Cyp53C__68781__Pst               ----------------------------------------------------------------------
CYP53C__80617__Gtr               ----------------------------------------------------------------------
CYP53C__127772__Cpu              ----------------------------------------------------------------------
CYP53C__94174__Shi               ----------------------------------------------------------------------
CYP53c__27029__Wco               ----------------------------------------------------------------------
CYP53C3__110015__Ppl             ----------------------------------------------------------------------
CYP53C__116910__Csu              ----------------------------------------------------------------------
CYP53C__1179842__Sla             ----------------------------------------------------------------------
CYP53C__55106__Dsq               ----------------------------------------------------------------------
CYP53C4__GL08839-P1.1__Gl        ----------------------------------------------------------------------
CYP53C4__47512__Gsp              ----------------------------------------------------------------------
CYP53C__129211__Tve              ----------------------------------------------------------------------
CYP53C9__27837__Pbr              ----------------------------------------------------------------------
CYP53C7__118978__Bad             ----------------------------------------------------------------------
CYP53C__183109__Pca              ----------------------------------------------------------------------
CYP53C2__130996__Pch             ----------------------------------------------------------------------
CYP53C__128292__Fme              ----------------------------------------------------------------------
Cyp53C__37267__Tve               ----------------------------------------------------------------------
CYP53C__70450__Ade               ----------------------------------------------------------------------
CYP53C__52716__Dsp               ----------------------------------------------------------------------
CYP53C__83844__Cpu               ----------------------------------------------------------------------
CYP53C__194303__Abi              ----------------------------------------------------------------------
CYP53C__194181__Abi              ----------------------------------------------------------------------
CYP53C__143663__Fme              ----------------------------------------------------------------------
CYP53C__162664__Fme          810 GLTVCGKRFPEGTCVSVPSYTIHRDPLVWGEDVDVFRPERWFERDPSIMQNAFNAFSFGPRFYDIILEEP  879
CYP53C__154594__Fme              ----------------------------------------------------------------------
CYP53C__149618__Fme              ----------------------------------------------------------------------
CYP53C__130308__Fme              ----------------------------------------------------------------------
CYP53C__24265__Fme               ----------------------------------------------------------------------
CYP53C__94457__Fme               ----------------------------------------------------------------------
CYP53C__115179__Fme              ----------------------------------------------------------------------
CYP53C1__5__Uma_                 ----------------------------------------------------------------------
CYP53C__48859__Fpi               ----------------------------------------------------------------------
CYP53C__86809__Fpi               ----------------------------------------------------------------------
CYP53C__138909__Wco              ----------------------------------------------------------------------
CYP53C__104840__Wco              ----------------------------------------------------------------------
CYP53C__77097__Wco               ----------------------------------------------------------------------
```

```
CYP53C__138864__Wco              -----------------------------------------------------------------------
CYP53C__104855__Wco         533  ----------------------------------------------------------------CSTADSV  539
CYP53C__154264__Wco              -----------------------------------------------------------------------
CYP53c__138853__Wco              -----------------------------------------------------------------------
CYP53C__154237__Wco              -----------------------------------------------------------------------
CYP53C__112429__Csu              -----------------------------------------------------------------------
CYP53C__151209__Csu              -----------------------------------------------------------------------
CYP53C__118598__Csu              -----------------------------------------------------------------------
CYP53C__101826__Pca              -----------------------------------------------------------------------
CYP53C__256510__Pca              -----------------------------------------------------------------------
CYP53C__212559__Pca              -----------------------------------------------------------------------
CYP53C__102576__Pca              -----------------------------------------------------------------------
CYP53C__212558__Pca              -----------------------------------------------------------------------
CYP53__1116154__Fpi              -----------------------------------------------------------------------
Consensus_aa:                    .......................................................................
Consensus_ss:


Conservation:                            5   56   5 58    7
CYP53C__1025718__Fpi_       515  ---LDTKEGFLRKPVECRVG-IKRRTV---------  537
Cyp53C__68781__Pst          517  ---FDTREGFLRKPVECKVG-IKRRSA---------  539
CYP53C__80617__Gtr          514  ---FETREGFLRKPVECRVG-IKRRN----------  535
CYP53C__127772__Cpu         538  ---FDTREGFLRKPVECRVG-IRRRV----------  559
CYP53C__94174__Shi          519  ---FATKEGFLRKPVDCKIG-IKRRNL---------  541
CYP53c__27029__Wco          516  ---FDTREGFLRKPVECKVG-MKRRSL---------  538
CYP53C3__110015__Ppl        515  ---LETKEGFLRKPVACKVG-LRRRSA---------  537
CYP53C__116910__Csu         512  ---FDTREGFLRKPVECRVG-IKRRHD---------  534
CYP53C__1179842__Sla        520  ---FDTREGFLRKPQECRVG-IKRRQT---------  542
CYP53C__55106__Dsq          518  ---LETREGFLRKPVECKVG-MKQRHT---------  540
CYP53C4__GL08839-P1.1__Gl   520  ---LDTREGFLRKPVECKVG-MKRRNV---------  542
CYP53C4__47512__Gsp         518  ---LDTREGFLRKPVECKVG-MKRRNA---------  540
CYP53C__129211__Tve         518  ---LATNEGFLRKPLKCIVG-MKRRNV---------  540
CYP53C9__27837__Pbr         516  ---LETLEGFLRKPVDCVVG-IRRRSL---------  538
CYP53C7__118978__Bad        519  ---FDTAEGFLRKPVDCQVG-IKKRAN---------  541
CYP53C__183109__Pca         514  ---FDTMEGFLRKPVECLVG-IKRRSL---------  536
CYP53C2__130996__Pch        499  ---FDTMEGFLRKPVECVVG-IRRRTL---------  521
CYP53C__128292__Fme         527  ---FDTREGFLRKPLRCRVG-MRRREL---------  549
Cyp53C__37267__Tve          516  ---LPTNEGFLRKPLKCNVG-MQRRNV---------  538
CYP53C__70450__Ade          506  ---SQTREGFLRKPVLCRIG-LKRRSPV--------  529
CYP53C__52716__Dsp          517  ---LETREGFLRKPVSCYVG-MKRRST---------  539
CYP53C__83844__Cpu          518  ---LGTCEGFLRKPTDCWVG-LRRRAL---------  540
CYP53C__194303__Abi         519  ---LVISEGFLRKPLSVDLG-IKRRDV---------  541
CYP53C__194181__Abi         523  ---LDTREGFLRKPLACRVG-IKRRDI---------  545
CYP53C__143663__Fme         527  ---LEIHEAFMRKPVACHVG-LKRRGA---------  549
CYP53C__162664__Fme         880  DKSLEIHKTFIRRPIACHIG-LKRQYD---------  905
CYP53C__154594__Fme         522  ---LETHEGFIRKPLTCRVG-MKRRDV---------  544
CYP53C__149618__Fme         529  ---LTIREDFIRKPVACRVG-MKRRNI---------  551
CYP53C__130308__Fme         528  ---LEVHEGFIRKPMACRVG-MKRRNV---------  550
CYP53C__24265__Fme          526  ---LEVHEGFIRKPMACRVG-MKRRNV---------  548
CYP53C__94457__Fme          529  ---LEVHDAFARKPNSCRIG-LKRRDV---------  551
CYP53C__115179__Fme         532  ---VEVFEGFIMRPKSCRVG-MKRRAI---------  554
```

```
CYP53C1__5__Uma_         513  ---LDTFEGFLRKPVKLEVG-LKRRN----------  534
CYP53C__48859__Fpi       532  ---LRVRDSLARRPLECMVG-ITRRKGELGYGSFEL  563
CYP53C__86809__Fpi       532  ---LRVRDSFARRPQECMVG-ITRRK----------  553
CYP53C__138909__Wco      534  ---LPVQDSFVRKPKRCMIG-IKSRKL---------  556
CYP53C__104840__Wco      536  ---LPVQDSFVRKPKRCVIG-IKSRKL---------  558
CYP53C__77097__Wco       540  ---LPVQDSFVRKPRWCAIG-IKLRKL---------  562
CYP53C__138864__Wco      534  ---LHVQDSLIRKPKWCMIG-IKSRKL---------  556
CYP53C__104855__Wco      540  RNQLPVQDGFARKPRRCMIG-IRSRKL---------  565
CYP53C__154264__Wco      491  ---VRSQAYALYVAGHAAMG-IEAVMLRQKEF----  518
CYP53c__138853__Wco      534  ---LPVQDSFGRRPKQCMVG-IKPRNL---------  556
CYP53C__154237__Wco      402  ---VRSQV----------------------------  406
CYP53C__112429__Csu      540  ---LPVHDNFIRKPLNCHVG-VKRRS----------  561
CYP53C__151209__Csu      540  ---MPVHDDFTRKVIECFIG-LKRREI---------  562
CYP53C__118598__Csu      557  ---LEVFDNFVRKPLNCRVG-MQRRDLSSKREWSI-  587
CYP53C__101826__Pca      494  ---LECSEGFVSKPKDVYVG-MQRRV----------  515
CYP53C__256510__Pca      511  ---LECHEGLVRKPNSVPVG-MRRRV----------  532
CYP53C__212559__Pca      522  -----VSQLFNVLDKDVG------------------  534
CYP53C__102576__Pca      523  ---LECSEGFVRKPKNVHVG-MQRRL----------  544
CYP53C__212558__Pca      351  ---LECSEGFVRKPKNVHVGSMQRRL----------  373
CYP53__1116154__Fpi      485  -ENMNLGPSILPHP-RAKVM-FRKRKASRA------  511
Consensus_aa:                 ...hph.pthlp+P.ph.lG.hc+R.h.........
Consensus_ss:                         eeee    eeee eee
```

References:

Jones DT (1999) Protein secondary structure prediction based on position-specific scoring matrices. J Mol Biol 292:195-202.
